# Palladium-catalyzed highly regioselective mono and double Sonogashira cross-coupling reactions of 5-substituted-1,2,3-triiodobenzene under ambient conditions†

**DOI:** 10.1039/d0ra01569e

**Published:** 2020-04-24

**Authors:** Raed M. Al-Zoubi, Mothana K. Al-Omari, Walid K. Al-Jammal, Michael J. Ferguson

**Affiliations:** Department of Chemistry, Jordan University of Science and Technology P. O. Box 3030 Irbid 22110 Jordan rmzoubi@just.edu.jo +962-2-7201071 +962-2-7201000-ext. 23651; Department of Chemistry, Gunning-Lemieux Chemistry Centre, University of Alberta Edmonton Alberta T6G2G2 Canada

## Abstract

An efficient synthesis of 2,3-diiodinated diphenylacetylene and iodinated *meta*-terphenylacetylene derivatives through highly regioselective mono and double Sonogashira cross-coupling reactions of 5-substituted-1,2,3-triiodobenzene is reported. Significantly, the regioselectivity of coupling reactions is exclusively performed at the terminal C–I bonds, the less sterically hindered and the most regioactive positions. The highest isolated yields were achieved from reactions of electron-poor/neutral 1,2,3-triiodoarene and electron-rich arylacetylene derivatives. The use of 2.0 equiv. of arylacetylenes in one-pot fashion afforded the iodinated *meta*-terphenylacetylenes in excellent site selectivity and in good isolated yields. Different functional groups were found to be suitable under optimized conditions. This report discloses the first method to synthesize hitherto unknown 2,3-diiodinated diphenylacetylenes and iodinated *meta*-terphenylacetylenes that is facile, highly regioselective, general in scope and produces remarkable building blocks for other chemical transformations.

## Abstract 2

Efficient and highly regioselective synthesis of 2,3-diiodinated diphenylacetylene and iodinated *meta*-terphenylacetylene derivatives *via* mono and double Sonogashira cross-coupling reactions.

## Graphical abstract

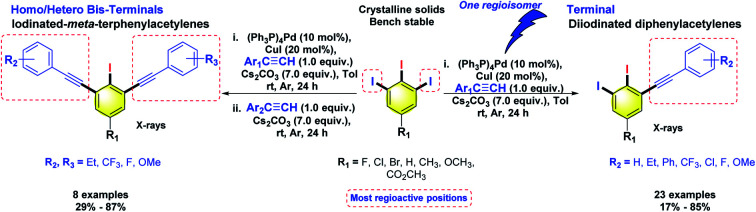

## Introduction

**Fig. 1:**
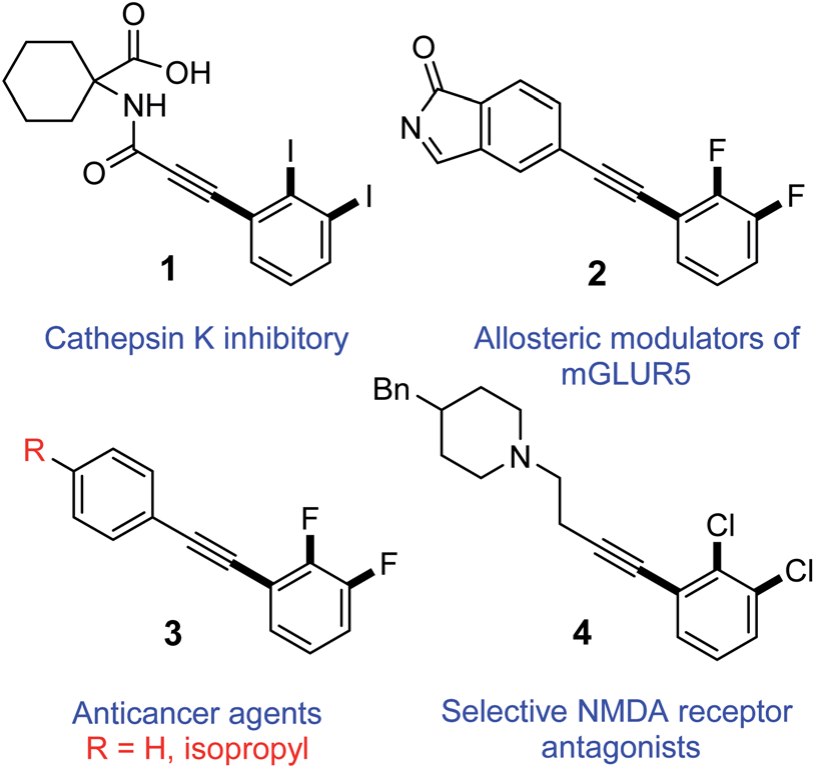
Some biologically active 2,3-dihalogenated phenylacetylene compounds in medicine.

Harnessing the regioselectivity to quickly access highly functionalized molecules by means of site-selective functional group transformation from adaptable building blocks is essential in synthetic chemistry and biology. Inspired by the broad biological activities of 2,3-dihalogenated phenylacetylene derivatives,^1–5^ only limited synthetic protocols have been reported and none for diiodinated motifs thus far.^6–14^ For instance, a 2,3-diiodinated phenylacetylene derivative (1, Fig. 1) is reported to have cathepsin K inhibitory action.^15^ Additionally, 2,3-difluorinated diphenylacetylene derivative 2, is reported as allosteric modulator for metabotropic glutamate receptor subtype 5 (mGluR5) for treating neural and psychiatric disorders associated with glutamate dysfunction.^3^ Furthermore, another 2,3-difluorinated diphenylacetylene derivative (3, Fig. 1) are reported to inhibit the proliferation of LS174T colon cancer by inhibition of c-myc and induction of the cyclin-dependent kinase inhibitor.^2,16^ Lastly, 2,3-dichlorinated phenylacetylene derivative (4, Fig. 1), is found to be useful as selectively active antagonists of *N*-methyl-d-aspartate (NMDA) receptor subtypes for treating conditions such as central nervous system trauma, hypoglycemia, anxiety, stroke, convulsions, cerebral ischemia, chronic pain or neurodegenerative disorders as Alzheimer's Disease, Parkinsonism and Huntington's disease.^5^

The status quo of these 2,3-dihalogenated phenylacetylenes and other derivatives^17,18^ with their remarkable applications in medicine encouraged us for developing a new method to access 2,3-diiodinated phenylacetylene molecules. Although few reports for the synthesis of 2,3-dihalogenated phenylacetylenes were published, no protocol for the synthesis of 2,3-diiodinated diphenylacetylene motifs is reported to date. Herein, we report the first synthetic method to access hitherto unknown 2,3-diiodinated diphenylacetylene derivatives by regioselective Sonogashira cross-coupling of 5-substituted-1,2,3-triiodobenzene that is efficient, scalable and affords moderate to good yields.

A broad functional groups were examined under the optimized reaction conditions and found tolerant providing the desired terminal coupling products in highly regioselective manner that are indeed difficult to make by alternative means.

## Results and discussion

Considering the remarkable bioactivities of many 2,3-dihalogenated phenylacetylene derivatives in literature and also to our finding in regioselective Suzuki–Miyaura cross-coupling of 5-substituted-1,2,3-triiodobenzene,^19^ we felt impelled to examine the Sonogashira cross-couplings on 5-substituted-1,2,3-triiodobenzenes aiming forward to diiodinated phenylacetylene derivatives. 5-Substituted-1,2,3-triiodobenzene starting materials were prepared according to our previous procedures from anilines or benzoic acids.^20–22^

**Scheme 1:**
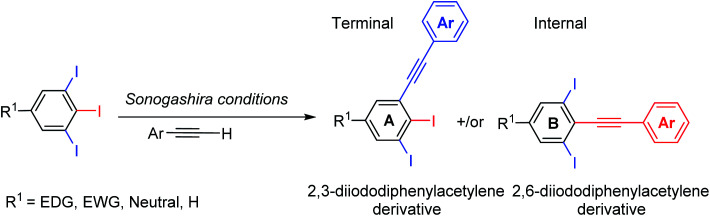
Possible diiodinated diphenylacetylene regioisomers from Sonogashira cross-coupling of 5-substituted-1,2,3-triiodoarenes.

Several regioselective Sonogashira of polyhalogenated arene systems are found in literature.^23–32^ For instance, Langer and co-workers published the synthesis of quinolino[3′,4′:4,5]pyrrolo[1,2-*f*]phenanthridines *via* regioselective Sonogashira of 4-chloro-3-iodo-2-methylquinoline with different aryl acetylenes in excellent regioselectivity towards the iodo substituent in moderate to good yields.^23^ Usuki and co-workers reported the total synthesis of desmosine, a biomarker and elastin cross-linker, in 13 steps and 11% overall yield through sequential regioselective Sonogashira cross-coupling reactions of 3,4,5-trihalopyridine.^25,26^ The Sonogashira cross-coupling reaction of 5-substituted-1,2,3-triiodobenzene bearing two regiochemically unsymmetrical C–I positions provides at most two possible regioisomeric coupling products, the internal and terminal diiodinated diphenylacetylenes (Scheme 1). Consequently, the satisfactory use of 1.0 mol equivalent of aryl acetylene is adequate to couple one of the three iodo substituents providing the desired diiodinated diphenylacetylene products.

**Scheme 2:**
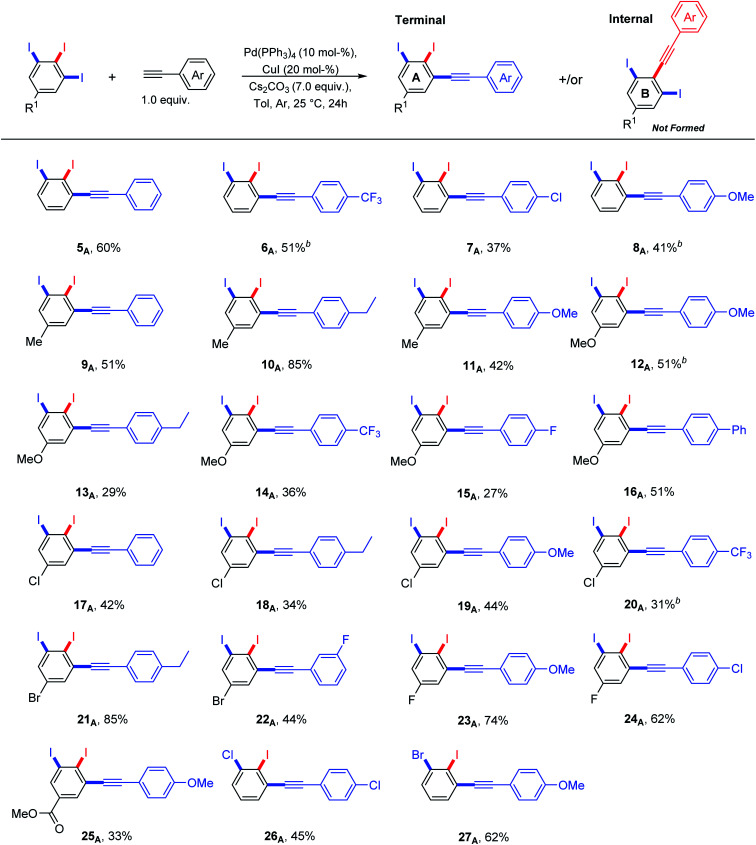
Terminal *vs.* internal diiodinated diphenylacetylene derivatives *via* regioselective Sonogashira cross-coupling of 5-substituted-1,2,3-triiodobenzenes and arylcetylenes. ^a^Yields are given for isolated compounds (reaction scale: 0.65 mmol). ^b^5–10% of *meta* bis-terminal coupling product was isolated.

A thorough optimization using 1,2,3-triiodobenzene 5 and phenylacetylene as model substrates to explore this hypothesis as shown in Table 1. Happily and as expected, the reaction was found to be highly regioselective to the terminal C–I bond, the more accessible and less hindered position, providing only the 2,3-diiododiphenylacetylene 5_A_. The order of addition proved to be essential to improve the yield and to minimize the Glaser Homo-coupling side reaction. Temperature and solvent were also found to be essential parameters in our optimization. The reaction of 1,2,3-triiodobenzene 5 with 1.2 equiv. of phenylacetylene in DMF at 100 °C provided a non clean reaction forming other side reactions and Glaser homo-coupling product (Table 1, entries 1 and 2). It is worth mentioning that due to the low polarity of the desired product, a difficult purification was observed in the presence of Glaser homo-coupling product. At elevated temperature such as 100 °C, it is not surprising that significant amount of multiple coupling reactions with other C–I bonds and other side reactions were possibly formed. Lowering the reaction temperature to 50 °C and 25 °C were found to be unsuccessful (Table 1, entry 3 and 4). Changing the base to 4-(dimethylamino)pyridine (DMAP) shutdown the reaction (Table 1, entry 5). Changing the solvent to anhydrous toluene provided the wanted product 5_A_ in 15% yield (Table 1, entry 6). Elongate reaction time to 24 hours at same temperature provided 29% (Table 1, entry 7). Increasing the Pd(PPh_3_)_4_ loading to 20 mol% gave 21% yield of the desired product with ∼5% of the *meta* bis-coupled product (Table 1, entry 8). While increasing the CuI loading to 20 mol% was found to be beneficial provided 37% yield of the desired product 5_A_ (Table 1, entry 9). The highest isolated yield for this transformation was achieved by the use of 10 mol% of Pd(PPh_3_)_4_, 20 mol% of CuI as a co-catalysis, 7.0 equiv. of Cs_2_CO_3_ as a base in anhydrous toluene at 25 °C for 24 h (Table 1, entry 10). Other conditions were found to be unsuccessful to further enhance the reaction (Table 1, entries 11–13). The coupling reaction over and above performed nicely on large scale (Table 1, entry 14). The scope of the regioselective Sonogashira reaction was then studied under the optimized conditions. Therefore, a variety of 5-substituted-1,2,3-triiodobenzene starting materials were examined under the optimized conditions providing the terminal coupling products in excellent site-selectivity (Scheme 2).

**Table 1:** Conditions for the regioselective Sonogashira cross-coupling reaction of 5-substituted-1,2,3-triiodobenzene 5 and phenylacetylene^a^

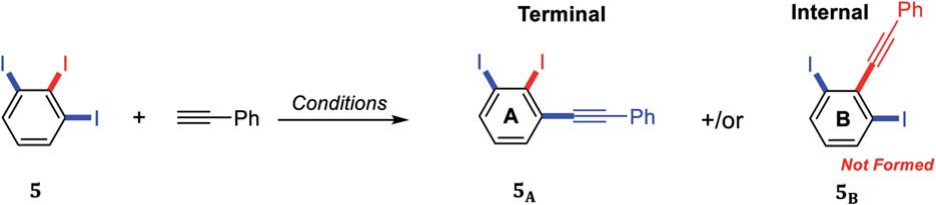							
Entry	Catalyst, co-catalyst (mol%)	Alkyne (equiv.)	Base (equiv.)	Solvent	*T* (°C)	Time (h)	% yield of 5_A_^b^ (%)
1	Pd(PPh_3_)_4_ (10%), CuI (10%)	1.2	Cs_2_CO_3_ (4)	DMF	100	12	NS^c^
2	Pd(PPh_3_)_4_ (10%), CuI (10%)	1.0	Cs_2_CO_3_ (4)	DMF	100	12	NS^c^
3	Pd(PPh_3_)_4_ (10%), CuI (10%)	1.0	Cs_2_CO_3_ (4)	DMF	50	12	NS^c^
4	Pd(PPh_3_)_4_ (10%), CuI (10%)	1.0	Cs_2_CO_3_ (4)	DMF	25	12	NS^c^
5	Pd(PPh_3_)_4_ (10%), CuI (10%)	1.0	DMAP (4)	DMF	25	12	0%
6	Pd(PPh_3_)_4_ (10%), CuI (10%)	1.0	Cs_2_CO_3_ (4)	Toluene	25	12	15%
7	Pd(PPh_3_)_4_ (10%), CuI (10%)	1.0	Cs_2_CO_3_ (4)	Toluene	25	24	29%
8	Pd(PPh_3_)_4_ (20%), CuI (10%)	1.0	Cs_2_CO_3_ (4)	Toluene	25	24	21%^d^
9	Pd(PPh_3_)_4_ (10%), CuI (20%)	1.0	Cs_2_CO_3_ (4)	Toluene	25	24	37%
**10**	**Pd(PPh**_**3**_ **)**_**4**_ **(10%), CuI (20%)**	**1.0**	**Cs**_**2**_ **CO**_**3**_ **(7)**	**Toluene**	**25**	**24**	**60%**
11	Pd(PPh_3_)_4_ (10%), CuI (20%)	1.0	Cs_2_CO_3_ (10)	Toluene	25	24	58%
12	Pd(PPh_3_)_4_ (5%), CuI (20%)	1.0	Cs_2_CO_3_ (7)	Toluene	25	24	42%
13	Pd(PPh_3_)_4_ (10%), CuI (20%)	1.0	K_2_CO_3_ (7)	Toluene	25	24	49%
14^e^	Pd(PPh_3_)_4_ (10%), CuI (20%)	1.0	Cs_2_CO_3_ (7)	Toluene	25	24	53%

a Conditions: all recations were carried out using 0.65 mmol (1.0 equiv., 0.08 M) of 1,2,3-triiodobenzene (5) in 8.0 mL anhyd. solvent.

b Isolated yield.

c NS: not separable mixture of products.

d 5% of bis-terminal coupling product was isolated.

e 1.0 gram scale (2.19 mmol).

It was found that the nature of R^1^ substituent has a big impact on the reactivity of Sonogashira cross-coupling reactions but not on regioselectivity. A combination between electron-poor/neutral 1,2,3-triiodoarenes and electron-rich arylacetylenes provided the terminal coupling products in high isolated yields (Scheme 2: 10_A_, 21_A_, 23_A_ and 24_A_). In contrast, electron-rich 1,2,3-triiodoarenes afforded moderate yields (Scheme 2: 12_A_–16_A_). The formation of the internal coupling regioisomer was not detected in all examples (Scheme 2: B). Gratefully, the coupling reaction with bromo or chloro substituents was not observed (Scheme 2: 17_A_–22_A_ and 26_A_–27_A_) and it can tolerate a wide range of functional groups. 5–10% of the *meta* bis-terminal coupling products were isolated in some cases (Scheme 2: 6_A_, 8_A_, 12_A_ and 20_A_).

**Fig. 2:**
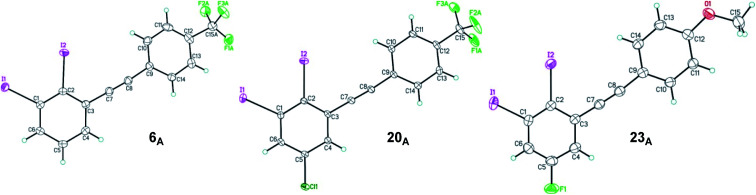
ORTEP view of 1,2-diiodo-3-((4-(trifluoromethyl)phenyl)ethynyl)benzene 6_A_, 5-chloro-1,2-diiodo-3-((4-(trifluoromethyl)phenyl)ethynyl)benzene 20_A_ and 5-fluoro-1,2-diiodo-3-((4-mehthoxyphenyl)ethynyl)benzene 23_A_. Thermal Gaussian ellipsoids at 30% probability level.

The structure of 2,3-diiodinated diarylacetylene compounds are further confirmed by X-ray diffraction methods for three coupling products, 1,2-diiodo-3-((4-(trifluoromethyl)phenyl)ethynyl)benzene 6_A_, 5-chloro-1,2-diiodo-3-((4-(trifluoromethyl)phenyl)ethynyl) benzene 20_A_ and 5-fluoro-1,2-diiodo-3-((4-methoxyphenyl)ethynyl) benzene 23_A_ as shown in Fig. 2.^33^

**Scheme 3:**
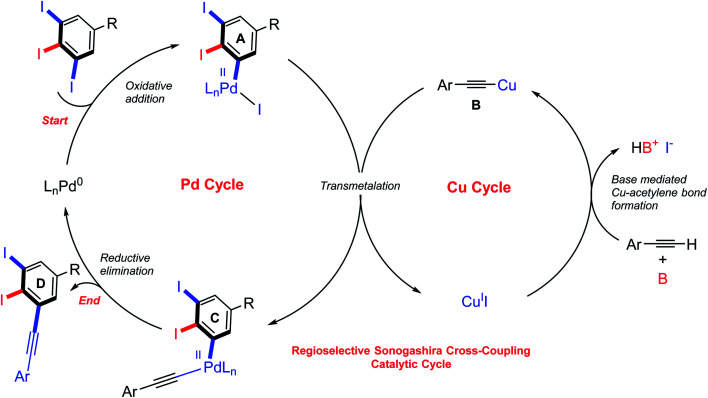
Proposed catalytic cycle for regioselective Sonogashira cross-coupling reaction of 5-substituted-1,2,3-triiodobenzene.

**Scheme 4:**
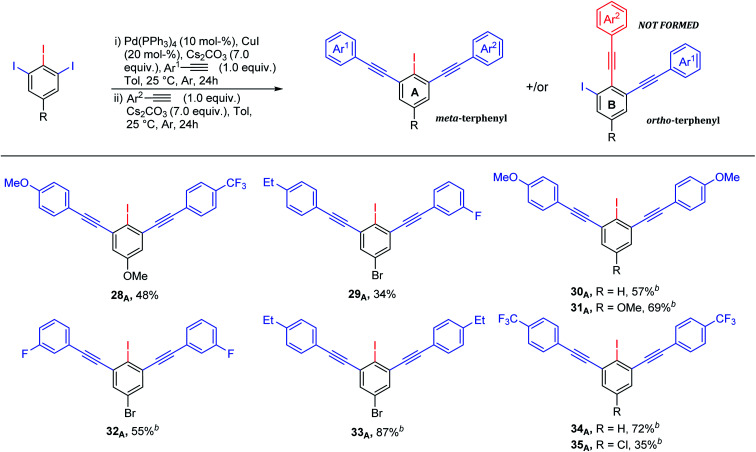
Iodinated terphenyls *via* one-pot double Sonogashira cross-coupling reaction of 1,2,3-triiodoarenes and arylacetylenes. ^a^Yields are given for isolated yields (reaction scale 0.66 mmol). ^b^2.0 equiv. of arylacetylene was used.

Although various approaches for regioselective Sonogashira cross-coupling have been previously reported,^34–46^ a plausible catalytic cycle for regioselective Sonogashira cross-coupling of 5-substituted-1,2,3-triiodobenzene is proposed in Scheme 3. Oxidative addition by Pd^0^ at the terminal C–I position, the less sterically hindered and more accessible position forming Pd^II^ intermediate A. The transmetallation of Pd^II^ intermediate A with copper(i) acetylide B, which was *in situ* generated with base and Cu^II^, to afford Pd^II^ intermediate C. Reductive elimination afforded the terminal coupled product D and regenerated the catalyst for another catalytic cycle. We then turn our attentions to examine the reactivity order of the other iodo groups under the optimized conditions. Therefore, one-pot double Sonogashira cross-coupling reactions of 5-substituted-1,2,3-triiodobenzene with 2.0 equiv. of arylacetylenes were performed. The excellent regioselectivity of the first coupling, *vide supra*, promoted the second coupling reaction to occur at the other terminal position providing exclusively the iodinated *meta*-terphenyl products in moderate to good yields with excellent regioselectivity (Scheme 4: 28_A_–35_A_). We did not observe the *ortho* bis-coupled products in all reactions. It is believed that the reaction may proceed *via* a reversible oxidative addition step.

**Fig. 3:**
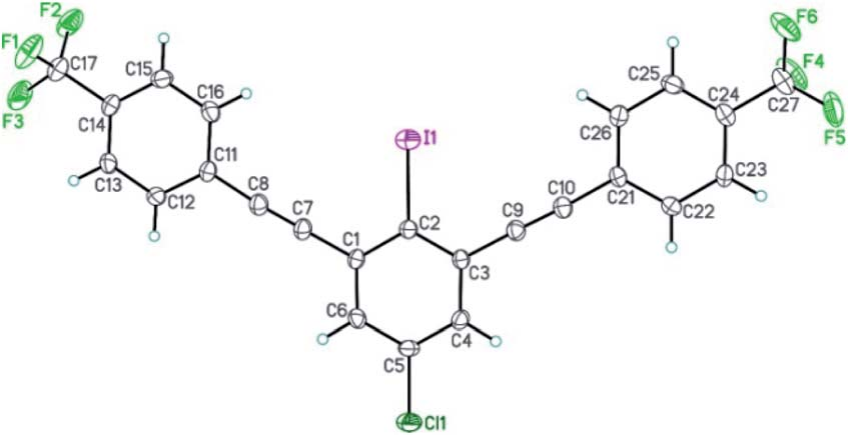
ORTEP view of 4,4′-((5-chloro-2-iodo-1,3-phenylene)bis(ethyne-2,1-diyl))bis((trifluoromethyl)benzene) 35_A_. Thermal Gaussian ellipsoids at 30% probability level.

The structure of bis-coupling products was further supported by X-ray crystallography of one of the derivative, 4,4′-((5-chloro-2-iodo-1,3-phenylene)bis(ethyne-2,1-diyl))bis((trifluoromethyl)benzene) 35_A_ as shown in Fig. 3.^33^

**Scheme 5:**
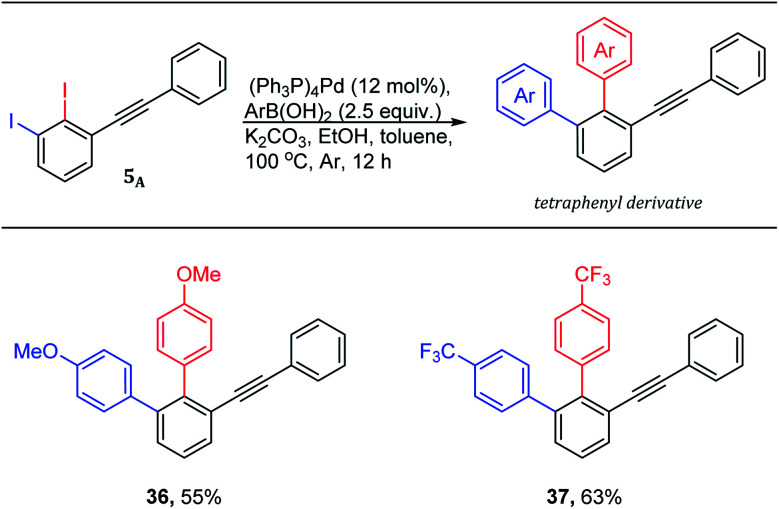
Tetraphenyls *via* one-pot double Suzuki–Miyaura cross-coupling reaction of 1,2-diiodo-3-(phenylethynyl)benzene 5_A_. ^a^Yields are given for isolated yields (reaction scale 0.66 mmol).

The quickly access of highly functionalized compounds from available starting materials is crucial in academia and industry. The 1,2,3-trisubstituted benzenes are demanded and challenging derivatives in literature. Therefore, 2,3-diiododiphenylacetylene 5_A_ was used to quickly access 1,2,3-trisubstitutedbenzene derivatives using Suzuki–Miyaura cross-coupling reactions (Scheme 5).

The use of 2.0 mol equiv. of arylboronic acid under the optimized conditions,^19^ provided the trisubstituted derivatives (Scheme 5: 36–37) through one-pot double Suzuki–Miyaura cross-coupling in good isolated yields. It is worth noting that the use of 1.0 mol equiv. of arylboronic acid under the same conditions is found to be inefficient providing a non separable mixture of ∼1 : 1 of both coupling products.

## Conclusion

In summary, we reported the first synthesis of hitherto unknown 2,3-diiodinated diphenylacetylene and iodinated *meta* terphenylacetylene derivatives *via* highly regioselective mono and double Sonogashira cross-coupling reaction from 5-substituted-1,2,3-triiodobenzene. The desired products were isolated in moderate to good yields (17–85%) and the reaction tolerated a broad range of functional groups. No coupling reaction with bromo or chloro groups was observed. The regioselectivity of coupling reactions exclusively performed at the terminal C–I bonds, the less sterically hindered and the most regioactive positions. The highest isolated yields were achieved from reactions of electron-poor/neutral 1,2,3-triiodoarene and electron-rich arylacetylene derivatives. The structure of the products was further supported by X-ray diffraction methods. Having other iodo substituents on these products, further chemical elaborations could easily be explored.

## Experimental

### General

All commercial reagents and chromatography solvents were used as obtained unless otherwise stated. Ethanol, toluene, ethyl acetate, hexanes, anhydrous sodium sulfate (Na_2_SO_4_, BDH), CuI (Sigma-Aldrich), Pd(PPh_3_)_4_ (Sigma-Aldrich) were used as received. Anhydrous solvents were distilled over appropriate drying agents prior to use. Analytical thin layer chromatography (TLC) was performed on Merck silica gel 60 F_254_. Merck silica gel 60 (0.063–0.2 mm) was used for column chromatography. Visualization of TLC was accomplished with UV light (254 nm). NMR spectra were recorded on a Bruker-Avance 400 MHz spectrometer. The residual solvent protons (^1^H) or the solvent carbon (^13^C) were used as internal standards. ^1^H-NMR data are presented as follows: chemical shift in ppm (*δ*) downfield from trimethylsilane (multiplicity, integration, coupling constant). The following abbreviations are used in reporting NMR data: s, singlet; bs, broad singlet; d, doublet; t, triplet; q, quartet; dq, doublet of quartets; dd, doublet of doublets; m, mutiplet. High resolution mass spectra were recorded using Chemical Ionization (CI) and electrospray ionization (ESI) techniques.

### General procedure for Sonogashira cross-coupling reactions of 5-substituted-1,2,3-triiodobenzenes

A flame-dried Schlenk flask was charged with 5-substituted-1,2,3-triiodobenzene (0.65 mmol, 0.08 M, 1.0 equiv.), aryl acetylene (1.0 equiv.), Cs_2_CO_3_ (7.0 equiv.) in 8.0 mL dry toluene. The mixture was stirred under argon at room temperature for 20 min. Tetrakis(triphenylphosphine)palladium (0) (10 mol%) and copper iodide (20 mol%) were added, capped with septum, carefully degassed with argon, and the reaction flask was wrapped with aluminum foil and stirred at room temperature for 24 h. The reaction mixture was then diluted with EtOAc and filtered over Celite 545®. Distilled water (100 mL) was added and extracted with EtOAc (2 × 50 mL). The organic layers were combined, washed with brine, dried with anhyd. Na_2_SO_4_, filtered and evaporated under reduced pressure. The crude product was purified by flash chromatography (100% hexane) to yield the pure desired product.

### Synthesis of 1,2-diiodo-3-(phenylethynyl)benzene (5_A_)

The title compound was synthesized using the general procedure and isolated in **60%** yield as colorless oil after flash chromatography. IR (cast film, cm^−1^) 3087, 3046, 2201, 1599, 1523, 912, 837, 749, 624. *δ*_H_ (400 MHz, CDCl_3_) *δ*: 7.81 (d, 1H, *J* = 7.9 Hz), 7.50–7.65 (m, 2H), 7.47 (d, 1H, *J* = 7.6 Hz), 7.30–7.40 (m, 3H), 7.03 (dd, 1H, *J* = 7.8 Hz). *δ*_C_ (100 MHz, CDCl_3_) *δ*: 138.9, 131.9, 131.8, 131.3, 129.2, 129.0, 128.6, 122.8, 114.7, 108.9, 93.5, 93.1. HRMS (EI) *m*/*z* for C_14_H_8_I_2_ [M]^+^: calcd 429.8715; found, 429.8709.

### Synthesis of 1,2-diiodo-3-((4-(trifluoromethyl)phenyl)ethynyl) benzene (6_A_)

The title compound was synthesized using the general procedure and isolated in **51%** yield as white solid after flash chromatography. IR (cast film, cm^−1^) 3109, 3046, 2204, 1588, 1541, 1042, 943, 812, 738, 661. *δ*_H_ (400 MHz, CDCl_3_) *δ*: 7.85 (d, 1H, *J* = 7.9 Hz), 7.68 (d, 2H, *J* = 8.0 Hz), 7.62 (d, 2H, *J* = 8.2 Hz), 7.48 (d, 1H, *J* = 7.6), 7.06 (dd, 1H, *J*^1^ = 7.8 Hz, *J*^2^ = 7.7 Hz). *δ*_C_ (100 MHz, CDCl_3_) *δ*: 139.5, 132.0, 131.6, 131.2, 130.7 (q, *J*_C–F_ = 33 Hz), 129.3, 126.6, 125.5 (q, *J*_C–F_ = 4 Hz), 124.0 (q, *J*_C–F_ = 271 Hz), 114.8, 109.1, 95.6, 91.4. Mp: 92–94 °C. HRMS (EI) *m*/*z* for C_15_H_7_F_3_I_2_ [M]^+^: calcd 497.8589; found, 497.8576.

### Synthesis of 1-((4-chlorophenyl)ethynyl)-2,3-diiodobenzene (7_A_)

The title compound was synthesized using the general procedure and isolated in **37%** yield as white solid after flash chromatography. IR (cast film, cm^−1^) 3086, 3012, 2203, 914, 824, 748, 638. *δ*_H_ (400 MHz, CDCl_3_) *δ*: 7.81 (d, 1H, *J* = 7.8 Hz), 7.40–7.60 (m, 3H), 7.32 (d, 2H, *J* = 8.4 Hz), 7.03 (dd, 1H, *J* = 7.8 Hz, *J* = 7.7 Hz). *δ*_C_ (100 MHz, CDCl_3_) *δ*: 139.2, 135.2, 133.0, 131.6, 131.4, 129.2, 129.0, 121.2, 114.7, 109.0, 94.4, 91.9. Mp: 101–103 °C. HRMS (EI) *m*/*z* for C_14_H_7_ClI_2_ [M]^+^: calcd 463.8326; found, 463.8317.

### Synthesis of 1,2-diiodo-3-((4-methoxyphenyl)ethynyl)benzene (8_A_)

The title compound was synthesized using the general procedure and isolated in **41%** yield as white solid after flash chromatography. IR (cast film, cm^−1^) 3087, 3024, 2215, 1606, 1546, 1324, 1157, 973, 843, 642. *δ*_H_ (400 MHz, CDCl_3_) *δ*: 7.78 (d, 1H, *J* = 7.8 Hz), 7.51 (d, 2H, *J* = 8.7 Hz), 7.44 (d, 1H, *J* = 7.6 Hz), 7.02 (dd, 1H, *J*^1^ = 7.7 Hz,, *J*^2^ = 7.8 Hz), 6.89 (d, 2H, *J* = 8.7 Hz). *δ*_C_ (100 MHz, CDCl_3_) *δ*: 160.3, 138.6, 133.3, 132.3, 131.1, 129.2, 114.9, 114.6, 114.3, 108.9, 93.4, 92.5, 55.5. Mp: 82–84 °C. HRMS (EI) *m*/*z* for C_15_H_10_I_2_O [M]^+^: calcd 459.8821; found, 459.8814.

### Synthesis of 1,2-diiodo-5-methyl-3-(phenylethynyl)benzene (9_A_)

The title compound was synthesized using the general procedure and isolated in **51%** yield as colorless oil after flash chromatography. IR (cast film, cm^−1^) 3086, 3015, 2209, 1604, 1573, 784, 652, 507. *δ*_H_ (400 MHz, CDCl_3_) *δ*: 7.67 (d, 1H, *J* = 1.1 Hz), 7.56–7.59 (m, 2H), 7.36–7.37 (m, 3H), 7.30 (d, 1H, *J* = 1.1 Hz), 2.24 (s, 3H). *δ*_C_ (100 MHz, CDCl_3_) *δ*: 139.9, 139.5, 132.4, 131.8, 131.3, 129.0, 128.6, 122.8, 110.5, 108.8, 93.5, 92.7, 20.4. HRMS (EI) *m*/*z* for C_15_H_10_I_2_ [M]^+^: calcd 443.8872; found, 443.8867.

### Synthesis of 1-((4-ethylphenyl)ethynyl)-2,3-diiodo-5-methyl benzene (10_A_)

The title compound was synthesized using the general procedure and isolated in **85%** yield as colorless oil after flash chromatography. IR (cast film, cm^−1^) 3104, 3086, 3042, 2198, 1594, 1546, 964, 862, 779, 634. *δ*_H_ (400 MHz, CDCl_3_) *δ*: 7.65 (s, 1H), 7.49 (d, 2H, *J* = 8.0 Hz), 7.29 (d, 1H, *J* = 1.0 Hz), 7.19 (d, 2H, *J* = 8.0 Hz), 2.64–2.70 (m, 2H), 2.23 (s, 3H), 1.23–1.27 (m, 3H). *δ*_C_ (100 MHz, CDCl_3_) *δ*: 145.5, 139.7, 139.5, 132.7, 132.3, 131.8, 131.5, 128.1, 120.0, 110.5, 108.7, 93.0, 29.1, 20.4, 15.5. HRMS (EI) *m*/*z* for C_17_H_14_I_2_ [M]^+^: calcd 471.9185; found, 471.9182.

### Synthesis of 1,2-diiodo-3-((4-methoxyphenyl)ethynyl)-5-methyl benzene (11_A_)

The title compound was synthesized using the general procedure and isolated in **42%** yield as white solid after flash chromatography. IR (cast film, cm^−1^) 3107, 3056, 2205, 1614, 1573, 1168, 1021, 983, 673. *δ*_H_ (400 MHz, CDCl_3_) *δ*: 7.64 (s, 1H), 7.51 (d, 2H, *J* = 8.7 Hz), 7.27 (s, 1H), 6.89 (d, 2H, *J* = 8.7 Hz), 3.83 (s, 3H), 2.23 (s, 3H). *δ*_C_ (100 MHz, CDCl_3_) *δ*: 160.2, 139.5, 139.4, 133.3, 132.1, 131.6, 114.9, 114.2, 110.4, 108.7, 92.9, 92.5, 55.5, 20.4. Mp: 91–93 °C HRMS (EI) *m*/*z* for C_16_H_12_I_2_O [M]^+^: calcd 473.8978; found, 473.8974.

### Synthesis of 1,2-diiodo-5-methoxy-3-((4-methoxyphenyl)ethynyl)benzene (12_A_)

The title compound was synthesized using the general procedure and isolated in **51%** yield as white solid after flash chromatography (5% EtOAc/hexane) using the general procedure. IR (cast film, cm^−1^) 3042, 3012, 2195, 1594, 1548, 1209, 1168, 1023, 918, 652. *δ*_H_ (400 MHz, CDCl_3_) *δ*: 7.52 (d, 2H, *J* = 8.6 Hz), 7.39 (d, 1H, *J* = 2.8 Hz), 7.02 (d, 1H, *J* = 2.7 Hz), 6.89 (d, 2H, *J* = 8.6 Hz), 3.83 (s, 3H), 3.78 (s, 3H). *δ*_C_ (100 MHz, CDCl_3_) *δ*: 160.3, 159.4, 133.3, 131.9, 125.6, 117.1, 114.7, 114.3, 108.8, 103.6, 93.1, 92.5, 55.8, 55.5. Mp: 83–85 °C. HRMS (EI) *m*/*z* for C_16_H_12_I_2_O_2_ [M]^+^: calcd exact 489.8927; found 489.8921.

### Synthesis of 1-((4-ethylphenyl)ethynyl)-2,3-diiodo-5-methoxy benzene (13_A_)

The title compound was synthesized using the general procedure and isolated in **29%** yield as white solid after flash chromatography. IR (cast film, cm^−1^) 3059, 3024, 2212, 1584, 1542, 1145, 1048, 956, 768. *δ*_H_ (400 MHz, CDCl_3_) *δ*: 7.50 (d, 2H, *J* = 8.0 Hz), 7.40 (d, 1H, *J* = 2.8), 7.20 (d, 2H, *J* = 7.8), 7.04 (d, 1H, *J* = 2.8), 3.78 (s, 3H), 2.6–2.7 (m, 2H), 1.24 (t, 3H, *J* = 7.6). *δ*_C_ (100 MHz, CDCl_3_) *δ*: 159.4, 145.7, 131.8, 128.2, 125.8, 119.8, 117.3, 108.8, 103.7, 93.2, 93.0, 55.8, 29.1, 15.5, (missing one peak due to overlapping). Mp: 96–98 °C. HRMS (EI) *m*/*z* for C_17_H_14_I_2_O [M]^+^: calcd 487.9134; found, 487.9121.

### Synthesis of 1,2-diiodo-5-methoxy-3-((4-(trifluoromethyl)phenyl)ethynyl)benzene (14_A_)

The title compound was synthesized using the general procedure and isolated in **36%** yield as white solid after flash chromatography. IR (cast film, cm^−1^) 3102, 3075, 3005, 2209, 1597, 1578, 1310, 1125, 993, 867, 631, 523. *δ*_H_ (400 MHz, CDCl_3_) *δ*: 7.68 (d, 2H, *J* = 8.2 Hz), 7.62 (d, 2H, *J* = 8.4 Hz), 7.44 (d, 1H, *J* = 2.9 Hz), 7.06 (d, 1H, *J* = 2.8 Hz), 3.8 (s, 3H). *δ*_C_ (100 MHz, CDCl_3_) *δ*: 159.5, 132.1, 130.9, 130.8 (q, *J*_C–F_ = 30 Hz), 126.5, 126.4, 125.5 (q, *J*_C–F_ = 4 Hz), 124.0 (q, *J*_C–F_ = 270 Hz), 117.7, 109.1, 103.7, 95.5, 91.1, 55.9. Mp: 92–94 °C. HRMS (EI) *m*/*z* for C_16_H_9_F_3_I_2_O [M]^+^: calcd 527.8695; found, 527.8682.

### Synthesis of 1-((4-fluorophenyl)ethynyl)-2,3-diiodo-5-methoxybenzene (15_A_)

The title compound was synthesized using the general procedure and isolated in **27%** yield as white solid after flash chromatography. IR (cast film, cm^−1^) 3104, 3078, 2204, 1608, 1599, 1359, 1146, 984, 873, 749, 653. *δ*_H_ (400 MHz, CDCl_3_) *δ*: 7.54–7.58 (m, 2H), 7.41 (d, 1H, *J* = 2.9 Hz), 7.07 (t, 2H, *J* = 8.7 Hz), 7.03 (d, 1H, *J* = 2.8 Hz), 3.78 (s, 3H). *δ*_C_ (100 MHz, CDCl_3_) *δ*: 163.0 (d, *J*_C–F_ = 149 Hz), 159.4, 133.8, 133.8 (d, *J*_C–F_ = 9 Hz), 125.9, 118.8 (d, *J*_C–F_ = 4 Hz), 117.4, 115.9 (d, *J*_C–F_ = 21 Hz), 108.9, 103.6, 93.1, 91.7, 55.8. Mp: 70–72 °C. HRMS (EI) *m*/*z* for C_15_H_9_FI_2_O [M]^+^: calcd 477.8727; found, 477.8713.

### Synthesis of 4-((2,3-diiodo-5-methoxyphenyl)ethynyl)-1,1′-biphenyl (16_A_)

The title compound was synthesized using the general procedure and isolated in **51%** yield white solid after flash chromatography. IR (cast film, cm^−1^) 3145, 3048, 2213, 1609, 1588, 1189, 1077, 961, 632. *δ*_H_ (400 MHz, CDCl_3_) *δ*: 7.55–7.70 (m, 5H), 7.47 (dd, 2H, *J*^1^ = 7.3 Hz, *J*^2^ = 7.8 Hz), 7.42 (d, 1H, *J* = 2.8 Hz), 7.37 (dd, 2H, *J*^1^ = 7.4 Hz, *J*^2^ = 7.2 Hz), 7.07 (d, 1H, *J* = 2.8),3.80 (s, 3H). *δ*_C_ (100 MHz, CDCl_3_) *δ*: 159.5, 141.8, 140.4, 132.3, 131.7, 129.1, 127.9, 127.3, 127.2, 125.9, 121.5, 117.4, 108.9, 103.8, 94.1, 92.8, 55.9. Mp: 85–87 °C. HRMS (EI) *m*/*z* for C_21_H_14_I_2_O [M]^+^: calcd 535.9134; found, 535.9119.

### Synthesis of 5-chloro-1,2-diiodo-3-(phenylethynyl)benzene (17_A_)

The title compound was synthesized using the general procedure and isolated in **42%** yield as white solid after flash chromatography. IR (cast film, cm^−1^) 3104, 3086, 2207, 1602, 1071, 943, 827, 634. *δ*_H_ (400 MHz, CDCl_3_) *δ*: 7.79 (d, 1H, *J* = 2.4 Hz), 7.57–7.59 (m, 2H), 7.46 (d, 1H, *J* = 2.3 Hz), 7.38–7.39 (m, 3H). *δ*_C_ (100 MHz, CDCl_3_) *δ*: 138.3, 134.6, 132.6, 131.9, 131.2, 129.4, 128.6, 122.3, 112.5, 109.2, 94.3, 92.5. Mp: 90–92 °C. HRMS (EI) *m*/*z* for C_14_H_7_ClI_2_ [M]^+^: calcd 463.8326; found, 463.8323.

### Synthesis of 5-chloro-1-((4-ethylphenyl)ethynyl)-2,3-diiodo benzene (18_A_)

The title compound was synthesized using the general procedure and isolated in **34%** yield as white solid after flash chromatography. IR (cast film, cm^−1^): 3104, 3089, 3018, 2212, 1579, 1542, 928, 813, 742, 642. *δ*_H_ (400 MHz, CDCl_3_) *δ*: 7.78 (d, 1H, *J* = 2.2 Hz), 7.49 (d, 2H, *J* = 8.0 Hz), 7.45 (d, 1H, *J* = 2.2 Hz), 7.21 (d, 2H, *J* = 7.9 Hz), 2.67 (q, 2H, *J* = 7.5 Hz), 1.25 (t, 3H, *J* = 7.6 Hz). *δ*_C_ (100 MHz, CDCl_3_) *δ*: 146.0, 138.0, 134.5, 132.8, 131.9, 131.0, 128.2, 119.4, 112.5, 109.2, 94.6, 92.0, 29.1, 15.5. Mp: 87–89 °C. HRMS (EI) *m*/*z* for C_16_H_11_ClI_2_ [M]^+^: calcd 491.8639; found, 491.8632.

### Synthesis of 5-chloro-1,2-diiodo-3-((4-methoxyphenyl)ethynyl)benzene (19_A_)

The title compound was synthesized using the general procedure and isolated in **44%** yield as white solid after flash chromatography. IR (cast film, cm^1^): 3121, 3049, 2209, 1613, 1597, 1310, 1149, 976, 842, 742, 619. *δ*_H_ (400 MHz, CDCl_3_) *δ*: 7.76 (d, 1H, *J* = 2.2 Hz), 7.51 (d, 2H, *J* = 8.7 Hz), 7.43 (d, 1H, *J* = 2.2 Hz), 6.89 (d, 2H, *J* = 8.8 Hz), 3.48 (s, 3H). *δ*_C_ (100 MHz, CDCl_3_) *δ*: 160.6, 137.9, 134.5, 133.6, 133.5, 132.9, 130.9, 114.3, 112.3, 109.1, 94.6, 91.6, 55.5. Mp: 112–114 °C. HRMS (EI) *m*/*z* for C_15_H_9_ClI_2_O [M]^+^: calcd 493.8431; found, 493.8425.

### Synthesis of 5-chloro-1,2-diiodo-3-((4-(trifluoromethyl)phenyl)ethynyl)benzene (20_A_)

The title compound was synthesized using the general procedure and isolated in **31%** yield as white solid after flash chromatography. IR (cast film, cm^−1^) 3107, 3088, 3012, 2196, 1612, 1579, 1012, 983, 867, 764, 642, 521. *δ*_H_ (400 MHz, CDCl_3_) *δ*: 7.81 (d, 1H, *J* = 2.4 Hz), 7.60–7.70 (m, 4H), 7.46 (d, 1H, *J* = 2.4 Hz). *δ*_C_ (100 MHz, CDCl_3_) *δ*: 138.8, 134.7, 133.0, 132.1, 131.8, 131.4, 131.0 (q, *J* = 32 Hz), 125.6 (q, *J* = 4 Hz), 123.9 (q, *J*_C–F_ = 271 Hz), 112.6, 109.5, 94.4, 92.4. Mp: 116–118 °C. HRMS (EI) *m*/*z* for C_15_H_6_ClF_3_I_2_ [M]^+^: calcd 531.8199; found, 531.8188.

### Synthesis of 5-bromo-1-((4-ethylphenyl)ethynyl)-2,3-diiodobenzene (21_A_)

The title compound was synthesized using the general procedure and isolated in **85%** yield as colorless oil after flash chromatography. IR (cast film, cm^1^): 3142, 3048, 3013, 2201, 1592, 943, 841, 746, 529. *δ*_H_ (400 MHz, CDCl_3_) *δ*: 7.93 (d, 1H, *J* = 2.2 Hz), 7.67 (d, 1H, *J* = 2.2 Hz), 7.47 (d, 2H, *J* = 8.1 Hz), 7.20 (d, 2H, *J* = 8.0 Hz), 2.68 (q, 2H, *J* = 7.6 Hz), 1.25 (t, 3H, *J* = 7.6 Hz). *δ*_C_ (100 MHz, CDCl_3_) *δ*: 146.0, 141.6, 135.4, 132.0, 128.2, 125.5, 120.1, 119.4, 99.3, 96.7, 85.0, 29.1, 15.5, (missing one peak due to overlapping). HRMS (EI) *m*/*z* for C_16_H_11_BrI_2_ [M]^+^: calcd 535.8133; found, 535.8128.

### Synthesis of 5-bromo-1-((3-fluorophenyl)ethynyl)-2,3-diiodo benzene (22_A_)

The title compound was synthesized using the general procedure and isolated in **44%** yield as white solid after flash chromatography. IR (cast film, cm^−1^) 3097, 3048, 2214, 1598, 948, 812, 794, 653. *δ*_H_ (400 MHz, CDCl_3_) *δ*: 7.95 (d, 1H, *J* = 2.2 Hz), 7.66 (d, 1H, *J* = 2.2 Hz), 7.32–7.34 (m, 2H), 7.23 (d, 1H, *J* = 9.2 Hz), 7.10–7.11 (m, 1H). *δ*_C_ (100 MHz, CDCl_3_) *δ*: 161.5 (d, *J*_C–F_ = 246 Hz), 142.2, 139.3, 135.6, 130.2 (d, *J*_C–F_ = 8 Hz), 129.9 (d, *J*_C–F_ = 3 Hz), 124.8, 124.0 (d, *J*_C–F_ = 10 Hz), 120.2, 118.8 (d, *J*_C–F_ = 22 Hz), 116.8 (d, *J*_C–F_ = 22 Hz), 99.4, 94.8 (d, *J*_C–F_ = 3 Hz), 86.2. Mp: 68–70 °C HRMS (EI) *m*/*z* for C_14_H_6_BrFI_2_ [M]^+^: calcd 525.7726; found, 525.7719.

### Synthesis of 5-fluoro-1,2-diiodo-3-((4-methoxyphenyl)ethynyl)benzene (23_A_)

The title compound was synthesized using the general procedure and isolated in **74%** yield as white solid after flash chromatography. IR (cast film, cm^−1^) 3102, 3042, 2191, 1608, 1597, 1023, 894, 742, 691. *δ*_H_ (400 MHz, CDCl_3_) *δ*: 7.51–7.57 (m, 3H), 7.20 (dd, 1H, *J*^1^ = 2.7 Hz, *J*^2^ = 8.6 Hz), 6.90 (d, 2H, *J* = 8.6 Hz), 3.84 (s, 3H). *δ*_C_ (100 MHz, CDCl_3_) *δ*: 161.6 (d, *J*_C–F_ = 251 Hz), 160.6, 133.5, 132.9 (d, *J*_C–F_ = 10 Hz), 130.9, 126.3 (d, *J*_C–F_ = 24 Hz), 118.4 (d, *J*_C–F_ = 23 Hz), 108.7 (d, *J*_C–F_ = 4 Hz), 108.6, 108.5, 94.5, 91.8 (d, *J*_C–F_ = 3 Hz), 55.5. Mp: 87–90 °C. HRMS (EI) *m*/*z* for C_15_H_9_FI_2_O [M]^+^: calcd 477.8727; found, 477.8718.

### Synthesis of 1-((4-chlorophenyl)ethynyl)-5-fluoro-2,3-diiodo benzene (24_A_)

The title compound was synthesized using the general procedure and isolated in **42%** yield as white solid after flash chromatography. IR (cast film, cm^−1^) 3089, 3041, 2201, 1597, 1524, 1016, 927, 813, 691. *δ*_H_ (400 MHz, CDCl_3_) *δ*: 7.57 (dd, 1H, *J*^1^ = 7.7 Hz, *J*^2^ = 2.4 Hz), 7.48 (d, 2H, *J* = 8.3 Hz), 7.34 (d, 2H, *J* = 8.2 Hz), 7.20 (dd, 1H, *J*^1^ = 8.6 Hz, *J*^2^ = 2.4 Hz). *δ*_C_ (100 MHz, CDCl_3_) *δ*: 161.5 (d, *J*_C–F_ = 252 Hz), 135.5, 133.1, 132.1 (d, *J*_C–F_ = 10 Hz), 129.0, 126.8 (d, *J*_C–F_ = 24 Hz), 120.8, 118.8 (d, *J*_C–F_ = 23 Hz), 108.9 (d, *J*_C–F_ = 4 Hz), 108.8 (d, *J*_C–F_ = 8 Hz), 93.5 (d, *J*_C–F_ = 3 Hz), 92.9. Mp: 113–114 °C. HRMS (EI) *m*/*z* for C_14_H_6_ClFI_2_ [M]^+^: calcd 481.8231; found, 481.8228.

### Synthesis of methyl 3,4-diiodo-5-((4-methoxyphenyl)ethynyl)benzoate (25_A_)

The title compound was synthesized using the general procedure and isolated in **33%** yield as white solid after flash chromatography. IR (cast film, cm^−1^) 3104, 3085, 2195, 1765, 1604, 1586, 1415, 1204, 1112, 876, 743. *δ*_H_ (400 MHz, CDCl_3_) *δ*: 8.37 (d, 1H, *J* = 1.8 Hz), 8.05 (d, 1H, *J* = 1.8 Hz), 7.52 (d, 2H, *J* = 8.8 Hz), 6.90 (d, 2H, *J* = 8.7 Hz), 3.92 (s, 3H), 3.84 (s, 3H). *δ*_C_ (100 MHz, CDCl_3_) *δ*: 165.1, 160.5, 138.7, 133.4, 132.5, 131.5, 131.1, 120.6, 114.5, 114.3, 108.9, 94.4, 91.9, 55.5, 52.8. Mp: 173–175 °C. HRMS (EI) *m*/*z* for C_17_H_12_I_2_O_3_ [M]^+^: calcd 517.8876; found, 517.8874.

### Synthesis of 1-chloro-3-((4-chlorophenyl)ethynyl)-2-iodobenzene (26_A_)

The title compound was synthesized using the general procedure and isolated in **45%** yield as white solid after flash chromatography. IR (cast film, cm^−1^) 3078, 3024, 2208, 1601, 1598, 1107, 867, 523. *δ*_H_ (400 MHz, CDCl_3_) *δ*: 7.52 (d, 2H, *J* = 8.4 Hz), 7.34–7.40 (m, 4H), 7.26 (dd, 1H, *J* = 7.7 Hz, *J* = 7.9 Hz). *δ*_C_ (100 MHz, CDCl_3_) *δ*: 139.8, 135.2, 133.9, 133.0, 132.5, 130.4, 129.0, 128.9, 121.3, 105.5, 93.0, 92.5. Mp: 90–92 °C. HRMS (EI) *m*/*z* for C_14_H_7_Cl_2_I [M]^+^: calcd 371.8969; found, 371.8960.

### Synthesis of 1-bromo-2-iodo-3-((4-methoxyphenyl)ethynyl) benzene (27_A_)

The title compound was synthesized using the general procedure and isolated in **62%** yield as white solid after flash chromatography. IR (cast film, cm^−1^) 3097, 3021, 2207, 1578, 1543, 1204, 1079, 894, 742. *δ*_H_ (400 MHz, CDCl_3_) *δ*: 7.53 (d, 3H, *J* = 8.2 Hz), 7.41 (d, 1H, *J* = 7.6 Hz), 7.17 (dd, 1H, *J*^1^ = 7.9, *J*^2^ = 7.8 Hz), 6.90 (d, 2H, *J* = 8.3 Hz), 3.84 (s, 3H). *δ*_C_ (100 MHz, CDCl_3_) *δ*: 160.3, 133.3, 133.2, 131.8, 130.7, 130.5, 129.1, 114.8, 114.3, 108.3, 93.9, 91.6, 55.5. Mp: 53–55 °C. HRMS (EI) *m*/*z* for C_15_H_10_BrIO [M]^+^: calcd 411.8960; found, 411.8954.

### General procedure for one-pot double Sonogashira cross-coupling reactions of 5-substituted-1,2,3-triiodobenzenes

A flame-dried Schlenk flask was charged with 5-substituted-1,2,3-triiodobenzene (0.65 mmol, 0.08 M, 1.0 equiv.), aryl acetylene (1.0 equiv.), Cs_2_CO_3_ (7.0 equiv.) in 8.0 mL dry toluene under argon. The mixture was stirred at room temperature for 20 min. Tetrakis(triphenylphosphine)palladium (0) (10 mol%) and copper iodide (20 mol%) were added, capped with septum, carefully degassed with argon, and the reaction flask was wrapped with aluminum foil and stirred at room temperature for 24 h. Aryl acetylene (1.0 equiv.) and Cs_2_CO_3_ (7.0 equiv.) were added to the mixture, carefully degassed with argon, wrapped with aluminum foil and stirred at room temperature for another 24 h. The reaction mixture was diluted with EtOAc and filtered over Celite 545®. Distilled water (100 mL) was added and extracted with EtOAc (2 × 50 mL). The organic layers were combined washed with brine, dried with anhyd. Na_2_SO_4_, filtered and evaporated under reduced pressure. The crude product was purified by flash chromatography (100% hexane) to yield the pure desired product.

### Synthesis of 2-iodo-5-methoxy-1-((4-methoxyphenyl)ethynyl)-3-((4-(trifluoromethyl)phenyl)ethynyl)benzene (28_A_)

The title compound was synthesized using the one-pot general procedure and isolated in **48%** yield as pale yellow oil after flash chromatography. IR (cast film, cm^−1^) 3124, 3086, 3006, 2203, 1601, 1583, 1204, 1139, 976, 867, 647. *δ*_H_ (400 MHz, CDCl_3_) *δ*: 7.71 (d, 2H, *J* = 8.1 Hz), 7.62 (d, 2H, *J* = 8.1 Hz), 7.56 (d, 2H, *J* = 8.5 Hz), 7.06 (d, 1H, *J* = 2.7 Hz), 7.04 (d, 1H, *J* = 2.8 Hz), 6.90 (d, 2H, *J* = 8.5 Hz), 3.84 (s, 3H), 3.82 (s, 3H). *δ*_C_ (100 MHz, CDCl_3_) *δ*: 160.3, 159.2, 133.4, 131.5 (q, *J*_C–F_ = 134 Hz), 132.0, 130.5 (q, *J*_C–F_ = 33 Hz), 126.8, 125.5, 125.4 (q, *J*_C–F_ = 4 Hz), 122.7, 118.2, 118.0, 114.9, 114.3, 97.0, 94.3, 93.7, 91.3, 90.8, 55.8, 55.5. HRMS (EI) *m*/*z* for C_25_H_16_F_3_IO_2_ [M]^+^: calcd 532.0147; found, 532.0133.

### Synthesis of 5-bromo-1-((4-ethylphenyl)ethynyl)-3-((3-fluoro phenyl)ethynyl)-2-iodobenzene (29_A_)

The title compound was synthesized using the one-pot general procedure and isolated in **34%** yield as white solid after flash chromatography. IR (cast film, cm^−1^) 3125, 3073, 3041, 2201, 1623, 1579, 1042, 941, 837, 622. *δ*_H_ (400 MHz, CDCl_3_) *δ*: 7.64 (dd, 2H, *J*^1^ = 10.9 Hz, *J*^2^ = 2.1 Hz), 7.50 (d, 2H, *J* = 7.9 Hz), 7.28–7.36 (m, 3H), 7.21 (d, 2H, *J* = 7.8 Hz), 7.00–7.15 (m, 1H), 2.67 (q, 2H, *J* = 7.6 Hz), 1.26 (m, 3H). *δ*_C_ (100 MHz, CDCl_3_) *δ*: 162.5 (d, *J*_C–F_ = 245 Hz), 136.6, 135.4, 135.0, 132.0, 130.2 (d, *J*_C–F_ = 8 Hz), 128.2, 127.9 (d, *J*_C–F_ = 2 Hz), 126.0, 125.1, 124.3 (d, *J*_C–F_ = 9 Hz), 119.5, 119.4, 118.7 (d, *J*_C–F_ = 23 Hz), 116.6 (d, *J*_C–F_ = 21 Hz), 96.8, 94.6, 85.8, 84.3, 29.1, 15.5, (missing one peak due to overlapping). Mp: 83–85 °C. HRMS (EI) *m*/*z* for C_24_H_15_BrFI [M]^+^: calcd 527.9386; found, 527.9382.

### Synthesis of 4,4′-((2-iodo-1,3-phenylene)bis(ethyne-2,1-diyl))bis(methoxybenzene) (30_A_)

The title compound was synthesized using the one-pot general procedure and isolated in **57%** yield as pale yellow oil after flash chromatography. IR (cast film, cm^−1^) 3124, 3082, 3012, 2214, 1624, 1602, 967, 962, 841, 723. *δ*_H_ (400 MHz, CDCl_3_) *δ*: 7.55 (d, 4H, *J* = 8.7 Hz), 7.40 (d, 2H, *J* = 7.5 Hz), 7.28 (d, 1H, *J* = 8.0 Hz), 6.90 (d, 4H, *J* = 8.7 Hz), 3.8 (s, 6H). *δ*_C_ (100 MHz, CDCl_3_) *δ*: 160.2, 133.3, 131.5, 131.2, 127.8, 115.2, 114.3, 107.7, 93.5, 91.2, 55.5. HRMS (EI) *m*/*z* for C_24_H_17_IO_2_ [M]^+^: calcd 464.0273; found, 464.0269.

### Synthesis of 4,4′-((2-iodo-5-methoxy-1,3-phenylene)bis(ethyne-2,1-diyl))bis(methoxybenzene) (31_A_)

The title compound was synthesized using the one-pot general procedure and isolated in **69%** yield as colorless oil after flash chromatography. IR (cast film, cm^−1^) 3124, 3075, 3042, 2198, 1614, 1587, 1345, 1207, 1184, 986, 748, 625. *δ*_H_ (400 MHz, CDCl_3_) *δ*: 7.55 (d, 4H, *J* = 8.6 Hz), 7.02 (s, 2H), 6.90 (d, 4H, *J* = 8.6 Hz), 3.84 (s, 6H), 3.82 (s, 3H). *δ*_C_ (100 MHz, CDCl_3_) *δ*: 160.2, 159.2, 133.4, 131.9, 117.5, 115.1, 114.3, 96.9, 93.3, 91.1, 55.8, 55.5. HRMS (EI) *m*/*z* for C_25_H_19_IO_3_ [M]^+^: calcd 494.0379; found, 494.0371.

### Synthesis of 3,3′-((5-bromo-2-iodo-1,3-phenylene)bis(ethyne-2,1-diyl))bis(fluorobenzene) (32_A_)

The title compound was synthesized using the one-pot general procedure and isolated in **55%** yield as white solid after flash chromatography. IR (cast film, cm^−1^) 3124, 3057, 2195, 1604, 1578, 684, 742, 628. *δ*_H_ (400 MHz, CDCl_3_) *δ*: 7.66 (s, 2H), 7.33–7.36 (m, 4H), 7.22–7.30 (m, 2H), 7.07–7.12 (m, 2H). *δ*_C_ (100 MHz, CDCl_3_) *δ*: 162.6 (d, *J*_C–F_ = 245 Hz), 136.8, 135.5, 130.3 (d, *J*_C–F_ = 8 Hz), 127.9 (d, *J*_C–F_ = 2 Hz), 125.4, 124.2 (d, *J*_C–F_ = 9 Hz), 119.5, 118.8 (d, *J*_C–F_ = 22 Hz), 116.7 (d, *J*_C–F_ = 21 Hz), 94.9 (d, *J*_C–F_ = 3 Hz), 85.6. Mp: 122–124 °C. HRMS (EI) *m*/*z* for C_22_H_10_BrF_2_I [M]^+^: calcd 517.8979; found, 517.8975.

### Synthesis of 4,4′-((5-bromo-2-iodo-1,3-phenylene)bis(ethyne-2,1-diyl))bis(ethylbenzene) (33_A_)

The title compound was synthesized using the one-pot general procedure and isolated in **87%** yield as colorless oil after flash chromatography. IR (cast film, cm^−1^) 3106, 3086, 3015, 2186, 1612, 1592, 837, 467. *δ*_H_ (400 MHz, CDCl_3_) *δ*: 7.61 (s, 2H), 7.44 (d, 4H, *J* = 7.8 Hz), 7.16–7.21 (m, 4H), 2.60–2.75 (m, 4H), 1.20–1.30 (m, 6H). *δ*_C_ (100 MHz, CDCl_3_) *δ*: 145.5, 133.8, 132.7, 131.9, 128.2, 125.7, 119.9, 91.6, 86.7, 81.7, 29.0, 15.4. HRMS (EI) *m*/*z* for C_26_H_20_BrI [M]^+^: calcd 537.9793; found, 537.9784.

### Synthesis of 4,4′-((2-iodo-1,3-phenylene)bis(ethyne-2,1-diyl))bis ((trifluoromethyl)benzene) (34_A_)

The title compound was synthesized using the one-pot general procedure and isolated in 72% yield as white solid after flash chromatography. IR (cast film, cm^−1^) 3108, 3086, 3046, 2216, 1587, 1537, 976, 891, 642. *δ*_H_ (400 MHz, CDCl_3_) *δ*: 7.72 (d, 4H, *J* = 8.1 Hz), 7.64 (d, 4H, *J* = 8.2 Hz), 7.50 (d, 2H, *J* = 7.6 Hz), 7.36 (dd, 1H, *J*^1^ = 7.9, *J*^2^ = 7.5 Hz). *δ*_C_ (100 MHz, CDCl_3_) *δ*: 132.5, 132.1, 130.9, 130.8, 128.1, 126.7, 125.6 (q, *J*_C–F_ = 4 Hz), 122.7, 108.1, 94.1, 91.9. Mp: 92–94 °C. HRMS (EI) *m*/*z* for C_24_H_11_F_6_I [M]^+^: calcd 539.9810; found, 539.9798.

### Synthesis of 4,4′-((5-chloro-2-iodo-1,3-phenylene)bis(ethyne-2,1-diyl))bis((trifluoromethyl)benzene) (35_A_)

The title compound was synthesized using the one-pot general procedure and isolated in **55%** yield as white solid after flash chromatography. IR (cast film, cm^−1^) 3097, 3064, 2207, 1599, 1548, 948, 816, 724, 634. *δ*_H_ (400 MHz, CDCl_3_) *δ*: 7.70 (d, 4H, *J* = 8.3 Hz), 7.64 (d, 4H, *J* = 8.9 Hz), 7.47 (s, 2H). *δ*_C_ (100 MHz, CDCl_3_) *δ*: 138.8, 134.3, 133.0, 132.2 (q, *J*_C–F_ = 34 Hz), 132.1 (q, *J*_C–F_ = 3 Hz), 131.0 (q, *J*_C–F_ = 33 Hz), 125.6 (q, *J*_C–F_ = 3 Hz), 124.0 (q, *J*_C–F_ = 171 Hz), 105.5, 93.0, 92.9. Mp: 116–118 °C. HRMS (EI) *m*/*z* for C_24_H_10_ClF_6_I [M]^+^: calcd 573.9420; found, 573.9412.

## Conflicts of interest

There are no conflicts to declare.

## Supplementary Material

RA-010-D0RA01569E-s001

RA-010-D0RA01569E-s002
