# Supplementary material for: Palladium-catalyzed highly regioselective mono and double Sonogashira cross-coupling reactions of 5-substituted-1,2,3-triiodobenzene under ambient conditions
Source: RSC Adv. 2020 Apr 24;10(28):16366–76. doi: 10.1039/d0ra01569e (PMC9053030; doi:10.1039/d0ra01569e)
Supplement: RA-010-D0RA01569E-s001 [file RA-010-D0RA01569E-s001.pdf]

Electronic Supplementary Material (ESI) for RSC Advances.
This journal is © The Royal Society of Chemistry 2020

Jordan University of Science and Technology – Department of Chemistry

PALLADIUM-CATALYZED HIGHLY REGIOSELECTIVE MONO AND DOUBLE SONOGASHIRA COUPLING REACTIONS OF 5-SUBSTITUTED-1,2,3-TRIIODOBENZENE UNDER AMBIENT CONDITIONS

Supporting Information

Table of Contents

Table of Contents ii

1. **Experimental Details and Compound Data** 1

1.1 **General Information** 1

1.2 **General procedure for Sonogashira cross-coupling reactions of 5-substituted-1,2,3-triiodoarenes** 1

1.2.1 *Synthesis of 1,2-diiodo-3-(phenylethynyl)benzene (**5**$_A$)* 2
1.2.2 *Synthesis of 1,2-diiodo-3-((4-(trifluoromethyl)phenyl) ethynyl) benzene (**6**$_A$)* 2
1.2.3 *Synthesis of 1-((4-chlorophenyl)ethynyl)-2,3-diiodobenzene (**7**$_A$)* 3
1.2.4 *Synthesis of 1,2-diiodo-3-((4-methoxyphenyl)ethynyl)benzene (**8**$_A$)* 3
1.2.5 *Synthesis of 1,2-diiodo-5-methyl-3-(phenylethynyl)benzene (**9**$_A$)* 4
1.2.6 *Synthesis of 1-((4-ethylphenyl)ethynyl)-2,3-diiodo-5-methyl benzene (**10**$_A$)* 4
1.2.7 *Synthesis of 1,2-diiodo-3-((4-methoxyphenyl)ethynyl)-5-methyl benzene (**11**$_A$)* 5
1.2.8 *Synthesis of 1,2-diiodo-5-methoxy-3-((4-methoxyphenyl) ethynyl) benzene (**12**$_A$)* 5
1.2.9 *Synthesis of 1-((4-ethylphenyl)ethynyl)-2,3-diiodo-5-methoxy benzene (**13**$_A$)* 6
1.2.10 *Synthesis of 1,2-diiodo-5-methoxy-3-((4-(trifluoromethyl) phenyl) ethynyl)benzene (**14**$_A$)* 6
1.2.11 *Synthesis of 1-((4-fluorophenyl)ethynyl)-2,3-diiodo-5-methoxy benzene (**15**$_A$)* 7
1.2.12 *Synthesis of 4-((2,3-diiodo-5-methoxyphenyl)ethynyl)-1,1'-biphenyl (**16**$_A$)* 7
1.2.13 *Synthesis of 5-chloro-1,2-diiodo-3-(phenylethynyl)benzene (**17**$_A$)* 8
1.2.14 *Synthesis of 5-chloro-1-((4-ethylphenyl)ethynyl)-2,3-diiodobenzene (**18**$_A$)* 8
1.2.15 *Synthesis of 5-chloro-1,2-diiodo-3-((4-methoxyphenyl) ethynyl) benzene (**19**$_A$)* 9
1.2.16 *Synthesis of 5-chloro-1,2-diiodo-3-((4-(trifluoromethyl)phenyl) ethynyl) benzene (**20**$_A$)* 9
1.2.17 *Synthesis of 5-bromo-1-((4-ethylphenyl)ethynyl)-2,3-diiodobenzene (**21**$_A$)* 10
1.2.18 *Synthesis of 5-bromo-1-((3-fluorophenyl)ethynyl)-2,3-diiodo benzene (**22**$_A$)* 10
1.2.19 *Synthesis of 5-fluoro-1,2-diiodo-3-((4-methoxyphenyl)ethynyl) benzene (**23**$_A$)* 11
1.2.20 *Synthesis of 1-((4-chlorophenyl)ethynyl)-5-fluoro-2,3-diiodo benzene (**24**$_A$)* 11
1.2.21 *Synthesis of methyl 3,4-diiodo-5-((4-methoxyphenyl)ethynyl) benzoate (**25**$_A$)* 12
1.2.22 *Synthesis of 1-chloro-3-((4-chlorophenyl)ethynyl)-2-iodobenzene (**26**$_A$)* 12
1.2.23 *Synthesis of 1-bromo-2-iodo-3-((4-methoxyphenyl)ethynyl)benzene (**27**$_A$)* 13

1.3 **General procedure for one-pot double Sonogashira cross-coupling reactions of 5-substituted-1,2,3-triiodoarenes** 13

1.3.1 *Synthesis of 2-iodo-5-methoxy-1-((4-methoxyphenyl)ethynyl)-3-((4-(trifluoromethyl)phenyl) ethynyl)benzene (**28**$_A$)* 14
1.3.2 *Synthesis of 5-bromo-1-((4-ethylphenyl)ethynyl)-3-((3-fluorophenyl) ethynyl)-2-iodobenzene (**29**$_A$)* 14
1.3.3 *Synthesis of 4,4'-((2-iodo-1,3-phenylene)bis(ethyne-2,1-diyl))bis (methoxybenzene) (**30**$_A$)* 15
1.3.4 *Synthesis of 4,4'-((2-iodo-5-methoxy-1,3-phenylene)bis(ethyne-2,1-diyl))bis(methoxybenzene) (**31**$_A$)* 15
1.3.5 *Synthesis of 3,3'-((5-bromo-2-iodo-1,3-phenylene)bis(ethyne-2,1-diyl))bis(fluorobenzene) (**32**$_A$)* 16
1.3.6 *Synthesis of 4,4'-((5-bromo-2-iodo-1,3-phenylene)bis(ethyne-2,1-diyl)) bis(ethylbenzene) (**33**$_A$)* 16
1.3.7 *Synthesis of 4,4'-((2-iodo-1,3-phenylene)bis(ethyne-2,1-diyl))bis((trifluoromethyl)benzene) (**34**$_A$)* 17
1.3.8 *Synthesis of 4,4'-((5-chloro-2-iodo-1,3-phenylene)bis(ethyne-2,1-diyl))bis((trifluoromethyl) benzene) (**35**$_A$)* 17

1.4 **General procedure for one-pot double Suzuki-Miyaura cross-coupling reactions from 1,2-diiodo-3-(phenylethynyl)benzene** (5$_A$) 18

1.4.1 *Synthesis of 4,4''-dimethoxy-3'-(phenylethynyl)-1,1':2',1''-terphenyl (**36**)* 18
1.4.2 *Synthesis of 3'-(phenylethynyl)-4,4''-bis(trifluoromethyl)-1,1':2',1''-terphenyl (**37**)* 19

1.5 **NMR for New Compounds** 20

1.5.1 *^{1}H-NMR of 1,2-diiodo-3-(phenylethynyl)benzene (**5**$_A$) in $CDCl_3$ at 25 ^{o}C.* 20
1.5.2 *^{13}C-NMR of 1,2-diiodo-3-(phenylethynyl)benzene (**5**$_A$) in $CDCl_3$ at 25 ^{o}C.* 21
1.5.3 *^{1}H-NMR of 1,2-diiodo-3-((4-(trifluoromethyl)phenyl)ethynyl) benzene (**6**$_A$) in $CDCl_3$ at 25 ^{o}C.* 22
1.5.4 *^{13}C-NMR of 1,2-diiodo-3-((4-(trifluoromethyl)phenyl)ethynyl) benzene (**6**$_A$) in $CDCl_3$ at 25 ^{o}C.* 23

1.5.5 *^{1}H-NMR of 1-((4-chlorophenyl)ethynyl)-2,3-diiodobenzene (**7**$_{A}$) in $CDCl_3$ at 25 ^{o}C.* ... 24
1.5.6 *^{13}C-NMR of 1-((4-chlorophenyl)ethynyl)-2,3-diiodobenzene (**7**$_{A}$) in $CDCl_3$ at 25 ^{o}C.* ... 25
1.5.7 *^{1}H-NMR of 1,2-diiodo-3-((4-methoxyphenyl)ethynyl)benzene (**8**$_{A}$) in $CDCl_3$ at 25 ^{o}C.* ... 26
1.5.8 *^{13}C-NMR of 1,2-diiodo-3-((4-methoxyphenyl)ethynyl)benzene (**8**$_{A}$) in $CDCl_3$ at 25 ^{o}C.* ... 27
1.5.9 *^{1}H-NMR of 1,2-diiodo-5-methyl-3-(phenylethynyl)benzene (**9**$_{A}$) in $CDCl_3$ at 25 ^{o}C.* ... 28
1.5.10 *^{13}C-NMR of 1,2-diiodo-5-methyl-3-(phenylethynyl)benzene (**9**$_{A}$) in $CDCl_3$ at 25 ^{o}C.* ... 29
1.5.11 *^{1}H-NMR of 1-((4-ethylphenyl)ethynyl)-2,3-diiodo-5-methylbenzene (**10**$_{A}$) in $CDCl_3$ at 25 ^{o}C* ... 30
1.5.12 *^{13}C-NMR of 1-((4-ethylphenyl)ethynyl)-2,3-diiodo-5-methylbenzene (**10**$_{A}$) in $CDCl_3$ at 25 ^{o}C* ... 31
1.5.13 *^{1}H-NMR of 1,2-diiodo-3-((4-methoxyphenyl)ethynyl)-5-methyl benzene (**11**$_{A}$) in $CDCl_3$ at 25 ^{o}C.* ... 32
1.5.14 *^{13}C-NMR of 1,2-diiodo-3-((4-methoxyphenyl)ethynyl)-5-methyl benzene (**11**$_{A}$) in $CDCl_3$ at 25 ^{o}C.* ... 33
1.5.15 *^{1}H-NMR of 1,2-diiodo-5-methoxy-3-((4-methoxyphenyl)ethynyl)benzene (**12**$_{A}$) in $CDCl_3$ at 25 ^{o}C* ... 34
1.5.16 *^{13}C-NMR of 1,2-diiodo-5-methoxy-3-((4-methoxyphenyl)ethynyl) benzene (**12**$_{A}$) in $CDCl_3$ at 25 ^{o}C.* ... 35
1.5.17 *^{1}H-NMR of 1-((4-ethylphenyl)ethynyl)-2,3-diiodo-5-methoxy benzene (**13**$_{A}$) in $CDCl_3$ at 25 ^{o}C.* ... 36
1.5.18 *^{13}C-NMR of 1-((4-ethylphenyl)ethynyl)-2,3-diiodo-5-methoxy benzene (**13**$_{A}$) in $CDCl_3$ at 25 ^{o}C.* ... 37
1.5.19 *^{1}H-NMR of 1,2-diiodo-5-methoxy-3-((4-(trifluoromethyl) phenyl) ethynyl)benzene (**14**$_{A}$) in $CDCl_3$ at 25 ^{o}C.* ... 38
1.5.20 *^{13}C-NMR of 1,2-diiodo-5-methoxy-3-((4-(trifluoromethyl) phenyl) ethynyl)benzene (**14**$_{A}$) in $CDCl_3$ at 25 ^{o}C.* ... 39
1.5.21 *^{1}H-NMR of 1-((4-fluorophenyl)ethynyl)-2,3-diiodo-5-methoxybenzene (**15**$_{A}$) in $CDCl_3$ at 25 ^{o}C.* ... 40
1.5.22 *^{13}C-NMR of 1-((4-fluorophenyl)ethynyl)-2,3-diiodo-5-methoxybenzene (**15**$_{A}$) in $CDCl_3$ at 25 ^{o}C.* ... 41
1.5.23 *^{1}H-NMR of 4-((2,3-diiodo-5-methoxyphenyl)ethynyl)-1,1'-biphenyl (**16**$_{A}$) in $CDCl_3$ at 25 ^{o}C.* ... 42
1.5.24 *^{13}C-NMR of 4-((2,3-diiodo-5-methoxyphenyl)ethynyl)-1,1'-biphenyl (**16**$_{A}$) in $CDCl_3$ at 25 ^{o}C.* ... 43
1.5.25 *^{1}H-NMR of 5-chloro-1,2-diiodo-3-(phenylethynyl)benzene (**17**$_{A}$) in $CDCl_3$ at 25 ^{o}C.* ... 44
1.5.26 *^{13}C-NMR of 5-chloro-1,2-diiodo-3-(phenylethynyl)benzene (**17**$_{A}$) in $CDCl_3$ at 25 ^{o}C.* ... 45
1.5.27 *^{1}H-NMR of 5-chloro-1-((4-ethylphenyl)ethynyl)-2,3-diiodobenzene (**18**$_{A}$) in $CDCl_3$ at 25 ^{o}C.* ... 46
1.5.28 *^{13}C-NMR of 5-chloro-1-((4-ethylphenyl)ethynyl)-2,3-diiodobenzene (**18**$_{A}$) in $CDCl_3$ at 25 ^{o}C.* ... 47
1.5.29 *^{1}H-NMR of 5-chloro-1,2-diiodo-3-((4-methoxyphenyl)ethynyl) benzene (**19**$_{A}$) in $CDCl_3$ at 25 ^{o}C.* ... 48
1.5.30 *^{13}C-NMR of 5-chloro-1,2-diiodo-3-((4-methoxyphenyl)ethynyl) benzene (**19**$_{A}$) in $CDCl_3$ at 25 ^{o}C.* ... 49
1.5.31 *^{1}H-NMR of 5-chloro-1,2-diiodo-3-((4-(trifluoromethyl)phenyl)ethynyl)benzene (**20**$_{A}$) in $CDCl_3$ at 25 ^{o}C.* ... 50
1.5.32 *^{13}C-NMR of 5-chloro-1,2-diiodo-3-((4-(trifluoromethyl)phenyl) ethynyl)benzene (**20**$_{A}$) in $CDCl_3$ at 25 ^{o}C.* ... 51
1.5.33 *^{1}H-NMR of 5-bromo-1-((4-ethylphenyl)ethynyl)-2,3-diiodobenzene (**21**$_{A}$) in $CDCl_3$ at 25 ^{o}C.* ... 52
1.5.34 *^{13}C-NMR of 5-bromo-1-((4-ethylphenyl)ethynyl)-2,3-diiodobenzene (**21**$_{A}$) in $CDCl_3$ at 25 ^{o}C.* ... 53
1.5.35 *^{1}H-NMR of 5-bromo-1-((3-fluorophenyl)ethynyl)-2,3-diiodo benzene (**22**$_{A}$) in $CDCl_3$ at 25 ^{o}C.* ... 54
1.5.36 *^{13}C-NMR of 5-bromo-1-((3-fluorophenyl)ethynyl)-2,3-diiodobenzene (**22**$_{A}$) in $CDCl_3$ at 25 ^{o}C.* ... 55
1.5.37 *^{1}H-NMR of 5-fluoro-1,2-diiodo-3-((4-methoxyphenyl)ethynyl) benzene (**23**$_{A}$) in $CDCl_3$ at 25 ^{o}C.* ... 56
1.5.38 *^{13}C-NMR of 5-fluoro-1,2-diiodo-3-((4-methoxyphenyl)ethynyl) benzene (**23**$_{A}$) in $CDCl_3$ at 25 ^{o}C.* ... 57
1.5.39 *^{1}H-NMR of 1-((4-chlorophenyl)ethynyl)-5-fluoro-2,3-diiodobenzene (**24**$_{A}$) in $CDCl_3$ at 25 ^{o}C.* ... 58
1.5.40 *^{13}C-NMR of 1-((4-chlorophenyl)ethynyl)-5-fluoro-2,3-diiodobenzene (**24**$_{A}$) in $CDCl_3$ at 25 ^{o}C* ... 59
1.5.41 *^{1}H-NMR of methyl 3,4-diiodo-5-((4-methoxyphenyl)ethynyl)benzoate (**25**$_{A}$) in $CDCl_3$ at 25 ^{o}C.* ... 60
1.5.42 *^{13}C-NMR of methyl 3,4-diiodo-5-((4-methoxyphenyl)ethynyl)benzoate (**25**$_{A}$) in $CDCl_3$ at 25 ^{o}C.* ... 61
1.5.43 *^{1}H-NMR of 1-chloro-3-((4-chlorophenyl)ethynyl)-2-iodobenzene (**26**$_{A}$) in $CDCl_3$ at 25 ^{o}C.* ... 62
1.5.44 *^{13}C-NMR of 1-chloro-3-((4-chlorophenyl)ethynyl)-2-iodobenzene (**26**$_{A}$) in $CDCl_3$ at 25 ^{o}C.* ... 63
1.5.45 *^{1}H-NMR of 1-bromo-2-iodo-3-((4-methoxyphenyl)ethynyl)benzene (**27**$_{A}$) in $CDCl_3$ at 25 ^{o}C.* ... 64
1.5.46 *^{13}C-NMR of 1-bromo-2-iodo-3-((4-methoxyphenyl)ethynyl)benzene (**27**$_{A}$) in $CDCl_3$ at 25 ^{o}C.* ... 65
1.5.47 *^{1}H-NMR of 2-iodo-5-methoxy-1-((4-methoxyphenyl)ethynyl)-3-((4-(trifluoromethyl)phenyl) ethynyl)benzene (**28**$_{A}$) in $CDCl_3$ at 25 ^{o}C.* ... 66
1.5.48 *^{13}C-NMR of 2-iodo-5-methoxy-1-((4-methoxyphenyl)ethynyl)-3-((4-(trifluoromethyl)phenyl) ethynyl)benzene (**28**$_{A}$) in $CDCl_3$ at 25 ^{o}C.* ... 67
1.5.49 *^{1}H-NMR of 5-bromo-1-((4-ethylphenyl)ethynyl)-3-((3-fluorophenyl) ethynyl)-2-iodobenzene (**29**$_{A}$) in $CDCl_3$ at 25 ^{o}C.* ... 68
1.5.50 *^{13}C-NMR of 5-bromo-1-((4-ethylphenyl)ethynyl)-3-((3-fluorophenyl) ethynyl)-2-iodobenzene (**29**$_{A}$) in $CDCl_3$ at 25 ^{o}C.* ... 69
1.5.51 *^{1}H-NMR of 4,4'-((2-iodo-1,3-phenylene)bis(ethyne-2,1-diyl))bis (methoxybenzene) (**30**$_{A}$) in $CDCl_3$ at 25 ^{o}C.* ... 70
1.5.52 *^{13}C-NMR of 4,4'-((2-iodo-1,3-phenylene)bis(ethyne-2,1-diyl))bis (methoxybenzene) (**30**$_{A}$) in $CDCl_3$ at 25 ^{o}C.* ... 71

1.5.53 *^{1}H-NMR of 4,4'-((2-iodo-5-methoxy-1,3-phenylene)bis(ethyne-2,1-diyl))bis(methoxybenzene) (**31$_{A}$**) in $CDCl_3$ at 25 ^{o}C.* 72

1.5.54 *^{13}C-NMR of 4,4'-((2-iodo-5-methoxy-1,3-phenylene)bis(ethyne-2,1-diyl))bis(methoxybenzene) (**31$_{A}$**) in $CDCl_3$ at 25 ^{o}C.* 73

1.5.55 *^{1}H-NMR of 3,3'-((5-bromo-2-iodo-1,3-phenylene)bis(ethyne-2,1-diyl))bis(fluorobenzene) (**32$_{A}$**) in $CDCl_3$ at 25 ^{o}C.* 74

1.5.56 *^{13}C-NMR of 3,3'-((5-bromo-2-iodo-1,3-phenylene)bis(ethyne-2,1-diyl))bis(fluorobenzene) (**32$_{A}$**) in $CDCl_3$ at 25 ^{o}C.* 75

1.5.57 *^{1}H-NMR of 4,4'-((5-bromo-2-iodo-1,3-phenylene)bis(ethyne-2,1-diyl)) bis(ethylbenzene) (**33$_{A}$**) in $CDCl_3$ at 25 ^{o}C.* 76

1.5.58 *^{13}C-NMR of 4,4'-((5-bromo-2-iodo-1,3-phenylene)bis(ethyne-2,1-diyl)) bis(ethylbenzene) (**33$_{A}$**) in $CDCl_3$ at 25 ^{o}C.* 77

1.5.59 *^{1}H-NMR of 4,4'-((2-iodo-1,3-phenylene)bis(ethyne-2,1-diyl))bis ((trifluoromethyl)benzene) (**34$_{A}$**) in $CDCl_3$ at 25 ^{o}C.* 78

1.5.60 *^{13}C-NMR of 4,4'-((2-iodo-1,3-phenylene)bis(ethyne-2,1-diyl))bis ((trifluoromethyl)benzene) (**34$_{A}$**) in $CDCl_3$ at 25 ^{o}C.* 79

1.5.61 *^{1}H-NMR of 4,4'-((5-chloro-2-iodo-1,3-phenylene)bis(ethyne-2,1-diyl))bis((trifluoromethyl) benzene) (**35$_{A}$**) in $CDCl_3$ at 25 ^{o}C.* 80

1.5.62 *^{13}C-NMR of 4,4'-((5-chloro-2-iodo-1,3-phenylene)bis(ethyne-2,1-diyl))bis((trifluoromethyl) benzene) (**35$_{A}$**) in $CDCl_3$ at 25 ^{o}C.* 81

1.5.63 *^{1}H-NMR of 4,4''-dimethoxy-3'-(phenylethynyl)-1,1':2',1''-terphenyl (**36**) in $CDCl_3$ at 25 ^{o}C* 82

1.5.64 *^{13}C-NMR of 4,4''-dimethoxy-3'-(phenylethynyl)-1,1':2',1''-terphenyl (**36**) in $CDCl_3$ at 25 ^{o}C.* 83

1.5.65 *^{1}H-NMR of 3'-(phenylethynyl)-4,4''-bis(trifluoromethyl)-1,1':2',1''-terphenyl (**37**) in $CDCl_3$ at 25 ^{o}C.* 84

1.5.66 *^{13}C-NMR of 3'-(phenylethynyl)-4,4''-bis(trifluoromethyl)-1,1':2',1''-terphenyl (**37**) in $CDCl_3$ at 25 ^{o}C.* 85

1.6 **X-ray of new compounds** 86

1.6.1 *X-ray data of 1,2-diiodo-3-{[4-(trifluoromethyl)phenyl]ethynyl}benzene (**6$_{A}$**)* 86

1.6.2 *X-ray data of 5-chloro-1,2-diiodo-3-{[4-(trifluoromethyl)phenyl]ethynyl} benzene (**20$_{A}$**)* 97

1.6.3 *X-ray data of 5-fluoro-1,2-diiodo-3-[(4-methoxyphenyl)ethynyl]benzene (**23$_{A}$**)* 107

1.6.4 *X-ray data of 5-chloro-2-iodo-1,3-bis{[4-trifluoromethyl)phenyl]ethynyl} benzene (**35$_{A}$**)* 117

1. Experimental Details and Compound Data

1.1 General Information

All commercial reagents and chromatography solvents were used as obtained unless otherwise stated. Ethanol, toluene, ethyl acetate, hexanes, anhydrous sodium sulfate (Na_2SO_4, BDH), CuI (Sigma-Aldrich), $Pd(PPh_3)_4$ (Sigma-Aldrich) were used as received. Anhydrous solvents were distilled over appropriate drying agents prior to use. Analytical thin layer chromatography (TLC) was performed on Merck silica gel 60 F_{254}. Merck Silica gel 60 (0.063 - 0.2 mm) was used for column chromatography. Visualization of TLC was accomplished with UV light (254 nm). NMR spectra were recorded on a Bruker-Avance 400 MHz spectrometer. The residual solvent protons (^1H) or the solvent carbon (^{13}C) were used as internal standards. ^1H-NMR data are presented as follows: chemical shift in ppm (δ) downfield from trimethylsilane (multiplicity, integration, coupling constant). The following abbreviations are used in reporting NMR data: s, singlet; bs, broad singlet; d, doublet; t, triplet; q, quartet; dq, doublet of quartets; dd, doublet of doublets; m, mutiplet.. High resolution mass spectra were recorded using Chemical Ionization (CI) and Electrospray ionization (ESI) techniques.

1.2 General procedure for Sonogashira cross-coupling reactions of 5-substituted-1,2,3-triiodoarenes

A flame-dried Shlenk flask was charged with 5-substituted-1,2,3-triiodoarene (0.65 mmol, 0.08M, 1.0 equiv.), aryl acetylene (1.0 equiv.), Cs_2CO_3 (7.0 equiv.) and 8.0 mL dry toluene. The mixture was stirred under argon at room temperature for 20 mins. Tetrakis(triphenylphosphine)palladium(0) (10 mol-%) and copper iodide (20 mol-%) were added, capped with septum, carefully degassed with argon, and the

reaction flask was wrapped with aluminum foil and stirred at room temperature for 24 h. The reaction mixture was then diluted with EtOAc and filtered over Celite 545®. Distilled water (100 mL) was added and extracted with EtOAc (2 x 50 mL). The organic layers were combined, washed with brine, dried with anhyd. Na_2SO_4, filtered and evaporated under reduced pressure. The crude product was purified by flash chromatography (100% hexane) to yield the pure desired product.

1.2.1 Synthesis of 1,2-diiodo-3-(phenylethynyl)benzene (5_A)

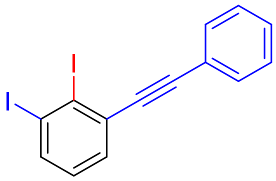

The title compound was synthesized using the general procedure and isolated in **60%** yield as colorless oil after flash chromatography. **IR** (cast film, cm^{-1}) 3087, 3046, 2201, 1599, 1523, 912, 837, 749, 624. **δ_H** (400 MHz, $CDCl_3$) δ: 7.81 (d, 1H, J = 7.9 Hz), 7.50-7.65 (m, 2H), 7.47 (d, 1H, J = 7.6 Hz), 7.30-7.40 (m, 3H), 7.03 (dd, 1H, J = 7.8 Hz). **δ_C** (100 MHz, $CDCl_3$) δ: 138.9, 131.9, 131.8, 131.3, 129.2, 129.0, 128.6, 122.8, 114.7, 108.9, 93.5, 93.1. **HRMS** (EI) m/z for $C_{14}H_8I_2$ $[M]^+$: calcd. 429.8715; found, 429.8709.

1.2.2 Synthesis of 1,2-diiodo-3-((4-(trifluoromethyl)phenyl) ethynyl) benzene (6_A)

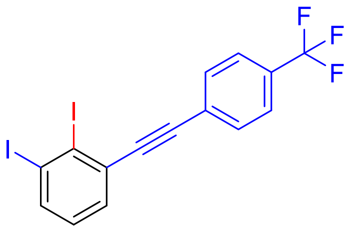

The title compound was synthesized using the general procedure and isolated in **51%** yield as white solid after flash chromatography. **IR** (cast film, cm^{-1}) 3109, 3046, 2204, 1588, 1541, 1042, 943, 812, 738, 661. **δ_H** (400 MHz, $CDCl_3$) δ: 7.85 (d, 1H, J = 7.9 Hz), 7.68 (d, 2H, J = 8.0 Hz), 7.62 (d, 2H, J = 8.2 Hz), 7.48 (d, 1H, J = 7.6), 7.06 (dd, 1H, J^1 = 7.8 Hz, J^2 = 7.7 Hz). **δ_C** (100 MHz, $CDCl_3$) δ: 139.5, 132.0, 131.6, 131.2, 130.7 (q, $J_{C\text{-}F}$ = 33 Hz), 129.3, 126.6, 125.5 (q, $J_{C\text{-}F}$ = 4

Hz), 124.0 (q, $J_{C\text{-}F}$ = 271 Hz), 114.8, 109.1, 95.6, 91.4. **M.p**: 92-94 °C. **HRMS** (EI) m/z for $C_{15}H_7F_3I_2$ [M]$^+$: calcd. 497.8589; found, 497.8576.

1.2.3 Synthesis of 1-((4-chlorophenyl)ethynyl)-2,3-diiodobenzene (7A)

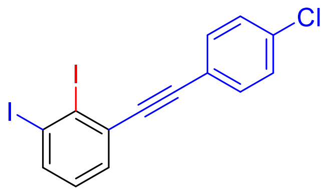

The title compound was synthesized using the general procedure and isolated in **37%** yield as white solid after flash chromatography. **IR** (cast film, cm^{-1}) 3086, 3012, 2203, 914, 824, 748, 638. **δH** (400 MHz, $CDCl_3$) δ: 7.81 (d, 1H, *J* = 7.8 Hz) , 7.40-7.60 (m, 3H), 7.32 (d, 2H, *J* = 8.4 Hz), 7.03 (dd, 1H, *J* = 7.8 Hz, , *J* = 7.7 Hz). **δC** (100 MHz, $CDCl_3$) δ: 139.2, 135.2, 133.0, 131.6, 131.4, 129.2, 129.0, 121.2, 114.7, 109.0, 94.4, 91.9. **M.p**: 101-103 °C. **HRMS** (EI) m/z for $C_{14}H_7ClI_2$ [M]$^+$: calcd. 463.8326; found, 463.8317.

1.2.4 Synthesis of 1,2-diiodo-3-((4-methoxyphenyl)ethynyl)benzene (8A)

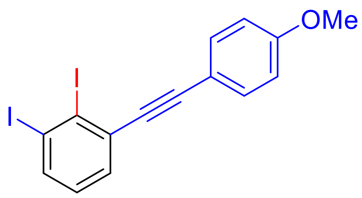

The title compound was synthesized using the general procedure and isolated in **41%** yield as white solid after flash chromatography. **IR** (cast film, cm^{-1}) 3087, 3024, 2215, 1606, 1546, 1324, 1157, 973, 843, 642. **δH** (400 MHz, $CDCl_3$) δ: 7.78 (d, 1H, *J* = 7.8 Hz), 7.51 (d, 2H, *J* = 8.7 Hz), 7.44 (d, 1H, *J* = 7.6 Hz), 7.02 (dd, 1H, J^1 = 7.7 Hz, , J^2 = 7.8 Hz), 6.89 (d, 2H, *J* = 8.7 Hz). **δC** (100 MHz, $CDCl_3$) δ: 160.3, 138.6, 133.3, 132.3, 131.1, 129.2, 114.9, 114.6, 114.3, 108.9, 93.4, 92.5, 55.51. **M.p**: 82-84 °C. **HRMS** (EI) m/z for $C_{15}H_{10}I_2O$ [M]$^+$: calcd. 459.8821; found, 459.8814.

1.2.5 Synthesis of 1,2-diiodo-5-methyl-3-(phenylethynyl)benzene (9_A)

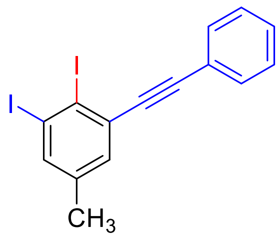

The title compound was synthesized using the general procedure and isolated in **51%** yield as colorless oil after flash chromatography. **IR** (cast film, cm^{-1}) 3086, 3015, 2209, 1604, 1573, 784, 652, 507. **δ_H** (400 MHz, $CDCl_3$) δ: 7.67 (d, 1H, *J* = 1.1 Hz) , 7.56-7.59 (m, 2H), 7.36-7.37 (m, 3H), 7.30 (d, 1H, *J* = 1.1 Hz), 2.24 (s, 3H). **δ_C** (100 MHz, $CDCl_3$) δ: 139.9, 139.5, 132.4, 131.8, 131.3, 129.0, 128.6, 122.8, 110.5, 108.8, 93.5, 92.7,20.4. **HRMS** (EI) m/z for $C_{15}H_{10}I_2$ $[M]^+$: calcd. 443.8872; found, 443.8867.

1.2.6 Synthesis of 1-((4-ethylphenyl)ethynyl)-2,3-diiodo-5-methyl benzene (10_A)

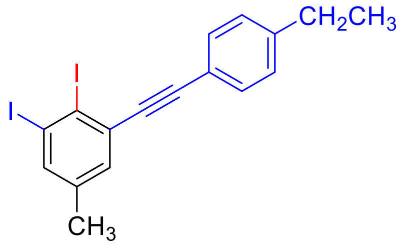

The title compound was synthesized using the general procedure and isolated in **85%** yield as colorless oil after flash chromatography. **IR** (cast film, cm^{-1}) 3104, 3086, 3042, 2198, 1594, 1546, 964, 862, 779, 634. **δ_H** (400 MHz, $CDCl_3$) δ: 7.65 (s, 1H) , 7.49 (d, 2H, *J* = 8.0 Hz), 7.29 (d, 1H, *J* = 1.0 Hz), 7.19 (d, 2H, *J* = 8.0 Hz), 2.64-2.70 (m, 2H), 2.23 (s, 3H), 1.23-1.27 (m, 3H). **δ_C** (100 MHz, $CDCl_3$) δ:145.5, 139.7, 139.5, 132.7, 132.3, 131.8, 131.5, 128.1, 120.0, 110.5, 108.7, 93.0, 29.1, 20.4, 15.5. **HRMS** (EI) m/z for $C_{17}H_{14}I_2$ $[M]^+$: calcd. 471.9185; found, 471.9182.

1.2.7 Synthesis of 1,2-diiodo-3-((4-methoxyphenyl)ethynyl)-5-methyl benzene (11$_A$)

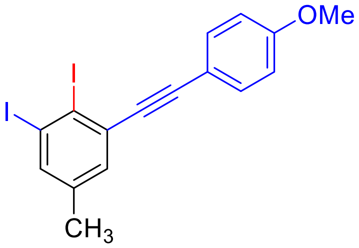

The title compound was synthesized using the general procedure and isolated in **42%** yield as white solid after flash chromatography. **IR** (cast film, cm^{-1}) 3107, 3056, 2205, 1614, 1573, 1168, 1021, 983, 673. **δ_H** (400 MHz, $CDCl_3$) δ: 7.64 (s, 1H), 7.51 (d, 2H, *J* = 8.7 Hz), 7.27 (s, 1H), 6.89 (d, 2H, *J* = 8.7 Hz), 3.83 (s, 3H), 2.23 (s, 3H). **δ_C** (100 MHz, $CDCl_3$) δ: 160.2, 139.5, 139.4, 133.3, 132.1, 131.6, 114.9, 114.2, 110.4, 108.7, 92.9, 92.5, 55.5, 20.4. **M.p**: 91-93 °C **HRMS** (EI) m/z for $C_{16}H_{12}I_2O$ $[M]^+$: calcd. 473.8978; found, 473.8974.

1.2.8 Synthesis of 1,2-diiodo-5-methoxy-3-((4-methoxyphenyl) ethynyl) benzene (12$_A$)

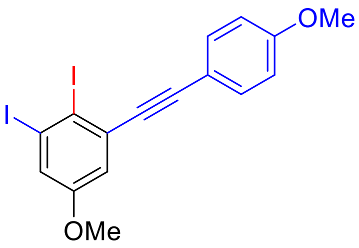

The title compound was synthesized using the general procedure and isolated in **51%** yield as white solid after flash chromatography. **IR** (cast film, cm^{-1}) 3042, 3012, 2195, 1594, 1548, 1209, 1168, 1023, 918, 652. **δ_H** (400 MHz, $CDCl_3$) δ: 7.52 (d, 2H, *J* = 8.6 Hz), 7.39 (d, 1H, *J* = 2.8 Hz), 7.02 (d, 1H, *J* = 2.7 Hz), 6.89 (d , 2H, *J* = 8.6 Hz), 3.83 (s, 3H), 3.78 (s, 3H). **δ_C** (100 MHz, $CDCl_3$) δ:160.3, 159.4, 133.3, 131.9, 125.6, 117.1, 114.7, 114.3, 108.8, 103.6, 93.1, 92.5, 55.8, 55.5. **M.p**: 83-85 °C. **HRMS** (EI) m/z for $C_{16}H_{12}I_2O_2$ $[M]^+$: calcd. Exact 489.8927; found 489.8921.

1.2.9 Synthesis of 1-((4-ethylphenyl)ethynyl)-2,3-diiodo-5-methoxy benzene (13A)

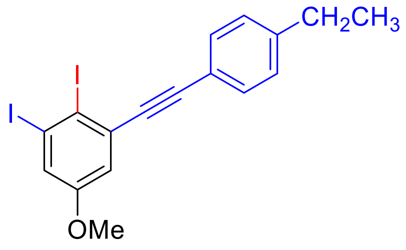

The title compound was synthesized using the general procedure and isolated in **29%** yield as white solid after flash chromatography. **IR** (cast film, cm^{-1}) 3059, 3024, 2212, 1584, 1542, 1145, 1048, 956, 768. **δ_H** (400 MHz, $CDCl_3$) δ: 7.50 (d, 2H, *J* = 8.0 Hz) , 7.40 (d, 1H, *J* = 2.8), 7.20 (d, 2H, *J* = 7.8), 7.04 (d, 1H, *J* = 2.8), 3.78 (s, 3H), 2.6-2.7 (m, 2H), 1.24 (t, 3H, *J* = 7.6). **δ_C** (100 MHz, $CDCl_3$) δ: 159.4, 145.7, 131.8, 128.2, 125.8, 119.8, 117.3, 108.8, 103.7, 93.2, 93.0, 55.8, 29.1, 15.5, (missing one peak due to overlapping). **M.p**: 96-98 °C. **HRMS** (EI) m/z for $C_{17}H_{14}I_2O$ $[M]^+$: calcd. 487.9134; found, 487.9121.

1.2.10 Synthesis of 1,2-diiodo-5-methoxy-3-((4-(trifluoromethyl) phenyl) ethynyl)benzene (14A)

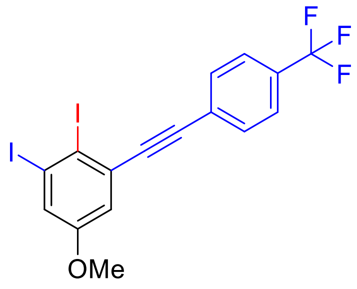

The title compound was synthesized using the general procedure and isolated in **36%** yield as white solid after flash chromatography. **IR** (cast film, cm^{-1}) 3102, 3075, 3005, 2209, 1597, 1578, 1310, 1125, 993, 867, 631, 523. **δ_H** (400 MHz, $CDCl_3$) δ: 7.68 (d, 2H, *J* = 8.2 Hz), 7.62 (d, 2H, *J* = 8.4 Hz), 7.44 (d, 1H, *J* = 2.9 Hz), 7.06 (d, 1H, *J* = 2.8 Hz), 3.8 (s, 3H). **δ_C** (100 MHz, $CDCl_3$) δ: 159.5, 132.1, 130.9, 130.8 (q, $J_{C\text{-}F}$ = 30 Hz), 126.5, 126.4, 125.5 (q, $J_{C\text{-}F}$ = 4 Hz), 124.0 (q, $J_{C\text{-}F}$ = 270 Hz), 117.7, 109.1, 103.7, 95.5, 91.1, 55.9. **M.p**: 92-94 °C. **HRMS** (EI) m/z for $C_{16}H_9F_3I_2O$ $[M]^+$: calcd. 527.8695; found, 527.8682.

1.2.11 Synthesis of 1-((4-fluorophenyl)ethynyl)-2,3-diiodo-5-methoxy benzene (15A)

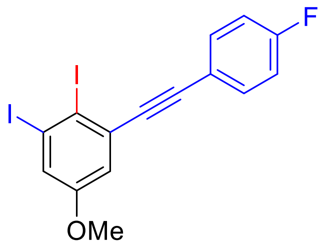

The title compound was synthesized using the general procedure and isolated in **27%** yield as white solid after flash chromatography. **IR** (cast film, cm^{-1}) 3104, 3078, 2204, 1608, 1599, 1359, 1146, 984, 873, 749, 653. **δH** (400 MHz, $CDCl_3$) δ: 7.54-7.58 (m, 2H) , 7.41 (d, 1H, *J* = 2.9 Hz), 7.07 (t, 2H, *J* = 8.7 Hz), 7.03 (d, 1H, *J* = 2.8 Hz), 3.78 (s, 3H). **δC** (100 MHz, $CDCl_3$) δ: 163.0 (d, J_{C-F} = 149 Hz), 159.4, 133.8, 133.8 (d, J_{C-F} = 9 Hz), 125.9, 118.8 (d, J_{C-F} = 4 Hz), 117.4, 115.9 (d, J_{C-F} = 21 Hz), 108.9, 103.6, 93.1, 91.7, 55.8. **M.p**: 70-72 °C. **HRMS** (EI) m/z for $C_{15}H_9FI_2O$ $[M]^+$: calcd. 477.8727; found, 477.8713.

1.2.12 Synthesis of 4-((2,3-diiodo-5-methoxyphenyl)ethynyl)-1,1'-biphenyl (16A)

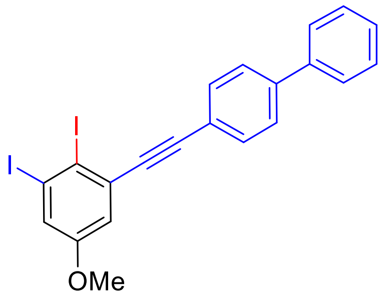

The title compound was synthesized using the general procedure and isolated in **51%** yield white solid after flash chromatography. **IR** (cast film, cm^{-1}) 3145, 3048, 2213, 1609, 1588, 1189, 1077, 961, 632. **δH** (400 MHz, $CDCl_3$) δ: 7.55-7.70 (m, 5H) , 7.47 (dd, 2H, J^1 = 7.3 Hz, J^2 = 7.8 Hz) , 7.42 (d, 1H, *J* = 2.8 Hz), 7.37 (dd, 2H, J^1 = 7.4 Hz, J^2 = 7.2 Hz) , 7.07 (d, 1H, *J* = 2.8) , 3.80 (s, 3H). **δC** (100 MHz, $CDCl_3$) δ:159.5, 141.8, 140.4, 132.3, 131.7, 129.1, 127.9, 127.3, 127.2, 125.9, 121.5, 117.4, 108.9, 103.8, 94.1, 92.8, 55.9. **M.p**: 85-87 °C. **HRMS** (EI) m/z for $C_{21}H_{14}I_2O$ $[M]^+$: calcd. 535.9134; found, 535.9119.

1.2.13 Synthesis of 5-chloro-1,2-diiodo-3-(phenylethynyl)benzene (17A)

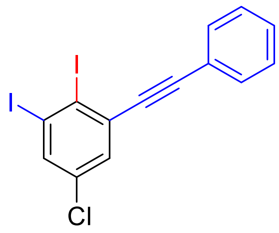

The title compound was synthesized using the general procedure and isolated in **42%** yield as white solid after flash chromatography. **IR** (cast film, cm^{-1}) 3104, 3086, 2207, 1602, 1071, 943, 827, 634. **δ_H** (400 MHz, $CDCl_3$) δ: 7.79 (d, 1H, *J* = 2.4 Hz) , 7.57-7.59 (m, 2H), 7.46 (d, 1H, *J* = 2.3 Hz), 7.38-7.39 (m, 3H). **δ_C** (100 MHz, $CDCl_3$) δ: 138.3, 134.6, 132.6, 131.9, 131.2, 129.4, 128.6, 122.3, 112.5, 109.2, 94.3, 92.5. **M.p**: 90-92. °C. **HRMS** (EI) m/z for $C_{14}H_7ClI_2$ $[M]^+$: calcd. 463.8326; found, 463.8323.

1.2.14 Synthesis of 5-chloro-1-((4-ethylphenyl)ethynyl)-2,3-diiodobenzene (18A)

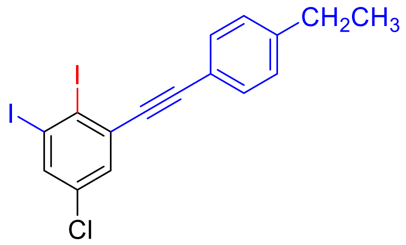

The title compound was synthesized using the general procedure and isolated in **34%** yield as white solid after flash chromatography. **IR** (cast film, cm^{-1}): 3104, 3089, 3018, 2212, 1579, 1542, 928, 813, 742, 642. **δ_H** (400 MHz, $CDCl_3$) δ: 7.78 (d, 1H, *J* = 2.2 Hz), 7.49 (d, 2H, *J* = 8.0 Hz), 7.45 (d, 1H, *J* = 2.2 Hz), 7.21 (d, 2H, *J* = 7.9 Hz), 2.67 (q, 2H, *J* = 7.5 Hz), 1.25 (t, 3H, *J* = 7.6 Hz). **δ_C** (100 MHz, $CDCl_3$) δ: 146.0, 138.0, 134.5, 132.8, 131.9, 131.0, 128.2, 119.4, 112.5, 109.2, 94.6, 92.0, 29.1, 15.5. **M.p**: 87-89 °C. **HRMS** (EI) m/z for $C_{16}H_{11}ClI_2$ $[M]^+$: calcd. 491.8639; found, 491.8632.

1.2.15 Synthesis of 5-chloro-1,2-diiodo-3-((4-methoxyphenyl) ethynyl) benzene (19A)

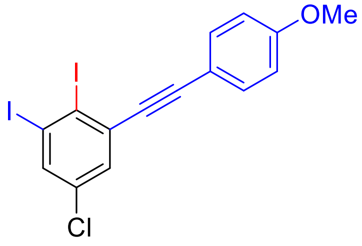

The title compound was synthesized using the general procedure and isolated in **44%** yield as white solid after flash chromatography. **IR** (cast film, cm^{1}): 3121, 3049, 2209, 1613, 1597, 1310, 1149, 976, 842, 742, 619. **δ_H** (400 MHz, $CDCl_3$) δ: 7.76 (d, 1H, *J* = 2.2 Hz), 7.51 (d, 2H, *J* = 8.7 Hz), 7.43 (d, 1H, *J* = 2.2 Hz), 6.89 (d, 2H, *J* = 8.8 Hz), 3.48 (s, 3H). **δ_C** (100 MHz, $CDCl_3$) δ: 160.6, 137.9, 134.5, 133.6, 133.5, 132.9, 130.9, 114.3, 112.3, 109.1, 94.6, 91.6, 55.5. **M.p**: 112-114 °C. **HRMS** (EI) m/z for $C_{15}H_9ClI_2O$ $[M]^+$: calcd. 493.8431; found, 493.8425.

1.2.16 Synthesis of 5-chloro-1,2-diiodo-3-((4-(trifluoromethyl)phenyl) ethynyl) benzene (20A)

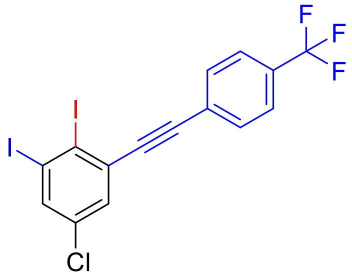

The title compound was synthesized using the general procedure and isolated in **31%** yield as white solid after flash chromatography. **IR** (cast film, cm^{-1}) 3107, 3088, 3012, 2196, 1612, 1579, 1012, 983, 867, 764, 642, 521. **δ_H** (400 MHz, $CDCl_3$) δ: 7.81 (d, 1H, *J* = 2.4 Hz,) , 7.60-7.70 (m, 4H) , 7.46 (d, 1H, *J* = 2.4 Hz). **δ_C** (100 MHz, $CDCl_3$) δ: 138.8, 134.7, 133.0, 132.1, 131.8, 131.4, 131.0 (q, *J* = 32 Hz), 125.6 (q, *J* = 4 Hz), 123.9 (q, J_{C-F} = 271 Hz), 112.6, 109.5, 94.4, 92.4. **M.p**: 116-118 °C. **HRMS** (EI) m/z for $C_{15}H_6ClF_3I_2$ $[M]^+$: calcd. 531.8199; found, 531.8188.

1.2.17 Synthesis of 5-bromo-1-((4-ethylphenyl)ethynyl)-2,3-diiodobenzene (21_A)

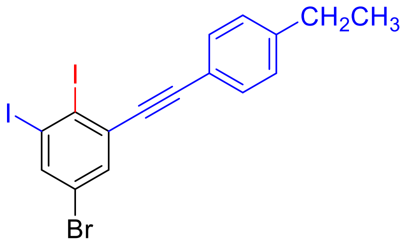

The title compound was synthesized using the general procedure and isolated in in **85%** yield colorless oily after flash chromatography. **IR** (cast film, cm^{1}): 3142, 3048, 3013, 2201, 1592, 943, 841, 746, 529. **δ_H** (400 MHz, $CDCl_3$) δ: 7.93 (d, 1H, *J* = 2.2 Hz), 7.67 (d, 1H, *J* = 2.2 Hz), 7.47 (d, 2H, *J* = 8.1 Hz), 7.20 (d, 2H, *J* = 8.0 Hz), 2.68 (q, 2H, *J* = 7.6 Hz), 1.25 (t, 3H, *J* = 7.6 Hz). **δ_C** (100 MHz, $CDCl_3$) δ: 146.0, 141.6, 135.4, 132.0, 128.2, 125.5, 120.1, 119.4, 99.3, 96.7, 85.0, 29.1, 15.5, (missing one peak due to overlapping). **HRMS** (EI) m/z for $C_{16}H_{11}BrI_2$ $[M]^+$: calcd. 535.8133; found, 535.8128.

1.2.18 Synthesis of 5-bromo-1-((3-fluorophenyl)ethynyl)-2,3-diiodo benzene (22_A)

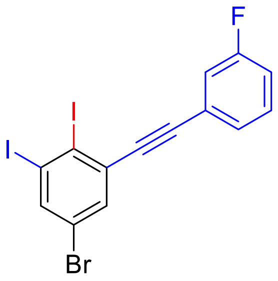

The title compound was synthesized using the general procedure and isolated in **44%** yield as white solid after flash chromatography. **IR** (cast film, cm^{-1}) 3097, 3048, 2214, 1598, 948, 812, 794, 653. **δ_H** (400 MHz, $CDCl_3$) δ: 7.95 (d, 1H, *J* = 2.2 Hz) , 7.66 (d, 1H, *J* = 2.2 Hz), 7.32-7.34 (m, 2H), 7.23 (d, 1H, *J* = 9.2 Hz), 7.10-7.11 (m, 1H,). **δ_C** (100 MHz, $CDCl_3$) δ: 161.5 (d, $J_{C\text{-}F}$ = 246 Hz), 142.2, 139.3, 135.6, 130.2 (d, $J_{C\text{-}F}$ = 8 Hz), 129.9 (d, $J_{C\text{-}F}$ = 3 Hz), 124.8, 124.0 (d, $J_{C\text{-}F}$ = 10 Hz), 120.2, 118.8 (d, $J_{C\text{-}F}$ = 22 Hz), 116.8 (d, $J_{C\text{-}F}$ = 22 Hz), 99.4, 94.8 (d, $J_{C\text{-}F}$ = 3 Hz), 86.2. **M.p**: 68-70 °C **HRMS** (EI) m/z for $C_{14}H_6BrFI_2$ $[M]^+$: calcd. 525.7726; found, 525.7719.

1.2.19 Synthesis of 5-fluoro-1,2-diiodo-3-((4-methoxyphenyl)ethynyl) benzene (23A)

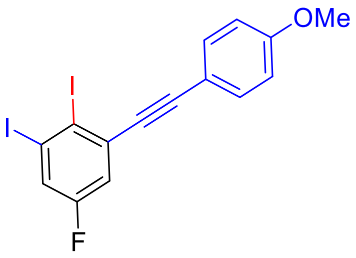

The title compound was synthesized using the general procedure and isolated in **74%** yield as white solid after flash chromatography. **IR** (cast film, cm^{-1}) 3102, 3042, 2191, 1608, 1597, 1023, 894, 742, 691. **δ_H** (400 MHz, $CDCl_3$) δ: 7.51-7.57 (m, 3H), 7.20 (dd, 1H, J^1 = 2.7 Hz, J^2 = 8.6 Hz), 6.90 (d, 2H, J = 8.6 Hz), 3.84 (s, 3H). **δ_C** (100 MHz, $CDCl_3$) δ: 161.6 (d, $J_{C\text{-}F}$ = 251 Hz), 160.6, 133.5, 132.9 (d, $J_{C\text{-}F}$ = 10 Hz), 130.9, 126.3 (d, $J_{C\text{-}F}$ = 24 Hz), 118.4 (d, $J_{C\text{-}F}$ = 23 Hz), 108.7 (d, $J_{C\text{-}F}$ = 4 Hz), 108.6, 108.5, 94.5, 91.8 (d, $J_{C\text{-}F}$ = 3 Hz), 55.5. **M.p**: 87-90 °C. **HRMS** (EI) m/z for $C_{15}H_9FI_2O$ $[M]^+$: calcd. 477.8727; found, 477.8718.

1.2.20 Synthesis of 1-((4-chlorophenyl)ethynyl)-5-fluoro-2,3-diiodo benzene (24A)

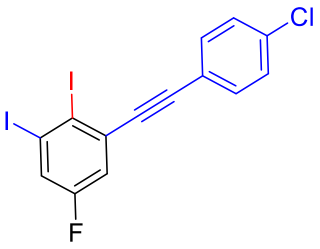

The title compound was synthesized using the general procedure and isolated in **42%** yield as white solid after flash chromatography. **IR** (cast film, cm^{-1}) 3089, 3041, 2201, 1597, 1524, 1016, 927, 813, 691. **δ_H** (400 MHz, $CDCl_3$) δ: 7.57 (dd, 1H, J^1 = 7.7 Hz, J^2 = 2.4 Hz), 7.48 (d, 2H, J = 8.3 Hz), 7.34 (d, 2H, J = 8.2 Hz), 7.20 (dd, 1H, J^1 = 8.6 Hz, J^2 = 2.4 Hz). **δ_C** (100 MHz, $CDCl_3$) δ: 161.5 (d, $J_{C\text{-}F}$ = 252 Hz), 135.5, 133.1, 132.1 (d, $J_{C\text{-}F}$ = 10 Hz), 129.0, 126.8 (d, $J_{C\text{-}F}$ = 24 Hz), 120.8, 118.8 (d, $J_{C\text{-}F}$ = 23 Hz), 108.9 (d, $J_{C\text{-}F}$ = 4 Hz), 108.8 (d, $J_{C\text{-}F}$ = 8 Hz), 93.5 (d, $J_{C\text{-}F}$ = 3 Hz), 92.9. **M.p**: 113-114 °C. **HRMS** (EI) m/z for $C_{14}H_6ClFI_2$ $[M]^+$: calcd. 481.8231; found, 481.8228.

1.2.21 Synthesis of methyl 3,4-diiodo-5-((4-methoxyphenyl)ethynyl) benzoate (25A)

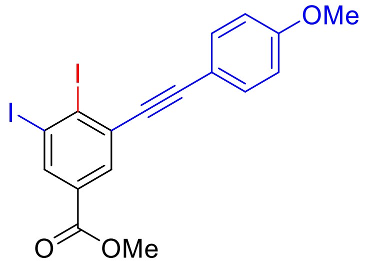

The title compound was synthesized using the general procedure and isolated in **33%** yield as a white solid after flash chromatography. **IR** (cast film, cm^{-1}) 3104, 3085, 2195, 1765, 1604, 1586, 1415, 1204, 1112, 876, 743. **δH** (400 MHz, $CDCl_3$) δ: 8.37 (d, 1H, *J* = 1.8 Hz), 8.05 (d, 1H, *J* = 1.8 Hz), 7.52 (d, 2H, *J* = 8.8 Hz), 6.90 (d, 2H, *J* = 8.7 Hz), 3.92 (s, 3H), 3.84 (s, 3H). **δC** (100 MHz, $CDCl_3$) δ: 165.1, 160.5, 138.7, 133.4, 132.5, 131.5, 131.1, 120.6, 114.5, 114.3, 108.9, 94.4, 91.9, 55.5, 52.8. **M.p**: 173-175 °C. **HRMS** (EI) m/z for $C_{17}H_{12}I_2O_3$ $[M]^+$: calcd. 517.8876; found, 517.8874.

1.2.22 Synthesis of 1-chloro-3-((4-chlorophenyl)ethynyl)-2-iodobenzene (26A)

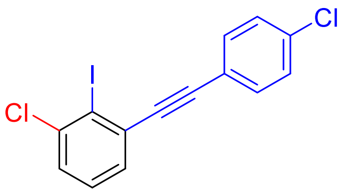

The title compound was synthesized using the general procedure and isolated in **45%** yield as white solid after flash chromatography. **IR** (cast film, cm^{-1}) 3078, 3024, 2208, 1601, 1598, 1107, 867, 523. **δH** (400 MHz, $CDCl_3$) δ: 7.52 (d, 2H, *J* = 8.4 Hz), 7.34-7.40 (m, 4H), 7.26 (dd, 1H, *J* = 7.7 Hz, *J* = 7.9 Hz). **δC** (100 MHz, $CDCl_3$) δ: 139.8, 135.2, 133.9, 133.0, 132.5, 130.4, 129.0, 128.9, 121.3, 105.5, 93.0, 92.5. **M.p**: 90-92 °C. **HRMS** (EI) m/z for $C_{14}H_7Cl_2I$ $[M]^+$: calcd. 371.8969; found, 371.8960.

1.2.23 Synthesis of 1-bromo-2-iodo-3-((4-methoxyphenyl)ethynyl)benzene (27A)

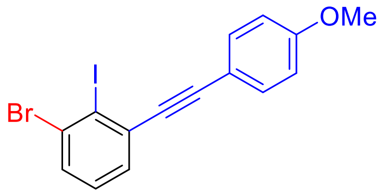

The title compound was synthesized using the general procedure and isolated in **62%** yield as white solid after flash chromatography. **IR** (cast film, cm^{-1}) 3097, 3021, 2207, 1578, 1543, 1204, 1079, 894, 742. δ_H (400 MHz, $CDCl_3$) δ: 7.53 (d, 3H, *J* = 8.2 Hz), 7.41 (d, 1H, *J* = 7.6 Hz), 7.17 (dd, 1H, J^1 = 7.9, J^2 = 7.8 Hz), 6.90 (d, 2H, *J* = 8.3 Hz), 3.84 (s, 3H). δ_C (100 MHz, $CDCl_3$) δ: 160.3, 133.3, 133.2, 131.8, 130.7, 130.5, 129.1, 114.8, 114.3, 108.3, 93.9, 91.6, 55.5. **M.p**: 53-55 °C. **HRMS** (EI) m/z for $C_{15}H_{10}BrIO$ $[M]^+$: calcd. 411.8960; found, 411.8954.

1.3 General procedure for one-pot double Sonogashira cross-coupling reactions of 5-substituted-1,2,3-triiodoarenes

A flame-dried Shlenk flask was charged with 5-substituted-1,2,3-triiodoarene (0.65 mmol, 0.08M, 1.0 equiv.), aryl acetylene (1.0 equiv.), Cs_2CO_3 (7.0 equiv) in 8.0 mL dry toluene under argon. The mixture was stirred at room temperature for 20 mins. Tetrakis(triphenylphosphine)palladium (0) (10 mol %) and copper iodide (20 mol %) were added, capped with septum, carefully degassed with argon, and the reaction flask was wrapped with aluminum foil and stirred at room temperature for 24 h. Aryl acetylene (1.0 equiv.) and Cs_2CO_3 (7.0 equiv) were added to the mixture, carefully degassed with argon, wrapped with aluminum foil and stirred at room temperature for another 24 h. The reaction mixture was diluted with EtOAc and filtered over Celite 545®. Distilled water (100 mL) was added and extracted with EtOAc (2 x 50 mL). The organic layers were combined washed with brine, dried with anhyd. Na_2SO_4, filtered and evaporated under reduced pressure. The crude product

was purified by flash chromatography (100% hexane) to yield the pure desired product.

1.3.1 Synthesis of 2-iodo-5-methoxy-1-((4-methoxyphenyl)ethynyl)-3-((4-(trifluoromethyl)phenyl)ethynyl)benzene (28_A)

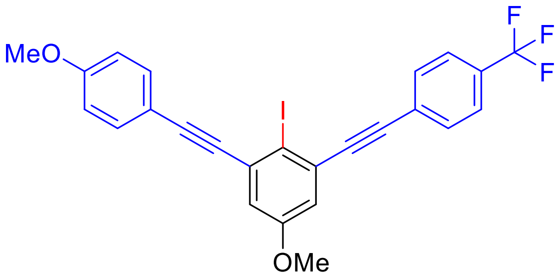

The title compound was synthesized using the one-pot general procedure and isolated in **48%** yield as pale yellow oil after flash chromatography. **IR** (cast film, cm^{-1}) 3124, 3086, 3006, 2203, 1601, 1583, 1204, 1139, 976, 867, 647. **δ_H** (400 MHz, $CDCl_3$) δ: 7.71 (d, 2H, *J* = 8.1 Hz), 7.62 (d, 2H, *J* = 8.1 Hz), 7.56 (d, 2H, *J* = 8.5 Hz), 7.06 (d, 1H , *J* = 2.7 Hz), 7.04 (d, 1H , *J* = 2.8 Hz), 6.90 (d, 2H , *J* = 8.5 Hz), 3.84 (s, 3H), 3.82 (s, 3H). **δ_C** (100 MHz, $CDCl_3$) δ: 160.3, 159.2, 133.4, 131.5 (q, J_{C-F} = 134 Hz), 132.0, 130.5 (q, J_{C-F} = 33 Hz), 126.8, 125.5,-125.4 (q, J_{C-F} = 4 Hz), 122.7, 118.2, 118.0, 114.9, 114.3, 97.0, 94.3, 93.7, 91.3, 90.8, 55.8, 55.5. **HRMS** (EI) m/z for $C_{25}H_{16}F_3IO_2$ $[M]^+$: calcd. 532.0147; found, 532.0133.

1.3.2 Synthesis of 5-bromo-1-((4-ethylphenyl)ethynyl)-3-((3-fluorophenyl) ethynyl)-2-iodobenzene (29_A)

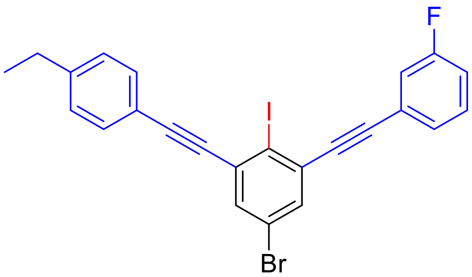

The title compound was synthesized using the one-pot general procedure and isolated in **34%** yield as white solid after flash chromatography. **IR** (cast film, cm^{-1}) 3125, 3073, 3041, 2201, 1623, 1579, 1042, 941, 837, 622. **δ_H** (400 MHz, $CDCl_3$) δ: 7.64 (dd, 2H, J^1 = 10.9 Hz, J^2 = 2.1 Hz), 7.50 (d, 2H, *J* = 7.9 Hz), 7.28-7.36 (m, 3H), 7.21 (d, 2H , *J* = 7.8 Hz), 7.00-7.15 (m, 1H), 2.67 (q, 2H , *J* = 7.6 Hz), 1.26 (m, 3H). **δ_C** (100 MHz, $CDCl_3$) δ: 162.5 (d, J_{C-F} = 245 Hz), 136.6, 135.4, 135.0, 132.0, 130.2 (d, J_{C-F} = 8 Hz), 128.2, 127.9 (d, J_{C-F} = 2

Hz), 126.0, 125.1, 124.3 (d, J_{C-F} = 9 Hz), 119.5, 119.4, 118.7 (d, J_{C-F} = 23 Hz), 116.6 (d, J_{C-F} = 21 Hz), 96.8, 94.6, 85.8, 84.3, 29.1, 15.5, (missing one peak due to overlapping). **M.p**: 83-85 °C. **HRMS** (EI) m/z for $C_{24}H_{15}BrFI$ $[M]^+$: calcd. 527.9386; found, 527.9382.

1.3.3 Synthesis of 4,4'-((2-iodo-1,3-phenylene)bis(ethyne-2,1-diyl))bis (methoxybenzene) (30$_A$)

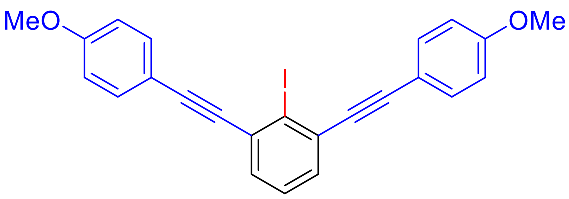

The title compound was synthesized using the one-pot general procedure and isolated in **57%** yield as a pale yellow oil after flash chromatography. **IR** (cast film, cm^{-1}) 3124, 3082, 3012, 2214, 1624, 1602, 967, 962, 841, 723. **δ$_H$** (400 MHz, $CDCl_3$) δ: 7.55 (d, 4H, J = 8.7 Hz), 7.40 (d, 2H, J = 7.5 Hz), 7.28 (d, 1H, J = 8.0 Hz), 6.90 (d, 4H, J = 8.7 Hz), 3.8 (s, 6H). **δ$_C$** (100 MHz, $CDCl_3$) δ: 160.2, 133.3, 131.5, 131.2, 127.8, 115.2, 114.3, 107.7, 93.5, 91.2, 55.5. **HRMS** (EI) m/z for $C_{24}H_{17}IO_2$ $[M]^+$: calcd. 464.0273; found, 464.0269.

1.3.4 Synthesis of 4,4'-((2-iodo-5-methoxy-1,3-phenylene)bis(ethyne-2,1-diyl))bis(methoxybenzene) (31$_A$)

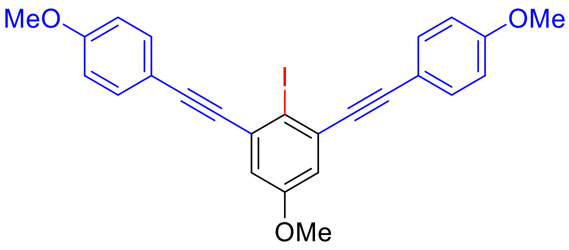

The title compound was synthesized using the one-pot general procedure and isolated in **69%** yield as colorless oil after flash chromatography. **IR** (cast film, cm^{-1}) 3124, 3075, 3042, 2198, 1614, 1587, 1345, 1207, 1184, 986, 748, 625. **δ$_H$** (400 MHz, $CDCl_3$) δ: 7.55 (d, 4H, J = 8.6 Hz), 7.02 (s, 2H), 6.90 (d, 4H, J = 8.6 Hz), 3.84 (s, 6H), 3.82 (s, 3H). **δ$_C$** (100 MHz, $CDCl_3$) δ: 160.2, 159.2, 133.4, 131.9, 117.5, 115.1,

114.3, 96.9, 93.3, 91.1, 55.8, 55.5. **HRMS** (EI) m/z for $C_{25}H_{19}IO_3$ $[M]^+$: calcd. 494.0379; found, 494.0371.

1.3.5 Synthesis of 3,3'-((5-bromo-2-iodo-1,3-phenylene)bis(ethyne-2,1-diyl))bis(fluorobenzene) (32$_A$)

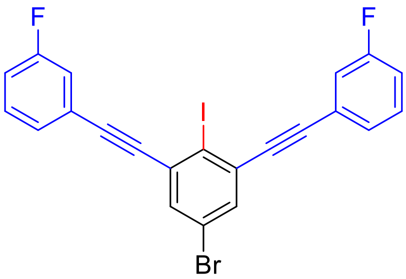

The title compound was synthesized using the one-pot general procedure and isolated in **55%** yield as a white solid after flash chromatography. **IR** (cast film, cm^{-1}) 3124, 3057, 2195, 1604, 1578, 684, 742, 628. **δ$_H$** (400 MHz, $CDCl_3$) δ: 7.66 (s, 2H), 7.33-7.36 (m, 4H), 7.22-7.30 (m, 2H), 7.07-7.12 (m, 2H). **δ$_C$** (100 MHz, $CDCl_3$) δ: 162.6 (d, J_{C-F} = 245 Hz), 136.8, 135.5, 130.3 (d, J_{C-F} = 8 Hz), 127.9 (d, J_{C-F} = 2 Hz), 125.4, 124.2 (d, J_{C-F} = 9 Hz), 119.5, 118.8 (d, J_{C-F} = 22 Hz), 116.7 (d, J_{C-F} = 21 Hz), 94.9 (d, J_{C-F} = 3 Hz), 85.6. **M.p**: 122-124 °C. **HRMS** (EI) m/z for $C_{22}H_{10}BrF_2I$ $[M]^+$: calcd. 517.8979; found, 517.8975.

1.3.6 Synthesis of 4,4'-((5-bromo-2-iodo-1,3-phenylene)bis(ethyne-2,1-diyl)) bis(ethylbenzene) (33$_A$)

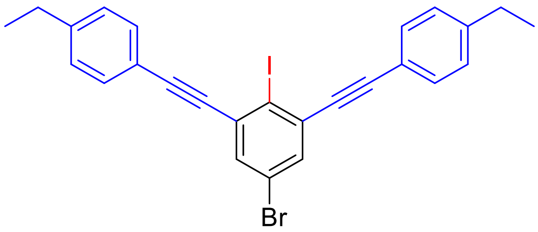

The title compound was synthesized using the one-pot general procedure and isolated in **87%** yield as colorless oil after flash chromatography. **IR** (cast film, cm^{-1}) 3106, 3086, 3015, 2186, 1612, 1592, 837, 467. **δ$_H$** (400 MHz, $CDCl_3$) δ: 7.61 (s, 2H), 7.44 (d, 4H, J = 7.8 Hz), 7.16-7.21 (m, 4H), 2.60-2.75 (m, 4H), 1.20-1.30 (m, 6H). **δ$_C$** (100 MHz, $CDCl_3$) δ: 145.5, 133.8, 132.7, 131.9, 128.2, 125.7, 119.9, 91.6, 86.7, 81.7, 29.0, 15.4. **HRMS** (EI) m/z for $C_{26}H_{20}BrI$ $[M]^+$: calcd. 537.9793; found, 537.9784.

1.3.7 Synthesis of 4,4'-((2-iodo-1,3-phenylene)bis(ethyne-2,1-diyl))bis((trifluoromethyl)benzene) (34_A)

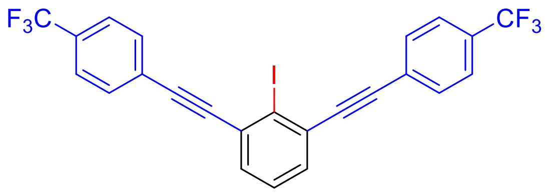

The title compound was synthesized using the one-pot general procedure and isolated in **72%** yield as a white solid after flash chromatography. **IR** (cast film, cm^{-1}) 3108, 3086, 3046, 2216, 1587, 1537, 976, 891, 642. **δ_H** (400 MHz, $CDCl_3$) δ: 7.72 (d, 4H, J = 8.1 Hz), 7.64 (d, 4H, J = 8.2 Hz), 7.50 (d, 2H, J = 7.6 Hz), 7.36 (dd, 1H, J^1 = 7.9, J^2 = 7.5 Hz). **δ_C** (100 MHz, $CDCl_3$) δ: 132.5, 132.1, 130.9, 130.8, 128.1, 126.7, 125.6 (q, $J_{C\text{-}F}$ = 4 Hz), 122.7, 108.1, 94.1, 91.9. **M.p**: 92-94 °C. **HRMS** (EI) m/z for $C_{24}H_{11}F_6I$ $[M]^+$: calcd. 539.9810; found, 539.9798.

1.3.8 Synthesis of 4,4'-((5-chloro-2-iodo-1,3-phenylene)bis(ethyne-2,1-diyl))bis((trifluoromethyl)benzene) (35_A)

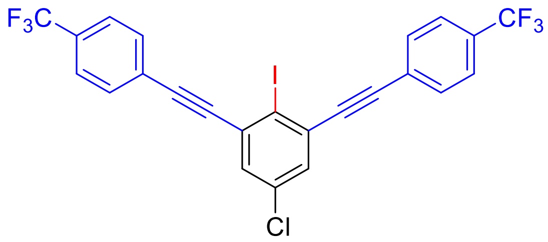

The title compound was synthesized using the one-pot general procedure and isolated in **55%** yield as a white solid after flash chromatography. **IR** (cast film, cm^{-1}) 3097, 3064, 2207, 1599, 1548, 948, 816, 724, 634. **δ_H** (400 MHz, $CDCl_3$) δ: 7.70 (d, 4H, J = 8.3 Hz), 7.64 (d, 4H, J = 8.9 Hz), 7.47 (s, 2H). **δ_C** (100 MHz, $CDCl_3$) δ: 138.8, 134.3, 133.0, 132.2 (q, $J_{C\text{-}F}$ = 34 Hz), 132.1 (q, $J_{C\text{-}F}$ = 3 Hz), 131.0 (q, $J_{C\text{-}F}$ = 33 Hz), 125.6 (q, $J_{C\text{-}F}$ = 3 Hz), 124.0 (q, $J_{C\text{-}F}$ = 171 Hz), 105.5, 93.0, 92.9. **M.p**: 116-118 °C. **HRMS** (EI) m/z for $C_{24}H_{10}ClF_6I$ $[M]^+$: calcd. 573.9420; found, 573.9412.

1.4 General procedure for one-pot double Suzuki-Miyaura cross-coupling reactions from 1,2-diiodo-3-(phenylethynyl)benzene (5_A)

A flame-dried round-bottom flask was charged with 1,2-diiodo-3-(phenylethynyl)benzene (**5_A**) (0.66 mmol, 1.0 equiv.), arylboronic acid (1.65 mmol, 2.5 equiv.), tetrakis(triphenylphosphine)palladium(0) (12 mol-%), toluene (0.1M), potassium carbonate (2 M solution, 1.4 mL), and ethanol (0.4 mL) under argon. The mixture was heated at 100 °C for 12 h and then cooled down to room temperature. 100 mL distilled water was added and aqueous layer was extracted with ethyl acetate (2 x 50 mL). The organic layers were combined, washed with brine, dried with Na_2SO_4, filtered, and then concentrated under reduced pressure. The crude residue was purified by flash chromatography (100% hexane) to yield the desired pure product.

1.4.1 Synthesis of 4,4''-dimethoxy-3'-(phenylethynyl)-1,1':2',1''-terphenyl (36)

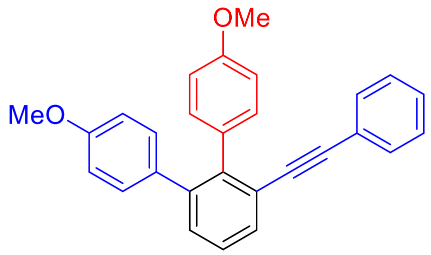

The title compound was synthesized using the general procedure for one-pot double Suzuki-Miyaura cross-coupling reaction and isolated in **55%** yield as white solid after flash chromatography. **IR** (cast film, cm^{-1}) 3102, 3098, 3016, 2208, 1618, 1602, 1589, 1207, 1153, 943, 679. δ_H (400 MHz, $CDCl_3$) δ: 7.60 (d, 1H, *J* = 6.5 Hz), 7.35 (d, 2H, *J* = 6.4 Hz), 7.20-7.28 (bs, 5H), 7.16 (d, 2H, *J* = 8.5 Hz), 7.02 (d, 2H , *J* = 8.5 Hz), 6.80 (d, 2H , *J* = 8.5 Hz), 6.73 (d, 2H , *J* = 8.5 Hz), 3.80 (s, 3H), 3.79 (s, 3H). δ_C (100 MHz, $CDCl_3$) δ: 158.5, 158.3, 142.5, 141.4, 133.9, 132.2, 132.1, 131.5, 131.2, 131.0, 130.6, 128.3,

128.1, 127.2, 123.9, 123.7, 113.4, 113.0, 92.5, 90.0, 55.3, 55.3. **M.p**: 119-121 °C. **HRMS** (EI) m/z for $C_{28}H_{22}O_2$ $[M]^+$: calcd. 390.1620; found, 390.1615.

1.4.2 Synthesis of 3'-(phenylethynyl)-4,4''-bis(trifluoromethyl)-1,1':2',1''-terphenyl (37)

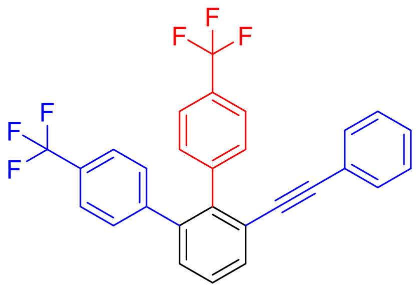

The title compound was synthesized using the general procedure for one-pot double Suzuki-Miyaura cross-coupling reactions and isolated in **66%** yield as white solid after flash chromatography. **IR** (cast film, cm^{-1}) 3124, 3082, 3016, 2198, 1608, 1589, 1562, 867, 721, 638. δ_H (400 MHz, $CDCl_3$) δ: 7.69-7.70 (m, 1H), 7.54 (d, 2H, J = 8.0 Hz), 7.33-7.47 (m, 6H), 7.19-7.26 (m, 5H), 7.1-7.12 (m, 2H). δ_C (100 MHz, $CDCl_3$) δ: 144.4, 143.1, 141.5, 140.2, 132.1, 131.4, 130.4, 130.2, 129.5 (q, J_{C-F} = 33 Hz), 129.2 (q, J_{C-F} = 32 Hz), 128.6, 128.5, 128.3, 125.1 (q, J_{C-F} = 4 Hz), 124.7 (q, J_{C-F} = 3 Hz), 124.1, 123.0 (q, J_{C-F} = 3 Hz) , 122.9, 120.3, 120.2, 93.9, 88.7. **M.p**: 117-119 °C. **HRMS** (EI) m/z for $C_{28}H_{16}F_6$ $[M]^+$: calcd. 466.1156; found, 466.1143.

1.5 NMR for New Compounds

1.5.1 ^{1}H-NMR of 1,2-diiodo-3-(phenylethynyl)benzene (5_A) in *$CDCl_3$* at 25 ºC.

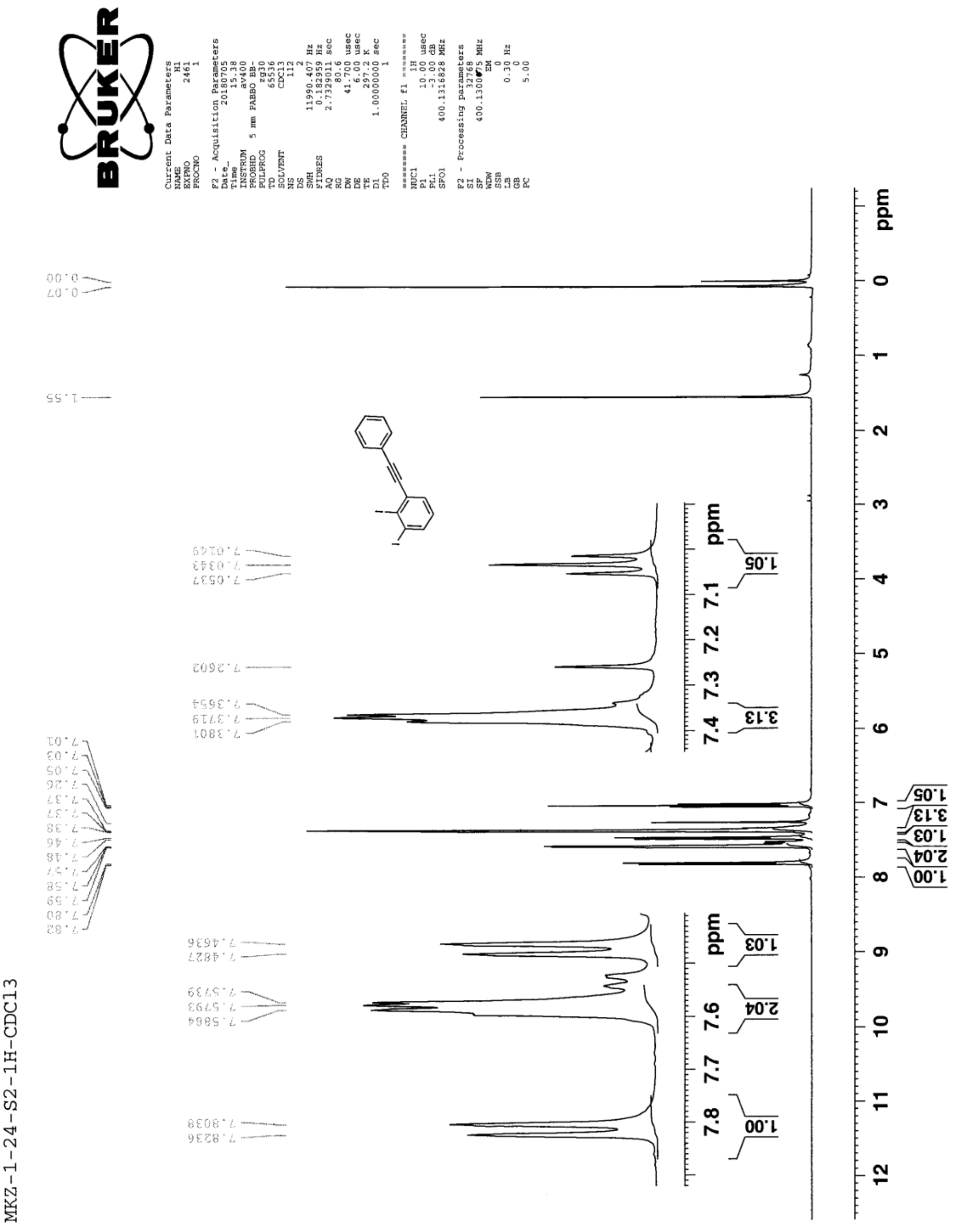

1.5.2 ^{13}C-NMR of 1,2-diiodo-3-(phenylethynyl)benzene (5_A) in *$CDCl_3$* at 25 ^oC.

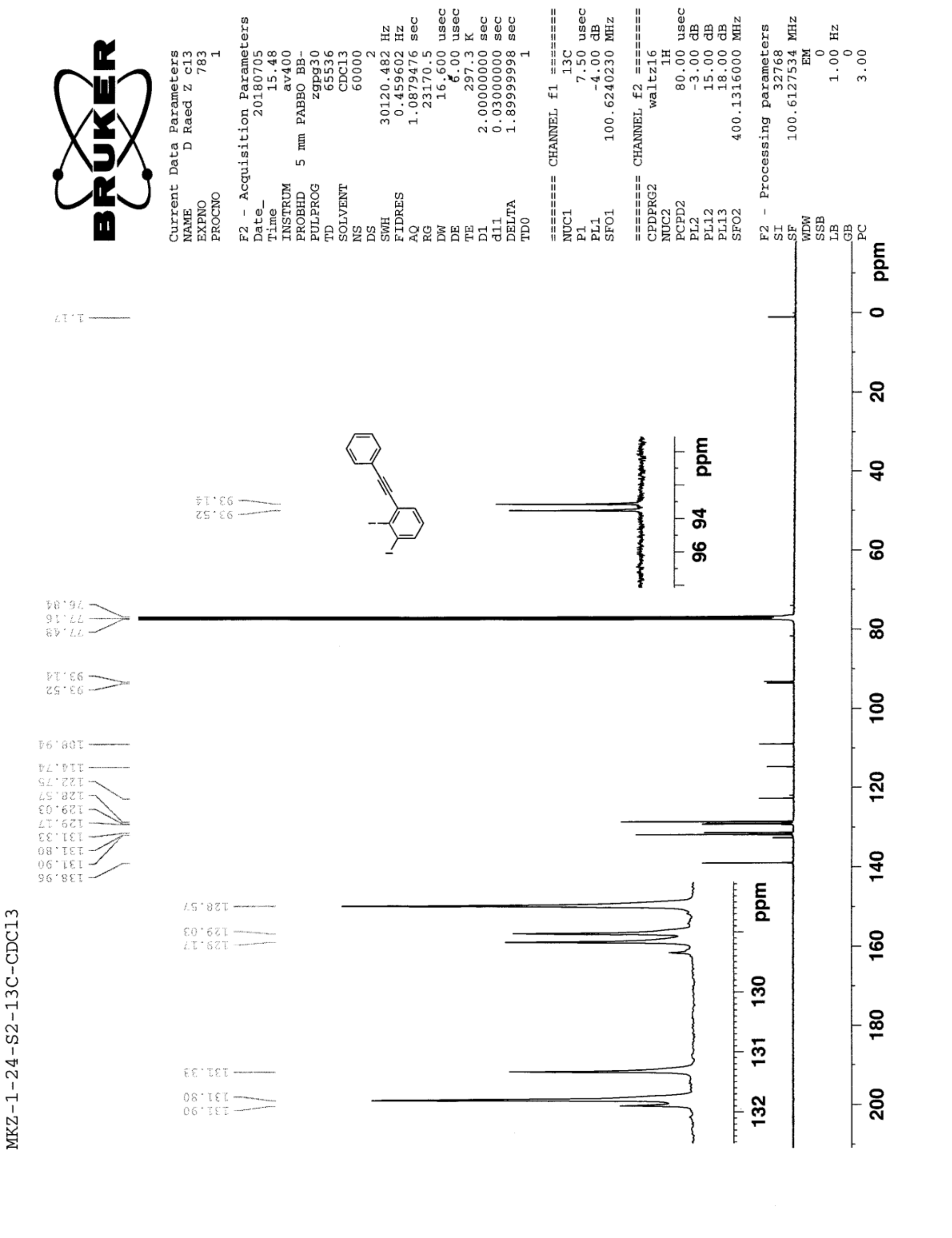

1.5.3 ^{1}H-NMR of 1,2-diiodo-3-((4-(trifluoromethyl)phenyl)ethynyl) benzene (6_A) in *$CDCl_3$* at 25 °C.

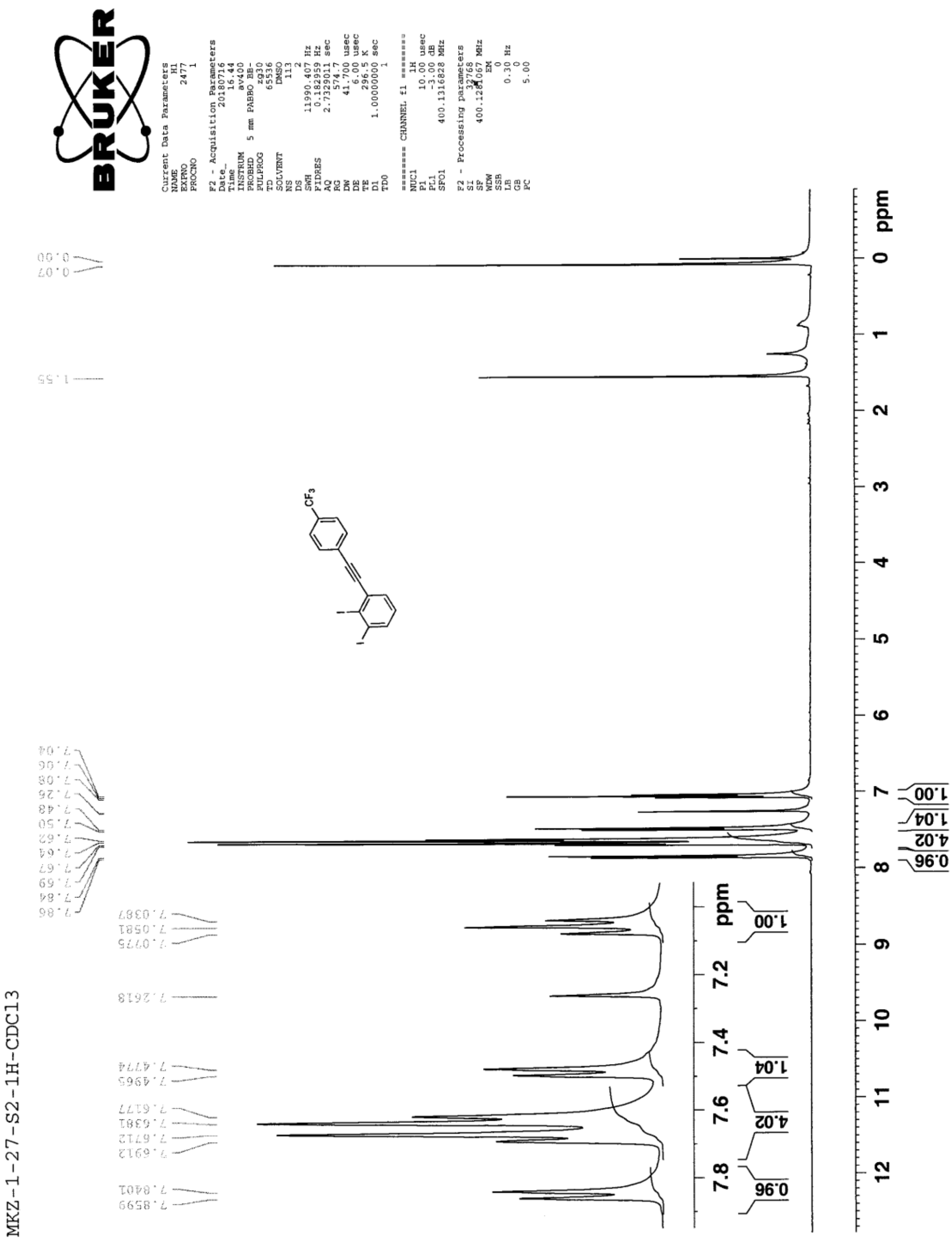

1.5.4 ^{13}C-NMR of 1,2-diiodo-3-((4-(trifluoromethyl)phenyl)ethynyl) benzene (6A) in *CDCl*3 at 25 ^{o}C.

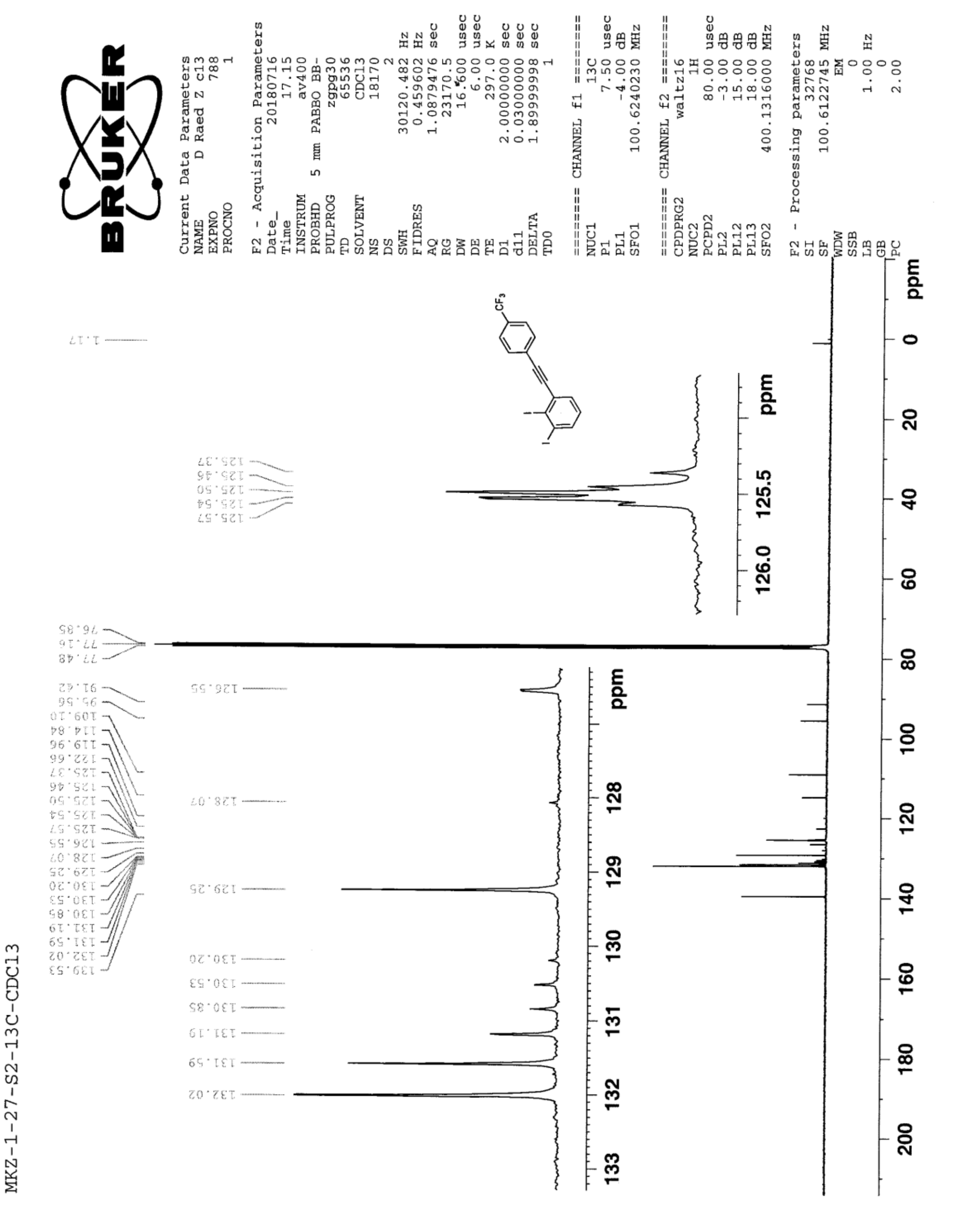

1.5.5 ^{1}H-NMR of 1-((4-chlorophenyl)ethynyl)-2,3-diiodobenzene (7_A) in *$CDCl_3$* at 25 °C.

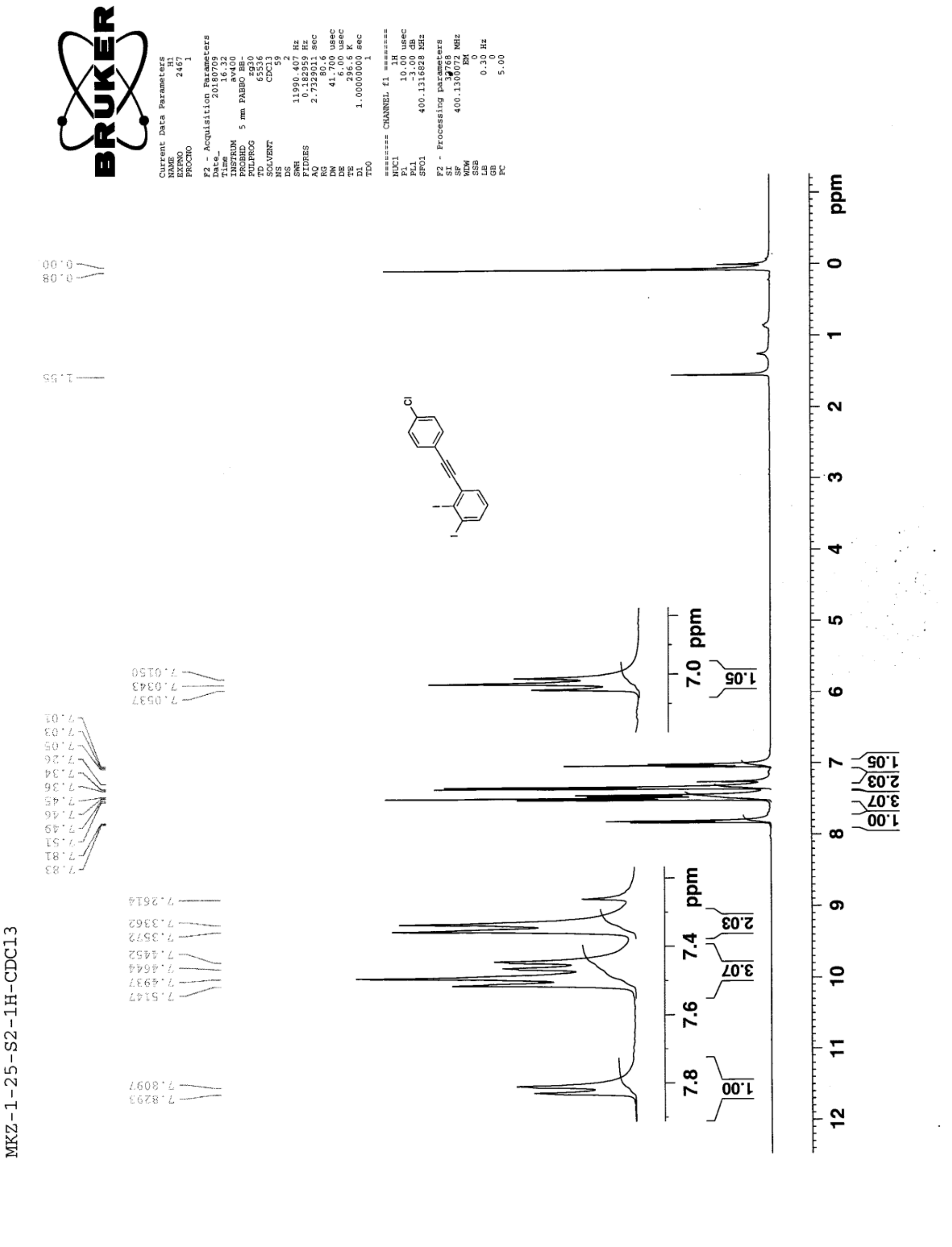

1.5.6 ^{13}C-NMR of 1-((4-chlorophenyl)ethynyl)-2,3-diiodobenzene (7_A) in *CDCl*$_3$ at 25 ^{o}C.

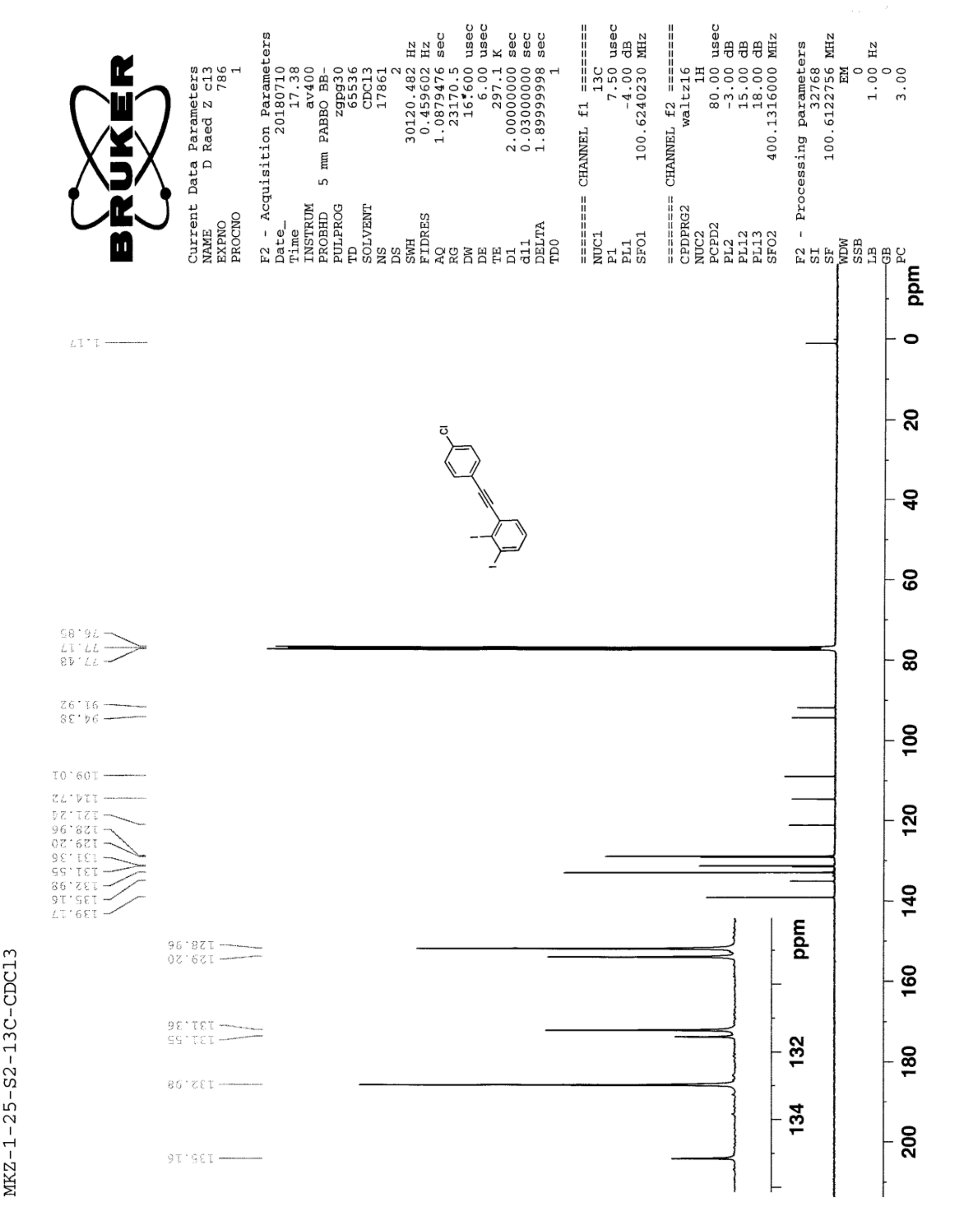

1.5.7 ^{1}H-NMR of 1,2-diiodo-3-((4-methoxyphenyl)ethynyl)benzene (8_A) in *$CDCl_3$* at 25 °C.

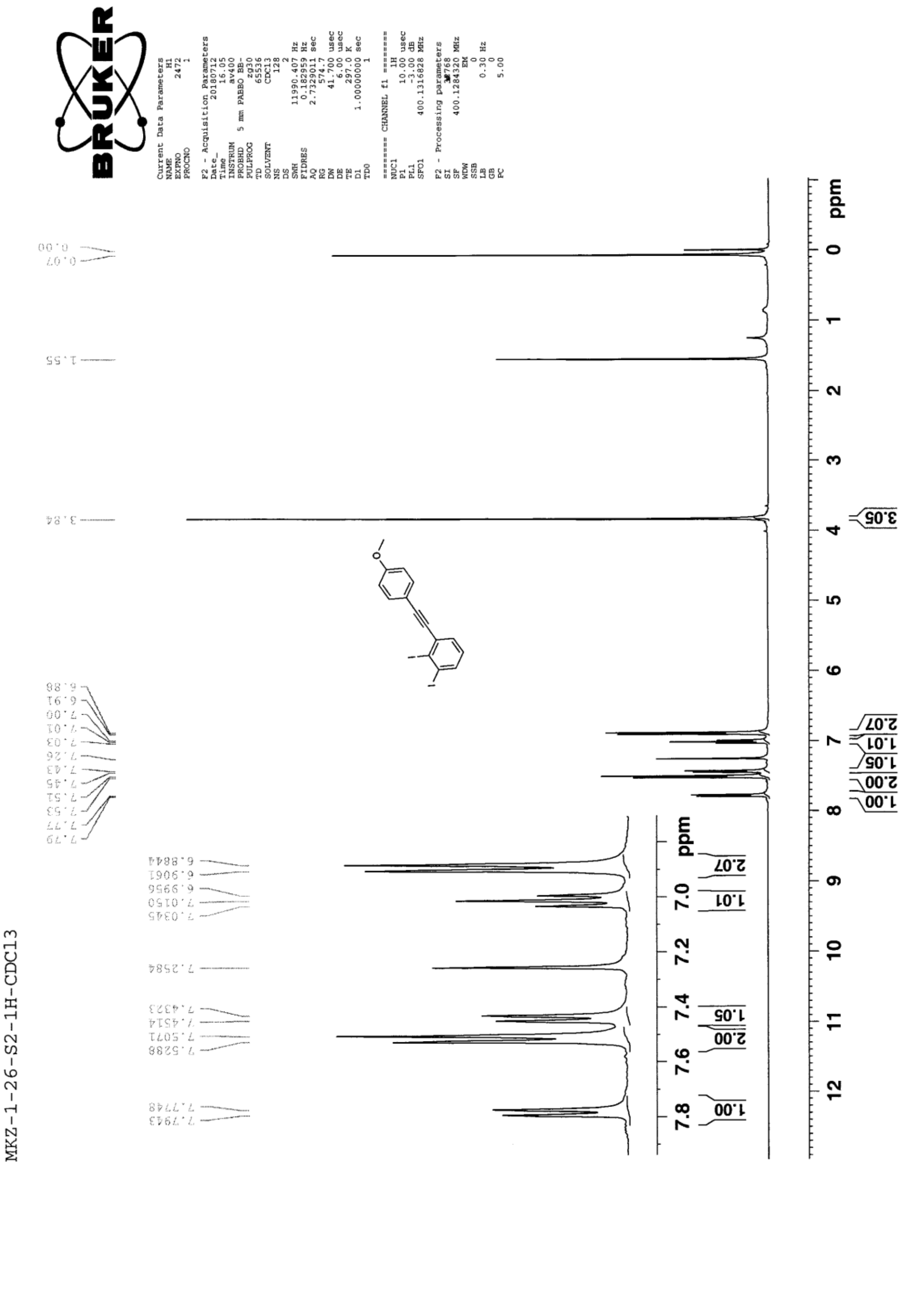

1.5.8 ^{13}C-NMR of 1,2-diiodo-3-((4-methoxyphenyl)ethynyl)benzene (8_A) in $CDCl_3$ at 25 °C.

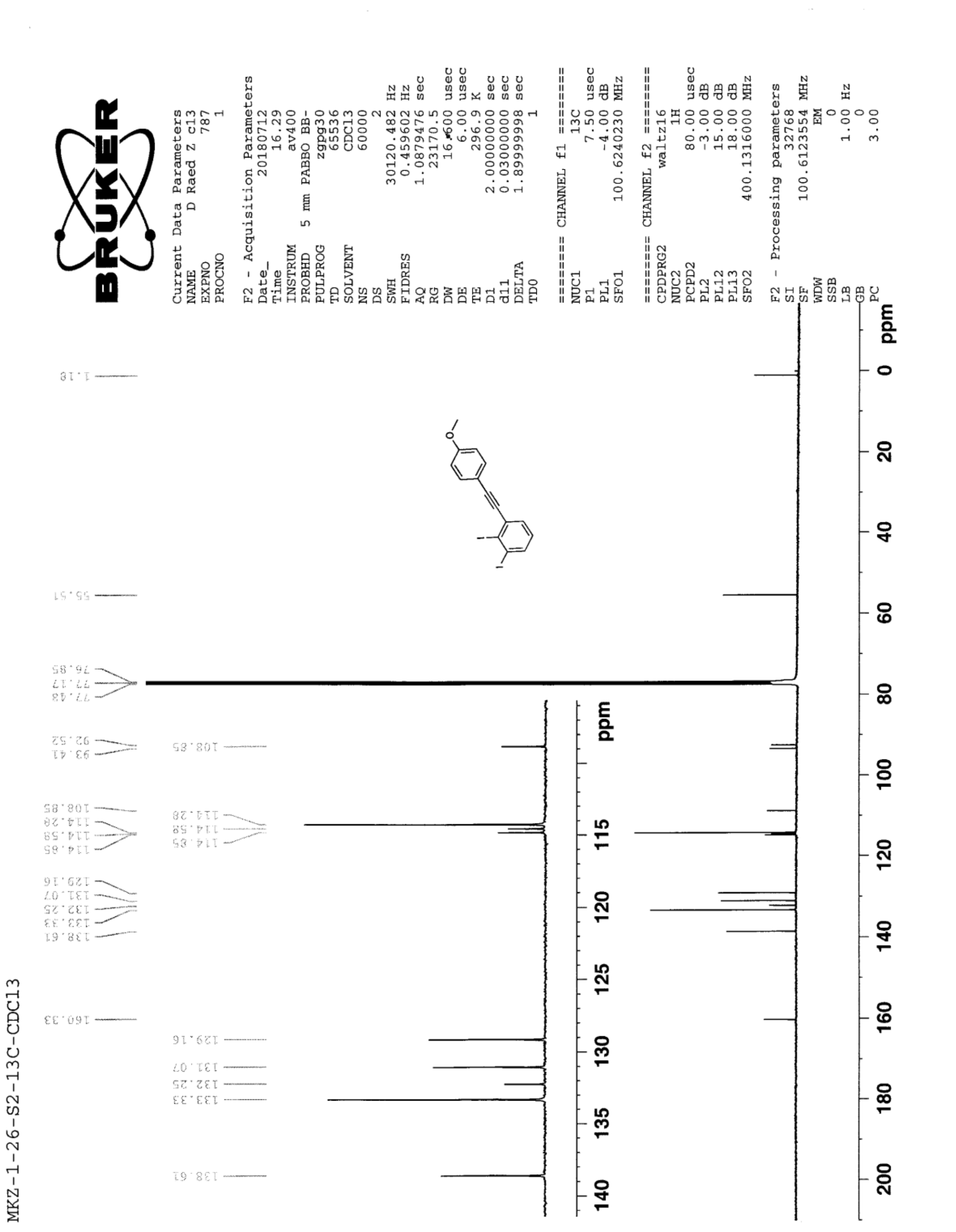

1.5.9 ^{1}H-NMR of 1,2-diiodo-5-methyl-3-(phenylethynyl)benzene (9_A) in *$CDCl_3$* at 25 °C.

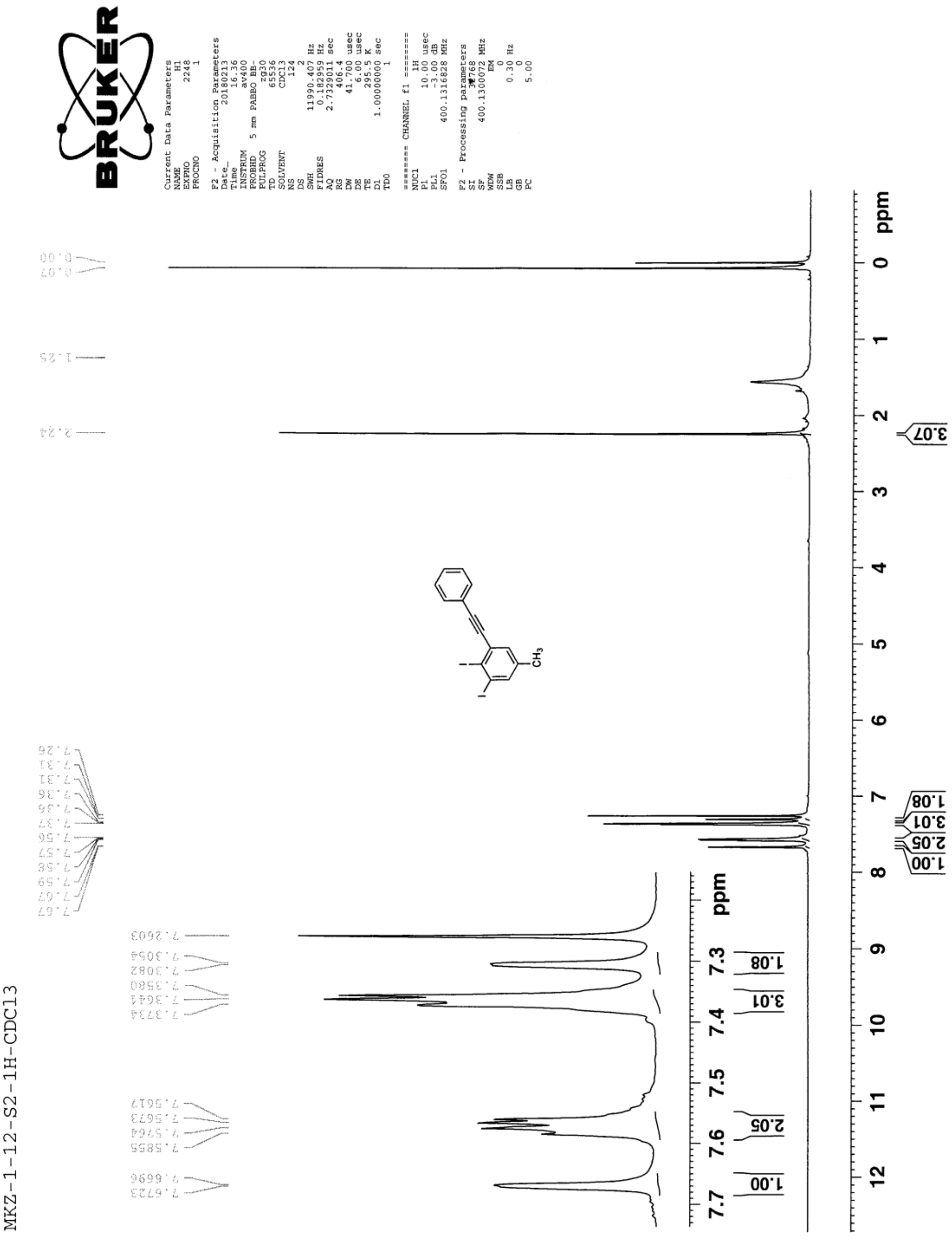

1.5.10 ^{13}C-NMR of 1,2-diiodo-5-methyl-3-(phenylethynyl)benzene (9_A) in *$CDCl_3$* at 25 °C.

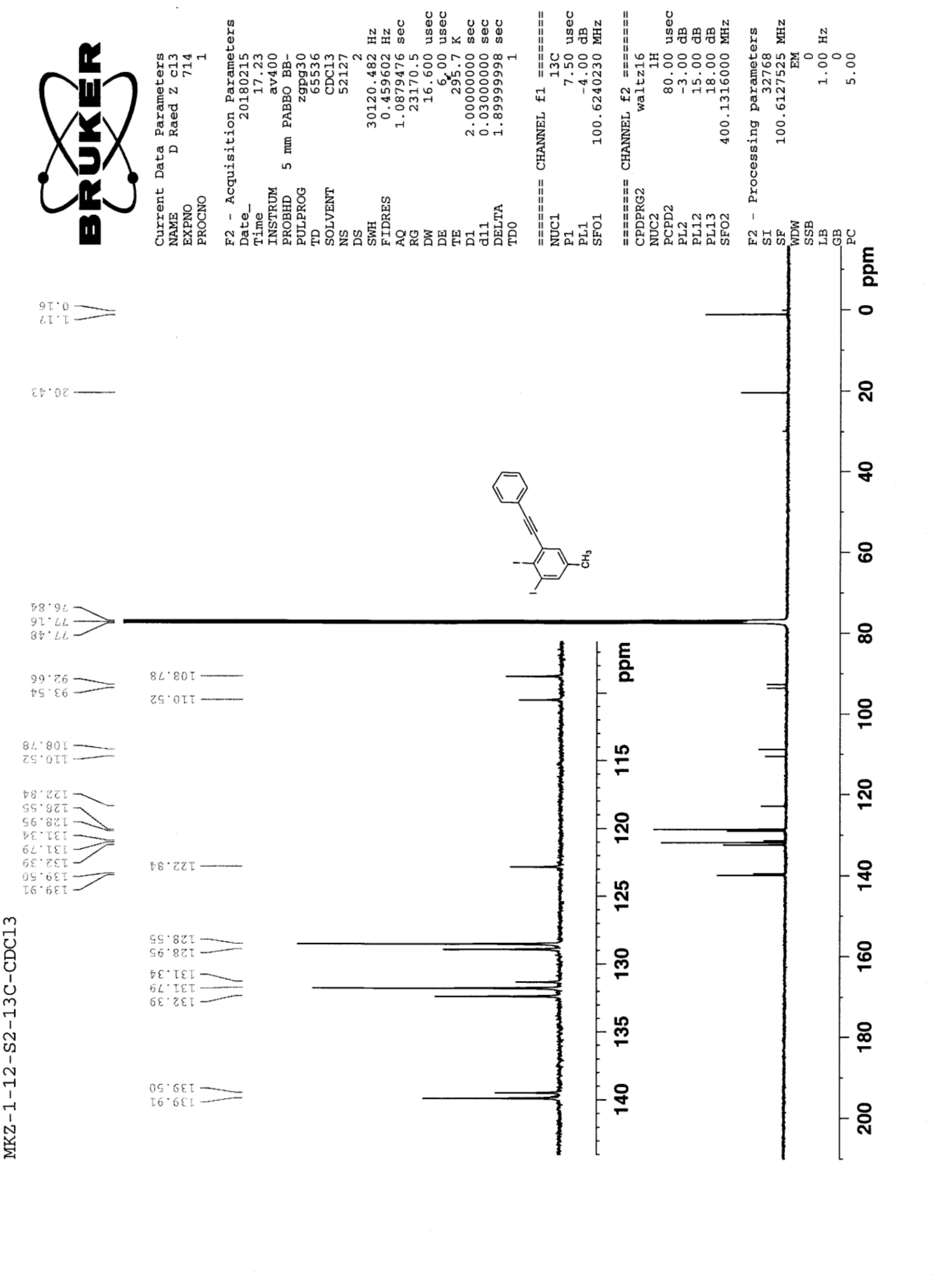

1.5.11 ¹H-NMR of 1-((4-ethylphenyl)ethynyl)-2,3-diiodo-5-methylbenzene (10_A) in *$CDCl_3$* at 25 °C

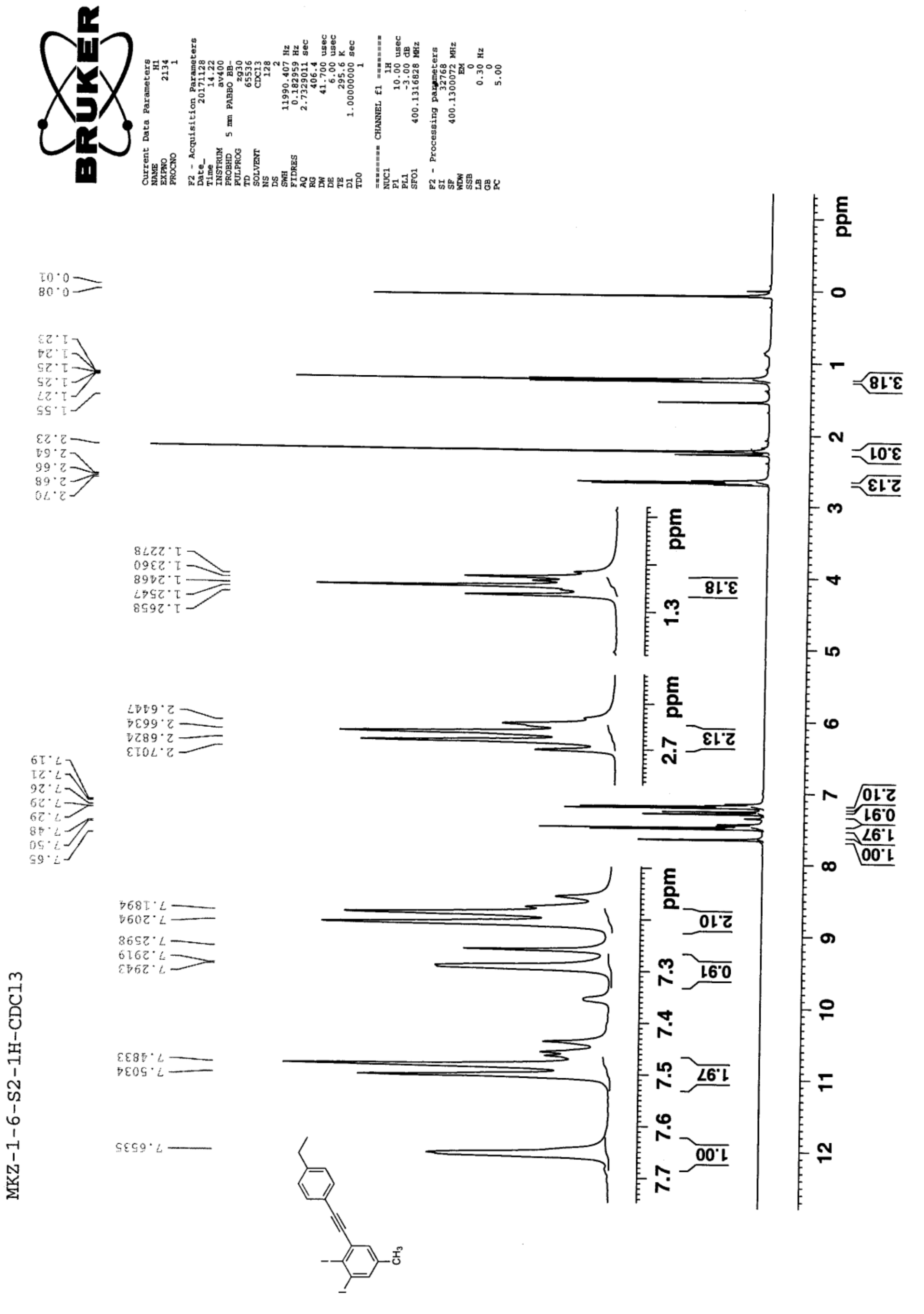

1.5.12 ^{13}C-NMR of 1-((4-ethylphenyl)ethynyl)-2,3-diiodo-5-methylbenzene (10A) in *CDCl3* at 25 °C

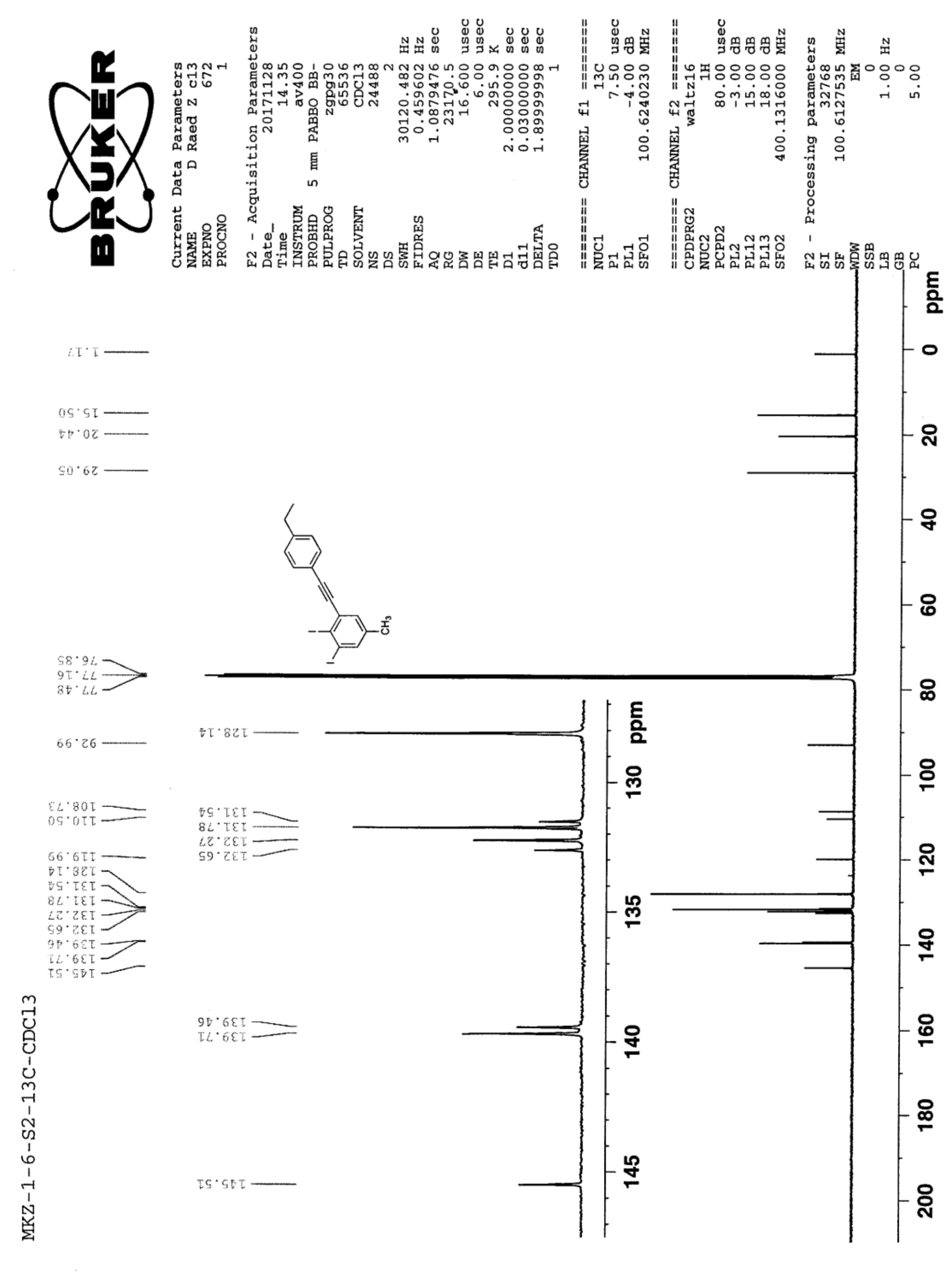

1.5.13 ¹H-NMR of 1,2-diiodo-3-((4-methoxyphenyl)ethynyl)-5-methyl benzene (11_A) in *CDCl₃* at 25 °C.

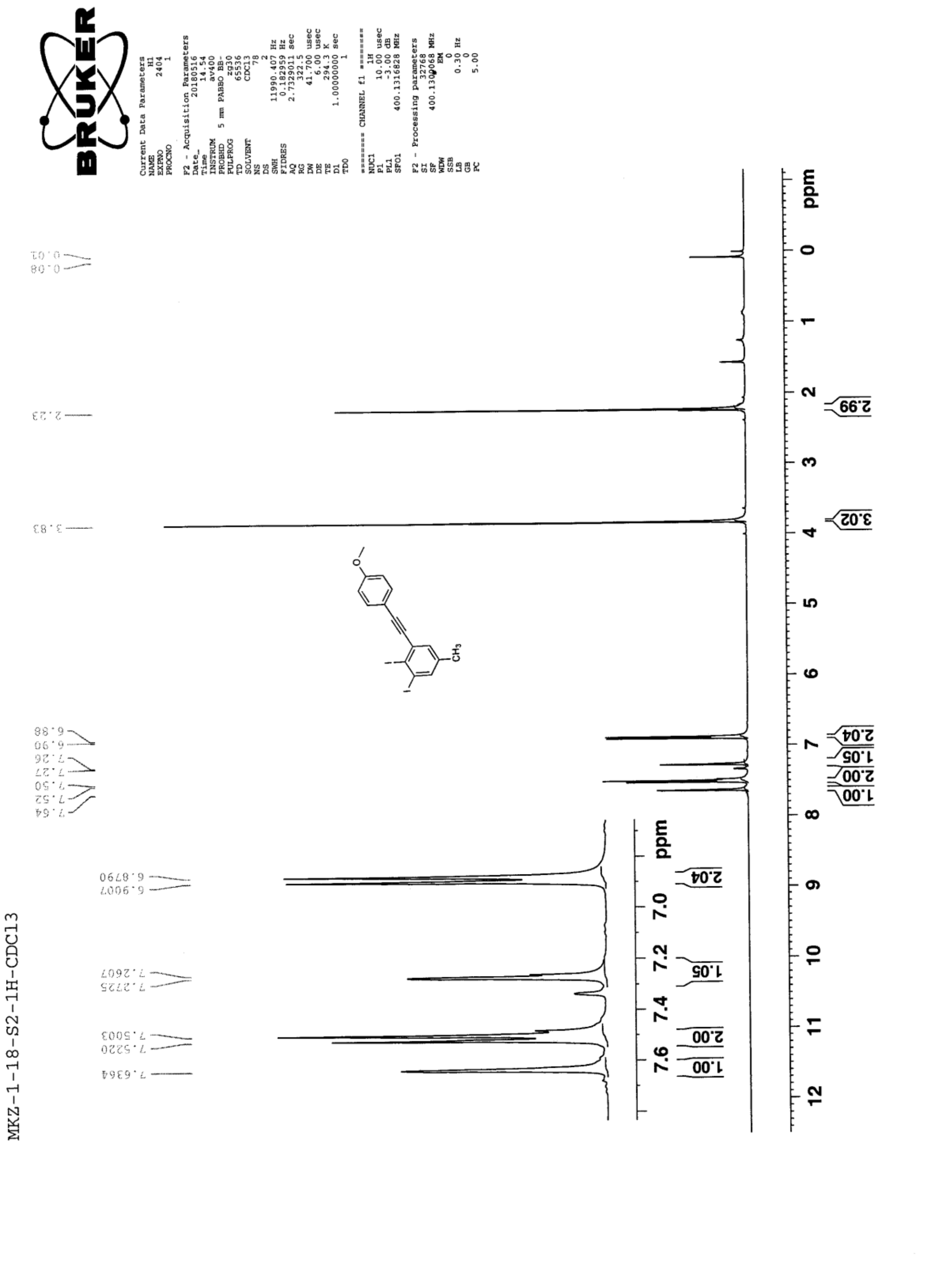

1.5.14 ^{13}C-NMR of 1,2-diiodo-3-((4-methoxyphenyl)ethynyl)-5-methyl benzene (11_A) in *$CDCl_3$* at 25 ^oC.

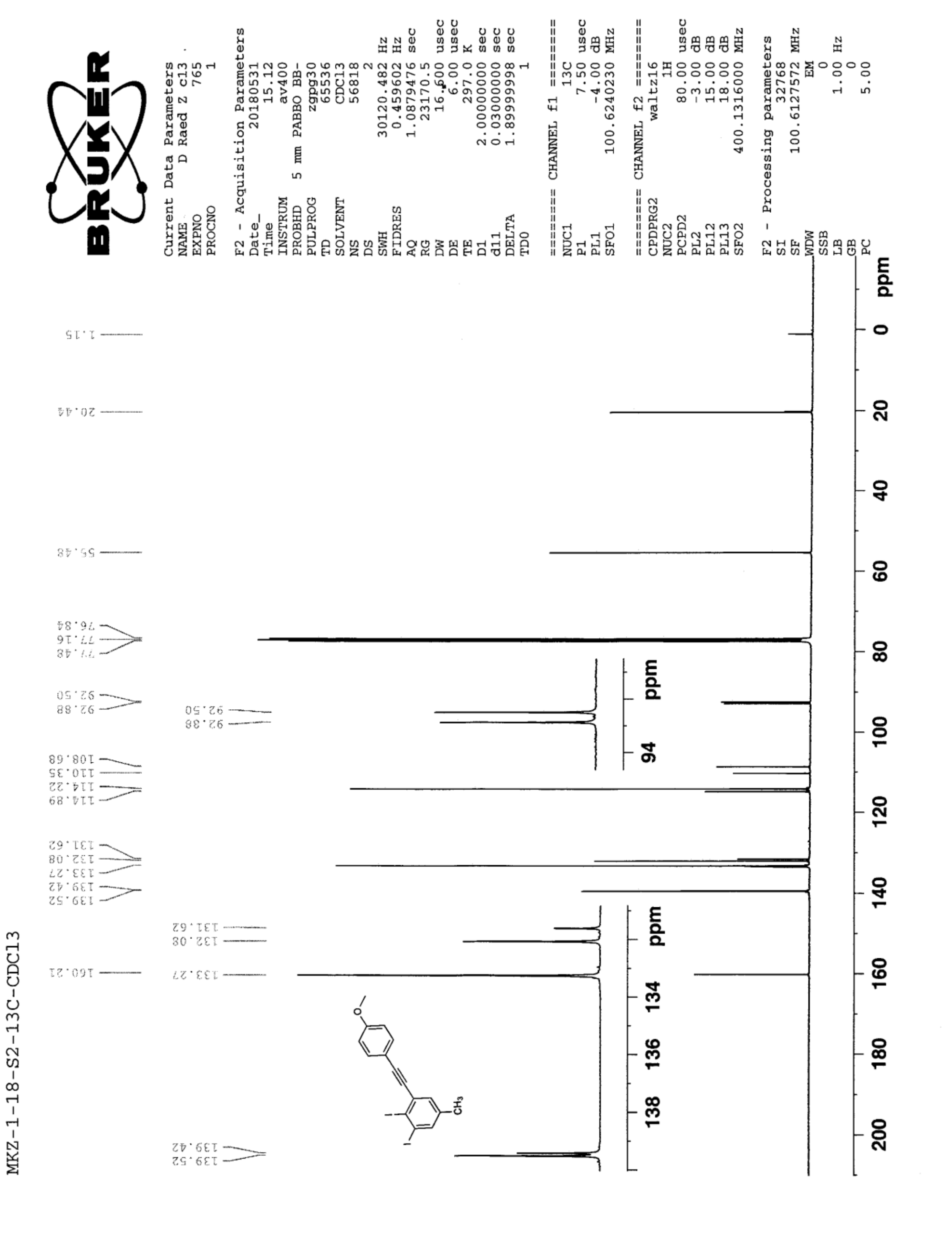

R. M. Al-Zoubi, M. K. Al-Omari, W. K. Al-Jammal and M. J. Ferguson

1.5.15 ^{1}H-NMR of 1,2-diiodo-5-methoxy-3-((4-methoxyphenyl) ethynyl) benzene (12_A) in *CDCl*$_3$ at 25 ^{o}C.

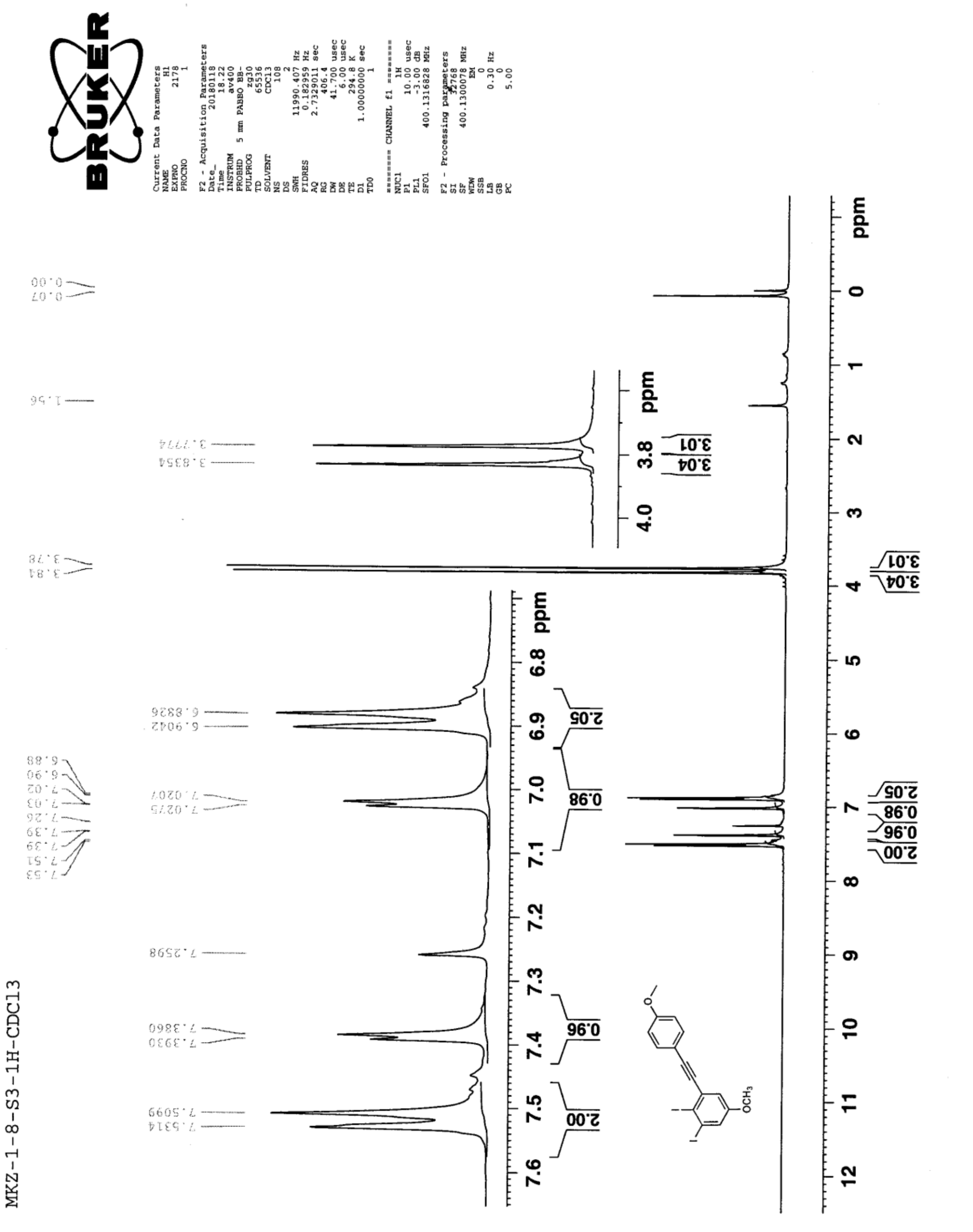

1.5.16 ^{13}C-NMR of 1,2-diiodo-5-methoxy-3-((4-methoxyphenyl) ethynyl) benzene (12_A) in *$CDCl_3$* at 25 °C.

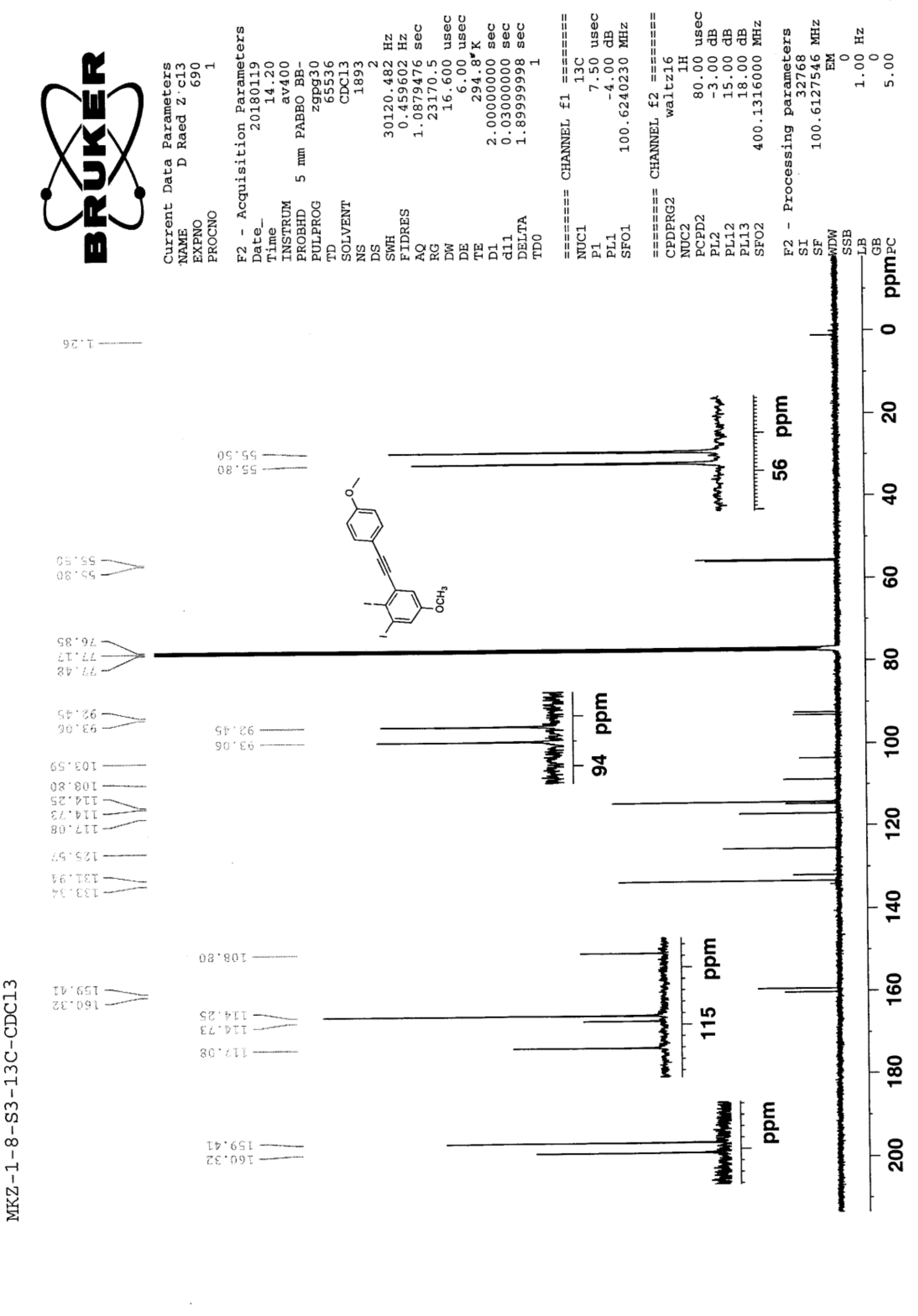

R. M. Al-Zoubi, M. K. Al-Omari, W. K. Al-Jammal and M. J. Ferguson

1.5.17 ^{1}H-NMR of 1-((4-ethylphenyl)ethynyl)-2,3-diiodo-5-methoxy benzene (13$_A$) in *CDCl*$_3$ at 25 °C.

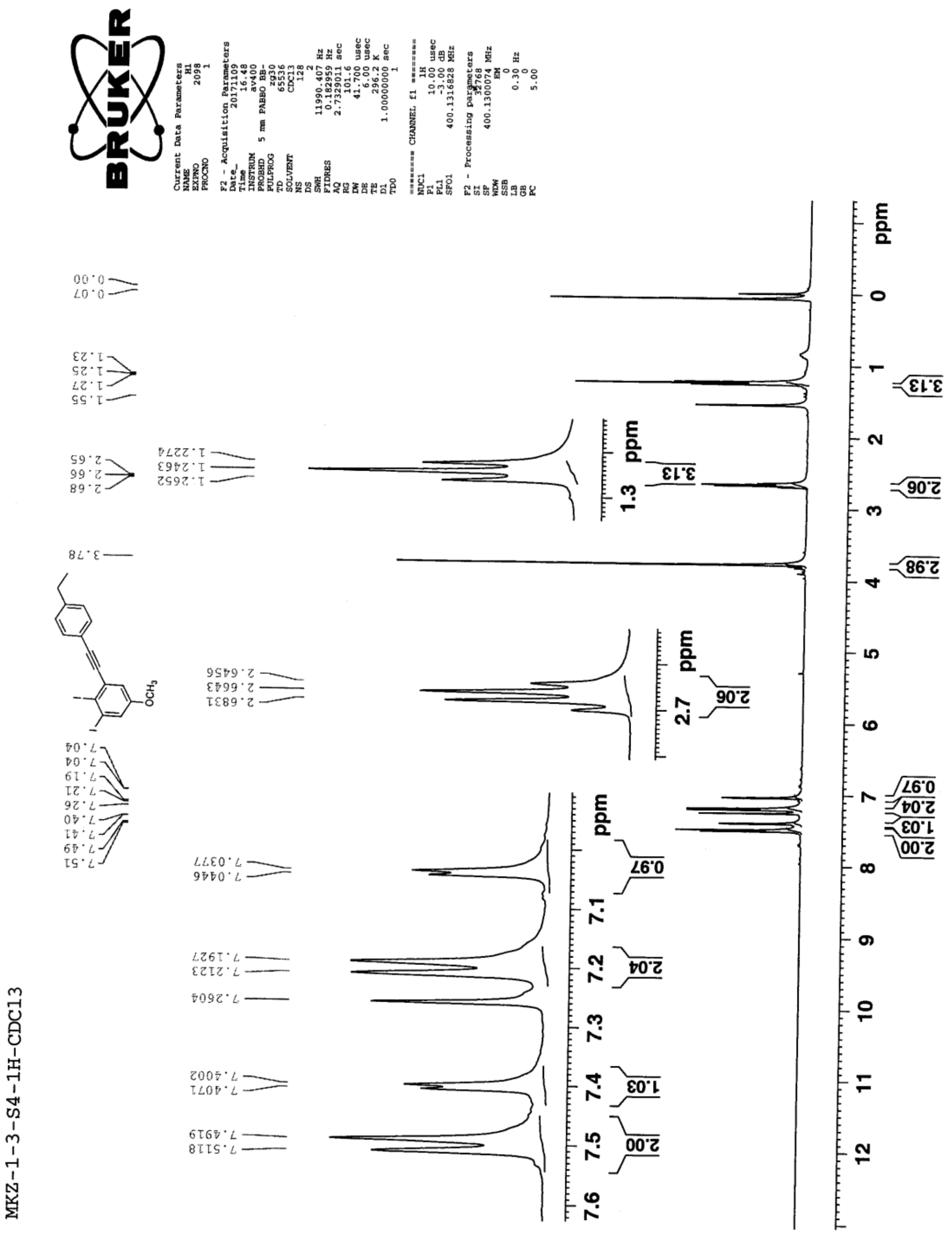

1.5.18 ^{13}C-NMR of 1-((4-ethylphenyl)ethynyl)-2,3-diiodo-5-methoxy benzene (13_A) in *$CDCl_3$* at 25 °C.

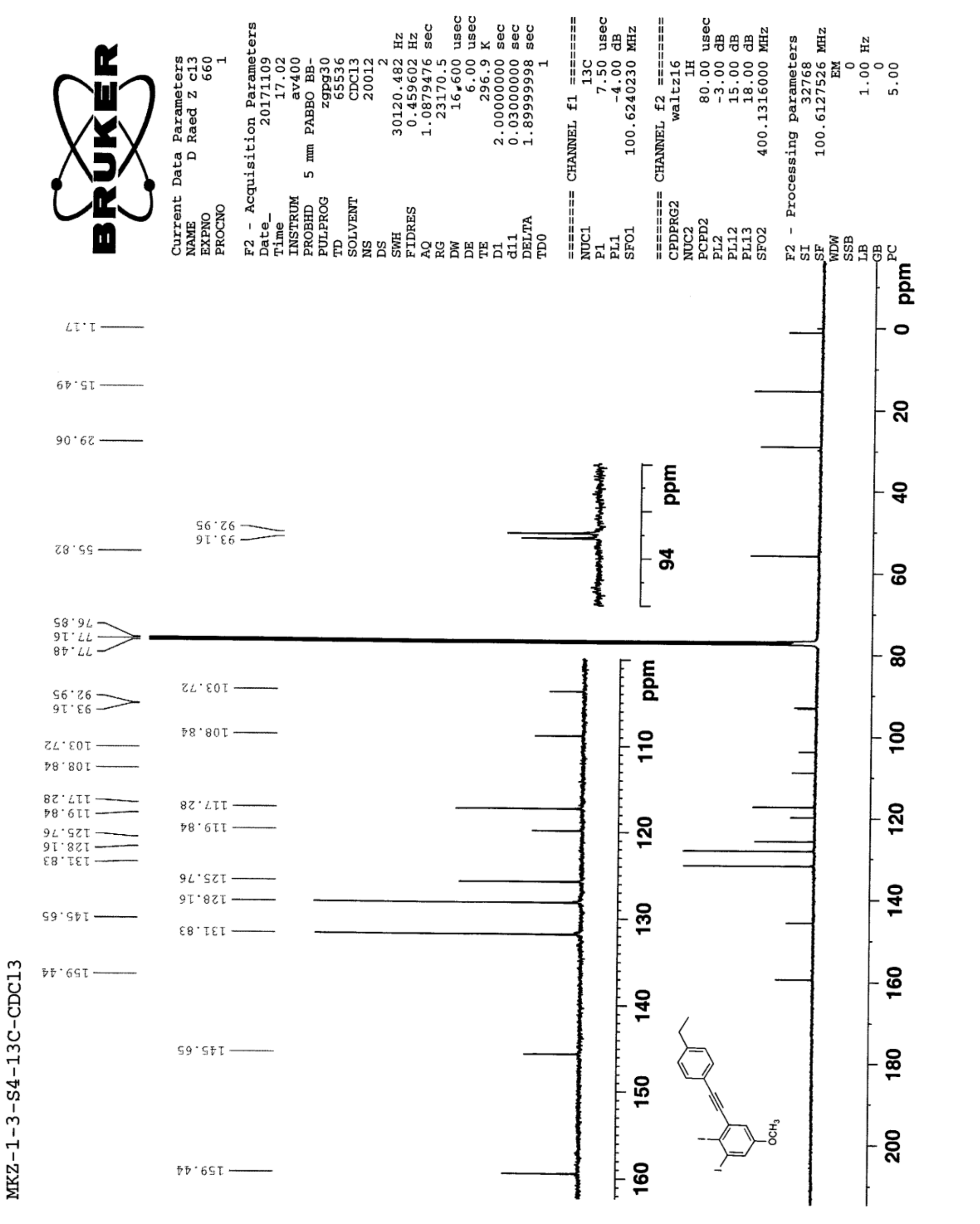

R. M. Al-Zoubi, M. K. Al-Omari, W. K. Al-Jammal and M. J. Ferguson

1.5.19 ^{1}H-NMR of 1,2-diiodo-5-methoxy-3-((4-(trifluoromethyl) phenyl) ethynyl)benzene (14_A) in *$CDCl_3$* at 25 °C.

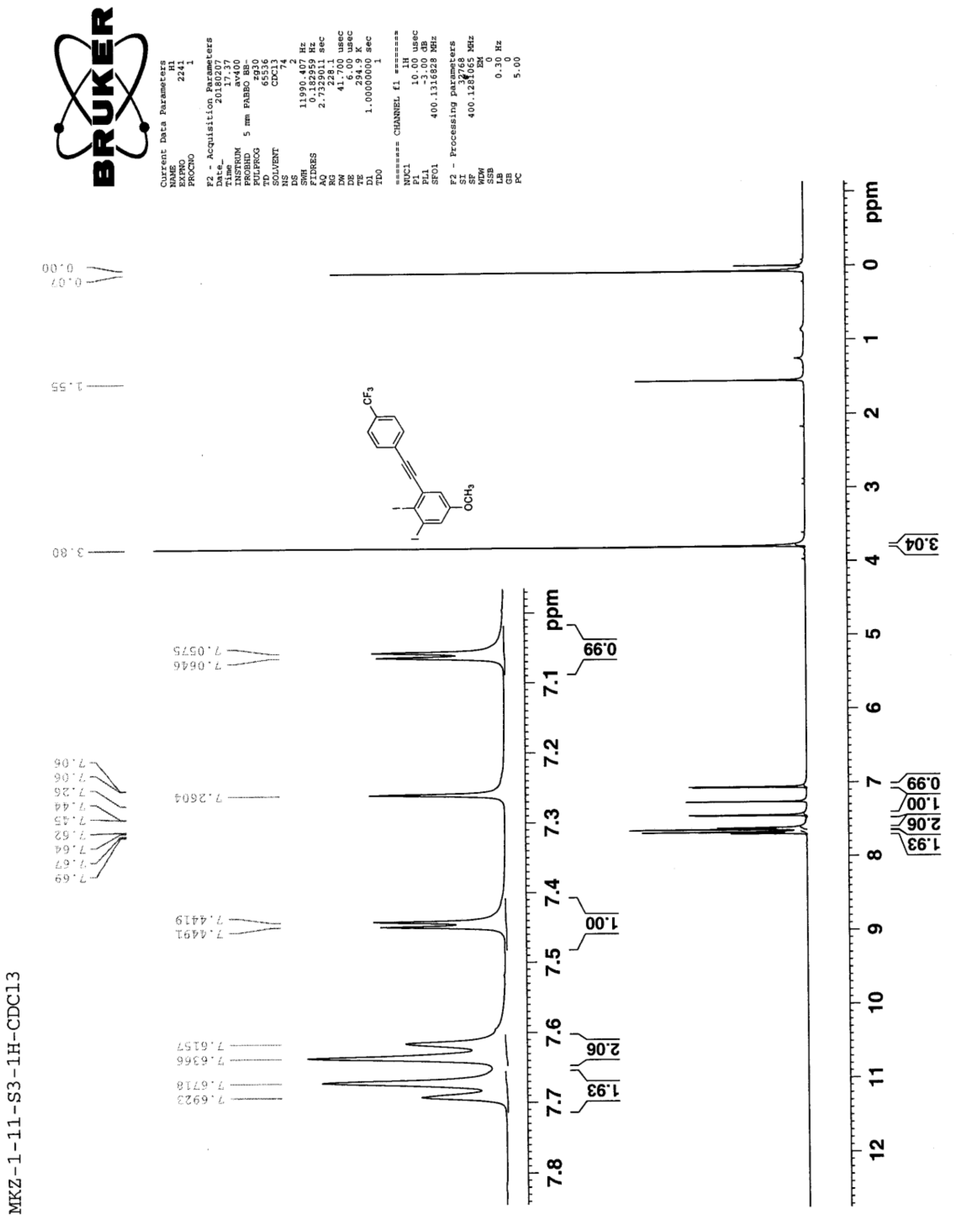

1.5.20 ^{13}C-NMR of 1,2-diiodo-5-methoxy-3-((4-(trifluoromethyl) phenyl) ethynyl)benzene (14$_A$) in *CDCl$_3$* at 25 ^{o}C.

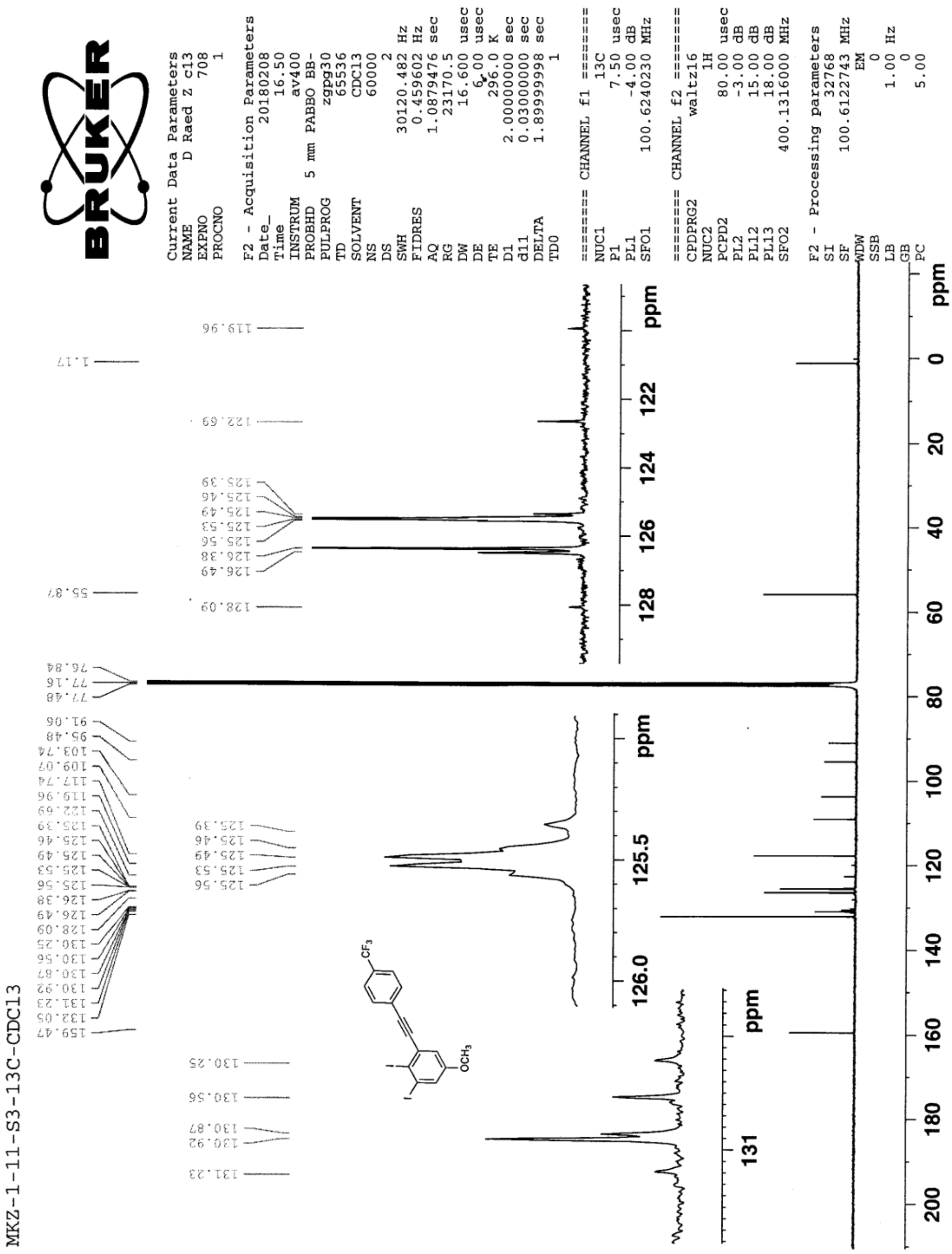

1.5.21 ^{1}H-NMR of 1-((4-fluorophenyl)ethynyl)-2,3-diiodo-5-methoxy benzene (15$_A$) in *$CDCl_3$* at 25 °C.

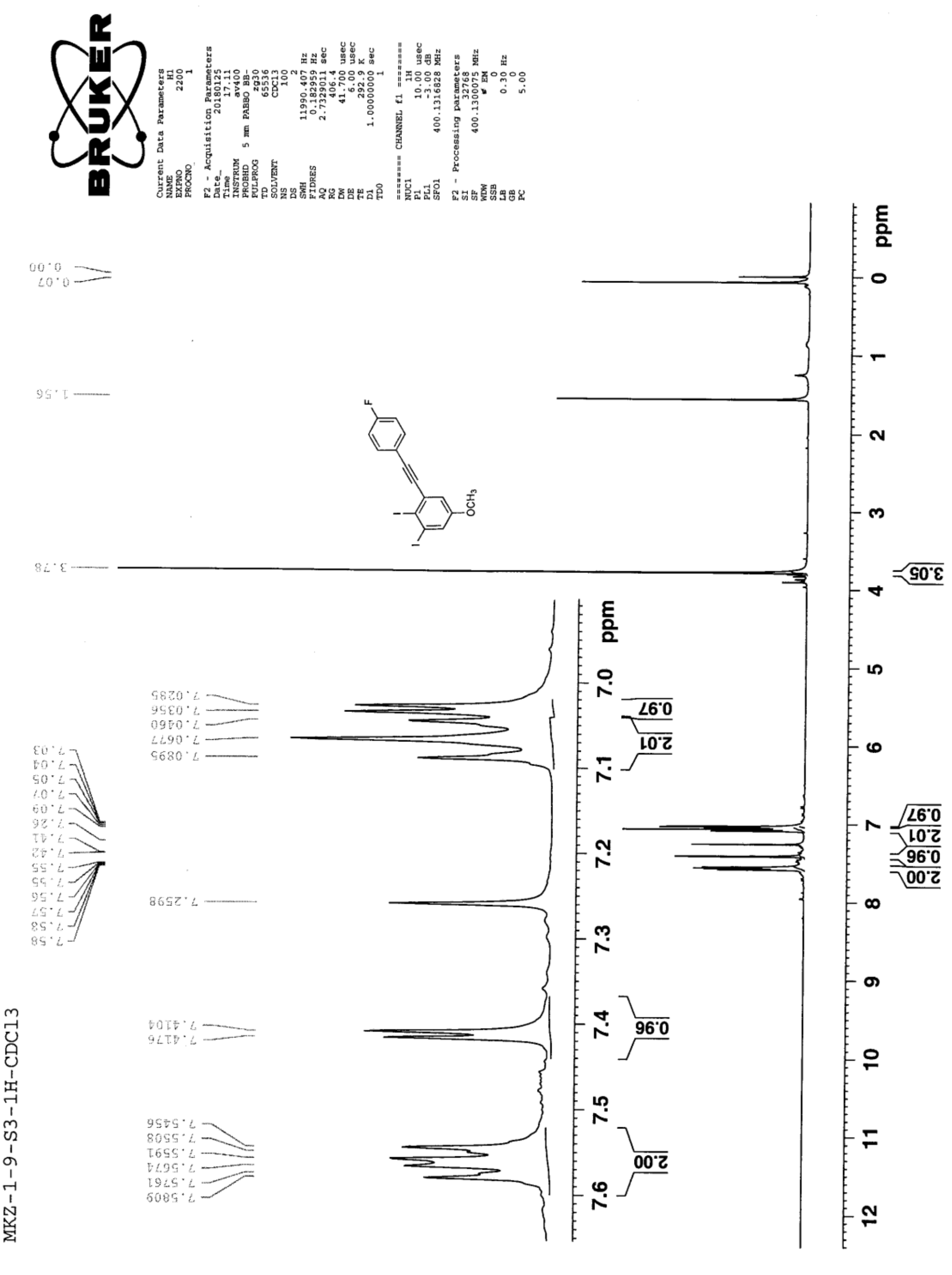

1.5.22 ^{13}C-NMR of 1-((4-fluorophenyl)ethynyl)-2,3-diiodo-5-methoxy benzene (15$_A$) in *CDCl$_3$* at 25 ^{o}C.

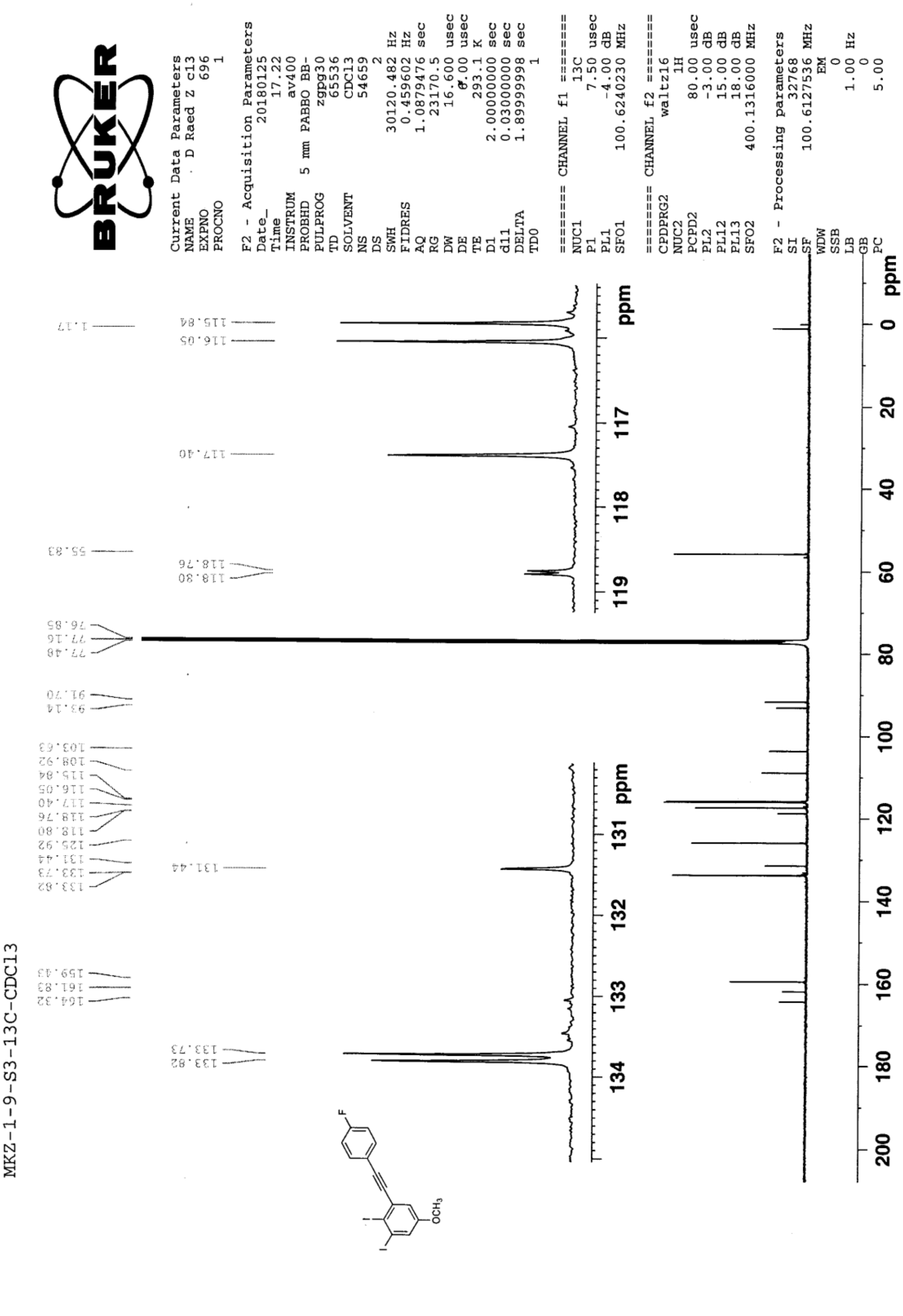

R. M. Al-Zoubi, M. K. Al-Omari, W. K. Al-Jammal and M. J. Ferguson

1.5.23 ^{1}H-NMR of 4-((2,3-diiodo-5-methoxyphenyl)ethynyl)-1,1'-biphenyl (16A) in *CDCl*3 at 25 °C.

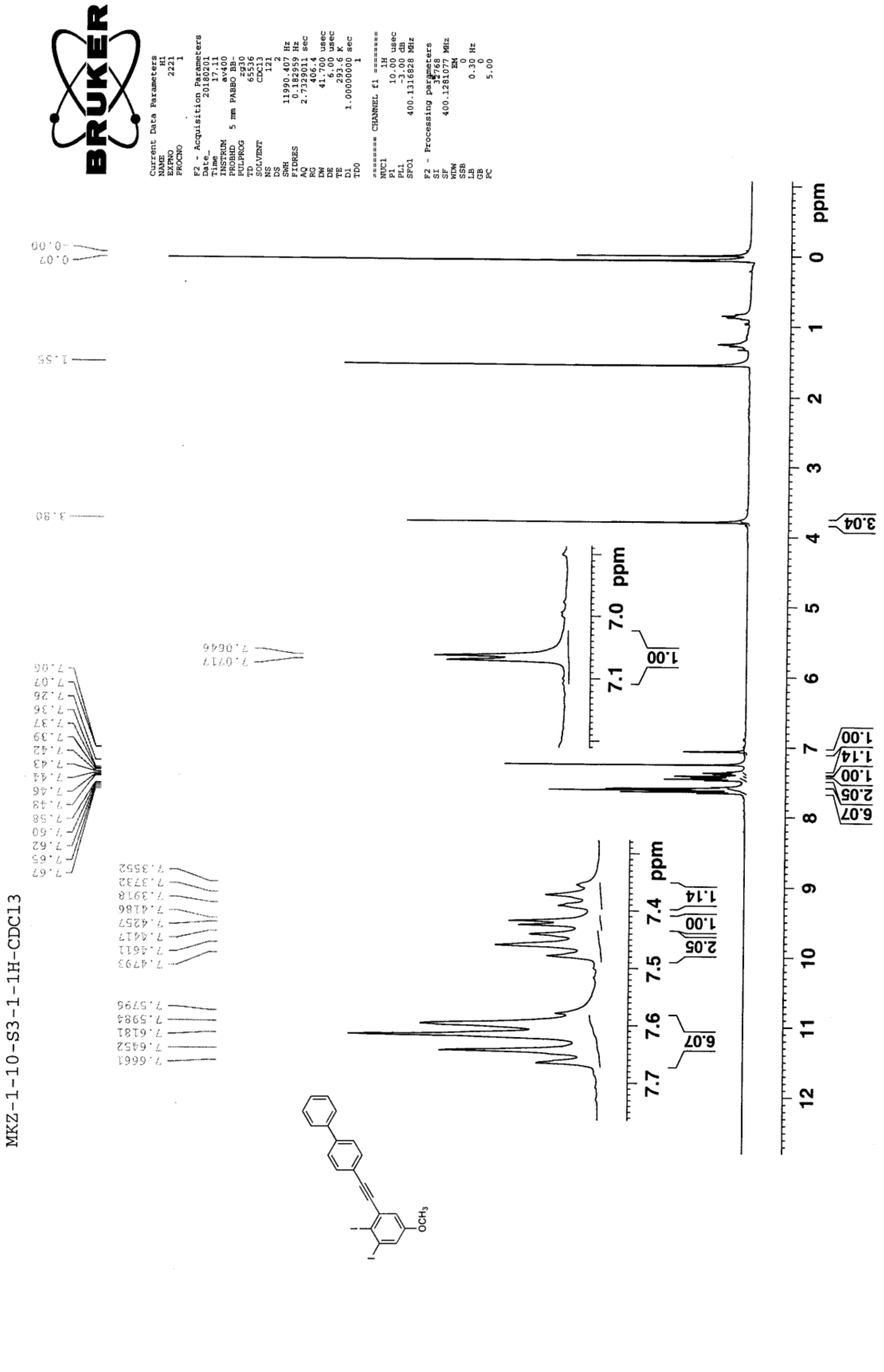

1.5.24 ^{13}C-NMR of 4-((2,3-diiodo-5-methoxyphenyl)ethynyl)-1,1'-biphenyl (16$_A$) in *CDCl$_3$* at 25 ^{o}C.

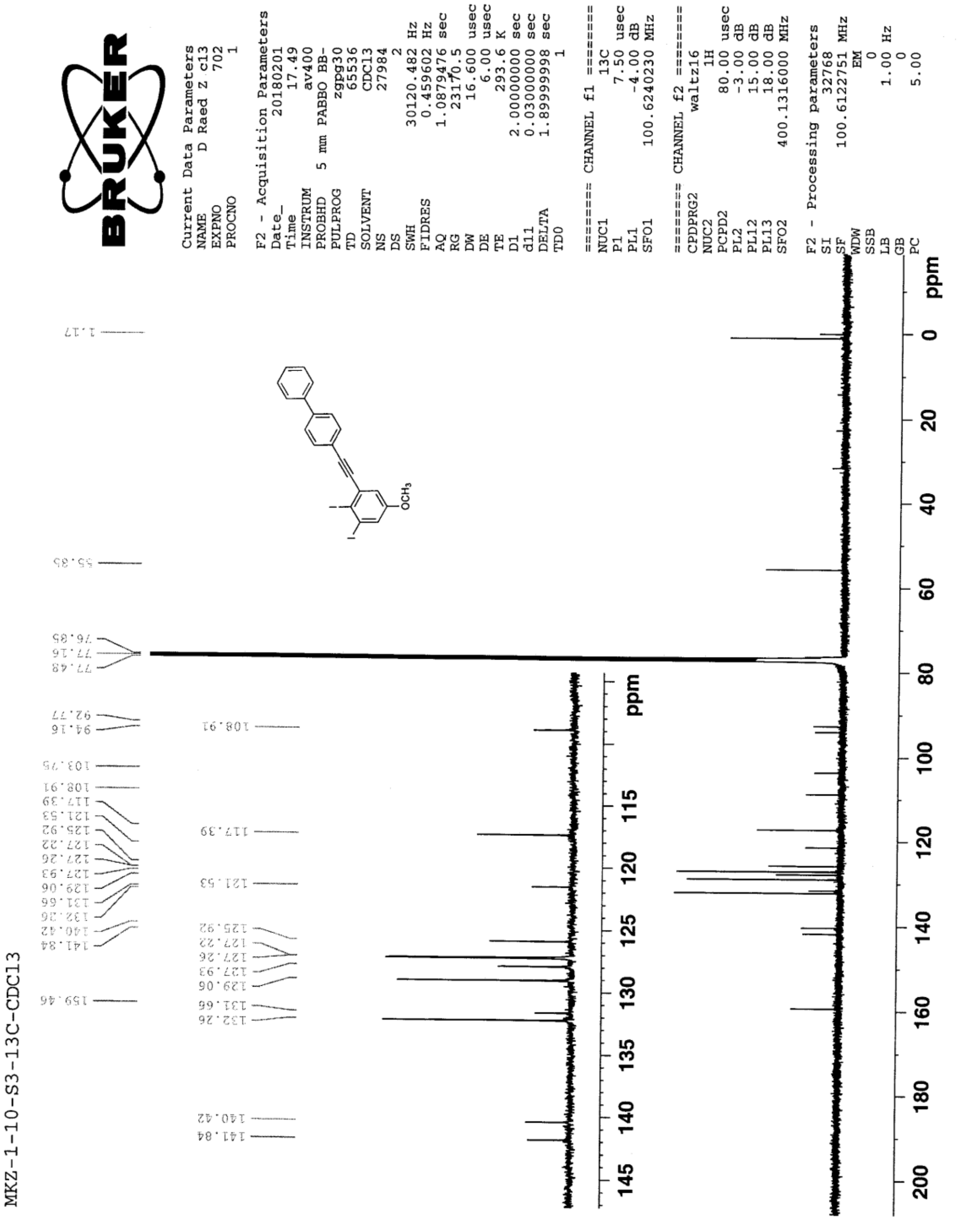

1.5.25 ^{1}H-NMR of 5-chloro-1,2-diiodo-3-(phenylethynyl)benzene (17_A) in *$CDCl_3$* at 25 °C.

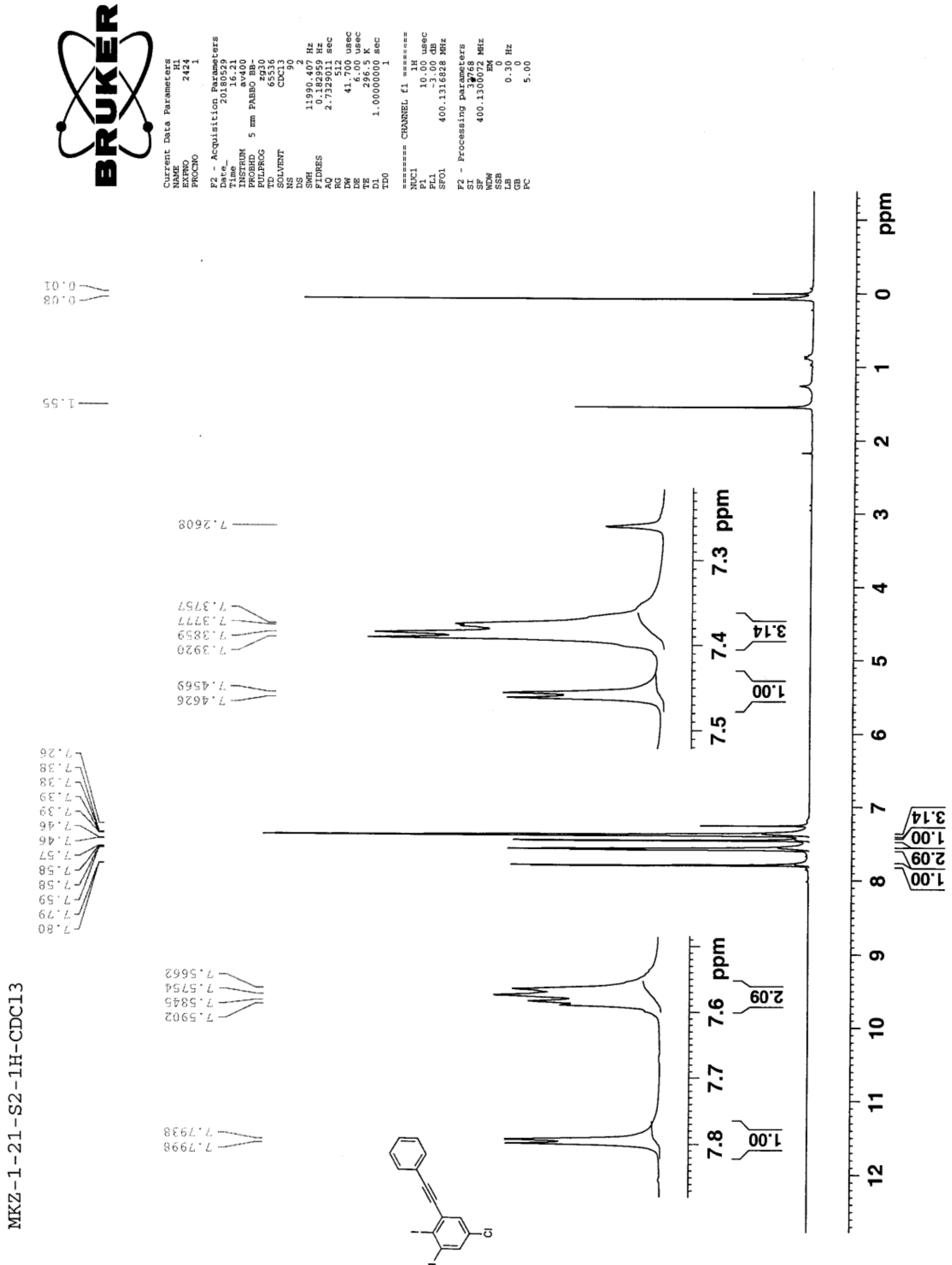

1.5.26 ^{13}C-NMR of 5-chloro-1,2-diiodo-3-(phenylethynyl)benzene (17$_A$) in *CDCl$_3$* at 25 ^{o}C.

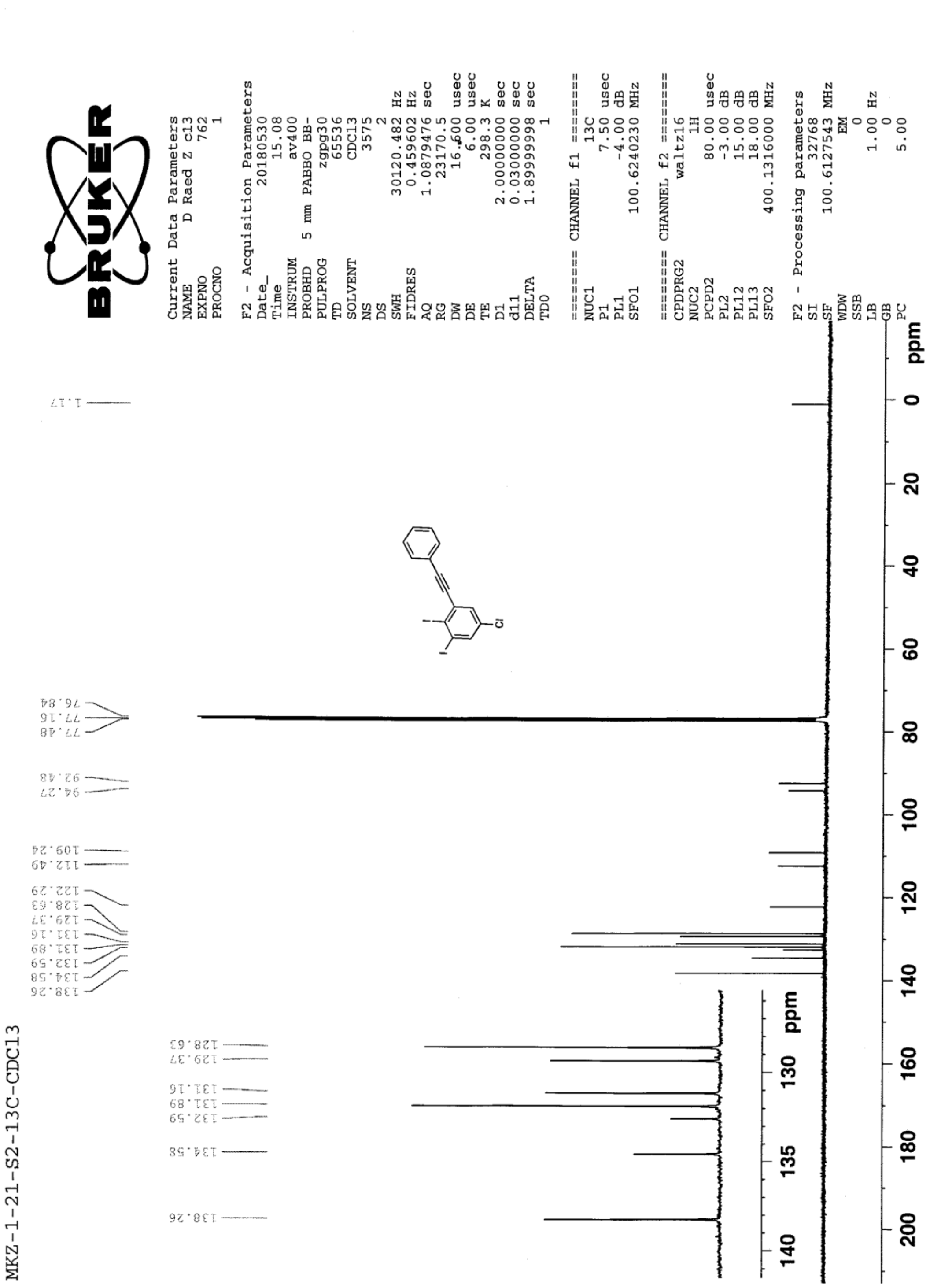

1.5.27 ^{1}H-NMR of 5-chloro-1-((4-ethylphenyl)ethynyl)-2,3-diiodobenzene (18_A) in *$CDCl_3$* at 25 °C.

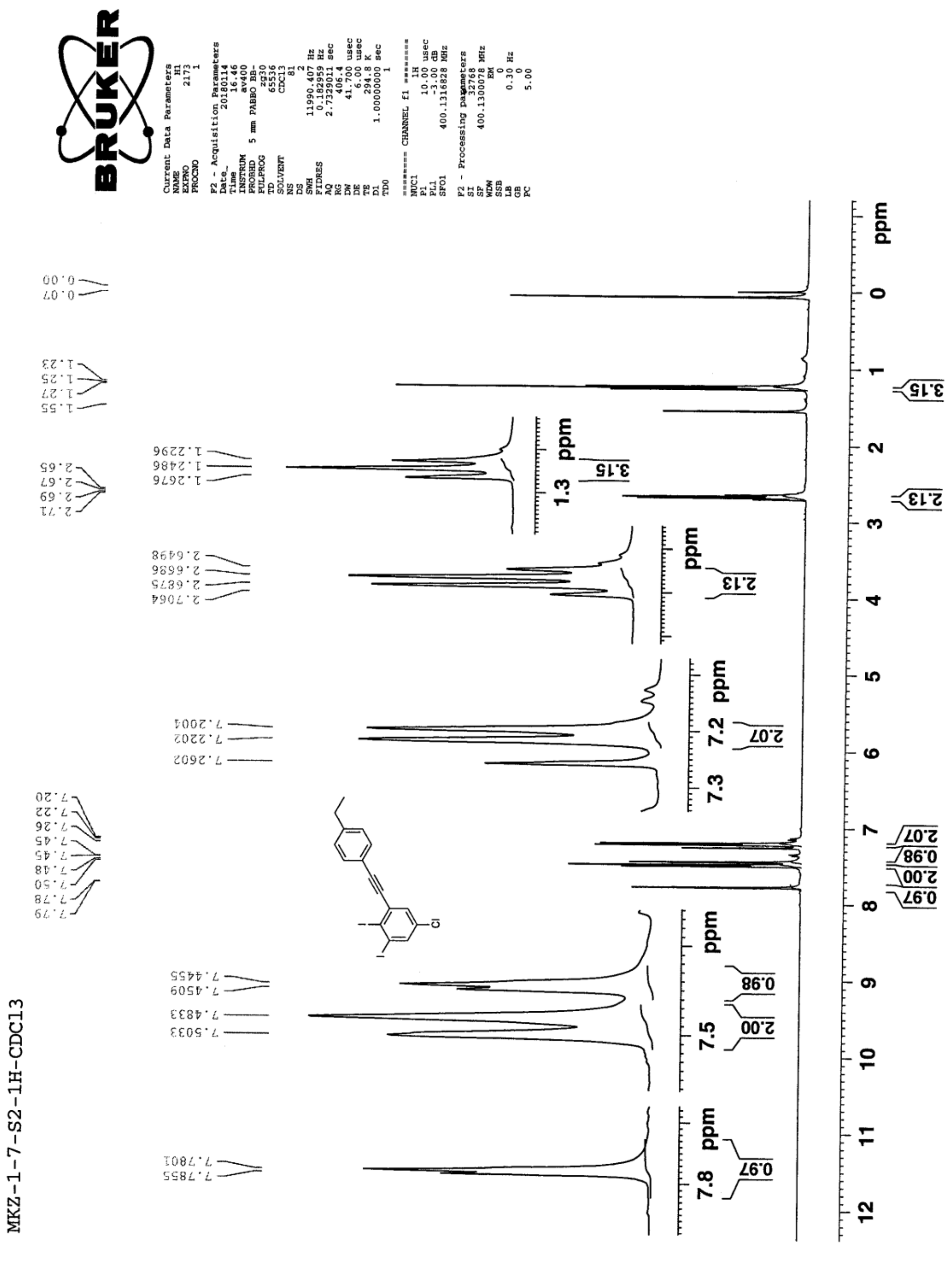

1.5.28 ^{13}C-NMR of 5-chloro-1-((4-ethylphenyl)ethynyl)-2,3-diiodobenzene (18$_A$) in *CDCl*$_3$ at 25 ^{o}C.

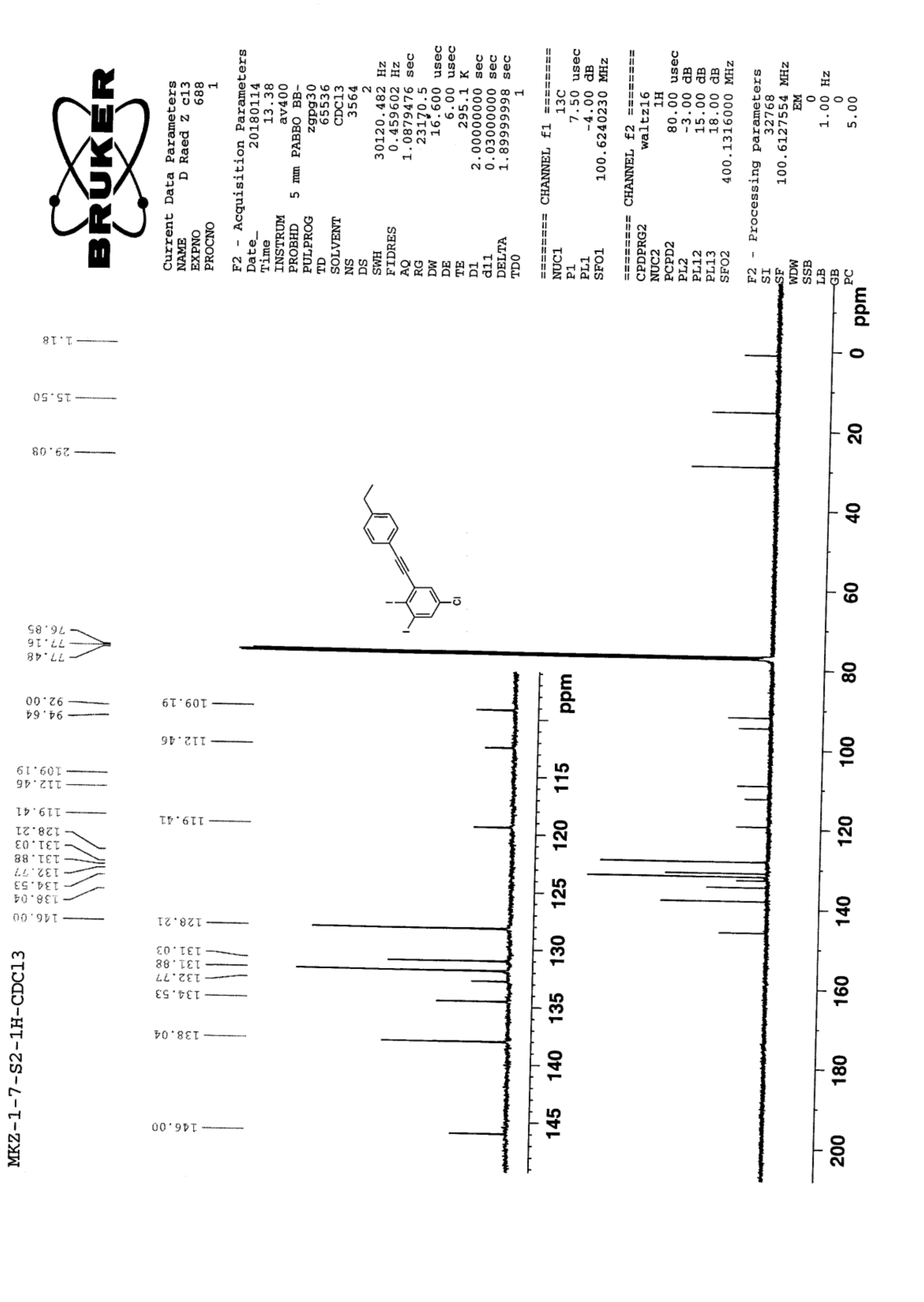

1.5.29 ^{1}H-NMR of 5-chloro-1,2-diiodo-3-((4-methoxyphenyl)ethynyl) benzene (19_A) in $CDCl_3$ at 25 °C.

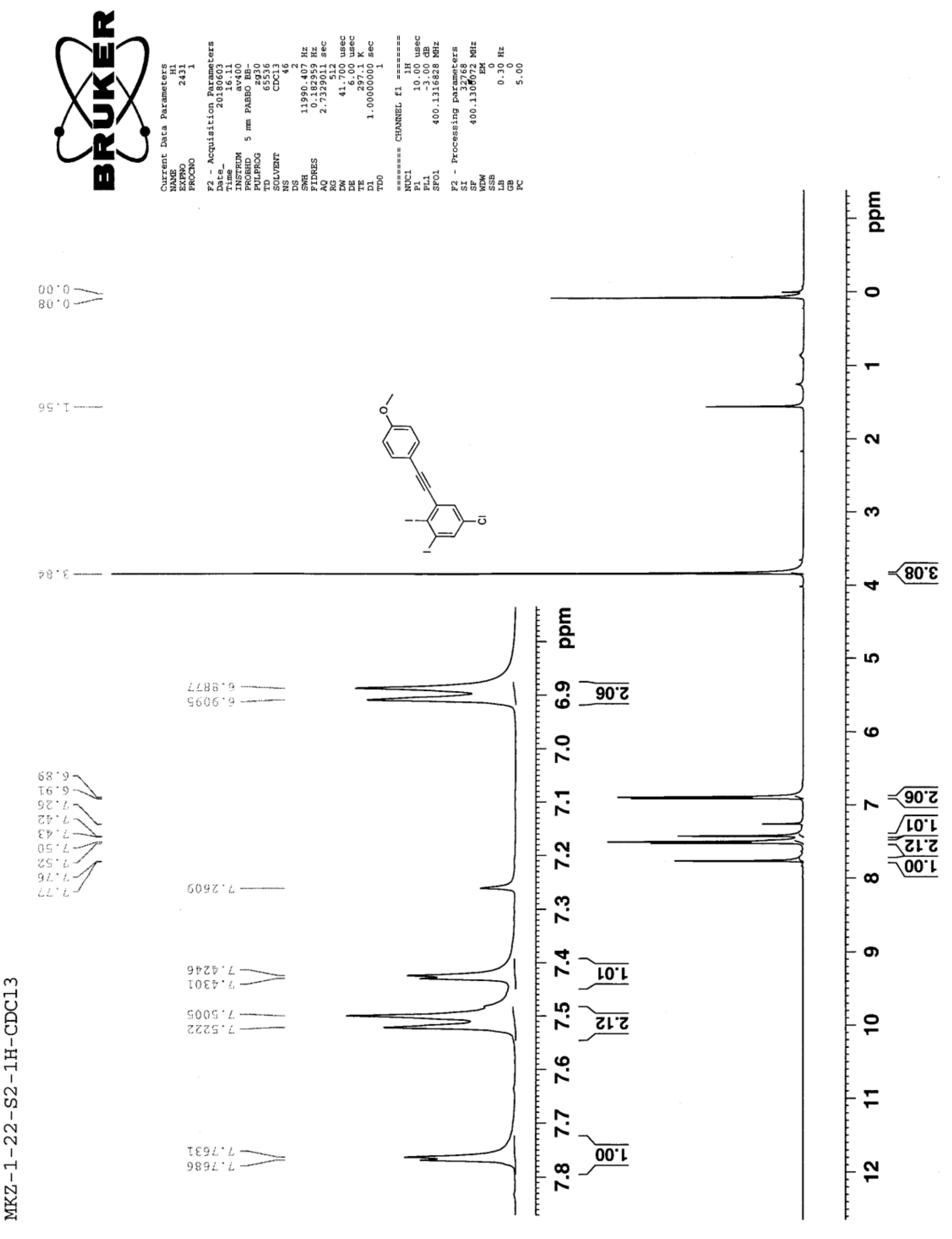

1.5.30 ^{13}C-NMR of 5-chloro-1,2-diiodo-3-((4-methoxyphenyl)ethynyl) benzene (19$_A$) in *CDCl$_3$* at 25 ^{o}C.

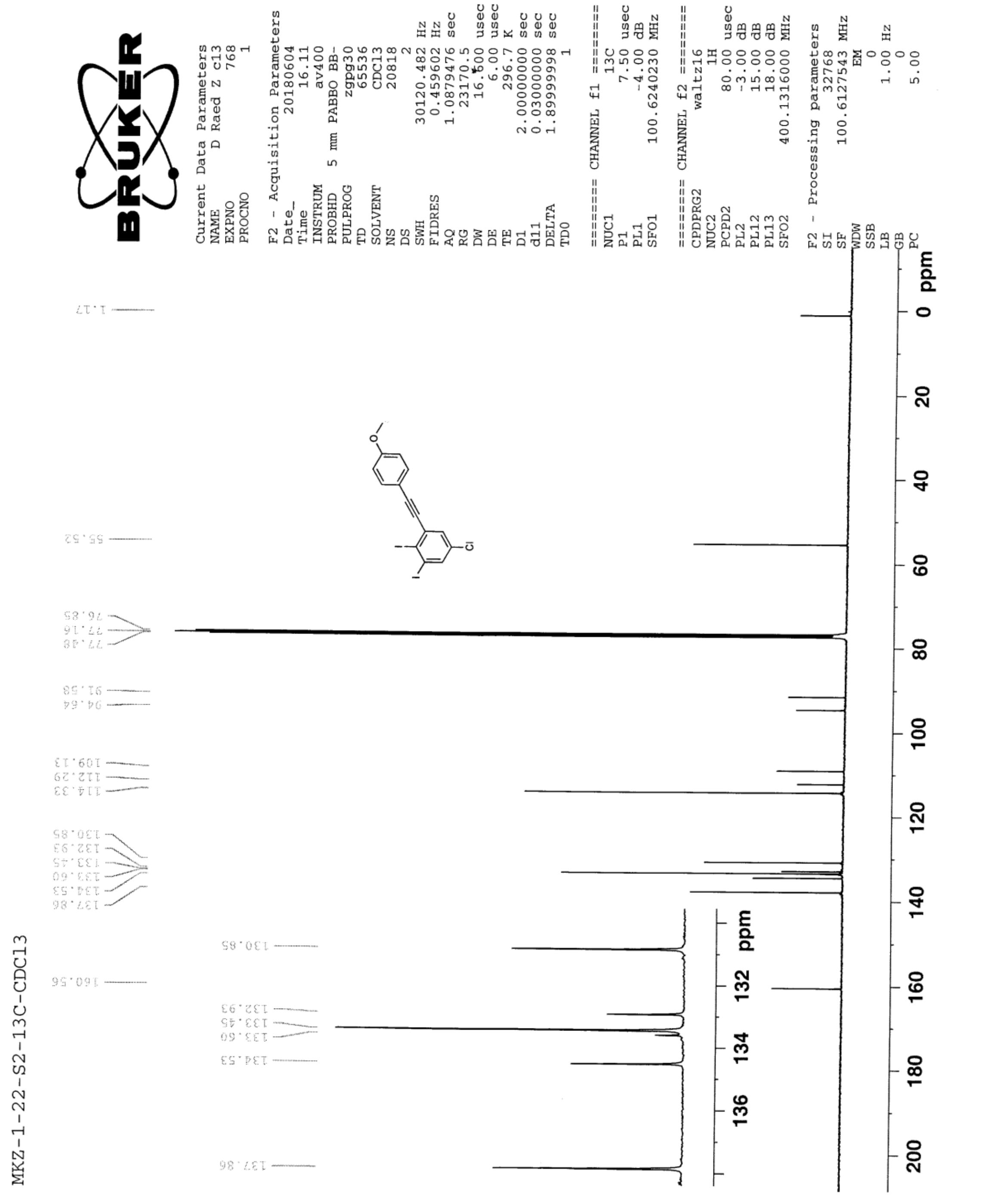

1.5.31 ¹H-NMR of 5-chloro-1,2-diiodo-3-((4-(trifluoromethyl)phenyl) ethynyl)benzene (20_A) in *$CDCl_3$* at 25 °C.

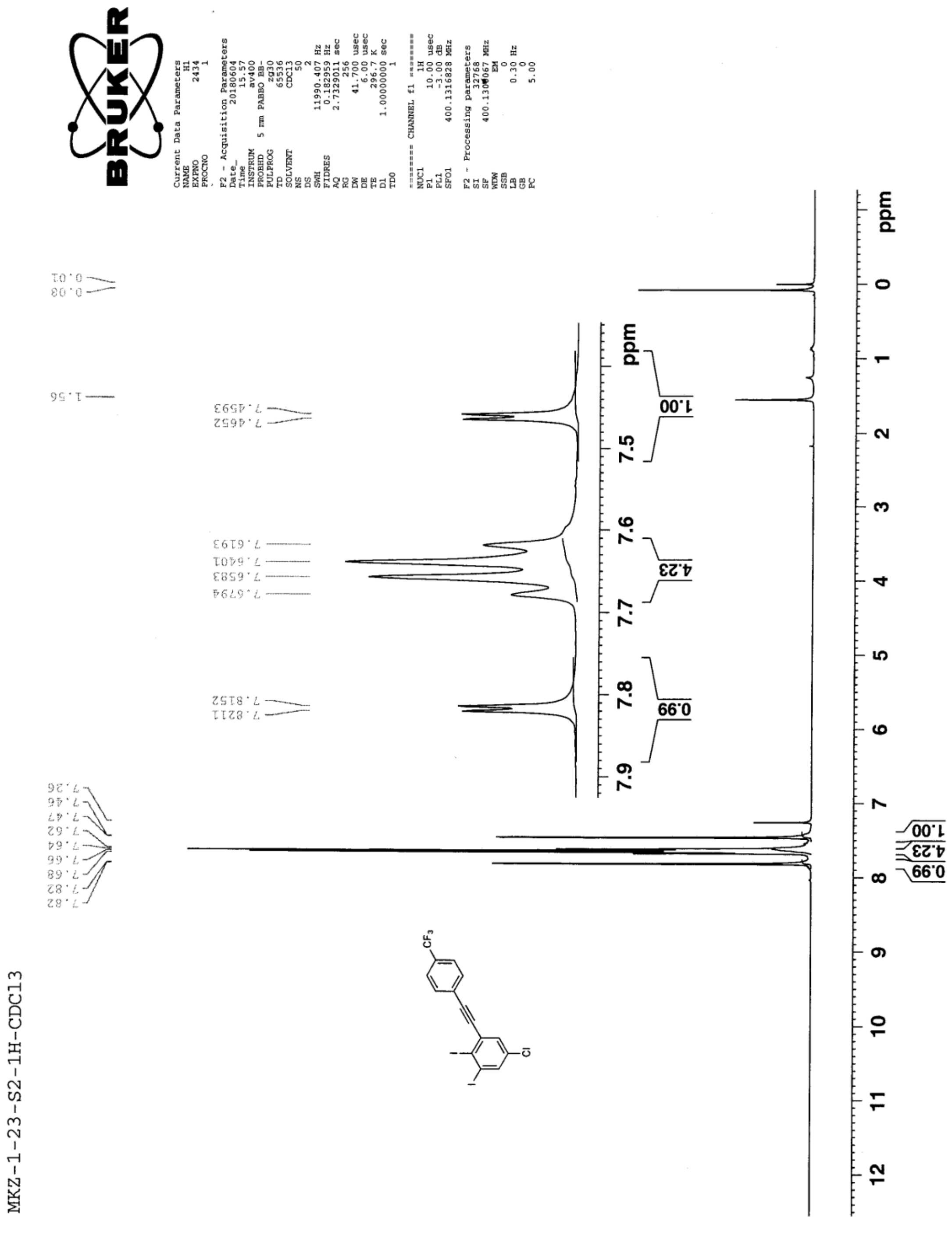

1.5.32 ^{13}C-NMR of 5-chloro-1,2-diiodo-3-((4-(trifluoromethyl)phenyl) ethynyl)benzene (20$_A$) in *CDCl$_3$* at 25 °C.

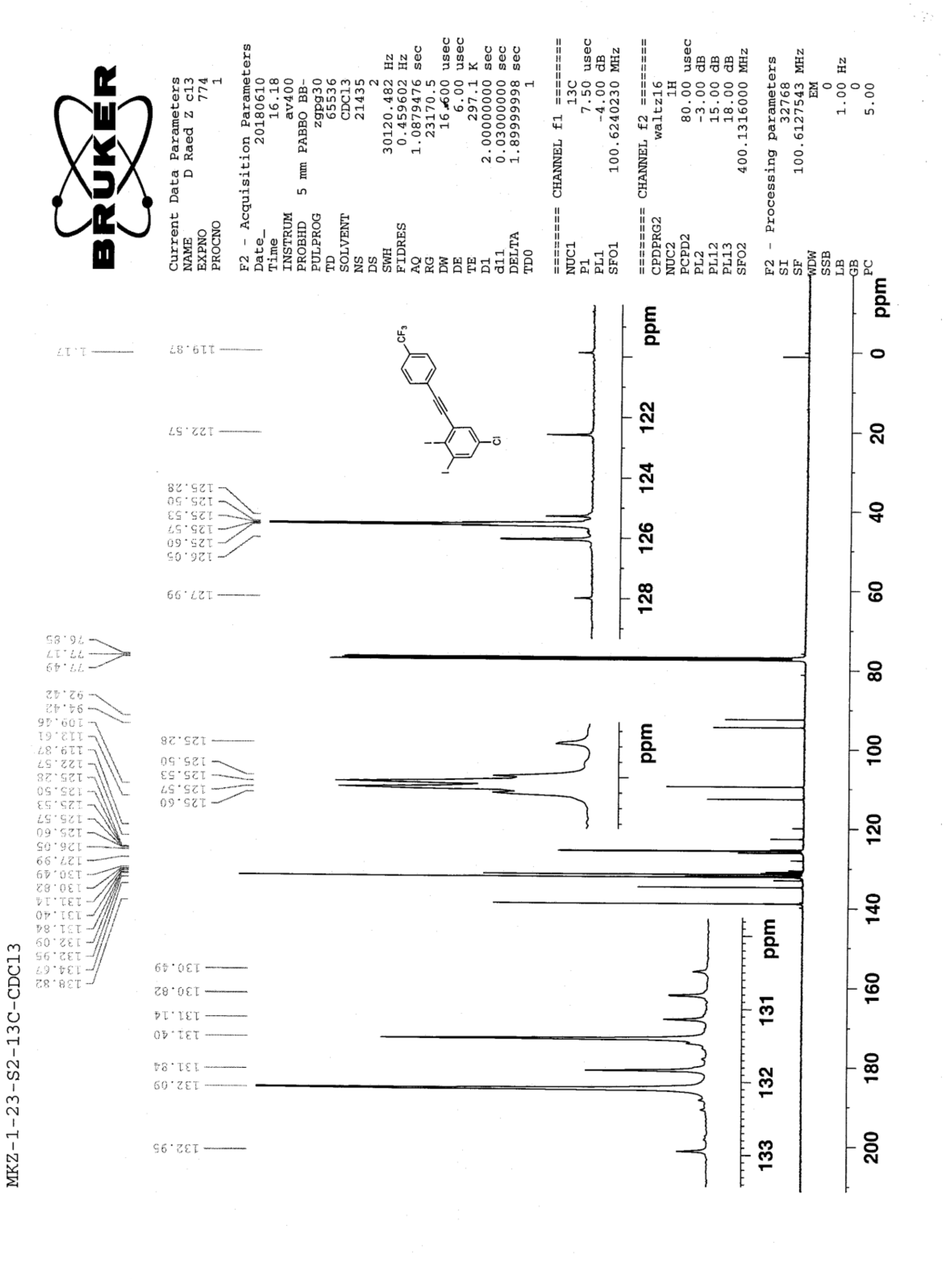

1.5.33 ^{1}H-NMR of 5-bromo-1-((4-ethylphenyl)ethynyl)-2,3-diiodobenzene (21_A) in *$CDCl_3$* at 25 °C.

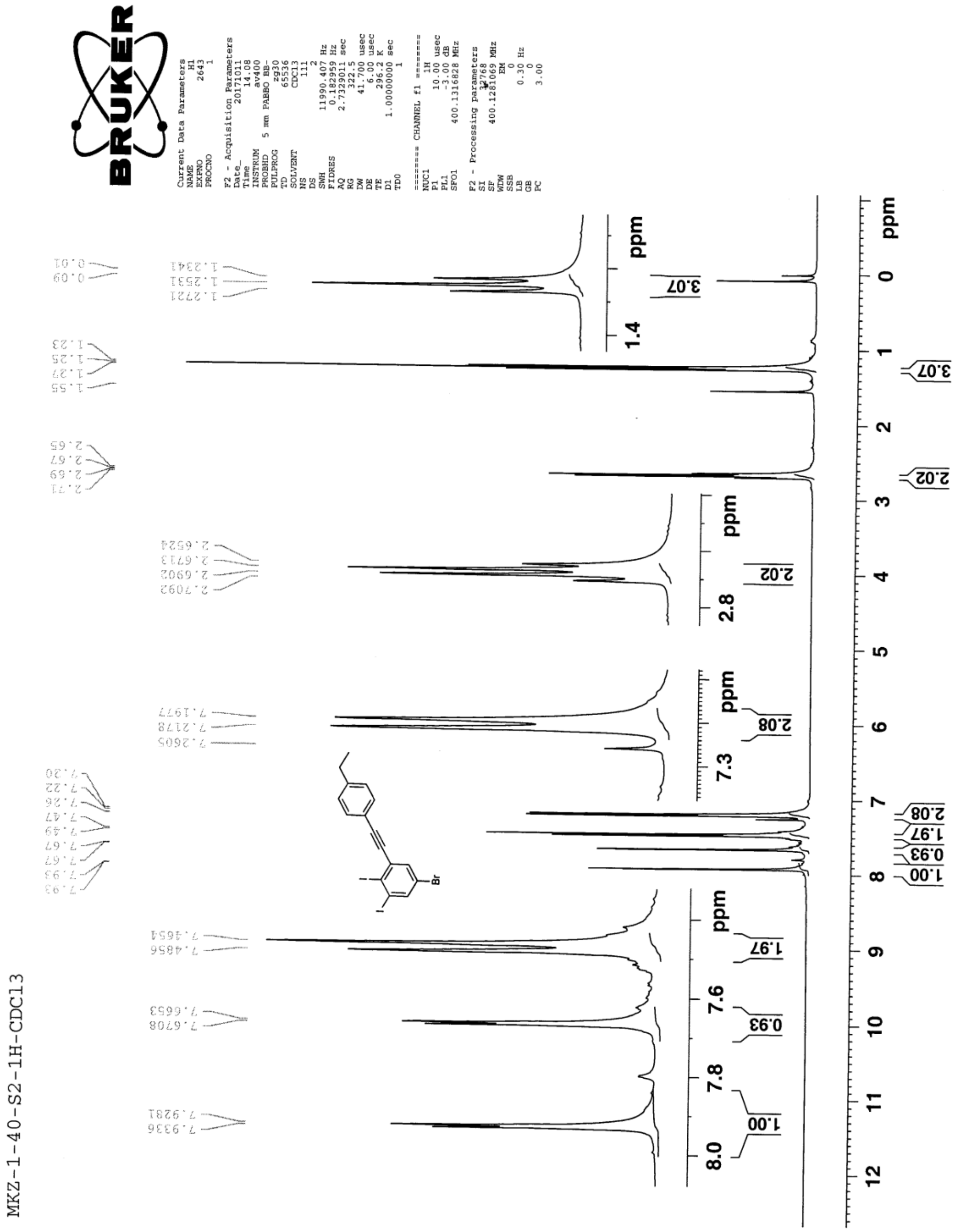

1.5.34 ^{13}C-NMR of 5-bromo-1-((4-ethylphenyl)ethynyl)-2,3-diiodobenzene (21$_A$) in *$CDCl_3$* at 25 °C.

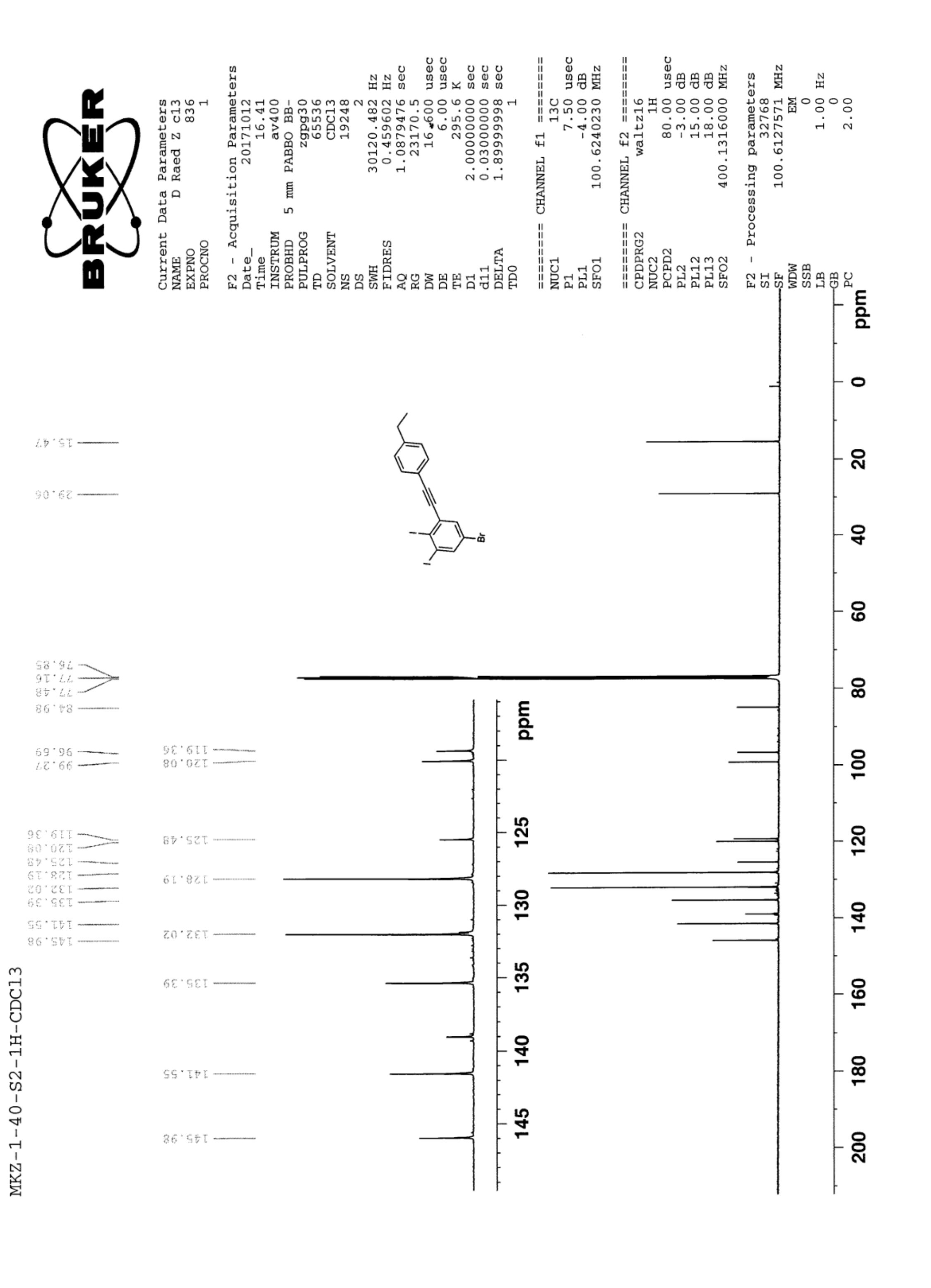

1.5.35 ^{1}H-NMR of 5-bromo-1-((3-fluorophenyl)ethynyl)-2,3-diiodo benzene (22_A) in $CDCl_3$ at 25 °C.

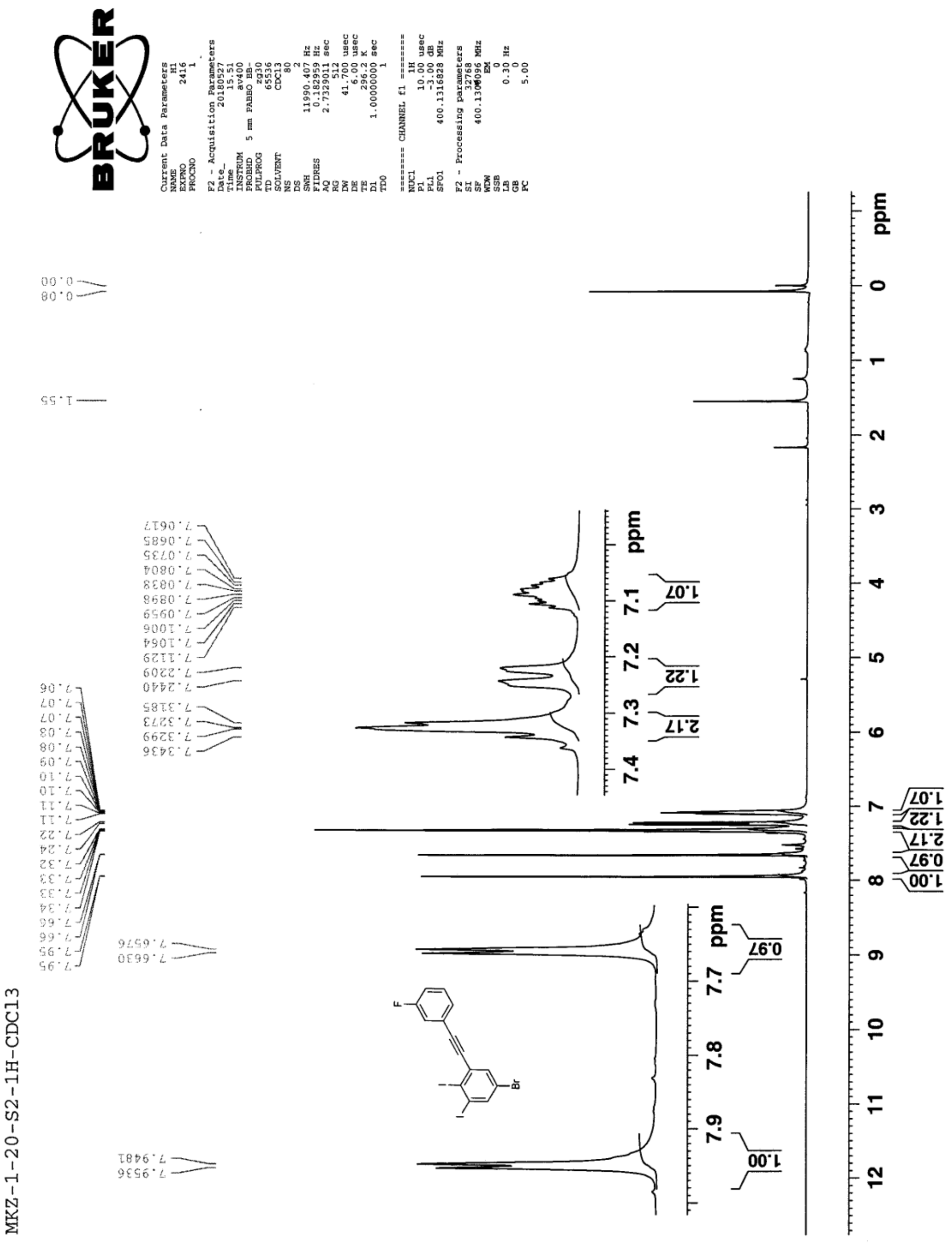

1.5.36 ¹³C-NMR of 5-bromo-1-((3-fluorophenyl)ethynyl)-2,3-diiodobenzene (22A) in *CDCl₃* at 25 °C.

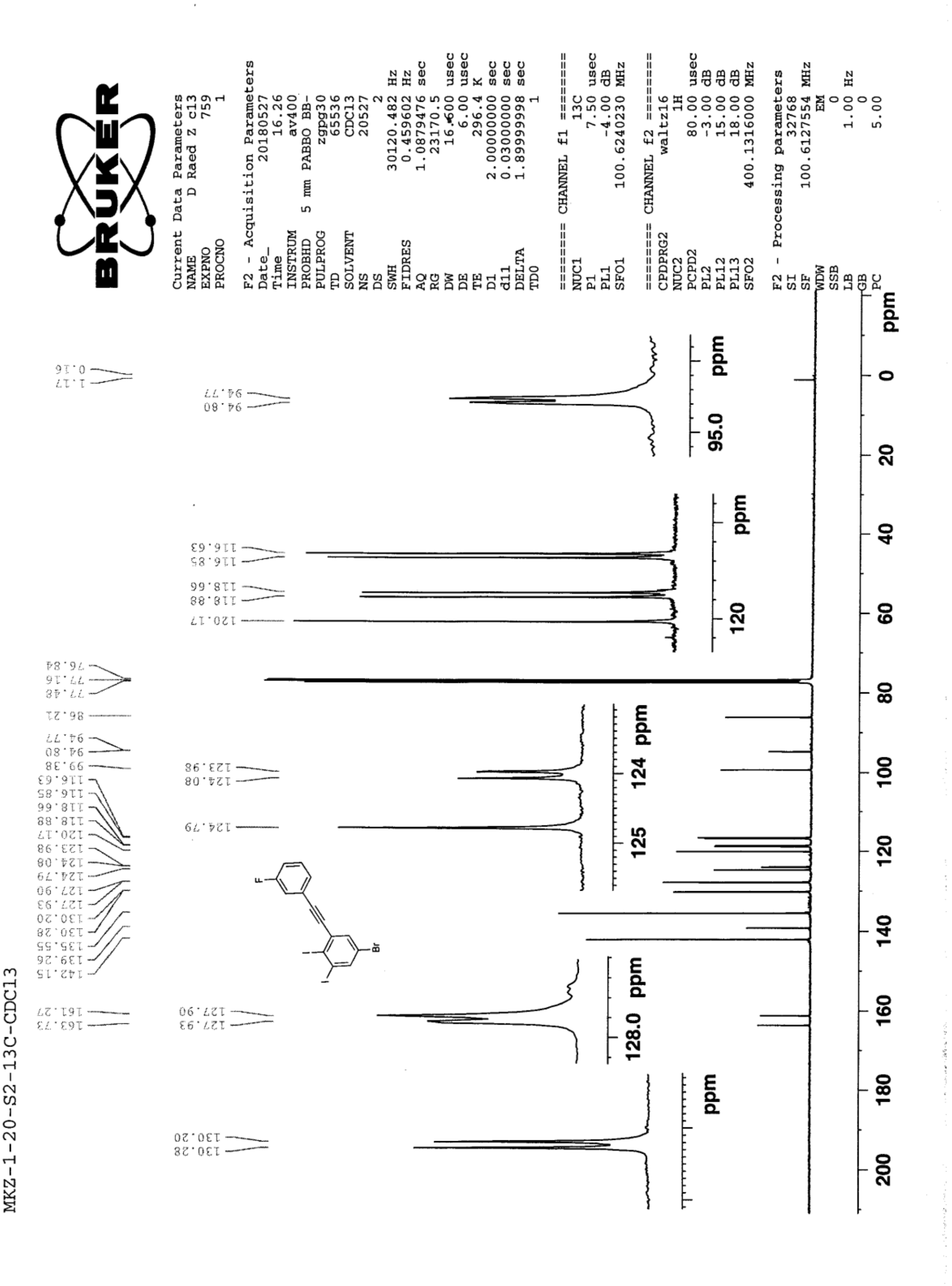

1.5.37 ^{1}H-NMR of 5-fluoro-1,2-diiodo-3-((4-methoxyphenyl)ethynyl) benzene (23_A) in $CDCl_3$ at 25 °C.

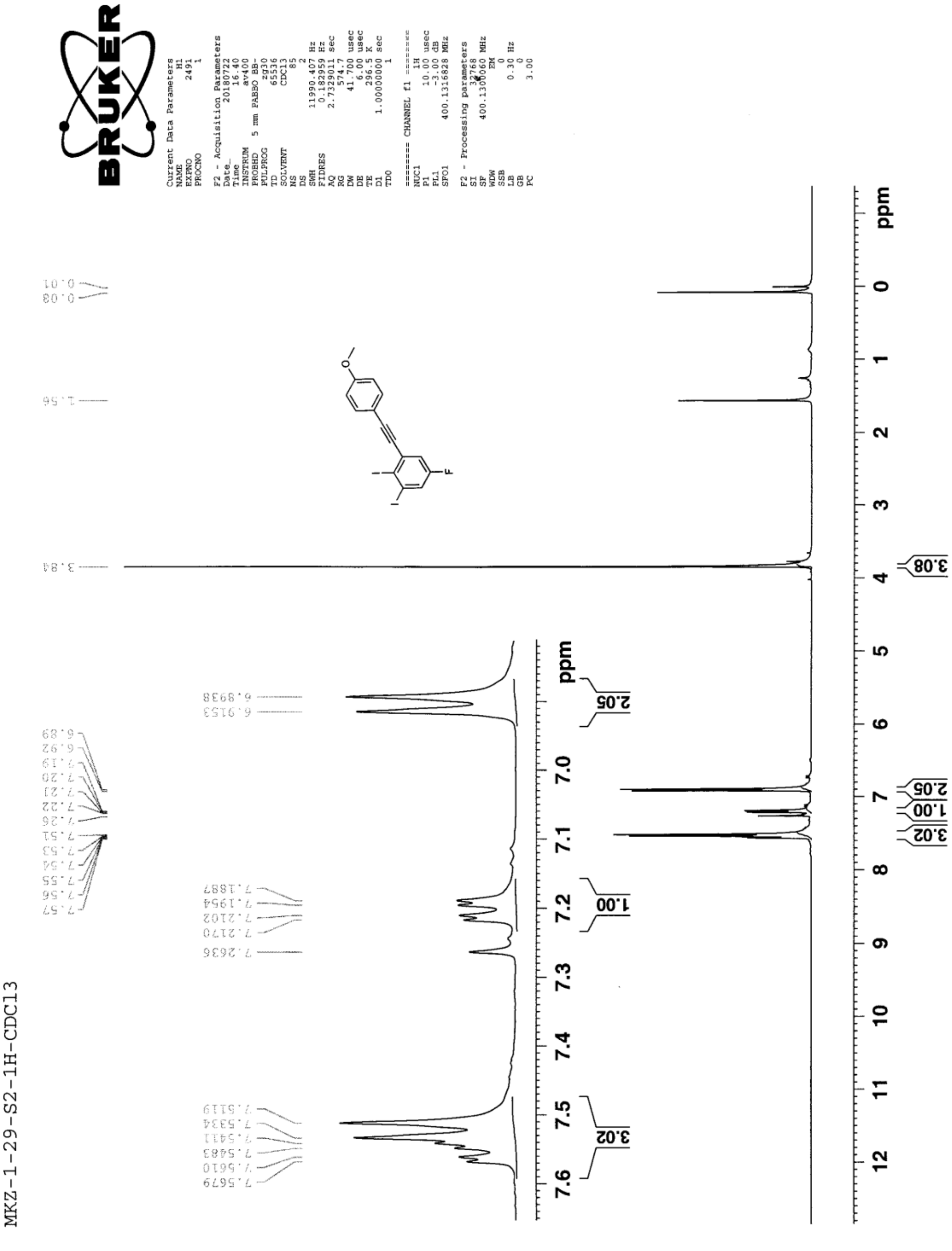

1.5.38 ^{13}C-NMR of 5-fluoro-1,2-diiodo-3-((4-methoxyphenyl)ethynyl) benzene (23_A) in *$CDCl_3$* at 25 °C.

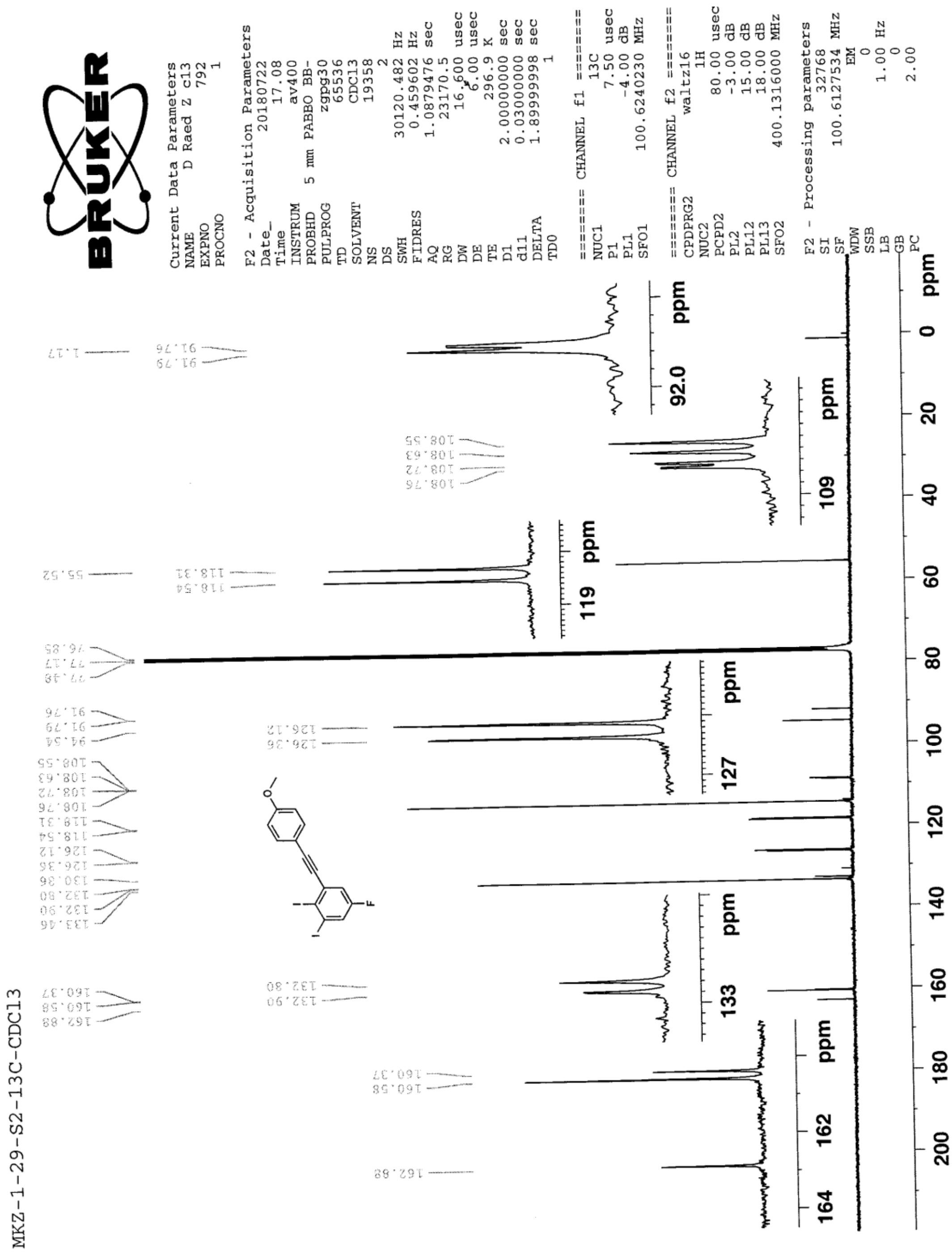

1.5.39 ¹H-NMR of 1-((4-chlorophenyl)ethynyl)-5-fluoro-2,3-diiodobenzene (24A) in *CDCl₃* at 25 °C.

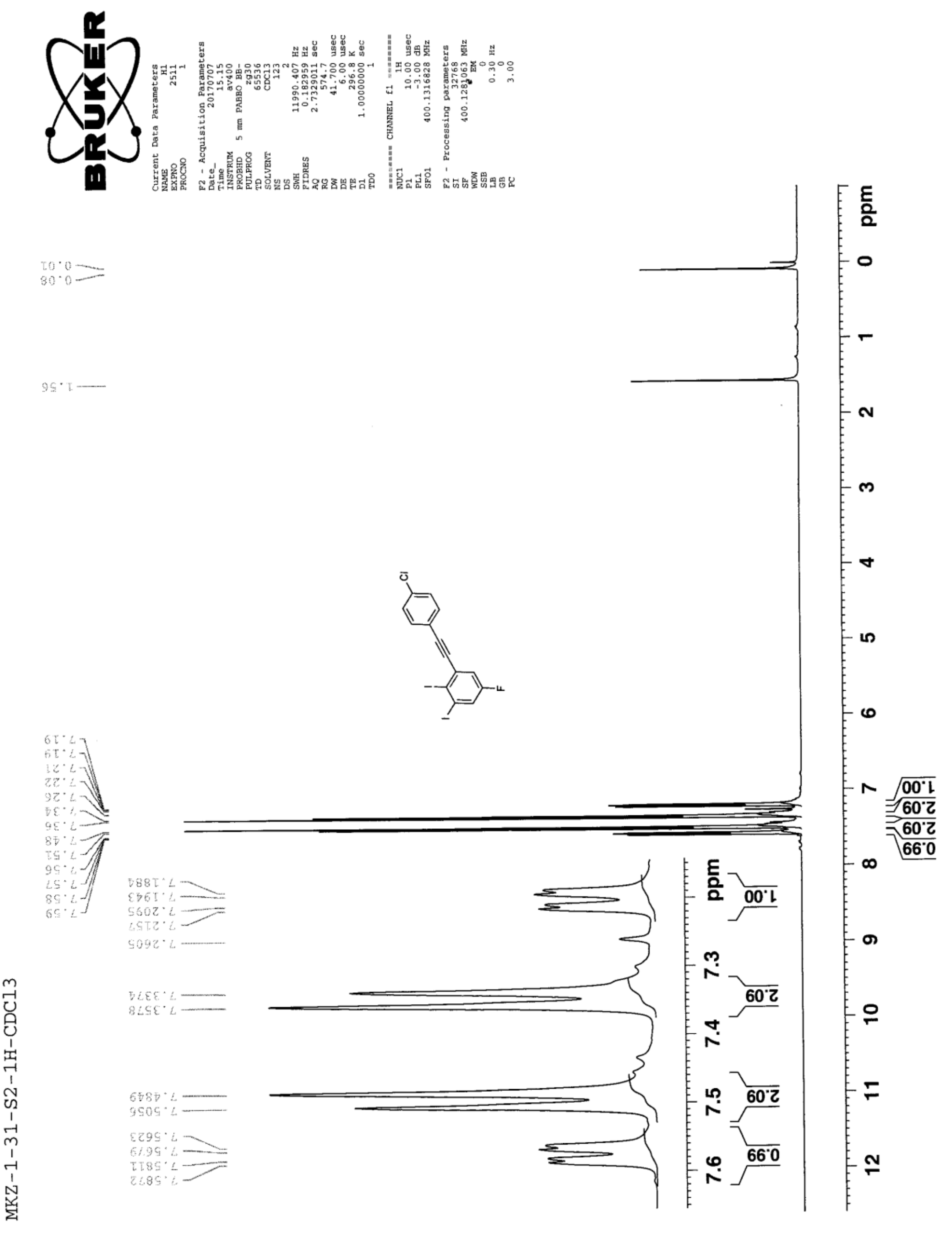

1.5.40 ^{13}C-NMR of 1-((4-chlorophenyl)ethynyl)-5-fluoro-2,3-diiodobenzene (24A) in *CDCl*3 at 25 °C.

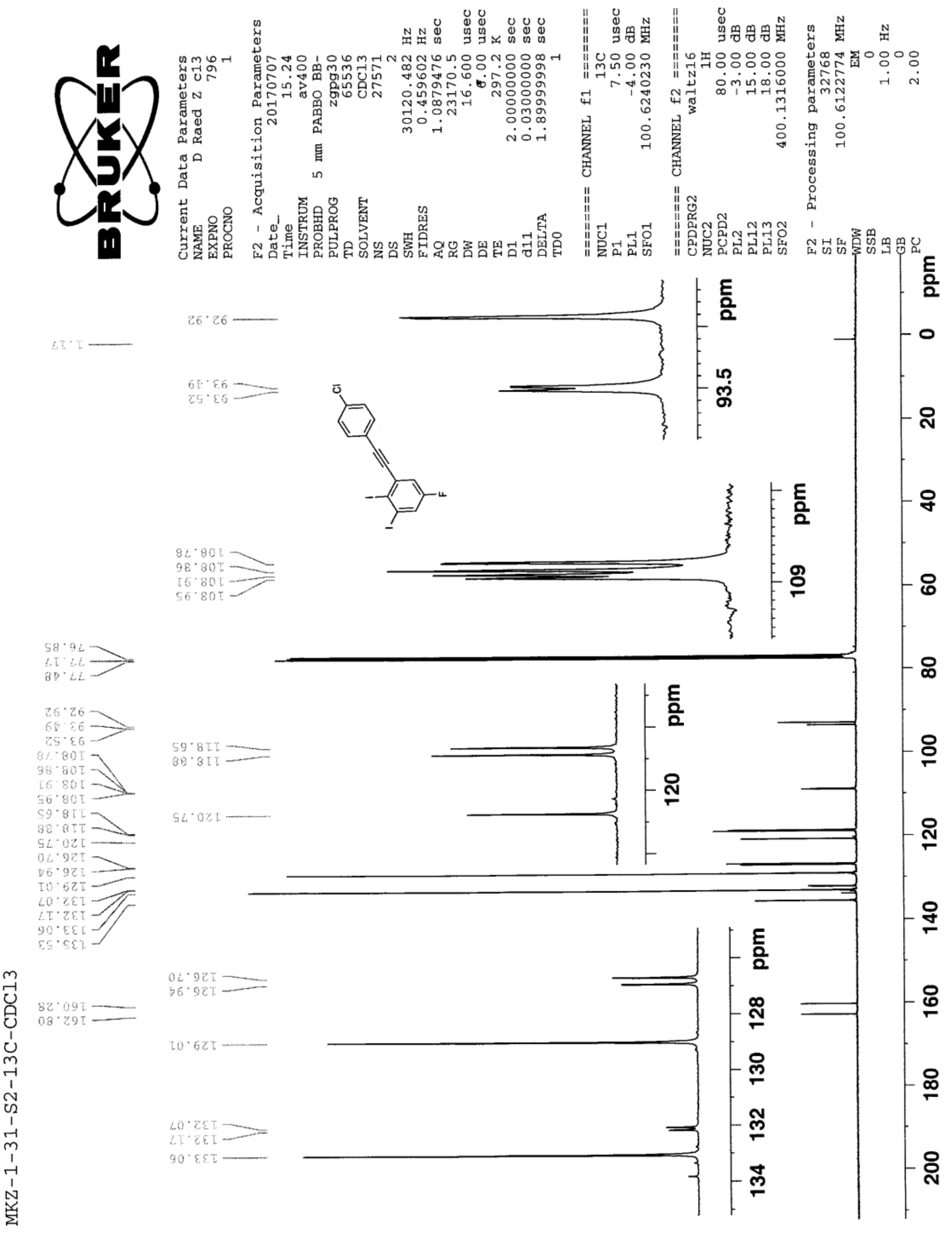

1.5.41 ^{1}H-NMR of methyl 3,4-diiodo-5-((4-methoxyphenyl) ethynyl) benzoate (25_A) in *$CDCl_3$* at 25 °C.

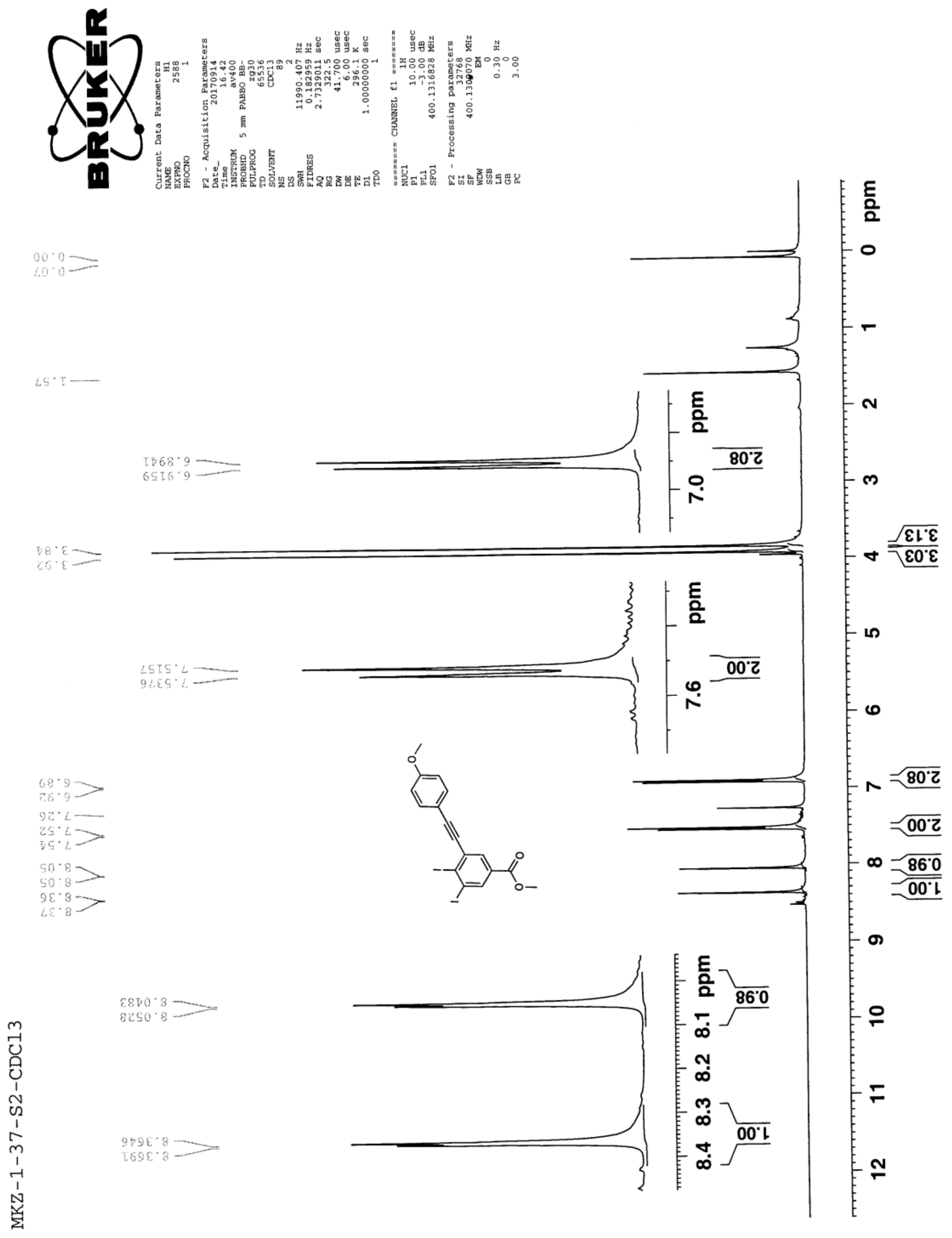

1.5.42 ^{13}C-NMR of methyl 3,4-diiodo-5-((4-methoxyphenyl) ethynyl) benzoate (25_A) in *$CDCl_3$* at 25 ^oC.

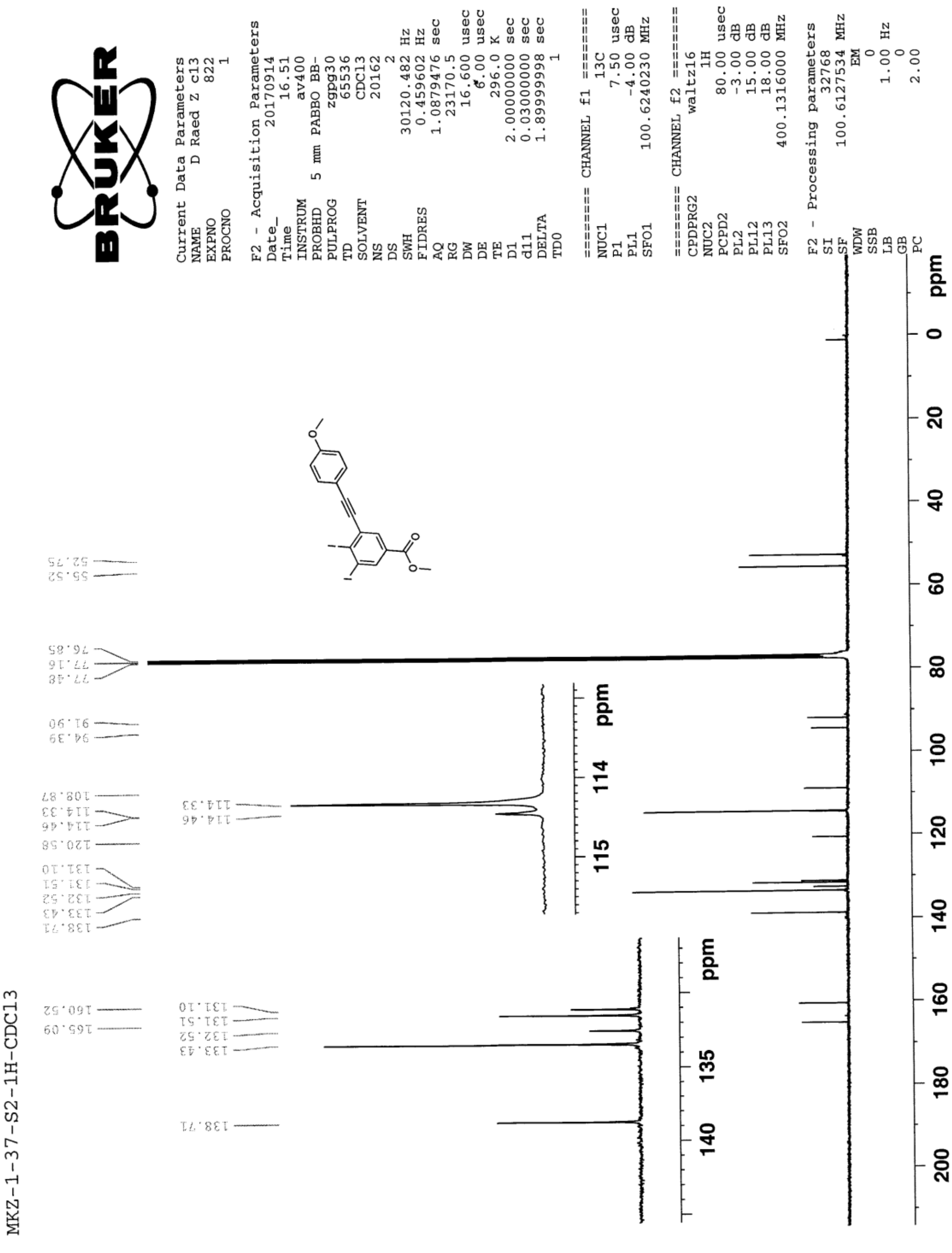

R. M. Al-Zoubi, M. K. Al-Omari, W. K. Al-Jammal and M. J. Ferguson

1.5.43 ^{1}H-NMR of 1-chloro-3-((4-chlorophenyl)ethynyl)-2-iodobenzene (26$_A$) in $CDCl_3$ at 25 ^{o}C.

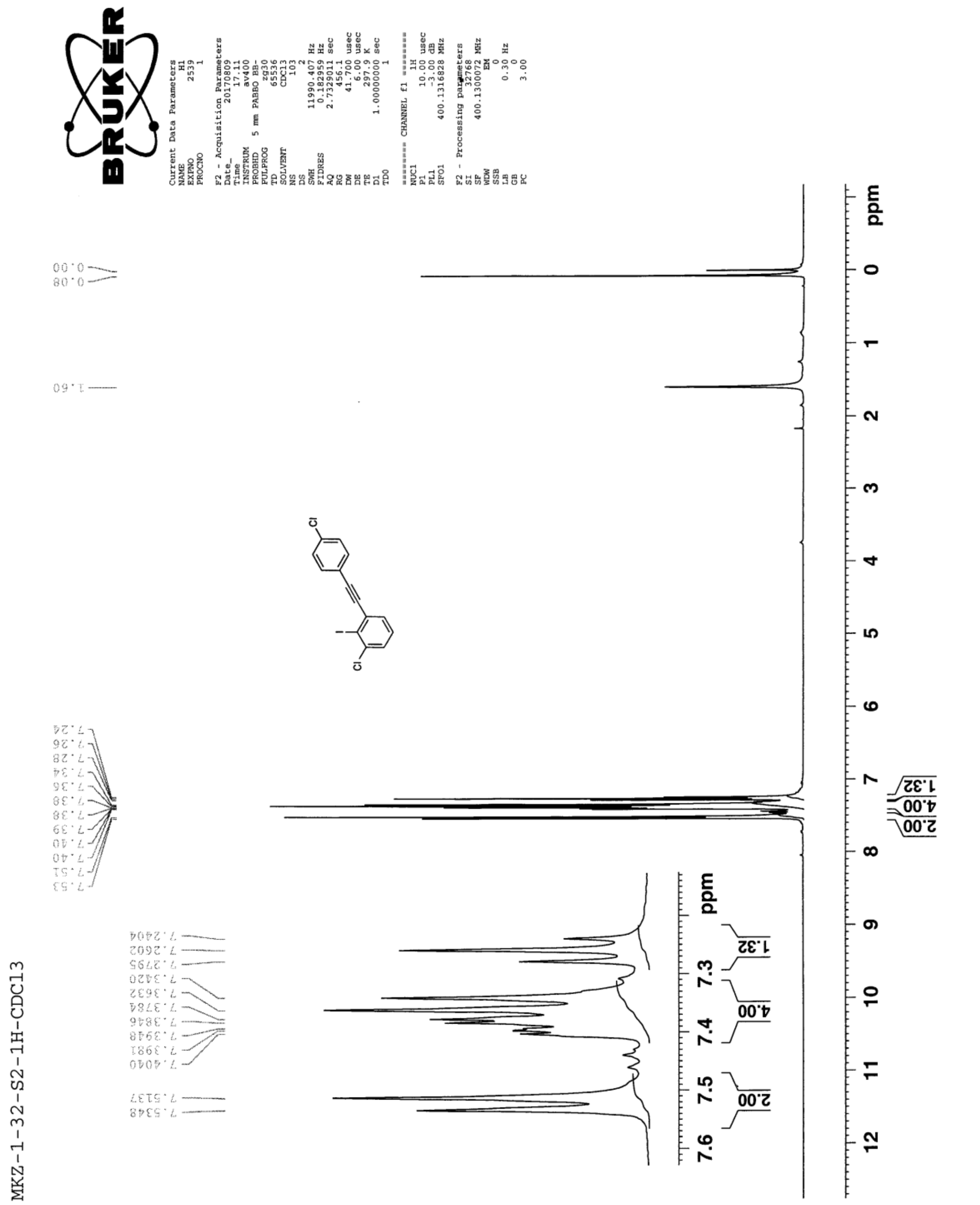

1.5.44 ^{13}C-NMR of 1-chloro-3-((4-chlorophenyl)ethynyl)-2-iodobenzene (26A) in *CDCl3* at 25 °C.

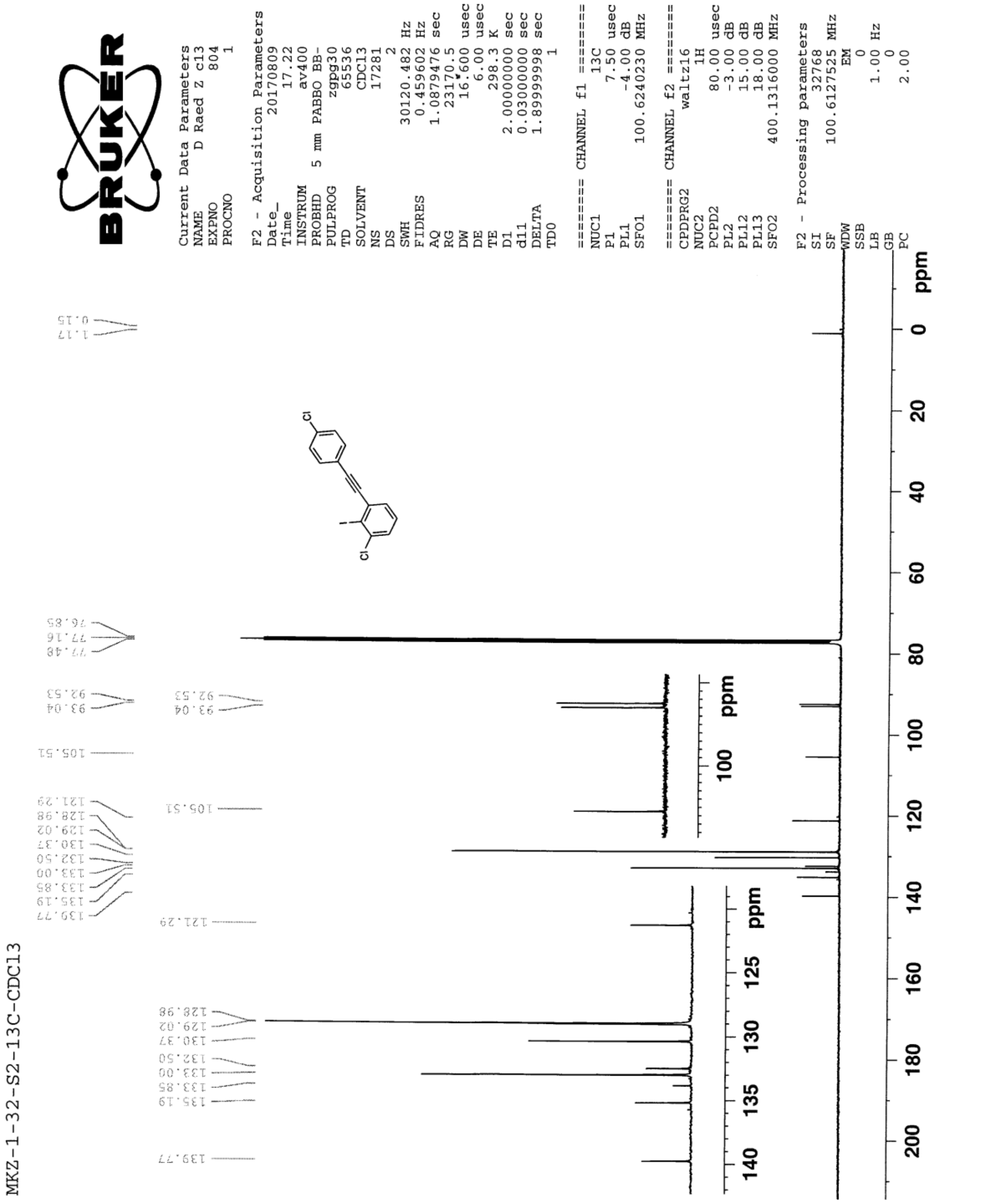

1.5.45 ^{1}H-NMR of 1-bromo-2-iodo-3-((4-methoxyphenyl)ethynyl)benzene (27$_A$) in *CDCl$_3$* at 25 ^{o}C.

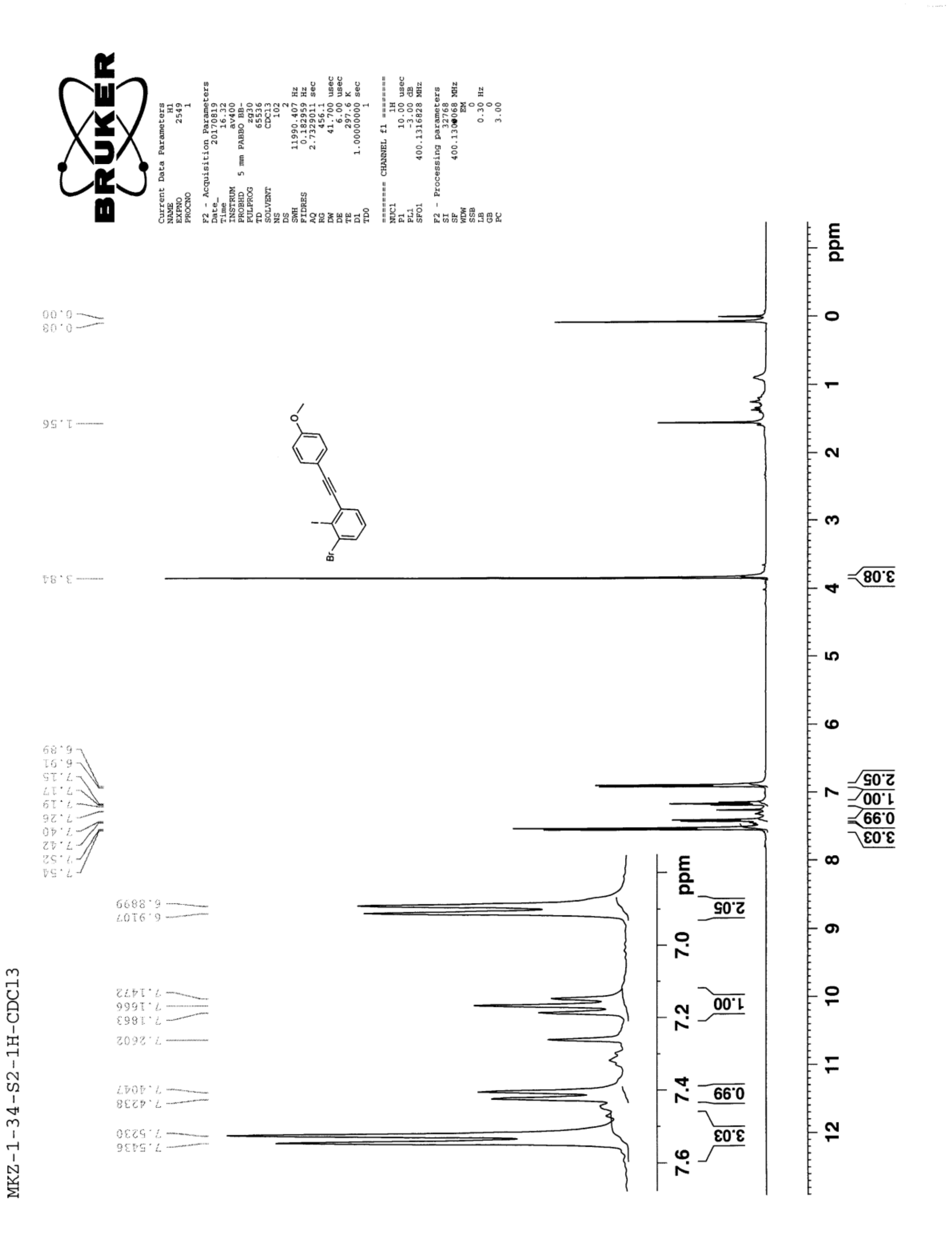

1.5.46 ^{13}C-NMR of 1-bromo-2-iodo-3-((4-methoxyphenyl)ethynyl)benzene (27$_A$) in *CDCl$_3$* at 25 ^{o}C.

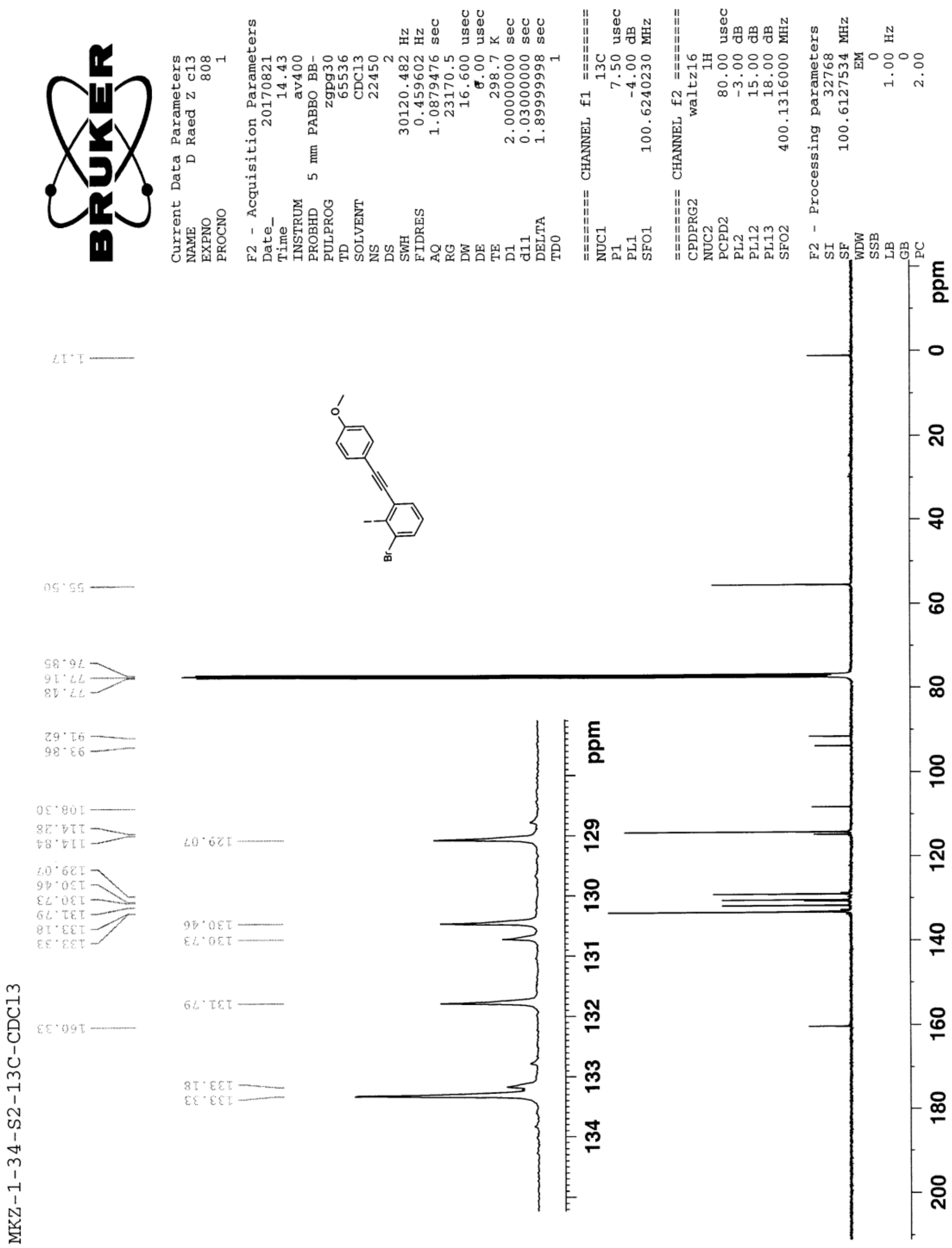

1.5.47 ^{1}H-NMR of 2-iodo-5-methoxy-1-((4-methoxyphenyl)ethynyl)-3-((4-(trifluoromethyl)phenyl)ethynyl)benzene (28$_A$) in *CDCl$_3$* at 25 ^{o}C.

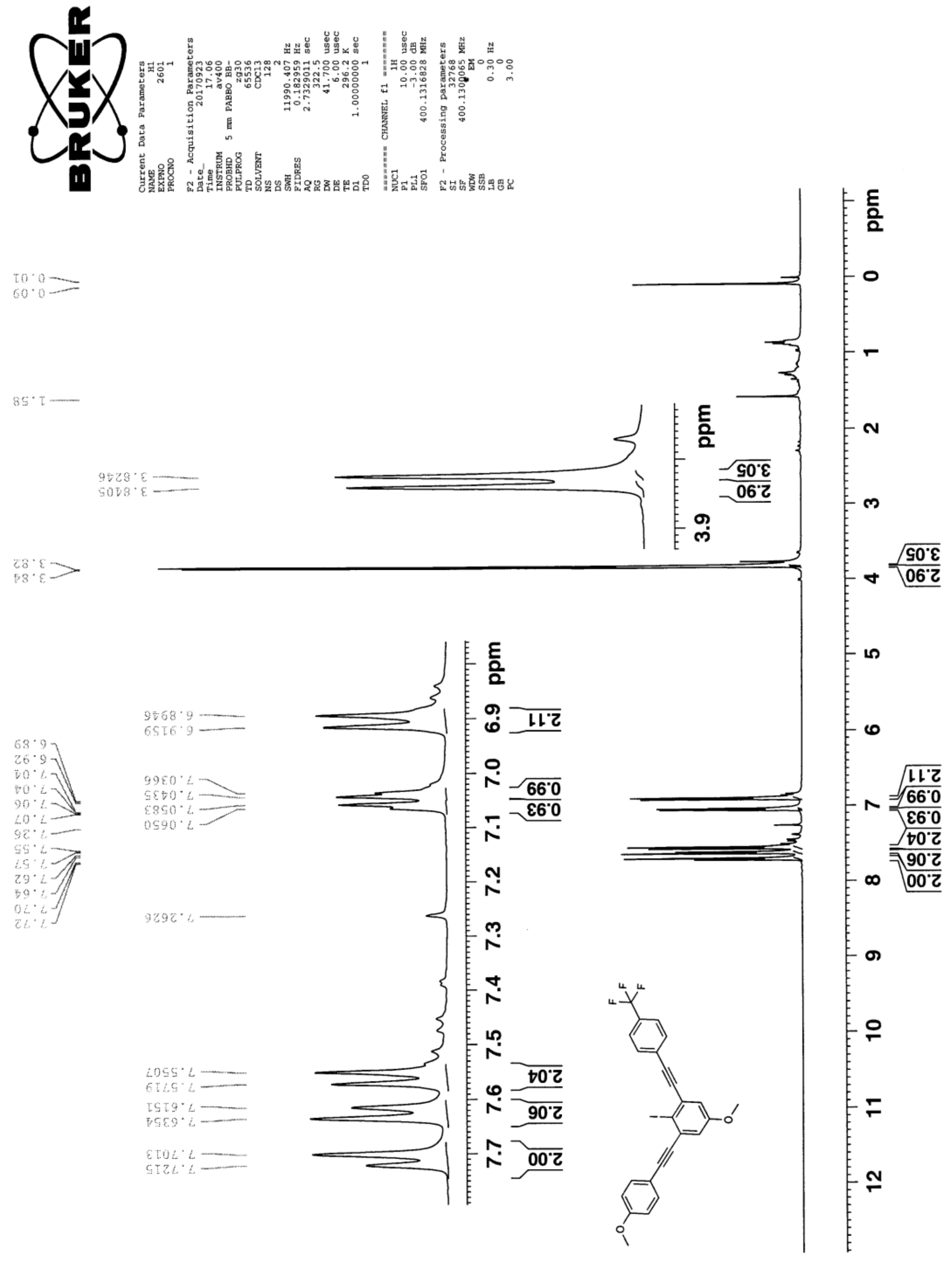

1.5.48 ^{13}C-NMR of 2-iodo-5-methoxy-1-((4-methoxyphenyl)ethynyl)-3-((4-(trifluoromethyl)phenyl)ethynyl)benzene (28_A) in *$CDCl_3$* at 25 °C.

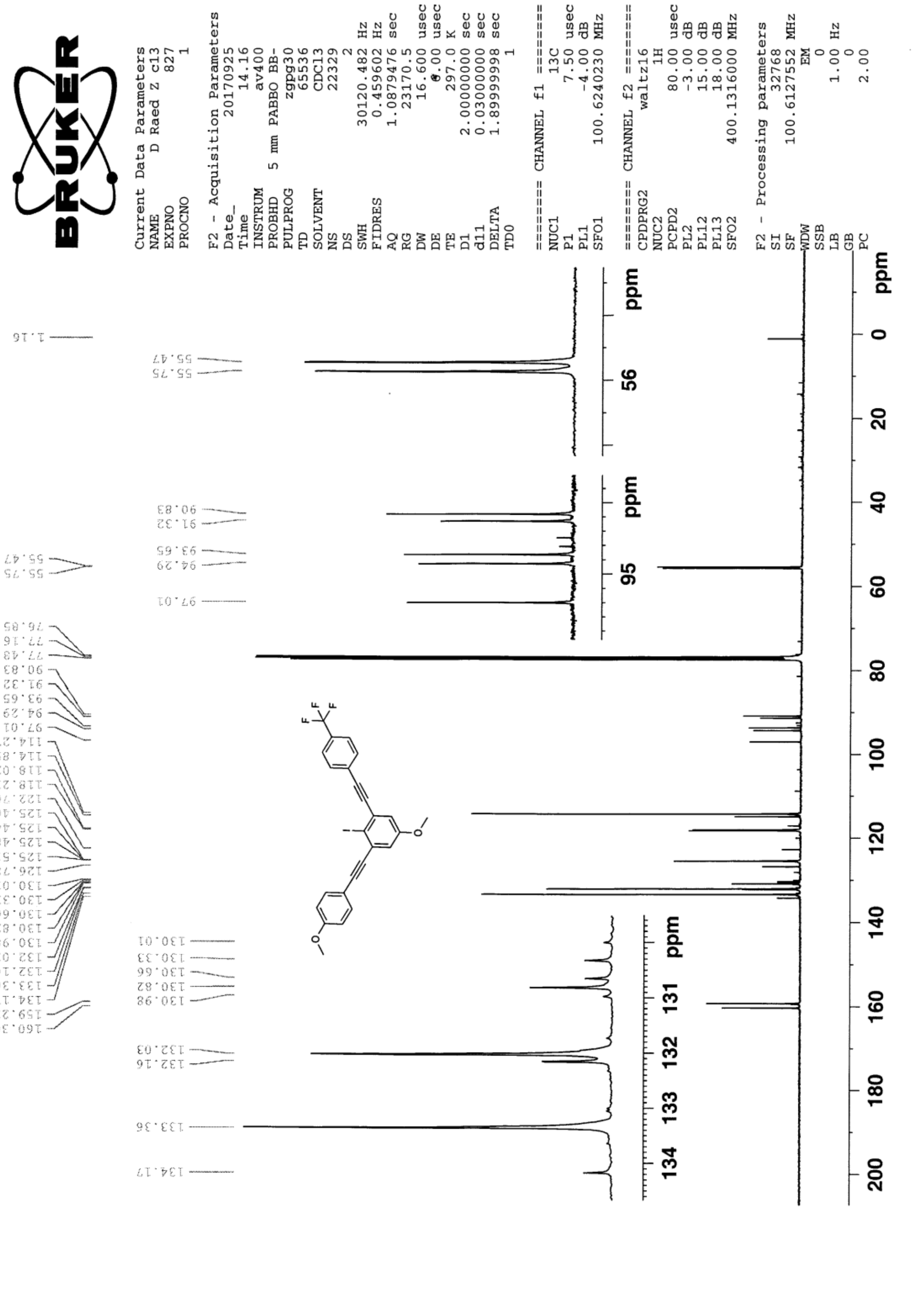

R. M. Al-Zoubi, M. K. Al-Omari, W. K. Al-Jammal and M. J. Ferguson

1.5.49 ¹H-NMR of 5-bromo-1-((4-ethylphenyl)ethynyl)-3-((3-fluorophenyl) ethynyl)-2-iodobenzene (29_A) in *$CDCl_3$* at 25 °C.

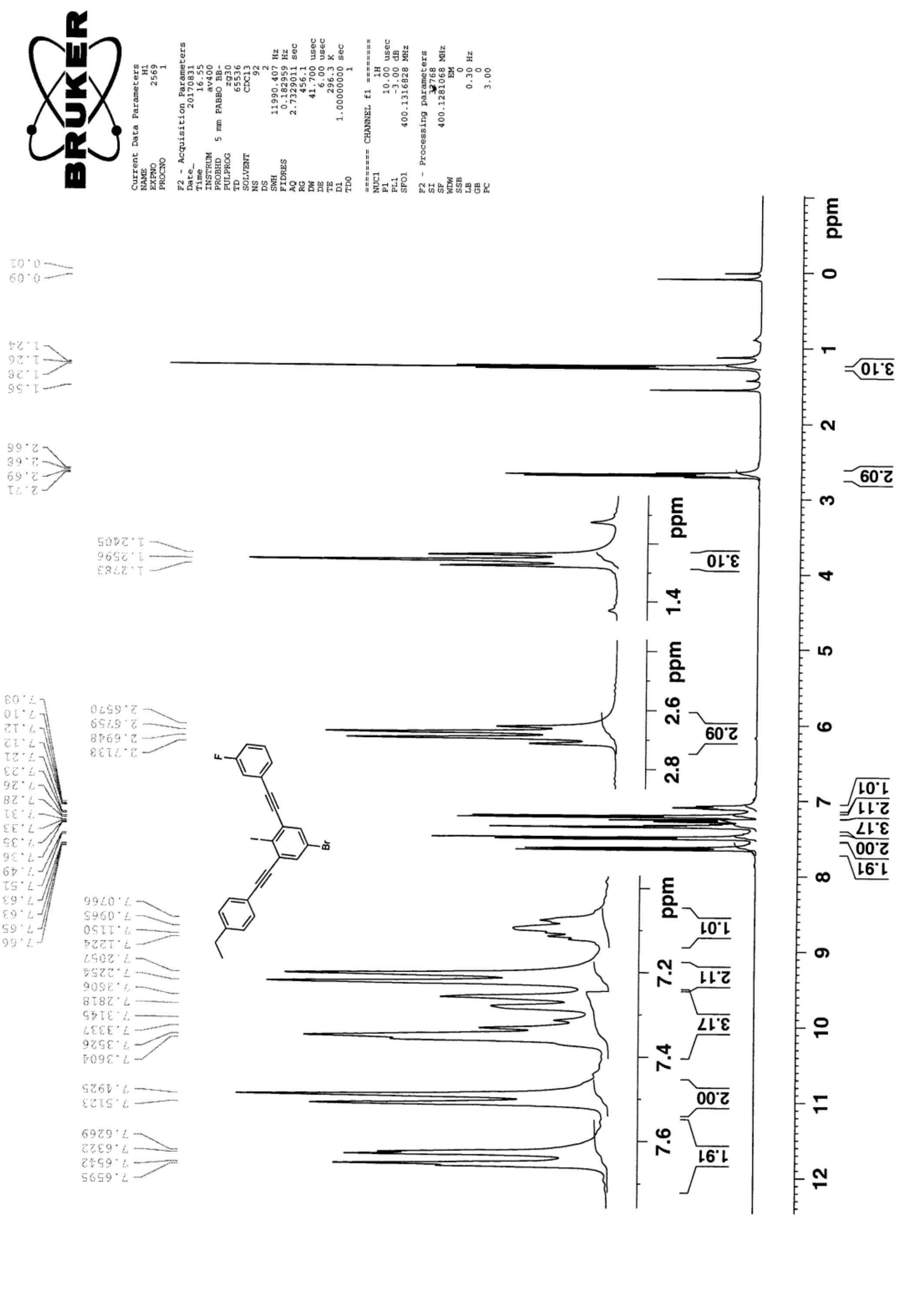

1.5.50 ^{13}C-NMR of 5-bromo-1-((4-ethylphenyl)ethynyl)-3-((3-fluorophenyl) ethynyl)-2-iodobenzene (29_A) in *$CDCl_3$* at 25 °C.

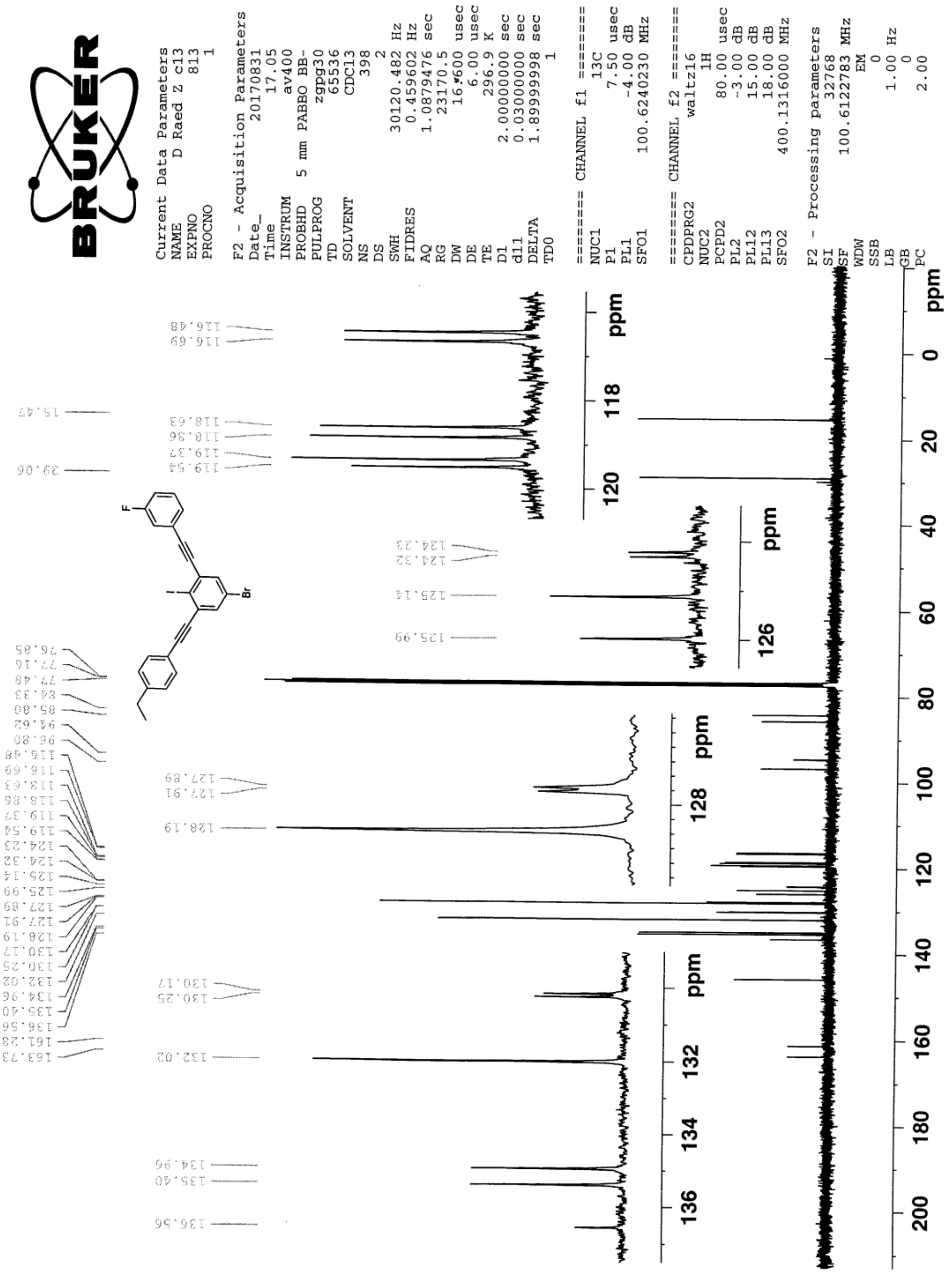

R. M. Al-Zoubi, M. K. Al-Omari, W. K. Al-Jammal and M. J. Ferguson

1.5.51 ^{1}H-NMR of 4,4'-((2-iodo-1,3-phenylene)bis(ethyne-2,1-diyl))bis (methoxybenzene) (30_A) in *$CDCl_3$* at 25 ^{o}C.

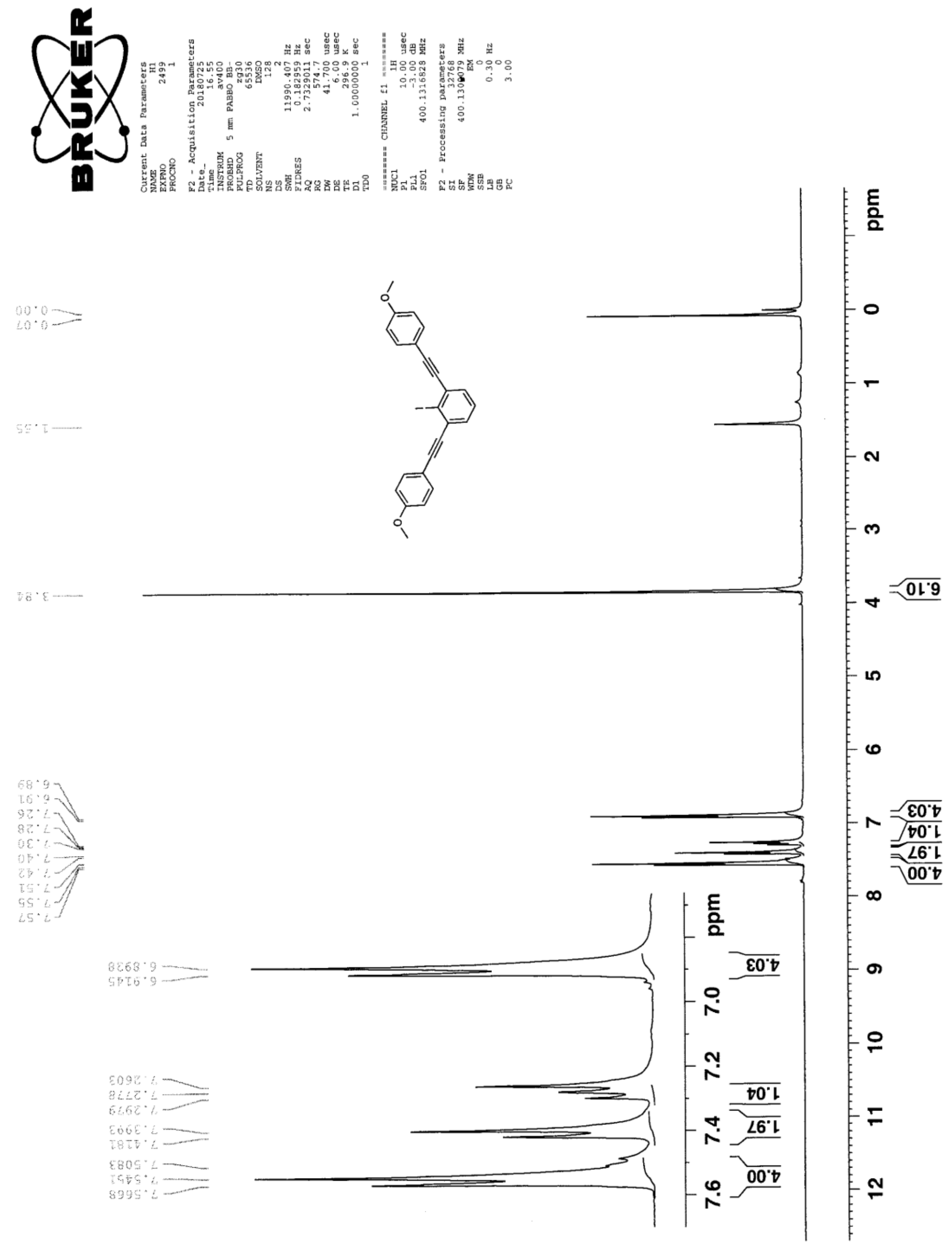

1.5.52 ^{13}C-NMR of 4,4'-((2-iodo-1,3-phenylene)bis(ethyne-2,1-diyl))bis (methoxybenzene) (30$_A$) in *CDCl$_3$* at 25 ^{o}C.

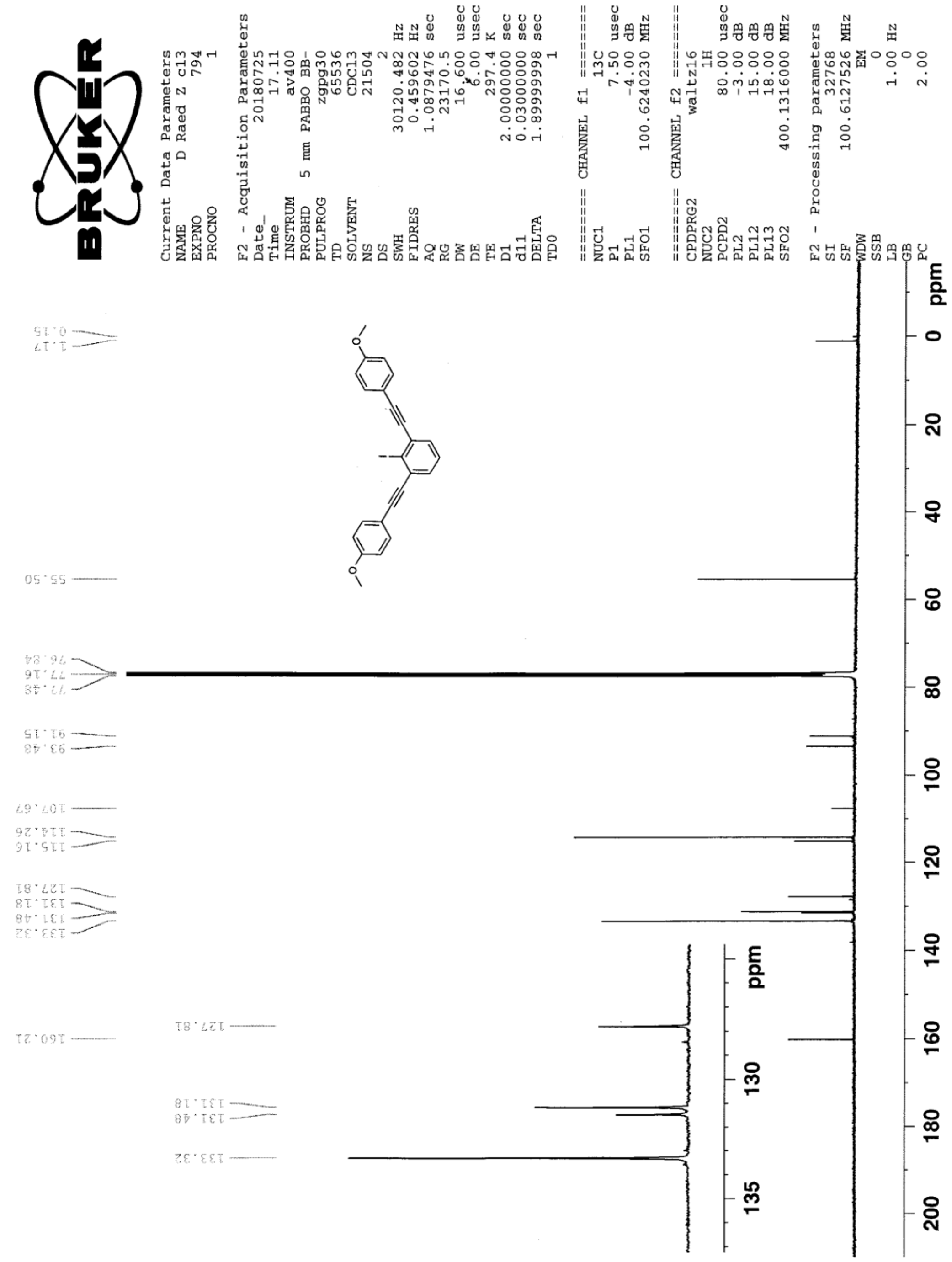

1.5.53 ^{1}H-NMR of 4,4'-((2-iodo-5-methoxy-1,3-phenylene)bis(ethyne-2,1-diyl))bis(methoxybenzene) (31_A) in *$CDCl_3$* at 25 °C.

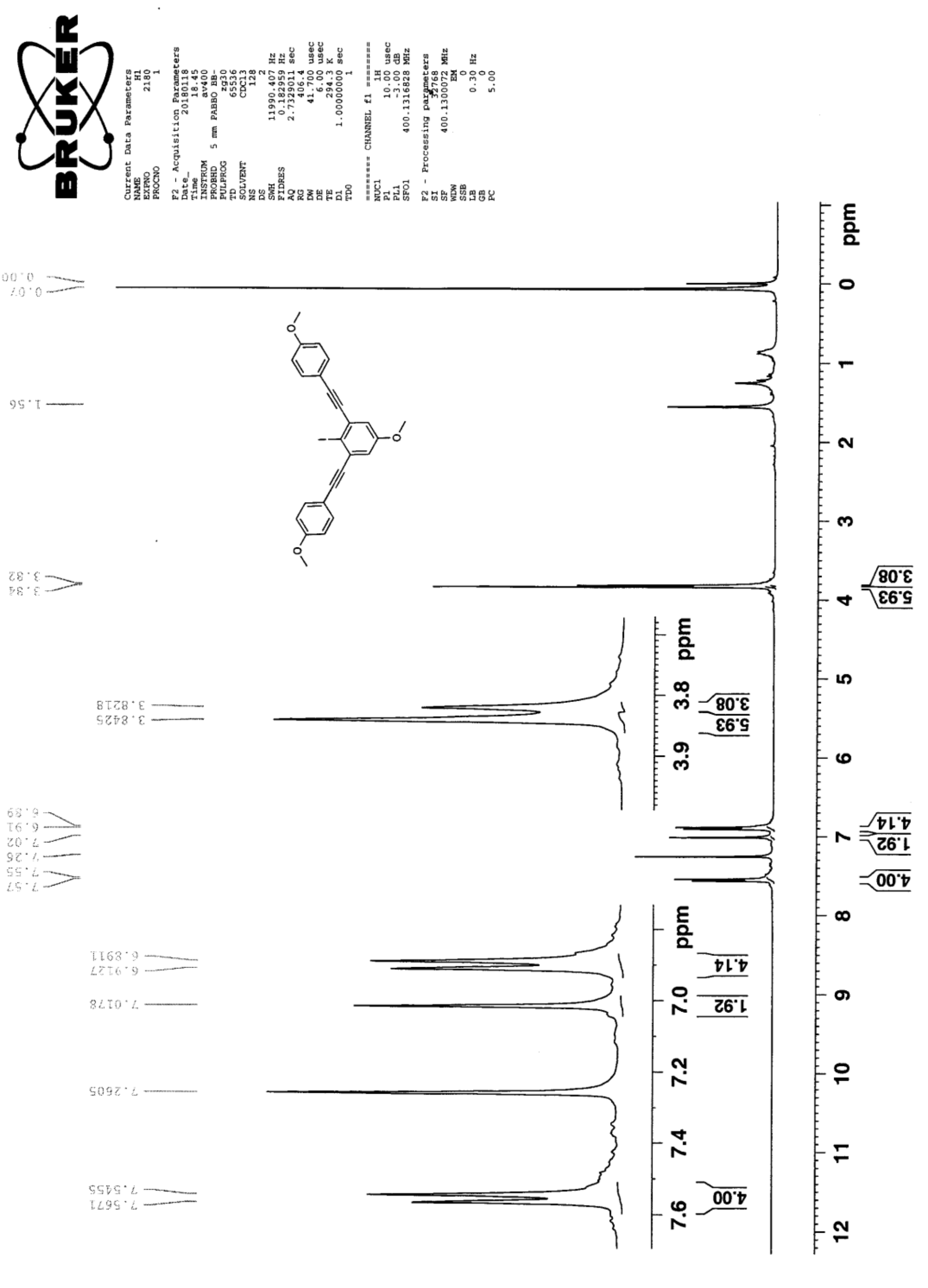

1.5.54 ^{13}C-NMR of 4,4'-((2-iodo-5-methoxy-1,3-phenylene)bis(ethyne-2,1-diyl))bis(methoxybenzene) (31_A) in $CDCl_3$ at 25 °C.

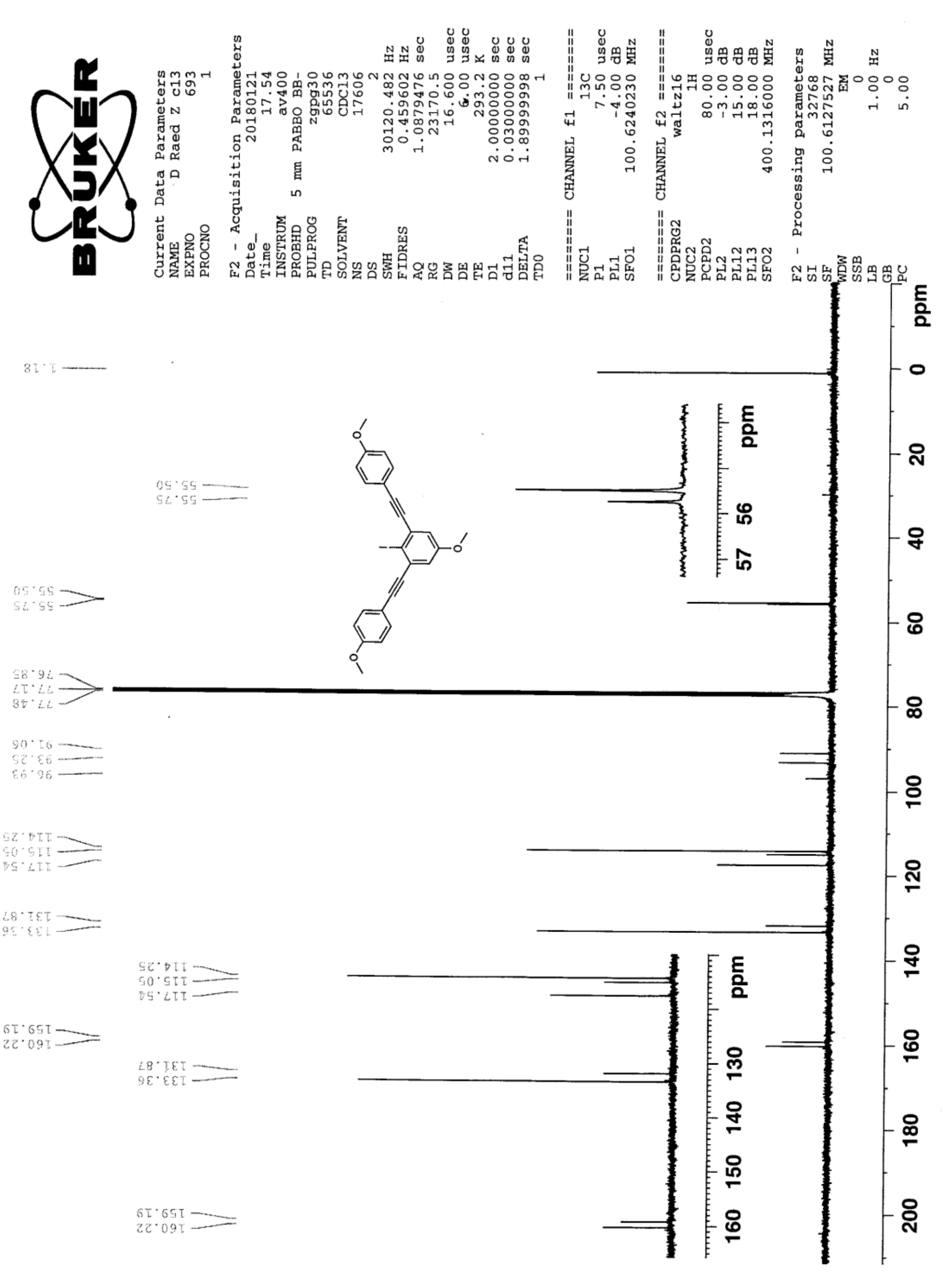

1.5.55 ^{1}H-NMR of 3,3'-((5-bromo-2-iodo-1,3-phenylene)bis(ethyne-2,1-diyl))bis(fluorobenzene) (32_A) in *$CDCl_3$* at 25 °C.

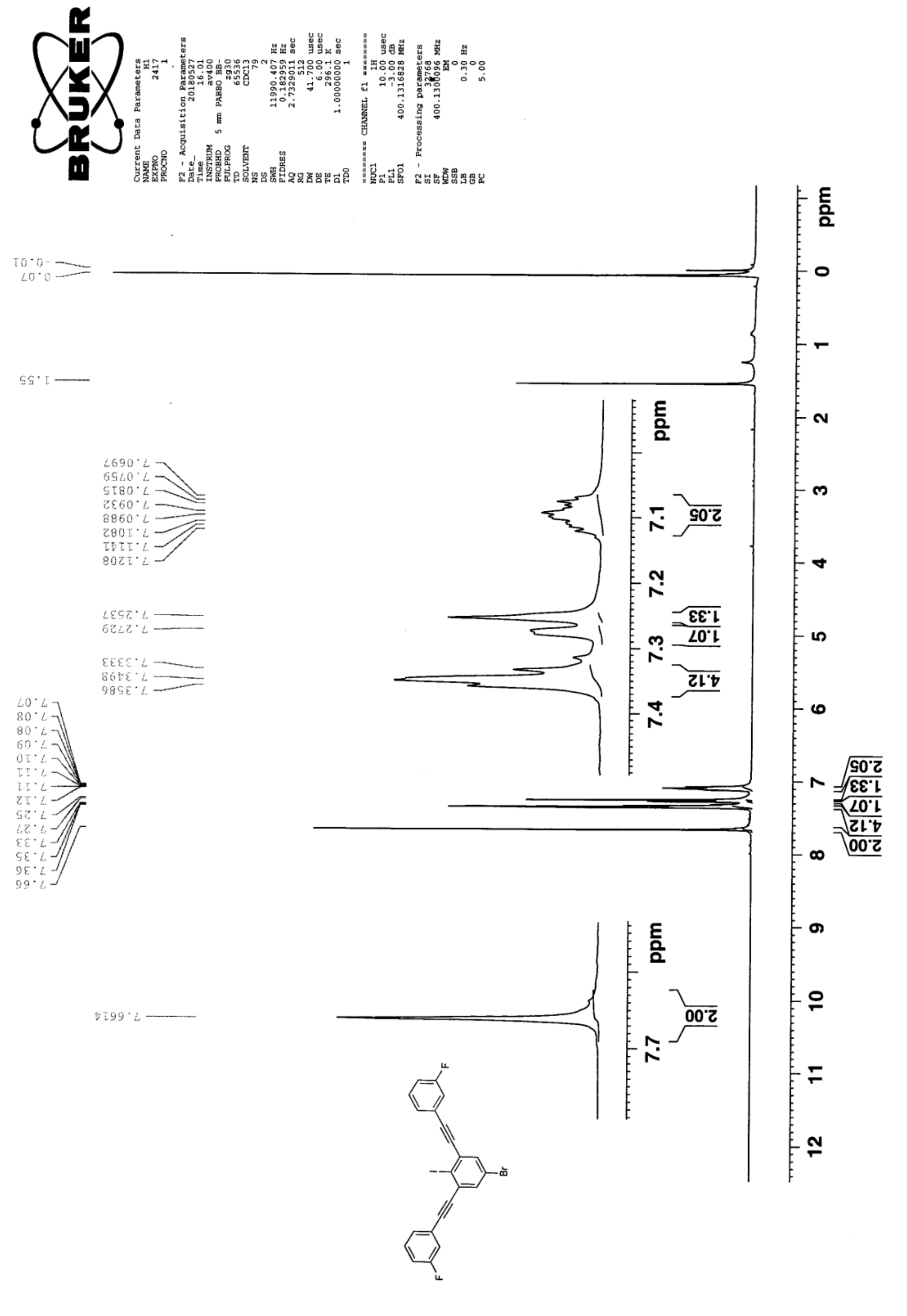

1.5.56 ^{13}C-NMR of 3,3'-((5-bromo-2-iodo-1,3-phenylene)bis(ethyne-2,1-diyl))bis(fluorobenzene) (32_A) in $CDCl_3$ at 25 °C.

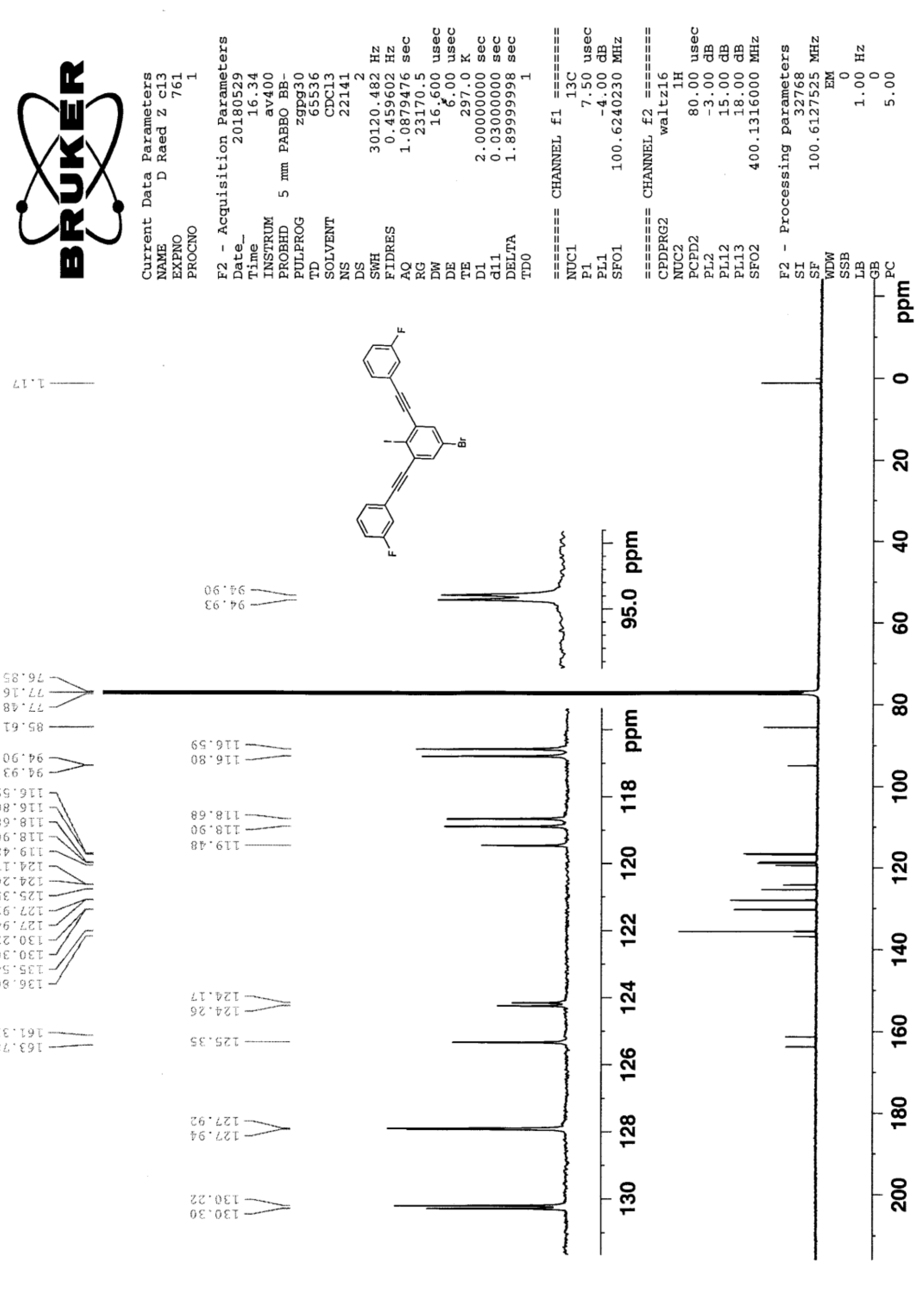

R. M. Al-Zoubi, M. K. Al-Omari, W. K. Al-Jammal and M. J. Ferguson

1.5.57 ^{1}H-NMR of 4,4'-((5-bromo-2-iodo-1,3-phenylene)bis(ethyne-2,1-diyl)) bis(ethylbenzene) (33_A) in *$CDCl_3$* at 25 ^{o}C.

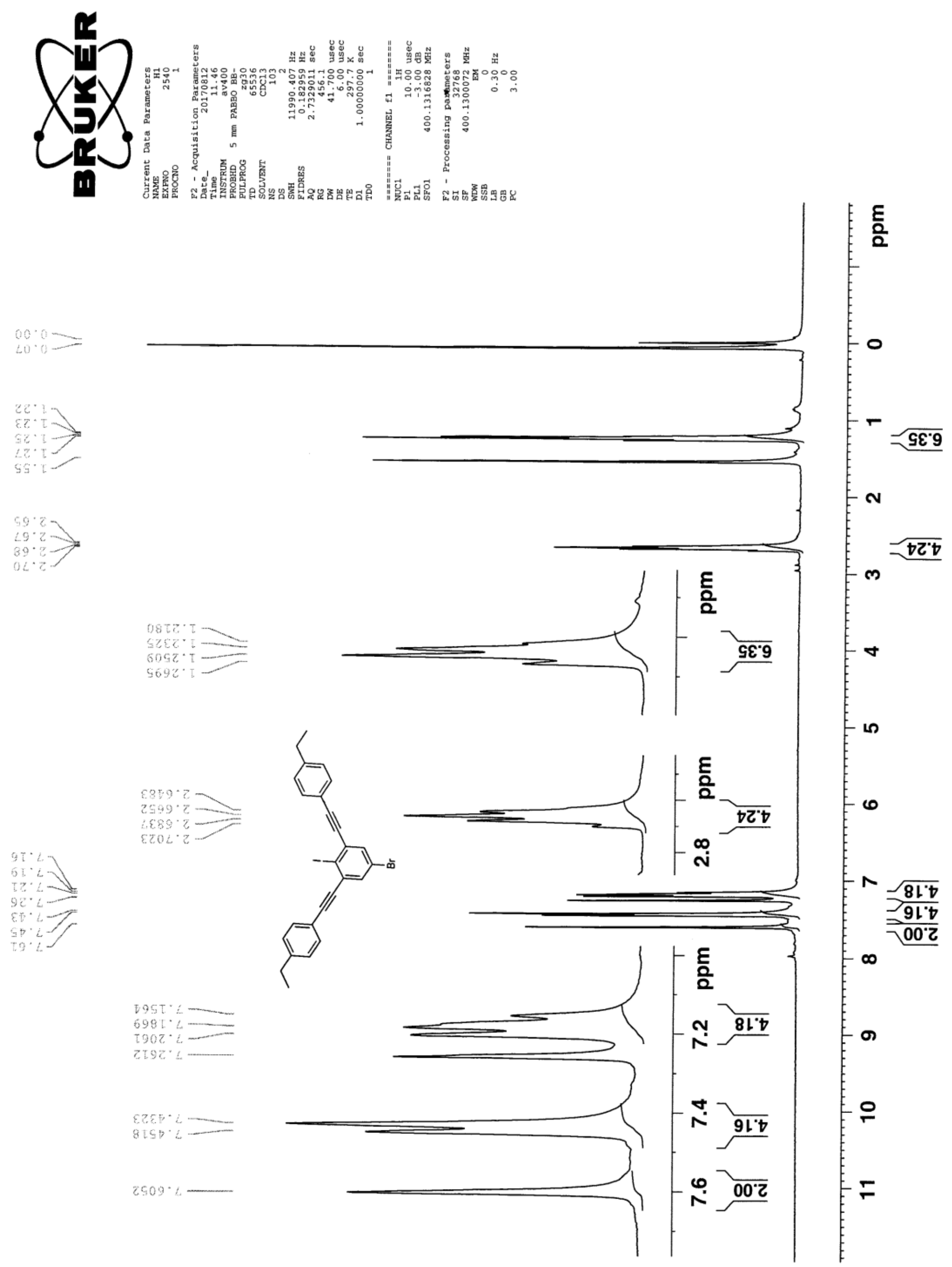

1.5.58 ^{13}C-NMR of 4,4'-((5-bromo-2-iodo-1,3-phenylene)bis(ethyne-2,1-diyl)) bis(ethylbenzene) (33A) in *$CDCl_3$* at 25 °C.

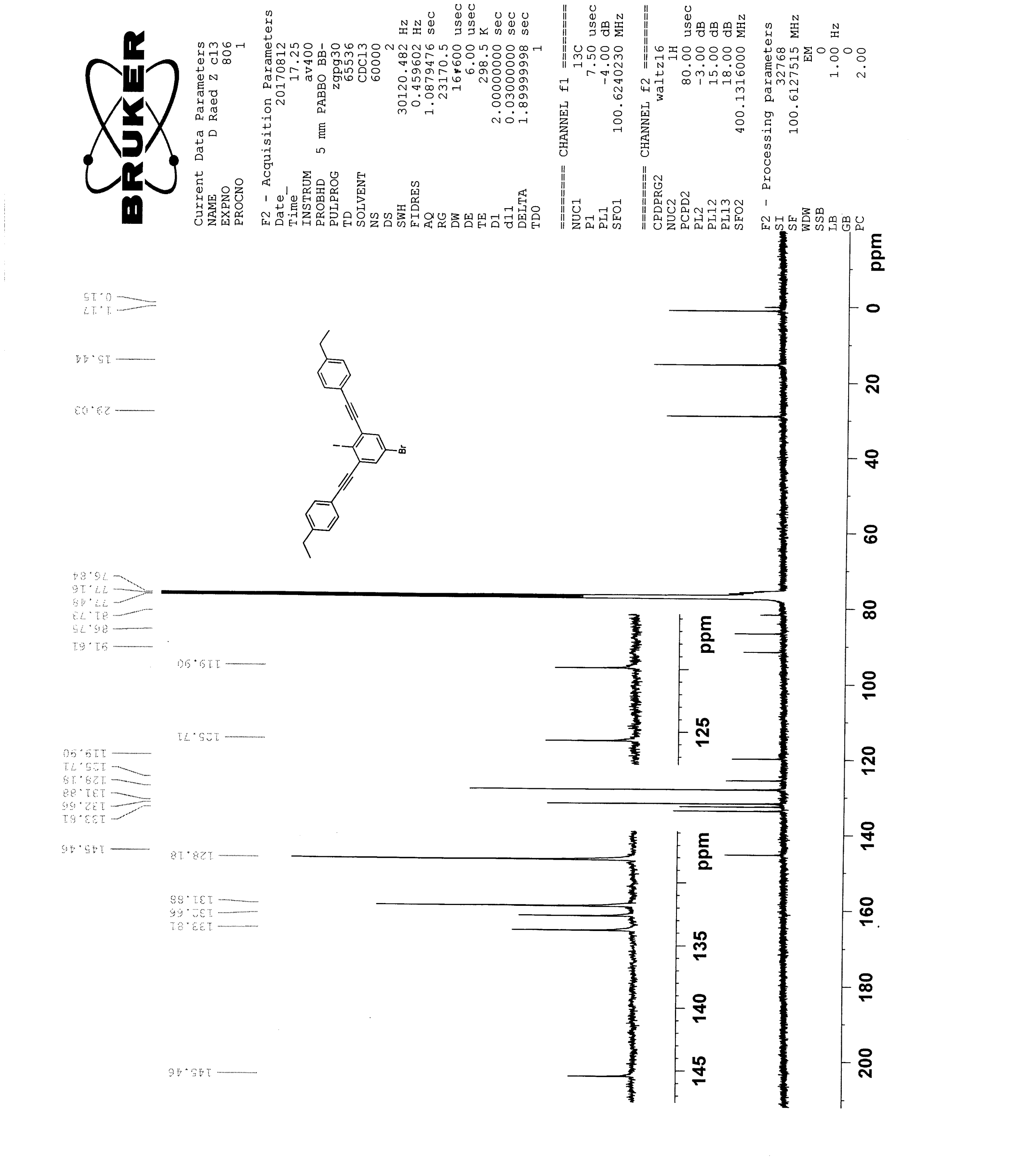

1.5.59 ¹H-NMR of 4,4'-((2-iodo-1,3-phenylene)bis(ethyne-2,1-diyl))bis((trifluoromethyl)benzene) (34_A) in CDCl3 at 25 °C.

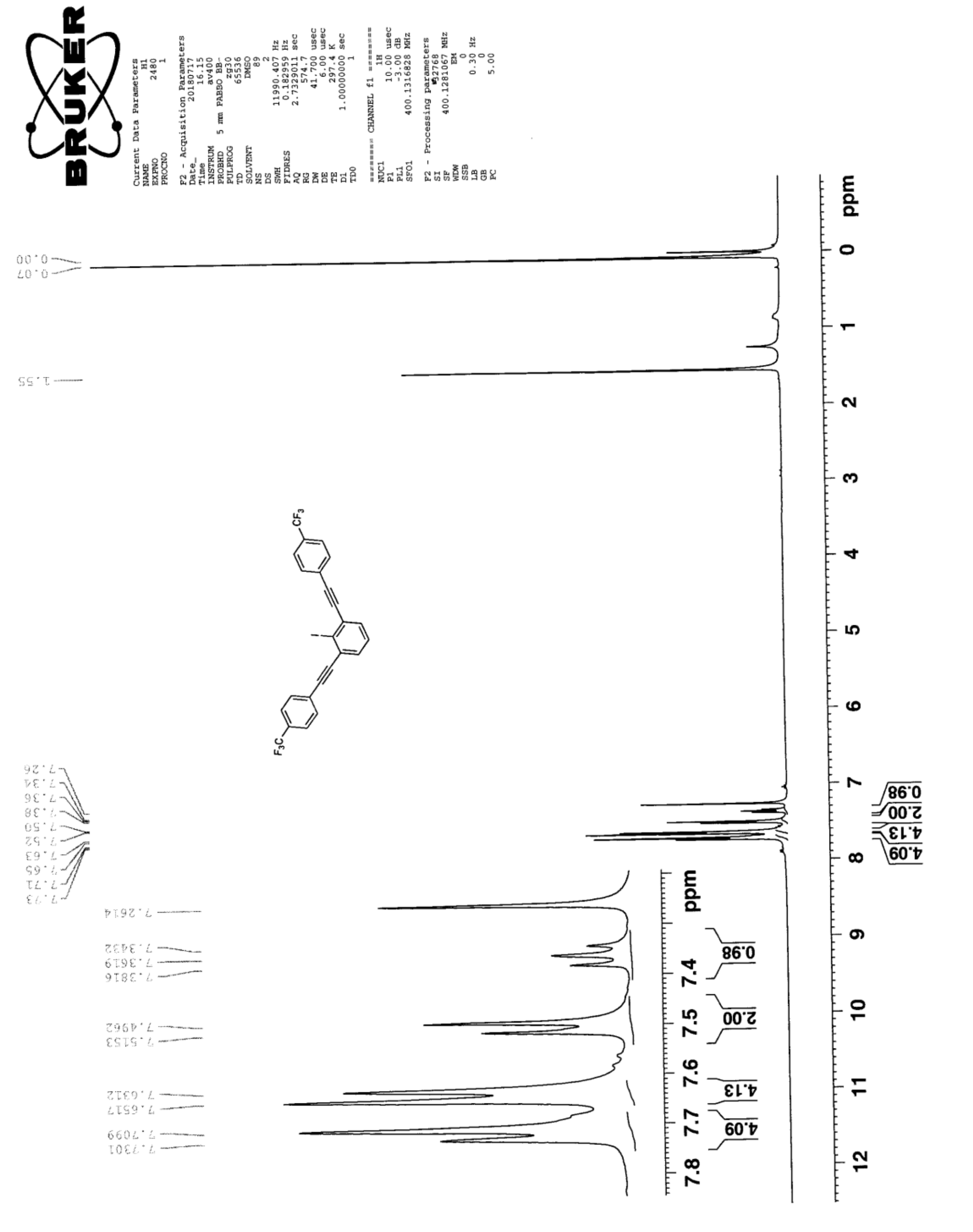

1.5.60 ^{13}C-NMR of 4,4'-((2-iodo-1,3-phenylene)bis(ethyne-2,1-diyl))bis((trifluoromethyl)benzene) (34$_A$) in *CDCl*$_3$ at 25 ^{o}C.

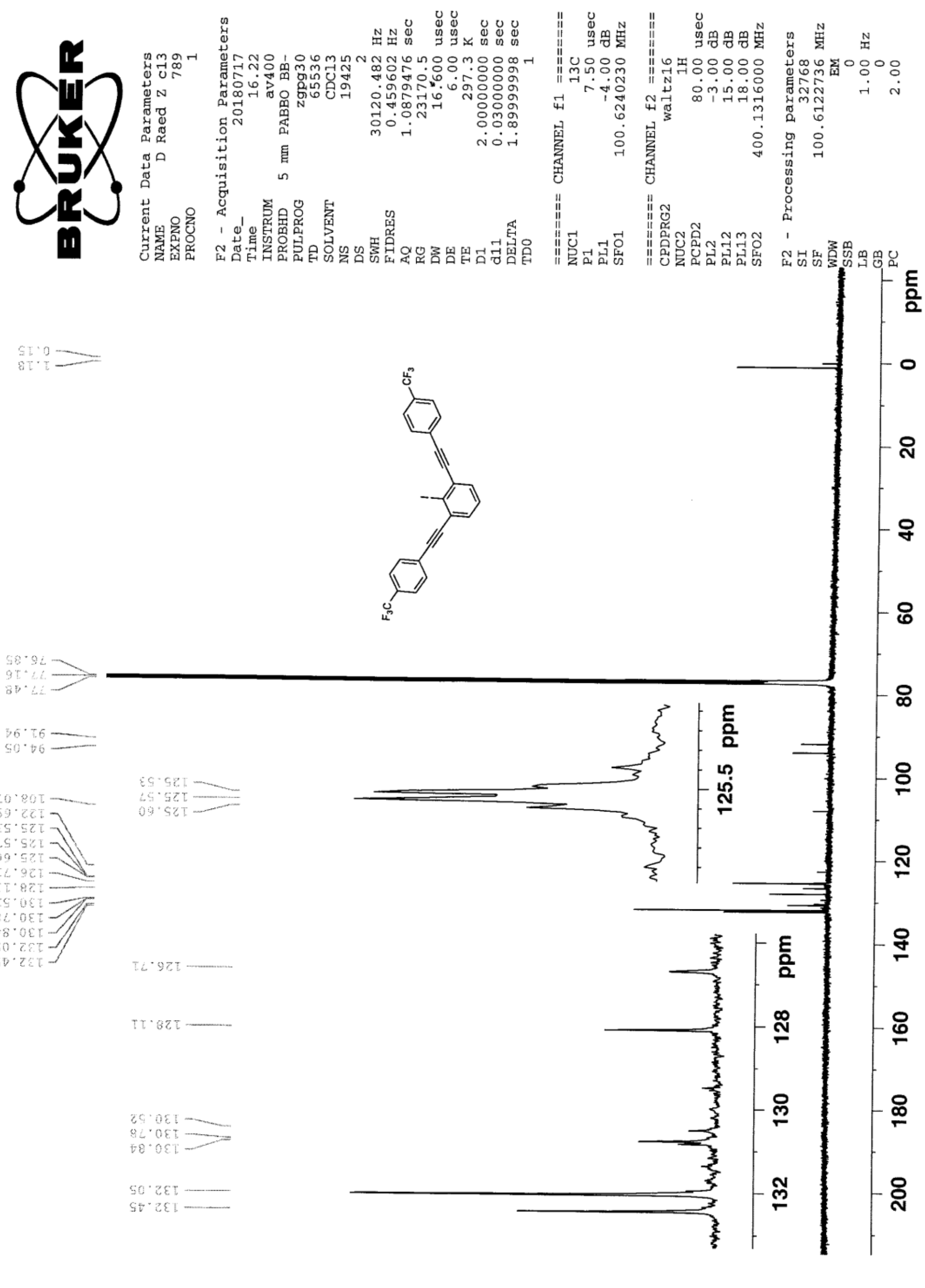

1.5.61 ^{1}H-NMR of 4,4'-((5-chloro-2-iodo-1,3-phenylene)bis(ethyne-2,1-diyl))bis((trifluoromethyl)benzene) (35$_A$) in *$CDCl_3$* at 25 ^oC.

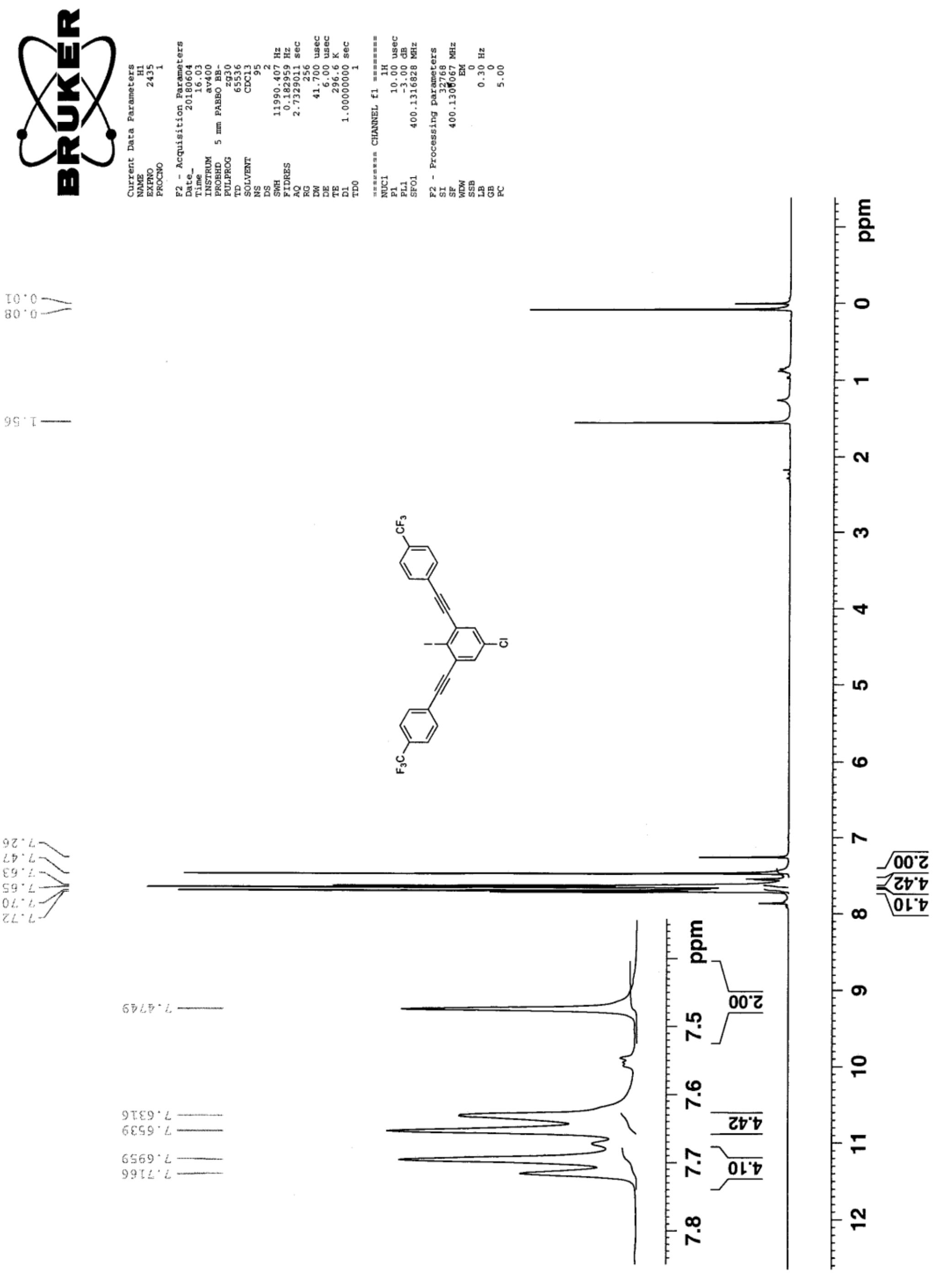

1.5.62 ^{13}C-NMR of 4,4'-((5-chloro-2-iodo-1,3-phenylene)bis(ethyne-2,1-diyl))bis((trifluoromethyl)benzene) (35_A) in *$CDCl_3$* at 25 °C.

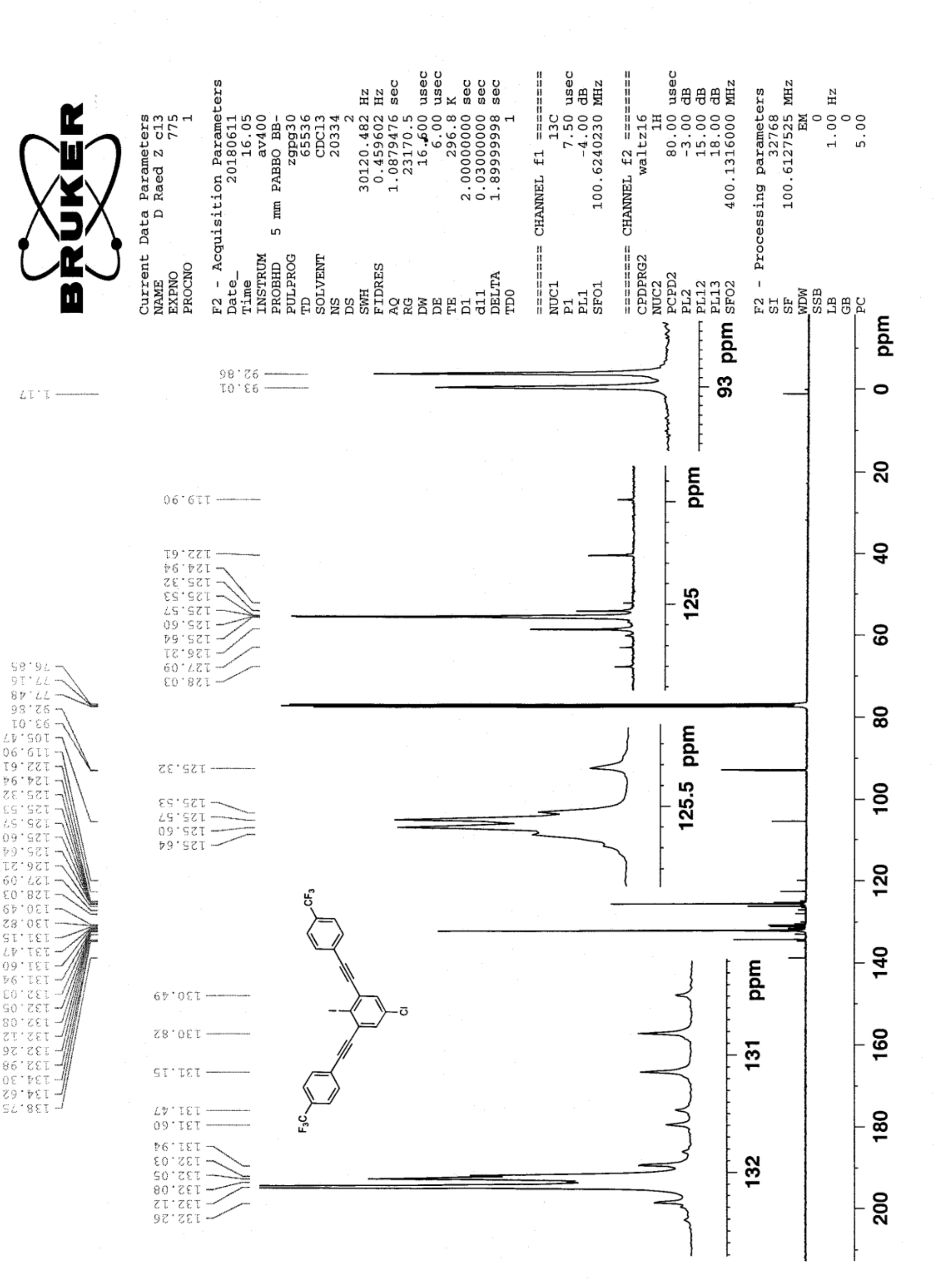

R. M. Al-Zoubi, M. K. Al-Omari, W. K. Al-Jammal and M. J. Ferguson

1.5.63 ^{1}H-NMR of 4,4''-dimethoxy-3'-(phenylethynyl)-1,1':2',1''-terphenyl (36) in *CDCl*$_3$ at 25 ^{o}C.

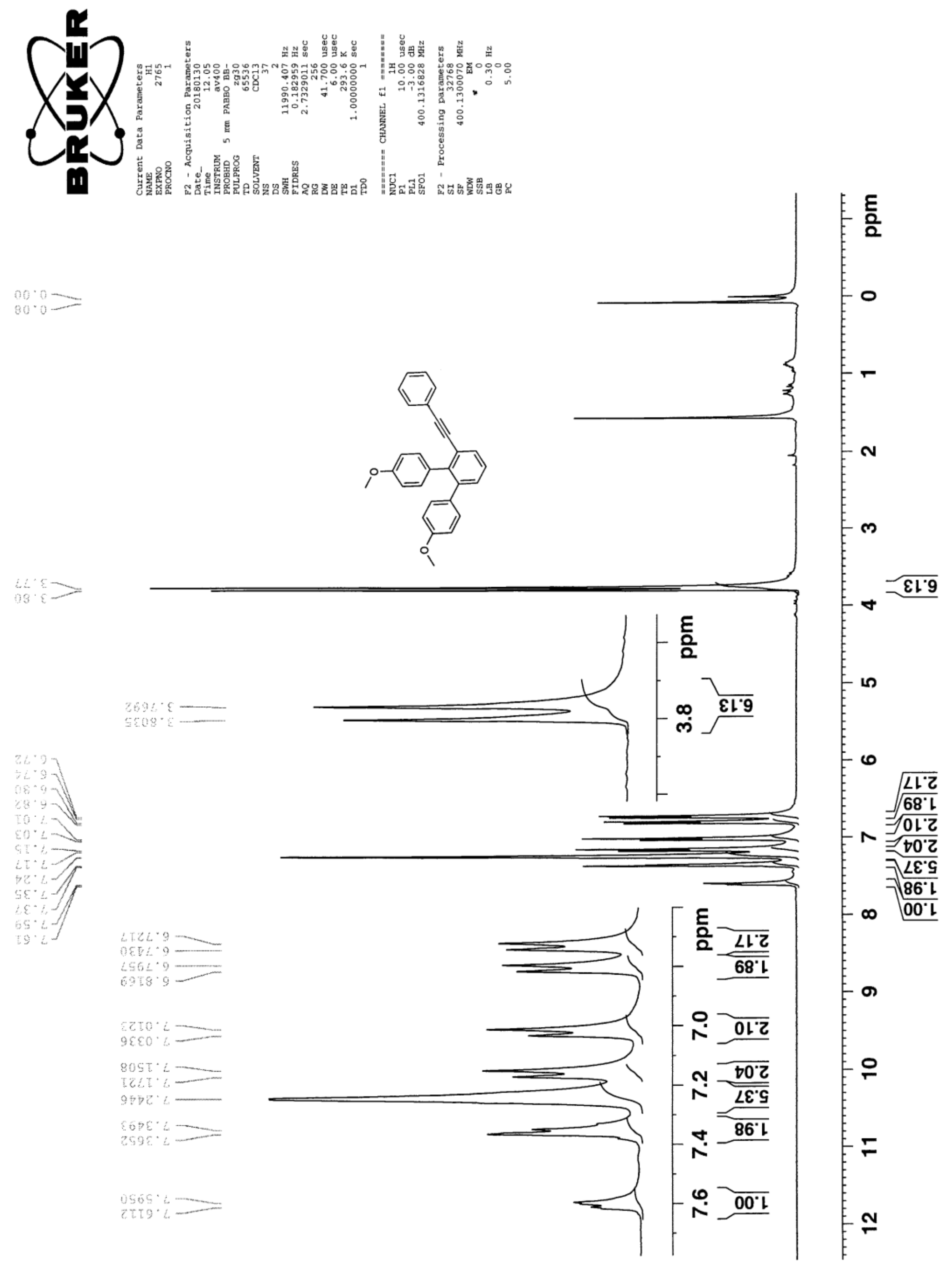

1.5.64 ^{13}C-NMR of 4,4''-dimethoxy-3'-(phenylethynyl)-1,1':2',1''-terphenyl (36) in *$CDCl_3$* at 25 ^{o}C.

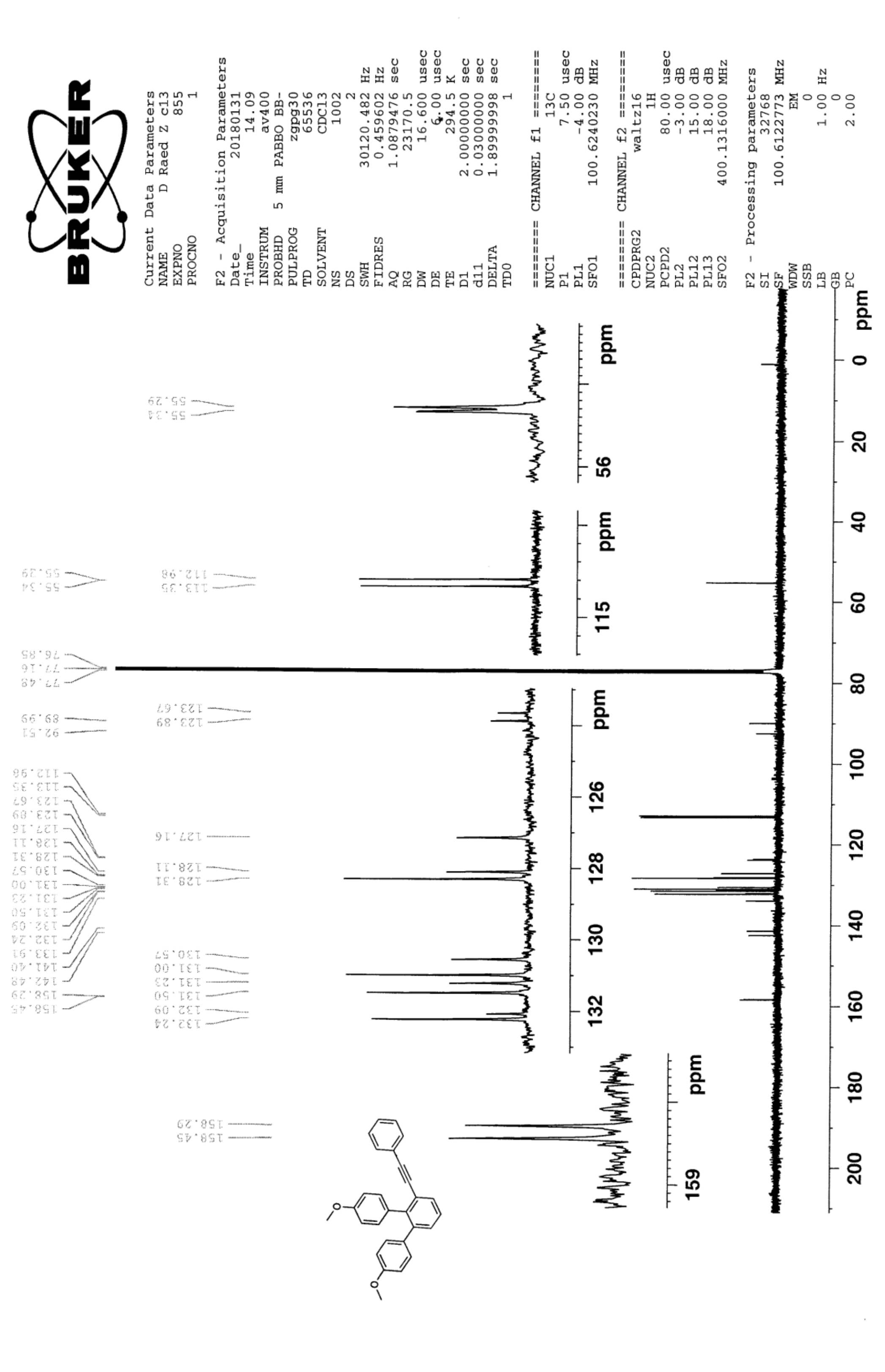

1.5.65 ^{1}H-NMR of 3'-(phenylethynyl)-4,4''-bis(trifluoromethyl)-1,1':2',1''-terphenyl (37) in *CDCl$_3$* at 25 ^{o}C.

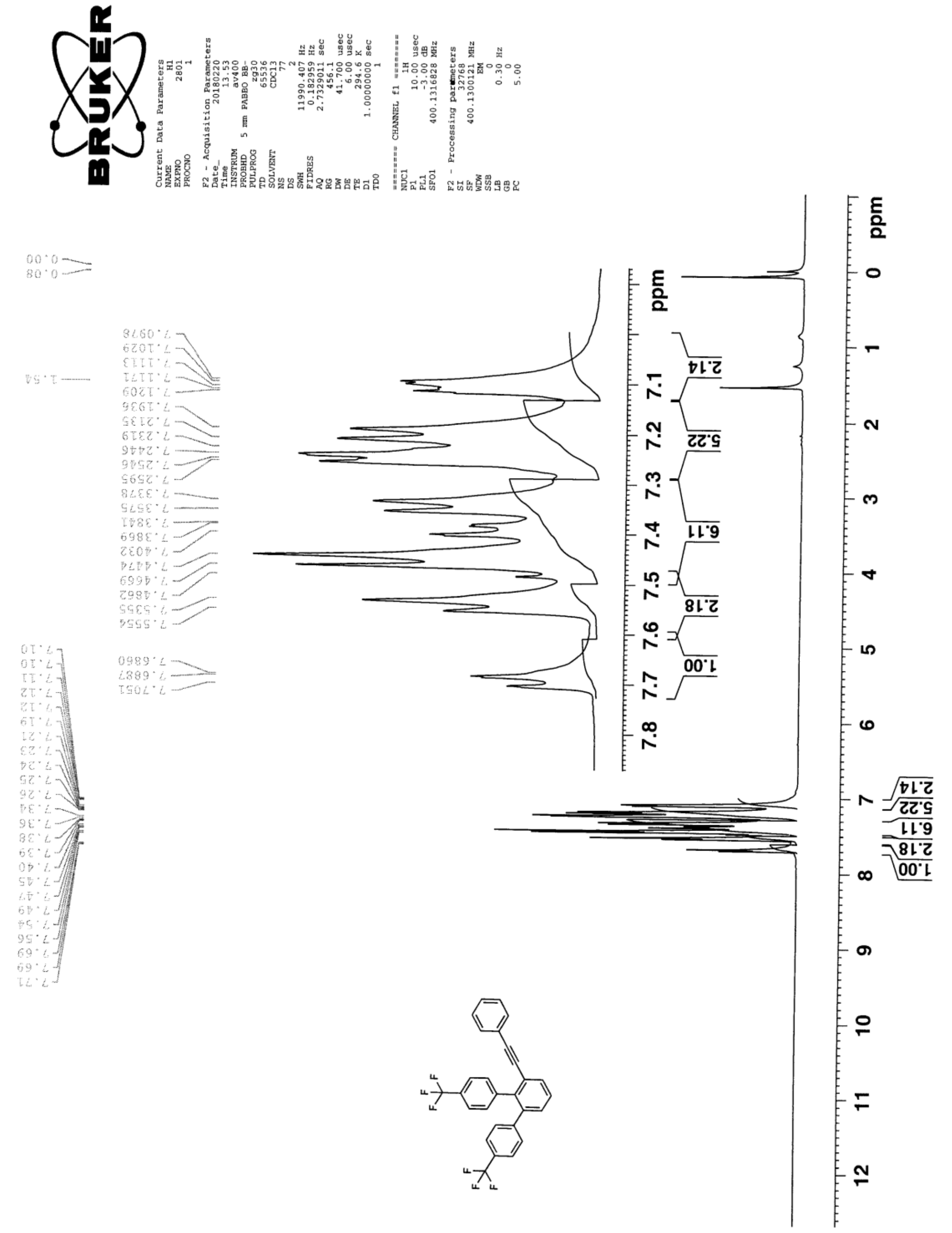

1.5.66 ^{13}C-NMR of 3'-(phenylethynyl)-4,4''-bis(trifluoromethyl)-1,1':2',1''-terphenyl (37) in *CDCl$_3$* at 25 °C.

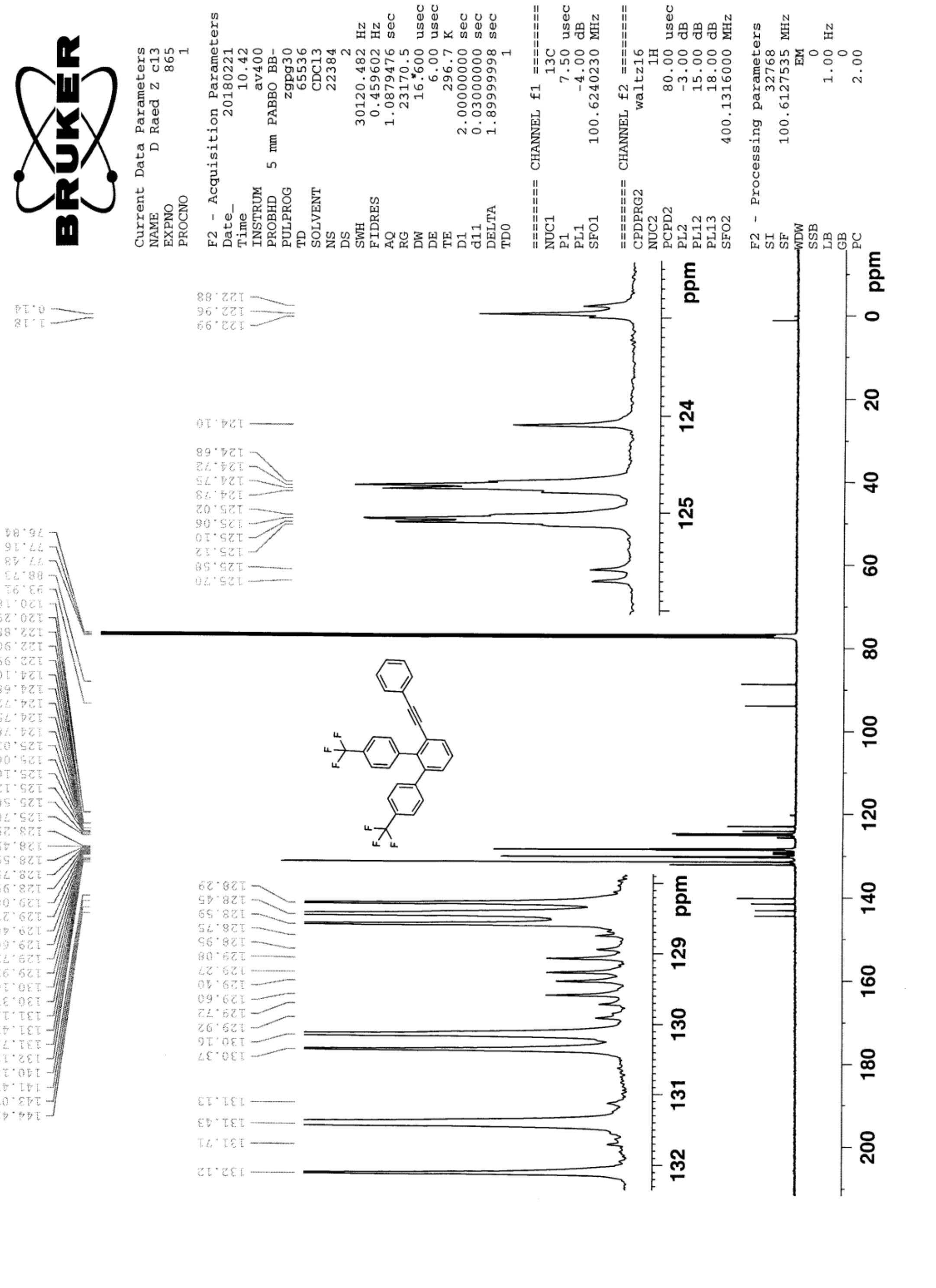

1.6.1 X-ray data of 1,2-diiodo-3-{[4-(trifluoromethyl)phenyl]ethynyl}benzene (6_A)

STRUCTURE REPORT

XCL Code: JUS1808 **Date:** 6 December 2018

Compound: 1,2-diiodo-3-{[4-(trifluoromethyl)phenyl]ethynyl}benzene
Formula: $C_{15}H_7F_3I_2$

Supervisor: R. Al-Zoubi, Jordan University of Science and Technology

Crystallographer: M. J. Ferguson

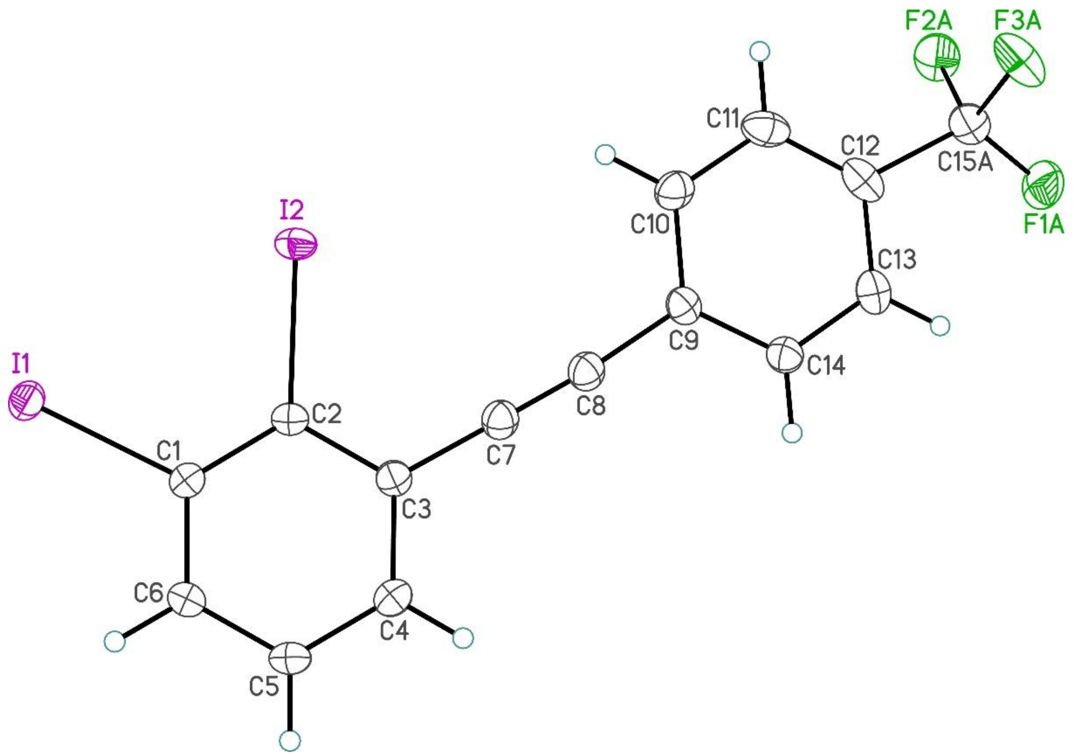

Figure Legends

Figure 1. Perspective view of the 1,2-diiodo-3-{[4-(trifluoromethyl)phenyl]ethynyl}benzene molecule showing the atom labelling scheme. Non-hydrogen atoms are represented by Gaussian ellipsoids at the 30% probability level. Only one orientation of the disordered trifluoromethyl group is shown. Hydrogen atoms are shown with arbitrarily small thermal parameters.

Figure 2. Same view of the molecule showing the alternate orientation of the disordered trifluoromethyl group.

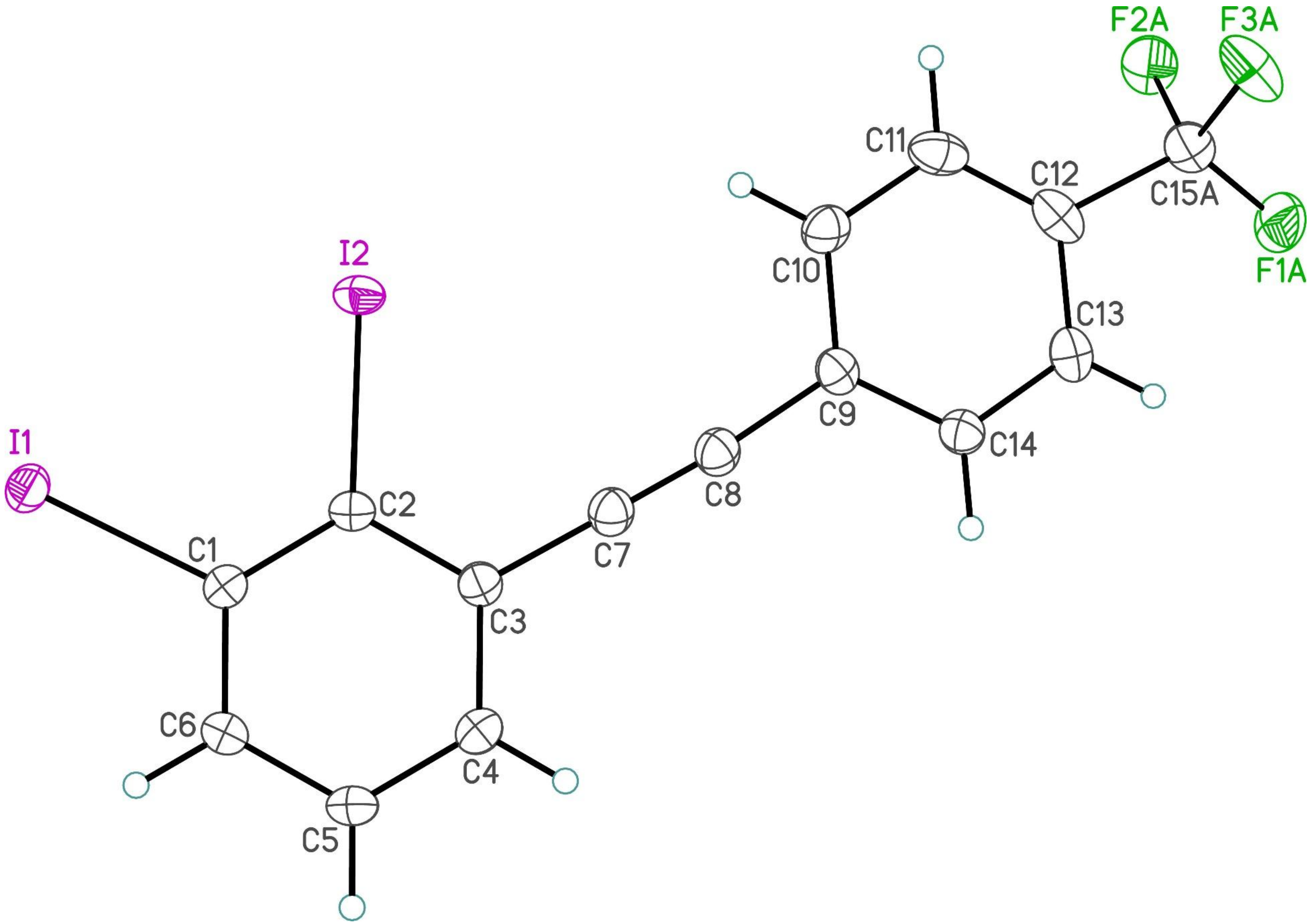
F2A
F3A
C11
C12
C15A
F1A
C10
I2
C13
C9
C14
I1
C8
C2
C7
C1
C3
C6
C4
C5

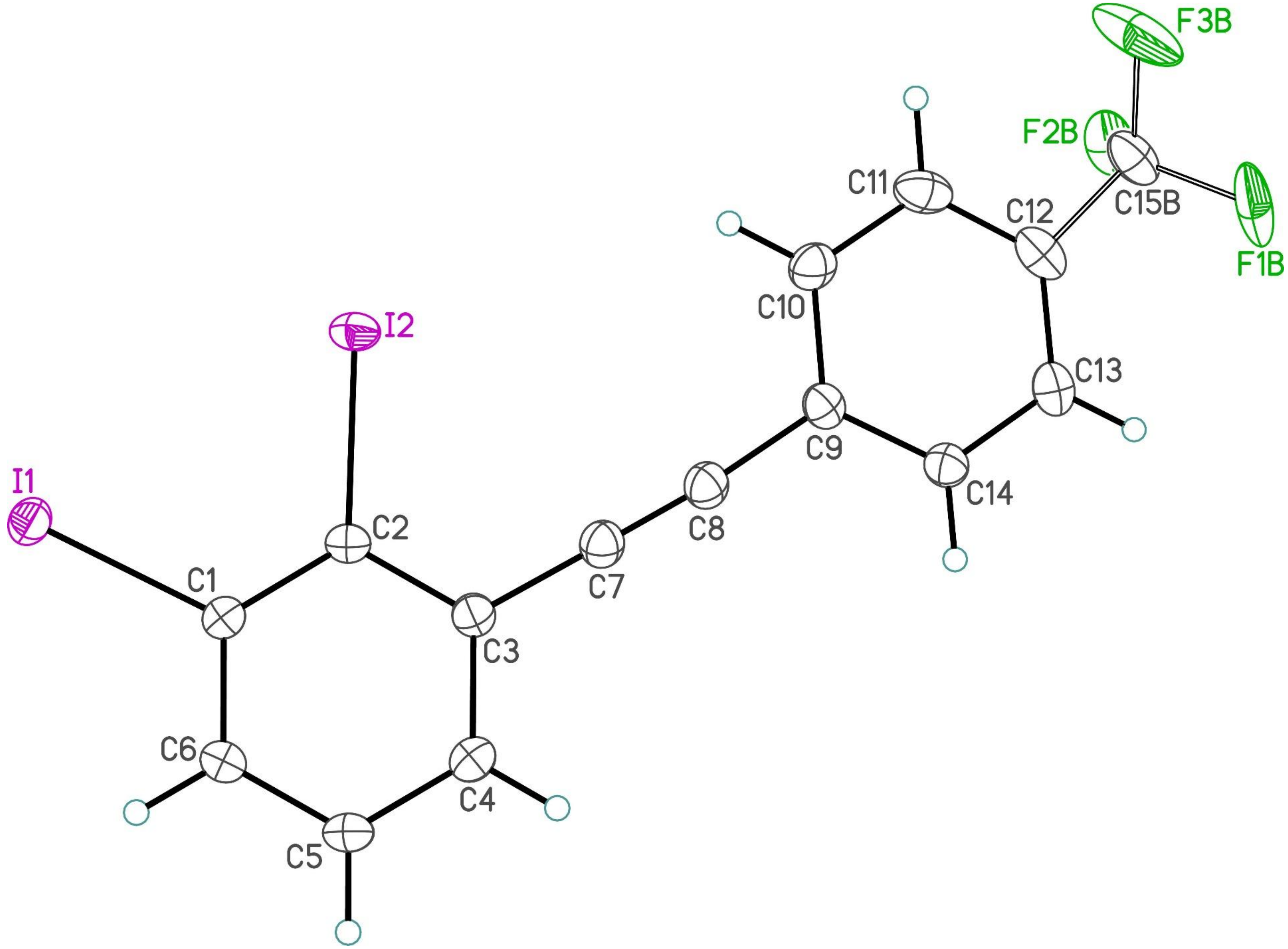
F3B
F2B
C11
C12
C15B
F1B
C10
I2
C13
C9
I1
C14
C8
C2
C1
C7
C3
C6
C4
C5

List of Tables

Table 1. Crystallographic Experimental Details

Table 2. Atomic Coordinates and Equivalent Isotropic Displacement Parameters

Table 3. Selected Interatomic Distances

Table 4. Selected Interatomic Angles

Table 5. Torsional Angles

Table 6. Anisotropic Displacement Parameters

Table 7. Derived Atomic Coordinates and Displacement Parameters for Hydrogen Atoms

Table 1. Crystallographic Experimental Details

A. Crystal Data	
formula	$C_{15}H_7F_3I_2$
formula weight	498.01
crystal dimensions (mm)	0.19 × 0.18 × 0.12
crystal system	monoclinic
space group	$P2_1/c$ (No. 14)
unit cell parameters[a]	
a (Å)	12.1687(8)
b (Å)	4.3193(3)
c (Å)	27.7962(17)
β (deg)	90.1567(9)
V (Å^3)	1460.97(17)
Z	4
ρ_{calcd} (g cm^{-3})	2.264
μ (mm^{-1})	4.324
B. Data Collection and Refinement Conditions	
diffractometer	Bruker D8/APEX II CCD[b]
radiation (λ [Å])	graphite-monochromated Mo Kα (0.71073)
temperature (°C)	–100
scan type	ω scans (0.3°) (10 s exposures)
data collection 2θ limit (deg)	61.06
total data collected	16567 ($-17 \le h \le 17$, $-6 \le k \le 6$, $-39 \le l \le 39$)
independent reflections	4473 (R_{int} = 0.0171)
number of observed reflections (*NO*)	4127 [$F_o^2 \ge 2\sigma(F_o^2)$]
structure solution method	intrinsic phasing (*SHELXT-2014*[c])
refinement method	full-matrix least-squares on F^2 (*SHELXL–2017*[d])
absorption correction method	Gaussian integration (face-indexed)
range of transmission factors	0.3623–0.2634
data/restraints/parameters	4473 / 0 / 218
goodness-of-fit (S)[e] [all data]	1.161
final R indices[f]	
R_1 [$F_o^2 \ge 2\sigma(F_o^2)$]	0.0250
wR_2 [all data]	0.0575
largest difference peak and hole	1.059 and –0.795 e Å^{-3}

[a]Obtained from least-squares refinement of 9886 reflections with 4.44° < 2θ < 61.02°.

[b]Programs for diffractometer operation, data collection, data reduction and absorption correction were those supplied by Bruker.

(continued)

Table 1. Crystallographic Experimental Details (continued)

[c]Sheldrick, G. M. *Acta Crystallogr.* **2015**, *A71*, 3–8. (*SHELXT-2014*)

[d]Sheldrick, G. M. *Acta Crystallogr.* **2015**, *C71*, 3–8. (*SHELXL-2017*)

[e]$S = [\Sigma w(F_o^2 - F_c^2)^2/(n - p)]^{1/2}$ (n = number of data; p = number of parameters varied; $w = [\sigma^2(F_o^2) + (0.0241P)^2 + 1.1974P]^{-1}$ where $P = [\mathrm{Max}(F_o^2, 0) + 2F_c^2]/3$).

[f]$R_1 = \Sigma||F_o| - |F_c||/\Sigma|F_o|$; $wR_2 = [\Sigma w(F_o^2 - F_c^2)^2/\Sigma w(F_o^4)]^{1/2}$.

Table 2. Atomic Coordinates and Equivalent Isotropic Displacement Parameters

Atom	*x*	*y*	*z*	U_{eq}, Å^2
I1	0.57436(2)	0.08974(4)	0.92983(2)	0.03552(5)*
I2	0.47038(2)	0.04206(4)	0.80711(2)	0.03373(5)*
F1A[a]	-0.0176(7)	0.5282(19)	0.5557(3)	0.063(2)*
F2A[a]	0.1456(7)	0.381(2)	0.5361(3)	0.061(2)*
F3A[a]	0.0208(6)	0.0502(14)	0.55431(17)	0.069(2)*
F1B[b]	-0.0044(7)	0.367(3)	0.5521(3)	0.082(4)*
F2B[b]	0.1590(7)	0.523(2)	0.5416(3)	0.0587(19)*
F3B[b]	0.1261(10)	0.0419(14)	0.54445(18)	0.105(4)*
C1	0.42064(19)	0.2673(5)	0.90982(8)	0.0272(4)*
C2	0.38129(19)	0.2496(5)	0.86277(8)	0.0264(4)*
C3	0.2778(2)	0.3791(6)	0.85183(9)	0.0304(5)*
C4	0.2176(2)	0.5249(7)	0.88824(10)	0.0377(6)*
C5	0.2580(2)	0.5385(7)	0.93474(10)	0.0407(6)*
C6	0.3593(2)	0.4096(6)	0.94558(9)	0.0350(5)*
C7	0.2338(2)	0.3693(7)	0.80382(10)	0.0357(5)*
C8	0.1986(2)	0.3626(7)	0.76386(10)	0.0358(5)*
C9	0.1649(2)	0.3476(6)	0.71426(9)	0.0319(5)*
C10	0.2283(2)	0.1774(7)	0.68185(10)	0.0391(6)*
C11	0.1984(3)	0.1700(7)	0.63341(11)	0.0447(7)*
C12	0.1068(3)	0.3282(7)	0.61760(9)	0.0411(6)*
C13	0.0424(2)	0.4936(7)	0.64961(10)	0.0397(6)*
C14	0.0715(2)	0.5014(7)	0.69783(10)	0.0359(5)*
C15A	0.0590(9)	0.329(4)	0.5657(6)	0.042(2)*
C15B	0.0989(12)	0.316(4)	0.5639(5)	0.047(3)*

Anisotropically-refined atoms are marked with an asterisk (*). The form of the anisotropic displacement parameter is: $\exp[-2\pi^2(h^2a^{*2}U_{11} + k^2b^{*2}U_{22} + l^2c^{*2}U_{33} + 2klb^*c^*U_{23} + 2hla^*c^*U_{13} + 2hka^*b^*U_{12})]$. Refined occupancies of 0.502(10)[a] and 0.498(10)[b].

Table 3. Selected Interatomic Distances (Å)

Atom1	Atom2	Distance	Atom1	Atom2	Distance
I1	C1	2.096(2)	C4	C5	1.383(4)
I2	C2	2.093(2)	C5	C6	1.385(4)
F1A	C15A	1.299(15)	C7	C8	1.190(4)
F2A	C15A	1.357(12)	C8	C9	1.439(4)
F3A	C15A	1.328(17)	C9	C10	1.397(4)
F1B	C15B	1.317(14)	C9	C14	1.392(4)
F2B	C15B	1.311(15)	C10	C11	1.394(4)
F3B	C15B	1.344(18)	C11	C12	1.378(5)
C1	C2	1.394(3)	C12	C13	1.386(4)
C1	C6	1.388(3)	C12	C15A	1.554(16)
C2	C3	1.410(3)	C12	C15B	1.497(15)
C3	C4	1.401(4)	C13	C14	1.386(4)
C3	C7	1.437(4)			

Table 4. Selected Interatomic Angles (deg)

Atom1	Atom2	Atom3	Angle	Atom1	Atom2	Atom3	Angle
I1	C1	C2	122.21(17)	C11	C12	C13	120.6(3)
I1	C1	C6	116.97(18)	C11	C12	C15A	126.6(6)
C2	C1	C6	120.8(2)	C11	C12	C15B	110.5(7)
I2	C2	C1	122.68(17)	C13	C12	C15A	112.6(6)
I2	C2	C3	118.32(17)	C13	C12	C15B	128.6(7)
C1	C2	C3	119.0(2)	C12	C13	C14	119.3(3)
C2	C3	C4	119.4(2)	C9	C14	C13	120.8(3)
C2	C3	C7	121.3(2)	F1A	C15A	F2A	108.6(13)
C4	C3	C7	119.3(2)	F1A	C15A	F3A	107.4(10)
C3	C4	C5	120.6(2)	F1A	C15A	C12	117.8(11)
C4	C5	C6	120.0(2)	F2A	C15A	F3A	106.2(11)
C1	C6	C5	120.2(2)	F2A	C15A	C12	105.8(8)
C3	C7	C8	179.2(3)	F3A	C15A	C12	110.5(11)
C7	C8	C9	175.3(3)	F1B	C15B	F2B	107.6(14)
C8	C9	C10	119.0(2)	F1B	C15B	F3B	106.6(13)
C8	C9	C14	121.5(2)	F1B	C15B	C12	107.5(9)
C10	C9	C14	119.4(2)	F2B	C15B	F3B	105.8(10)
C9	C10	C11	119.5(3)	F2B	C15B	C12	114.3(11)
C10	C11	C12	120.3(3)	F3B	C15B	C12	114.6(13)

Table 5. Torsional Angles (deg)

Atom1	Atom2	Atom3	Atom4	Angle	Atom1	Atom2	Atom3	Atom4	Angle
I1	C1	C2	I2	0.4(3)	C10	C11	C12	C13	-0.8(4)
I1	C1	C2	C3	-178.90(17)	C10	C11	C12	C15A	-176.4(8)
C6	C1	C2	I2	179.39(19)	C10	C11	C12	C15B	173.5(7)
C6	C1	C2	C3	0.1(4)	C11	C12	C13	C14	0.7(4)
I1	C1	C6	C5	178.5(2)	C15A	C12	C13	C14	176.9(7)
C2	C1	C6	C5	-0.5(4)	C15B	C12	C13	C14	-172.5(8)
I2	C2	C3	C4	-178.7(2)	C11	C12	C15A	F1A	-169.6(8)
I2	C2	C3	C7	0.2(3)	C11	C12	C15A	F2A	-48.1(14)
C1	C2	C3	C4	0.6(4)	C11	C12	C15A	F3A	66.5(10)
C1	C2	C3	C7	179.5(2)	C13	C12	C15A	F1A	14.5(14)
C2	C3	C4	C5	-0.9(4)	C13	C12	C15A	F2A	136.1(9)
C7	C3	C4	C5	-179.9(3)	C13	C12	C15A	F3A	-109.4(8)
C3	C4	C5	C6	0.5(5)	C11	C12	C15B	F1B	157.9(10)
C4	C5	C6	C1	0.2(5)	C11	C12	C15B	F2B	-82.7(14)
C8	C9	C10	C11	-177.9(3)	C11	C12	C15B	F3B	39.7(11)
C14	C9	C10	C11	1.4(4)	C13	C12	C15B	F1B	-28.3(16)
C8	C9	C14	C13	177.7(3)	C13	C12	C15B	F2B	91.0(13)
C10	C9	C14	C13	-1.5(4)	C13	C12	C15B	F3B	-146.6(8)
C9	C10	C11	C12	-0.2(4)	C12	C13	C14	C9	0.5(4)

Table 6. Anisotropic Displacement Parameters (U_{ij}, Å^2)

Atom	U_{11}	U_{22}	U_{33}	U_{23}	U_{13}	U_{12}
I1	0.03233(9)	0.03586(9)	0.03837(9)	0.00274(6)	-0.00175(6)	0.00537(6)
I2	0.04156(10)	0.03051(8)	0.02917(8)	-0.00087(6)	0.00960(6)	0.00302(6)
F1A	0.057(3)	0.093(5)	0.040(3)	0.010(3)	-0.008(2)	0.020(4)
F2A	0.051(3)	0.100(6)	0.033(2)	0.008(4)	0.015(2)	-0.002(4)
F3A	0.088(5)	0.075(4)	0.042(2)	-0.014(2)	-0.009(2)	-0.020(3)
F1B	0.043(4)	0.168(12)	0.035(3)	0.011(5)	-0.011(3)	-0.049(5)
F2B	0.052(3)	0.086(5)	0.037(3)	0.017(3)	0.002(2)	-0.023(3)
F3B	0.198(11)	0.077(4)	0.040(3)	-0.022(2)	-0.003(4)	0.001(4)
C1	0.0269(10)	0.0233(10)	0.0315(11)	0.0025(8)	0.0022(8)	-0.0016(8)
C2	0.0290(11)	0.0226(10)	0.0277(10)	0.0012(8)	0.0059(8)	-0.0032(8)
C3	0.0288(11)	0.0323(12)	0.0300(11)	0.0014(9)	0.0015(9)	-0.0027(9)
C4	0.0293(12)	0.0447(15)	0.0390(14)	-0.0015(11)	0.0017(10)	0.0038(11)
C5	0.0371(14)	0.0513(16)	0.0338(13)	-0.0084(12)	0.0065(11)	0.0077(12)
C6	0.0370(13)	0.0404(14)	0.0276(11)	-0.0031(10)	0.0016(9)	0.0033(11)
C7	0.0315(12)	0.0385(13)	0.0370(13)	0.0012(11)	-0.0004(10)	-0.0014(10)
C8	0.0306(12)	0.0399(14)	0.0369(13)	0.0003(11)	-0.0002(10)	-0.0022(10)
C9	0.0289(11)	0.0353(12)	0.0314(11)	0.0005(10)	0.0005(9)	-0.0083(10)
C10	0.0285(12)	0.0418(14)	0.0472(15)	-0.0033(12)	0.0035(10)	-0.0037(11)
C11	0.0466(16)	0.0444(15)	0.0432(15)	-0.0101(13)	0.0158(12)	-0.0103(13)
C12	0.0528(16)	0.0416(14)	0.0288(12)	0.0011(11)	0.0012(11)	-0.0200(13)
C13	0.0399(14)	0.0426(15)	0.0366(13)	0.0061(11)	-0.0069(11)	-0.0069(12)
C14	0.0349(13)	0.0408(14)	0.0320(12)	-0.0006(10)	0.0011(10)	0.0001(11)
C15A	0.039(6)	0.052(4)	0.034(4)	0.003(3)	0.009(5)	-0.011(6)
C15B	0.056(7)	0.055(5)	0.031(4)	-0.003(3)	0.003(6)	-0.022(7)

The form of the anisotropic displacement parameter is:
$\exp[-2\pi^2(h^2a^{*2}U_{11} + k^2b^{*2}U_{22} + l^2c^{*2}U_{33} + 2klb^*c^*U_{23} + 2hla^*c^*U_{13} + 2hka^*b^*U_{12})]$

Table 7. Derived Atomic Coordinates and Displacement Parameters for Hydrogen Atoms

Atom	x	y	z	U_{eq}, Å^2
H4	0.148358	0.615176	0.880922	0.045
H5	0.216279	0.636256	0.959251	0.049
H6	0.386839	0.418613	0.977558	0.042
H10	0.291283	0.067694	0.692721	0.047
H11	0.241337	0.055183	0.611187	0.054
H13	-0.021146	0.600429	0.638620	0.048
H14	0.027300	0.613203	0.719944	0.043

1.6.2 X-ray data of 5-chloro-1,2-diiodo-3-{[4-(trifluoromethyl)phenyl]ethynyl} benzene (20_A)

STRUCTURE REPORT

XCL Code: JUS1810 **Date:** 3 December 2018

Compound: 5-chloro-1,2-diiodo-3-{[4-(trifluoromethyl)phenyl]ethynyl}benzene
Formula: $C_{15}H_6ClF_3I_2$

Supervisor: R. Al-Zoubi, Jordan University of Science and Technology

Crystallographer: M. J. Ferguson

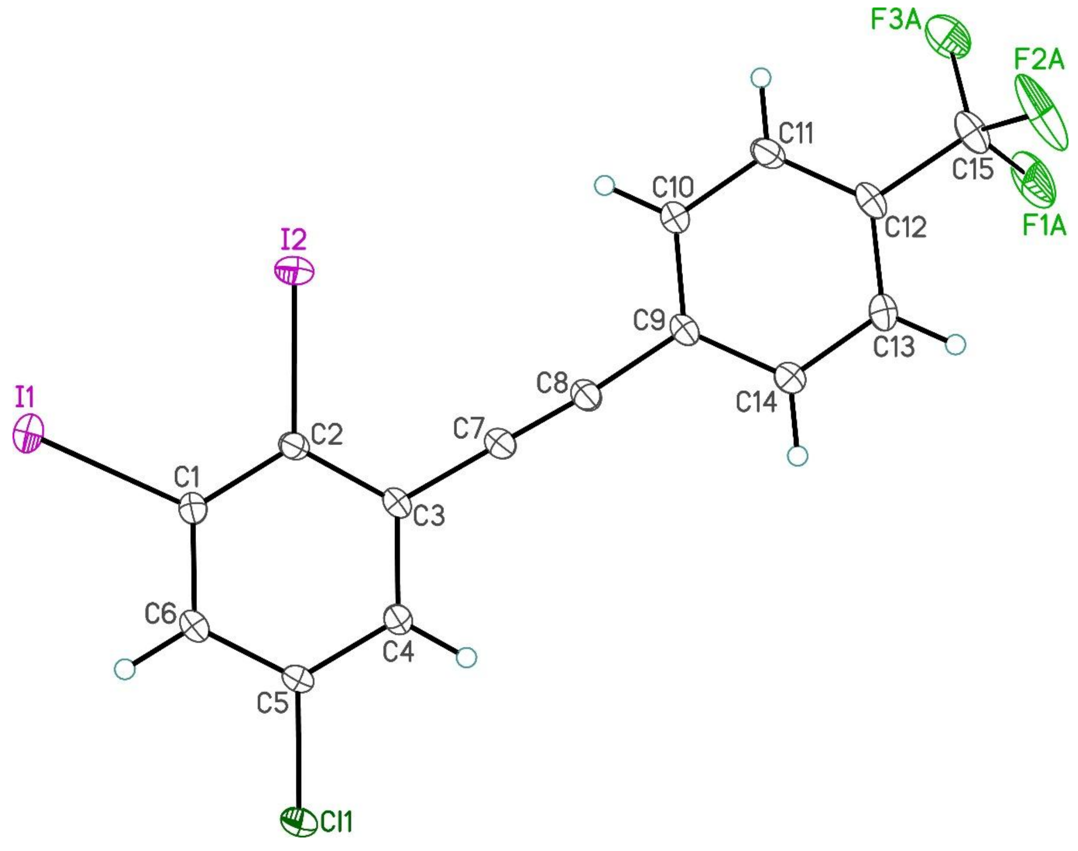

Figure Legends

Figure 1. Perspective view of the 5-chloro-1,2-diiodo-3-{[4-(trifluoromethyl)phenyl]ethynyl}benzene molecule showing the atom labelling scheme. Only the major orientation of the disordered trifluoromethyl groups is shown. Non-hydrogen atoms are represented by Gaussian ellipsoids at the 30% probability level. Hydrogen atoms are shown with arbitrarily small thermal parameters.

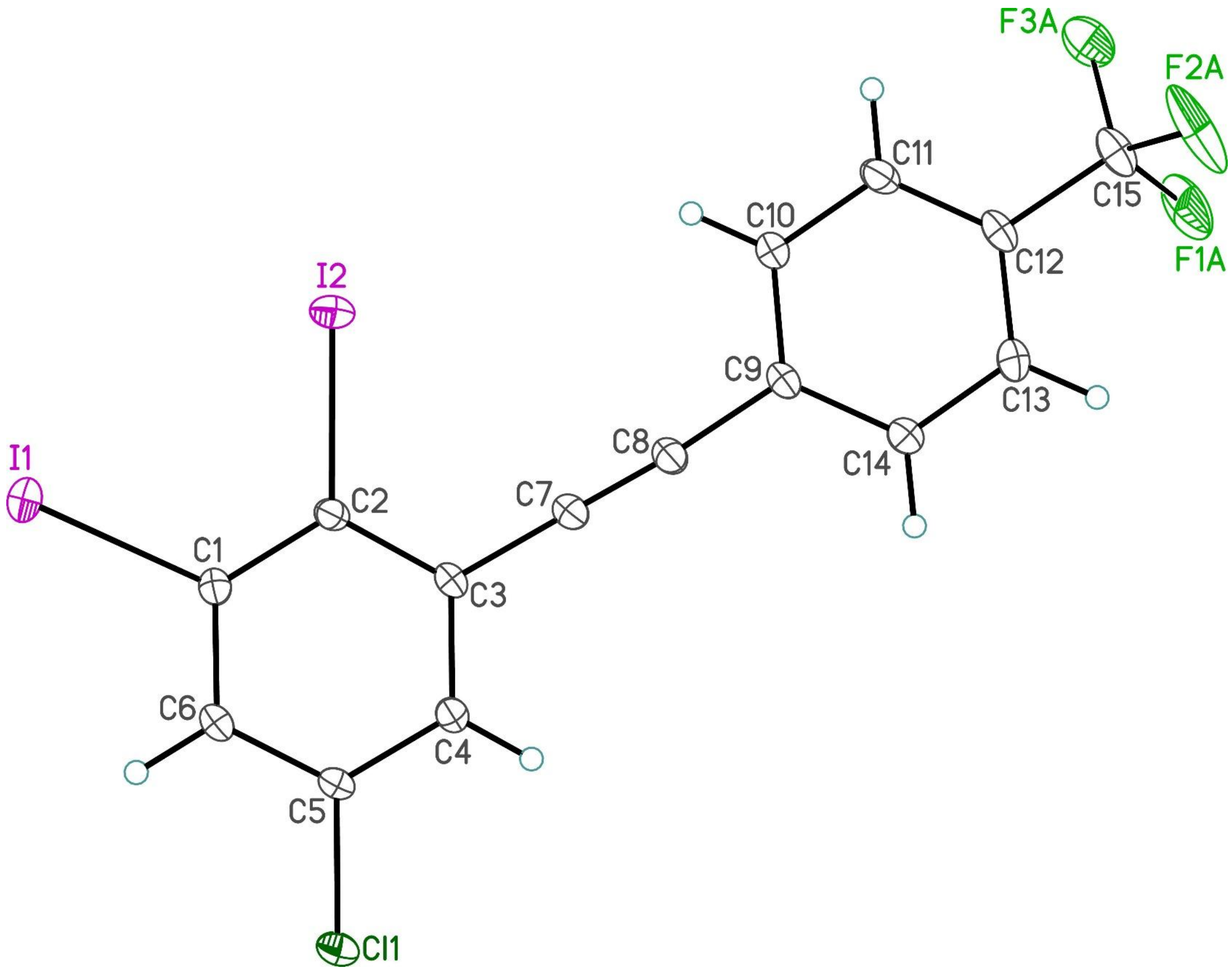
F3A
F2A
C11
C15
C10
F1A
C12
I2
C9
C13
C8
C14
I1
C7
C2
C1
C3
C6
C4
C5
Cl1

List of Tables

Table 1. Crystallographic Experimental Details

Table 2. Atomic Coordinates and Equivalent Isotropic Displacement Parameters

Table 3. Selected Interatomic Distances

Table 4. Selected Interatomic Angles

Table 5. Torsional Angles

Table 6. Anisotropic Displacement Parameters

Table 7. Derived Atomic Coordinates and Displacement Parameters for Hydrogen Atoms

Table 1. Crystallographic Experimental Details

A. Crystal Data	
formula	$C_{15}H_6ClF_3I_2$
formula weight	532.45
crystal dimensions (mm)	0.22 × 0.18 × 0.07
crystal system	triclinic
space group	$P\bar{1}$ (No. 2)
unit cell parameters[a]	
a (Å)	8.4281(5)
b (Å)	9.5746(5)
c (Å)	11.4227(6)
α (deg)	72.6614(8)
β (deg)	71.6046(7)
γ (deg)	64.6041(7)
V (Å^3)	775.78(7)
Z	2
ρ_{calcd} (g cm^{-3})	2.279
μ (mm^{-1})	4.246
B. Data Collection and Refinement Conditions	
diffractometer	Bruker D8/APEX II CCD[b]
radiation (λ [Å])	graphite-monochromated Mo Kα (0.71073)
temperature (°C)	–100
scan type	ω scans (0.3°) (10 s exposures)
data collection 2θ limit (deg)	61.26
total data collected	9508 ($-12 \le h \le 12$, $-13 \le k \le 13$, $-16 \le l \le 16$)
independent reflections	4763 (R_{int} = 0.0091)
number of observed reflections (*NO*)	4325 [$F_o^2 \ge 2\sigma(F_o^2)$]
structure solution method	intrinsic phasing (*SHELXT-2014*[c])
refinement method	full-matrix least-squares on F^2 (*SHELXL–2016*[d])
absorption correction method	Gaussian integration (face-indexed)
range of transmission factors	0.4694–0.3060
data/restraints/parameters	4763 / 0 / 203
goodness-of-fit (S)[e] [all data]	1.021
final R indices[f]	
R_1 [$F_o^2 \ge 2\sigma(F_o^2)$]	0.0180
wR_2 [all data]	0.0426
largest difference peak and hole	1.103 and –0.604 e Å^{-3}

[a]Obtained from least-squares refinement of 9944 reflections with 4.80° < 2θ < 61.14°.

(continued)

Table 1. Crystallographic Experimental Details (continued)

[b]Programs for diffractometer operation, data collection, data reduction and absorption correction were those supplied by Bruker

[c]Sheldrick, G. M. *Acta Crystallogr.* **2015**, *A71*, 3–8. (*SHELXT-2014*)

[d]Sheldrick, G. M. *Acta Crystallogr.* **2015**, *C71*, 3–8. (*SHELXL-2017*)

[e]$S = [\Sigma w(F_o^2 - F_c^2)^2/(n - p)]^{1/2}$ (n = number of data; p = number of parameters varied; $w = [\sigma^2(F_o^2) + (0.0165P)^2 + 0.6203P]^{-1}$ where $P = [\mathrm{Max}(F_o^2, 0) + 2F_c^2]/3$).

[f]$R_1 = \Sigma||F_o| - |F_c||/\Sigma|F_o|$; $wR_2 = [\Sigma w(F_o^2 - F_c^2)^2/\Sigma w(F_o^4)]^{1/2}$.

Table 2. Atomic Coordinates and Equivalent Isotropic Displacement Parameters

Atom	*x*	*y*	*z*	U_{eq}, Å^2
I1	0.44908(2)	0.08527(2)	0.14714(2)	0.03707(4)*
I2	0.25724(2)	0.37122(2)	0.35374(2)	0.03377(4)*
Cl1	1.05747(6)	-0.19302(6)	0.36331(5)	0.03706(11)*
F1A[a]	0.1791(3)	0.7698(3)	1.0301(2)	0.0708(8)*
F2A[a]	-0.0121(4)	0.6629(3)	1.0946(2)	0.0831(11)*
F3A[a]	-0.0486(4)	0.8712(2)	0.9507(2)	0.0779(8)*
F1B[b]	0.137(3)	0.695(2)	1.0837(19)	0.055(5)
F2B[b]	-0.096(2)	0.7246(18)	1.0398(15)	0.034(4)
F3B[b]	0.030(2)	0.8685(19)	0.9569(15)	0.032(4)
C1	0.5799(2)	0.07908(19)	0.27788(15)	0.0238(3)*
C2	0.5027(2)	0.18285(18)	0.36200(15)	0.0217(3)*
C3	0.5946(2)	0.16239(18)	0.45313(15)	0.0218(3)*
C4	0.7641(2)	0.0427(2)	0.45541(16)	0.0243(3)*
C5	0.8414(2)	-0.05230(19)	0.36643(16)	0.0245(3)*
C6	0.7502(2)	-0.0383(2)	0.27907(16)	0.0260(3)*
C7	0.5171(2)	0.2612(2)	0.54510(16)	0.0249(3)*
C8	0.4514(2)	0.3407(2)	0.62378(16)	0.0253(3)*
C9	0.3587(2)	0.4396(2)	0.71634(16)	0.0240(3)*
C10	0.2050(2)	0.5720(2)	0.69627(16)	0.0272(3)*
C11	0.1113(2)	0.6681(2)	0.78531(17)	0.0292(4)*
C12	0.1722(3)	0.6318(2)	0.89390(17)	0.0281(3)*
C13	0.3244(3)	0.5000(2)	0.91547(18)	0.0343(4)*
C14	0.4185(3)	0.4038(2)	0.82681(18)	0.0320(4)*
C15	0.0718(3)	0.7332(3)	0.9918(2)	0.0380(4)*

Anisotropically-refined atoms are marked with an asterisk (*). The form of the anisotropic displacement parameter is: $\exp[-2\pi^2(h^2a^{*2}U_{11} + k^2b^{*2}U_{22} + l^2c^{*2}U_{33} + 2klb^*c^*U_{23} + 2hla^*c^*U_{13} + 2hka^*b^*U_{12})]$. Refined occupancies of 0.912(5)[a] and 0.088(5)[b].

Table 3. Selected Interatomic Distances (Å)

Atom1	Atom2	Distance	Atom1	Atom2	Distance
I1	C1	2.0930(17)	C3	C7	1.433(2)
I2	C2	2.0887(16)	C4	C5	1.382(2)
Cl1	C5	1.7368(17)	C5	C6	1.385(2)
F1A	C15	1.321(3)	C7	C8	1.195(2)
F2A	C15	1.321(3)	C8	C9	1.437(2)
F3A	C15	1.334(3)	C9	C10	1.394(2)
F1B	C15	1.226(19)	C9	C14	1.400(3)
F2B	C15	1.375(15)	C10	C11	1.390(2)
F3B	C15	1.163(16)	C11	C12	1.383(3)
C1	C2	1.398(2)	C12	C13	1.387(3)
C1	C6	1.394(2)	C12	C15	1.502(2)
C2	C3	1.407(2)	C13	C14	1.388(2)
C3	C4	1.399(2)			

Table 4. Selected Interatomic Angles (deg)

Atom1	Atom2	Atom3	Angle	Atom1	Atom2	Atom3	Angle
I1	C1	C2	123.10(12)	C9	C10	C11	120.33(17)
I1	C1	C6	116.08(12)	C10	C11	C12	119.49(17)
C2	C1	C6	120.80(15)	C11	C12	C13	120.83(16)
I2	C2	C1	121.91(12)	C11	C12	C15	120.32(18)
I2	C2	C3	118.87(11)	C13	C12	C15	118.83(18)
C1	C2	C3	119.20(15)	C12	C13	C14	119.90(17)
C2	C3	C4	119.74(14)	C9	C14	C13	119.80(17)
C2	C3	C7	121.23(15)	F1A	C15	F2A	105.2(2)
C4	C3	C7	119.03(15)	F1A	C15	F3A	104.9(2)
C3	C4	C5	119.64(16)	F1A	C15	C12	112.51(18)
Cl1	C5	C4	119.64(13)	F2A	C15	F3A	108.1(2)
Cl1	C5	C6	118.86(13)	F2A	C15	C12	112.55(17)
C4	C5	C6	121.50(16)	F3A	C15	C12	113.06(19)
C1	C6	C5	118.95(15)	F1B	C15	F2B	104.9(12)
C3	C7	C8	178.48(19)	F1B	C15	F3B	108.8(12)
C7	C8	C9	175.39(19)	F1B	C15	C12	116.2(9)
C8	C9	C10	119.25(16)	F2B	C15	F3B	99.5(10)
C8	C9	C14	121.10(16)	F2B	C15	C12	108.5(6)
C10	C9	C14	119.64(15)	F3B	C15	C12	116.9(8)

Table 5. Torsional Angles (deg)

Atom1	Atom2	Atom3	Atom4	Angle	Atom1	Atom2	Atom3	Atom4	Angle
I1	C1	C2	I2	-6.4(2)	C10	C9	C14	C13	0.1(3)
I1	C1	C2	C3	175.11(12)	C9	C10	C11	C12	0.3(3)
C6	C1	C2	I2	175.00(13)	C10	C11	C12	C13	-0.6(3)
C6	C1	C2	C3	-3.5(3)	C10	C11	C12	C15	-179.38(18)
I1	C1	C6	C5	-177.74(13)	C11	C12	C13	C14	0.7(3)
C2	C1	C6	C5	0.9(3)	C15	C12	C13	C14	179.47(19)
I2	C2	C3	C4	-176.20(12)	C11	C12	C15	F1A	-132.4(2)
I2	C2	C3	C7	4.4(2)	C11	C12	C15	F2A	109.0(3)
C1	C2	C3	C4	2.3(2)	C11	C12	C15	F3A	-13.8(3)
C1	C2	C3	C7	-177.05(16)	C11	C12	C15	F1B	-178.2(13)
C2	C3	C4	C5	1.3(3)	C11	C12	C15	F2B	64.0(8)
C7	C3	C4	C5	-179.28(16)	C11	C12	C15	F3B	-47.4(10)
C3	C4	C5	Cl1	176.10(13)	C13	C12	C15	F1A	48.8(3)
C3	C4	C5	C6	-4.0(3)	C13	C12	C15	F2A	-69.8(3)
Cl1	C5	C6	C1	-177.24(14)	C13	C12	C15	F3A	167.4(2)
C4	C5	C6	C1	2.8(3)	C13	C12	C15	F1B	3.0(13)
C8	C9	C10	C11	178.98(17)	C13	C12	C15	F2B	-114.8(8)
C14	C9	C10	C11	-0.1(3)	C13	C12	C15	F3B	133.8(10)
C8	C9	C14	C13	-178.88(19)	C12	C13	C14	C9	-0.4(3)

Table 6. Anisotropic Displacement Parameters (U_{ij}, Å^2)

Atom	U_{11}	U_{22}	U_{33}	U_{23}	U_{13}	U_{12}
I1	0.03946(7)	0.04242(7)	0.03313(7)	-0.01448(5)	-0.01690(5)	-0.00749(6)
I2	0.02610(6)	0.02719(6)	0.04124(7)	-0.01034(5)	-0.01030(5)	0.00095(4)
Cl1	0.0244(2)	0.0370(2)	0.0426(3)	-0.0202(2)	-0.00876(18)	0.00449(17)
F1A	0.0694(12)	0.0927(16)	0.0776(15)	-0.0666(13)	0.0063(10)	-0.0373(11)
F2A	0.118(2)	0.0710(14)	0.0545(13)	-0.0428(11)	0.0533(14)	-0.0605(16)
F3A	0.0773(17)	0.0584(11)	0.0795(14)	-0.0514(11)	-0.0257(12)	0.0230(11)
C1	0.0262(8)	0.0242(7)	0.0214(7)	-0.0069(6)	-0.0059(6)	-0.0076(6)
C2	0.0200(7)	0.0196(7)	0.0229(7)	-0.0061(5)	-0.0033(6)	-0.0046(6)
C3	0.0230(7)	0.0201(7)	0.0220(7)	-0.0084(6)	-0.0013(6)	-0.0075(6)
C4	0.0230(8)	0.0256(8)	0.0246(8)	-0.0099(6)	-0.0049(6)	-0.0061(6)
C5	0.0206(7)	0.0224(7)	0.0269(8)	-0.0098(6)	-0.0037(6)	-0.0024(6)
C6	0.0265(8)	0.0252(8)	0.0247(8)	-0.0119(6)	-0.0028(6)	-0.0053(6)
C7	0.0234(8)	0.0242(7)	0.0255(8)	-0.0081(6)	-0.0026(6)	-0.0069(6)
C8	0.0244(8)	0.0256(8)	0.0255(8)	-0.0093(6)	-0.0024(6)	-0.0079(6)
C9	0.0241(8)	0.0244(7)	0.0233(7)	-0.0102(6)	-0.0003(6)	-0.0085(6)
C10	0.0262(8)	0.0301(8)	0.0233(8)	-0.0098(6)	-0.0036(6)	-0.0067(7)
C11	0.0257(8)	0.0254(8)	0.0304(9)	-0.0105(7)	-0.0012(7)	-0.0038(7)
C12	0.0308(9)	0.0275(8)	0.0269(8)	-0.0144(7)	0.0037(7)	-0.0124(7)
C13	0.0391(10)	0.0374(10)	0.0279(9)	-0.0154(7)	-0.0096(8)	-0.0078(8)
C14	0.0314(9)	0.0297(9)	0.0322(9)	-0.0142(7)	-0.0099(7)	-0.0011(7)
C15	0.0440(11)	0.0361(10)	0.0349(10)	-0.0208(8)	0.0055(8)	-0.0162(9)

The form of the anisotropic displacement parameter is:
$\exp[-2\pi^2(h^2a^{*2}U_{11} + k^2b^{*2}U_{22} + l^2c^{*2}U_{33} + 2klb^*c^*U_{23} + 2hla^*c^*U_{13} + 2hka^*b^*U_{12})]$

Table 7. Derived Atomic Coordinates and Displacement Parameters for Hydrogen Atoms

Atom	x	y	z	U_{eq}, Å^2
H4	0.825829	0.026761	0.517786	0.029
H6	0.802955	-0.107557	0.220897	0.031
H10	0.164151	0.596713	0.621359	0.033
H11	0.006301	0.758089	0.771689	0.035
H13	0.364075	0.475653	0.990826	0.041
H14	0.523292	0.313854	0.841038	0.038

1.6.3 X-ray data of 5-fluoro-1,2-diiodo-3-[(4-methoxyphenyl)ethynyl]benzene (23_A)

STRUCTURE REPORT

XCL Code: JUS1809 **Date:** 3 December 2018

Compound: 5-fluoro-1,2-diiodo-3-[(4-methoxyphenyl)ethynyl]benzene
Formula: $C_{15}H_9FI_2O$

Supervisor: R. Al-Zoubi, Jordan University of Science and Technology

Crystallographer: M. J. Ferguson

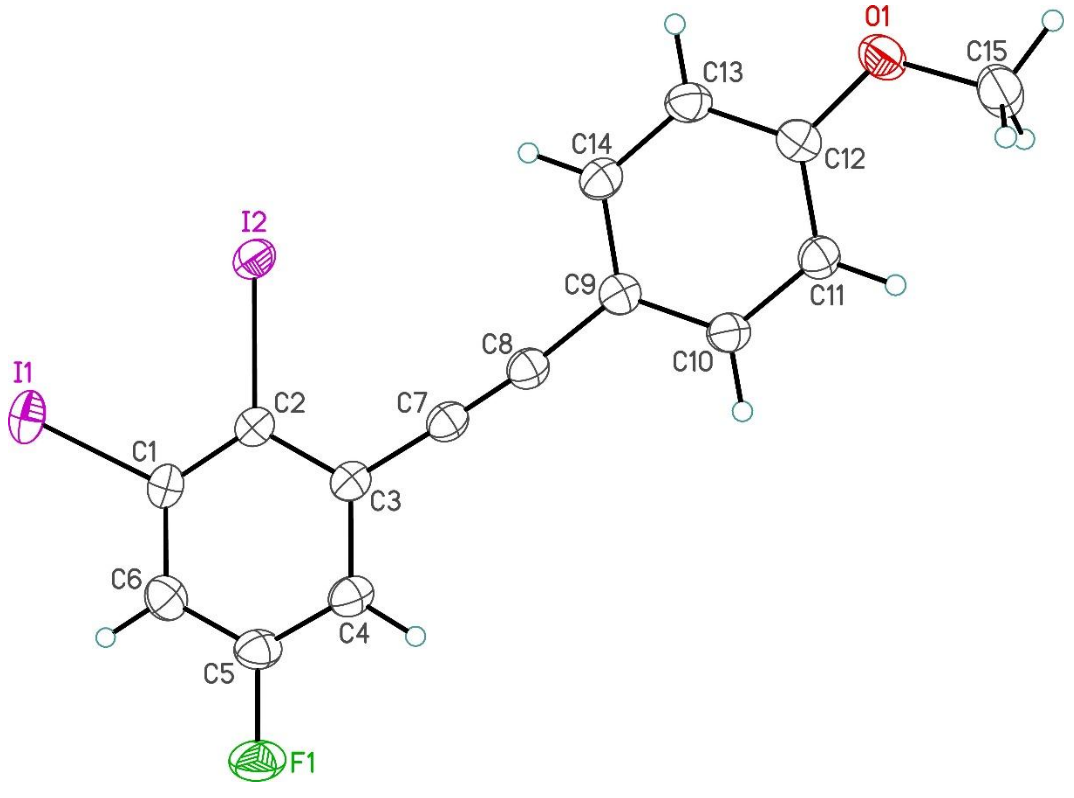

Figure Legends

Figure 1. Perspective view of the 5-fluoro-1,2-diiodo-3-[(4-methoxyphenyl)ethynyl]benzene molecule showing the atom labelling scheme. Non-hydrogen atoms are represented by Gaussian ellipsoids at the 30% probability level. Hydrogen atoms are shown with arbitrarily small thermal parameters.

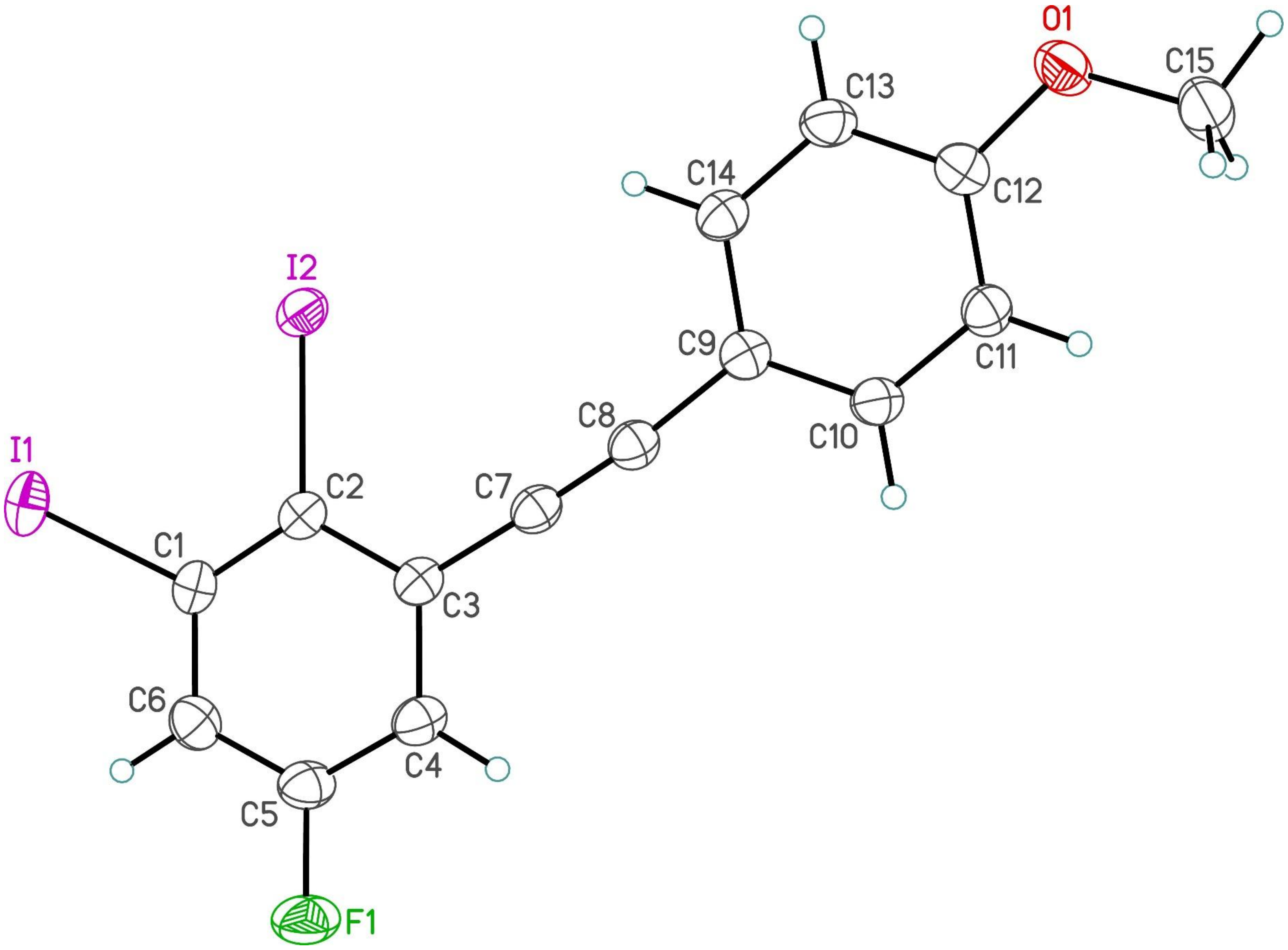
O1
C15
C13
C14
C12
I2
C9
C11
C8
C10
I1
C2
C7
C1
C3
C6
C4
C5
F1

List of Tables

Table 1. Crystallographic Experimental Details

Table 2. Atomic Coordinates and Equivalent Isotropic Displacement Parameters

Table 3. Selected Interatomic Distances

Table 4. Selected Interatomic Angles

Table 5. Torsional Angles

Table 6. Anisotropic Displacement Parameters

Table 7. Derived Atomic Coordinates and Displacement Parameters for Hydrogen Atoms

Table 1. Crystallographic Experimental Details

A. Crystal Data	
formula	$C_{15}H_9FI_2O$
formula weight	478.02
crystal dimensions (mm)	0.16 × 0.13 × 0.03
crystal system	orthorhombic
space group	$P2_12_12_1$ (No. 19)
unit cell parameters[a]	
a (Å)	4.15290(8)
b (Å)	11.4859(2)
c (Å)	30.5943(6)
V (Å^3)	1459.35(5)
Z	4
ρ_{calcd} (g cm^{-3})	2.176
μ (mm^{-1})	33.86
B. Data Collection and Refinement Conditions	
diffractometer	Bruker D8/APEX II CCD[b]
radiation (λ [Å])	Cu Kα (1.54178) (microfocus source)
temperature (°C)	–100
scan type	ω and ϕ scans (1.0°) (5 s exposures)
data collection 2θ limit (deg)	148.17
total data collected	9797 ($-5 \le h \le 5$, $-13 \le k \le 14$, $-37 \le l \le 38$)
independent reflections	2934 (R_{int} = 0.0232)
number of observed reflections (*NO*)	2918 [$F_o^2 \ge 2\sigma(F_o^2)$]
structure solution method	intrinsic phasing (*SHELXT-2014*[c])
refinement method	full-matrix least-squares on F^2 (*SHELXL–2016*[d])
absorption correction method	Gaussian integration (face-indexed)
range of transmission factors	0.1121–0.0046
data/restraints/parameters	2934 / 0 / 174
Flack absolute structure parameter[e]	0.037(8)
goodness-of-fit (S)[f] [all data]	1.112
final R indices[g]	
R_1 [$F_o^2 \ge 2\sigma(F_o^2)$]	0.0149
wR_2 [all data]	0.0380
largest difference peak and hole	0.305 and –0.294 e Å^{-3}

[a]Obtained from least-squares refinement of 9894 reflections with 8.22° < 2θ < 147.26°.

[b]Programs for diffractometer operation, data collection, data reduction and absorption correction were those supplied by Bruker

(continued)

Table 1. Crystallographic Experimental Details (continued)

[c]Sheldrick, G. M. *Acta Crystallogr.* **2015**, *A71*, 3–8. (*SHELXT-2014*)

[d]Sheldrick, G. M. *Acta Crystallogr.* **2015**, *C71*, 3–8. (*SHELXL-2017*)

[e]Flack, H. D. *Acta Crystallogr.* **1983**, *A39*, 876–881; Flack, H. D.; Bernardinelli, G. *Acta Crystallogr.* **1999**, *A55*, 908–915; Flack, H. D.; Bernardinelli, G. *J. Appl. Cryst.* **2000**, *33*, 1143–1148. The Flack parameter will refine to a value near zero if the structure is in the correct configuration and will refine to a value near one for the inverted configuration. The value observed herein is indicative of racemic twinning, and was accomodated during the refinement (using the *SHELXL-2014* TWIN instruction [see reference *d*]).

[f]$S = [\Sigma w(F_o^2 - F_c^2)^2/(n - p)]^{1/2}$ (n = number of data; p = number of parameters varied; $w = [\sigma^2(F_o^2) + (0.0147P)^2 + 0.2798P]^{-1}$ where $P = [\mathrm{Max}(F_o^2, 0) + 2F_c^2]/3$).

[g]$R_1 = \Sigma||F_o| - |F_c||/\Sigma|F_o|$; $wR_2 = [\Sigma w(F_o^2 - F_c^2)^2/\Sigma w(F_o^4)]^{1/2}$.

Table 2. Atomic Coordinates and Equivalent Isotropic Displacement Parameters

Atom	x	y	z	U_{eq}, Å^2
I1	-0.06974(6)	0.63448(2)	0.52821(2)	0.05149(8)*
I2	0.24743(6)	0.67450(2)	0.63847(2)	0.04729(7)*
F1	0.2990(10)	0.1948(2)	0.54633(8)	0.0723(9)*
O1	1.1160(9)	0.5739(3)	0.87119(9)	0.0561(7)*
C1	0.1469(8)	0.5001(3)	0.56417(11)	0.0401(7)*
C2	0.2755(9)	0.5159(3)	0.60570(10)	0.0385(6)*
C3	0.4262(10)	0.4211(3)	0.62718(10)	0.0394(7)*
C4	0.4359(11)	0.3119(3)	0.60624(11)	0.0470(8)*
C5	0.2982(12)	0.3013(3)	0.56583(12)	0.0507(9)*
C6	0.1561(10)	0.3915(4)	0.54378(11)	0.0496(9)*
C7	0.5640(10)	0.4345(3)	0.66967(11)	0.0414(7)*
C8	0.6764(9)	0.4488(3)	0.70508(11)	0.0426(8)*
C9	0.7992(9)	0.4771(3)	0.74767(11)	0.0409(7)*
C10	0.9692(10)	0.3973(3)	0.77266(11)	0.0436(8)*
C11	1.0819(11)	0.4262(3)	0.81398(11)	0.0429(7)*
C12	1.0227(10)	0.5374(3)	0.83070(11)	0.0432(8)*
C13	0.8563(10)	0.6189(3)	0.80560(13)	0.0481(9)*
C14	0.7451(12)	0.5889(3)	0.76452(11)	0.0450(7)*
C15	1.2571(15)	0.4896(4)	0.89985(13)	0.0617(11)*

Anisotropically-refined atoms are marked with an asterisk (*). The form of the anisotropic displacement parameter is: $\exp[-2\pi^2(h^2a^{*2}U_{11} + k^2b^{*2}U_{22} + l^2c^{*2}U_{33} + 2klb^*c^*U_{23} + 2hla^*c^*U_{13} + 2hka^*b^*U_{12})]$.

Table 3. Selected Interatomic Distances (Å)

Atom1	Atom2	Distance	Atom1	Atom2	Distance
I1	C1	2.098(3)	C4	C5	1.368(6)
I2	C2	2.083(3)	C5	C6	1.370(6)
F1	C5	1.361(5)	C7	C8	1.191(5)
O1	C12	1.364(4)	C8	C9	1.437(5)
O1	C15	1.432(5)	C9	C10	1.387(5)
C1	C2	1.390(5)	C9	C14	1.401(5)
C1	C6	1.395(6)	C10	C11	1.388(5)
C2	C3	1.418(5)	C11	C12	1.397(5)
C3	C4	1.409(5)	C12	C13	1.395(6)
C3	C7	1.429(5)	C13	C14	1.383(5)

Table 4. Selected Interatomic Angles (deg)

Atom1	Atom2	Atom3	Angle	Atom1	Atom2	Atom3	Angle
C12	O1	C15	117.7(3)	C1	C6	C5	117.9(3)
I1	C1	C2	123.2(3)	C3	C7	C8	178.2(4)
I1	C1	C6	115.8(3)	C7	C8	C9	174.5(4)
C2	C1	C6	121.0(3)	C8	C9	C10	122.0(3)
I2	C2	C1	122.2(3)	C8	C9	C14	119.0(3)
I2	C2	C3	118.3(2)	C10	C9	C14	119.0(3)
C1	C2	C3	119.5(3)	C9	C10	C11	121.0(3)
C2	C3	C4	119.0(3)	C10	C11	C12	119.5(4)
C2	C3	C7	121.1(3)	O1	C12	C11	124.3(4)
C4	C3	C7	119.9(3)	O1	C12	C13	115.7(3)
C3	C4	C5	118.6(4)	C11	C12	C13	119.9(3)
F1	C5	C4	118.4(4)	C12	C13	C14	119.9(4)
F1	C5	C6	117.7(4)	C9	C14	C13	120.6(4)
C4	C5	C6	123.9(4)				

Table 5. Torsional Angles (deg)

Atom1	Atom2	Atom3	Atom4	Angle	Atom1	Atom2	Atom3	Atom4	Angle
C15	O1	C12	C11	-6.0(6)	C3	C4	C5	F1	-177.9(4)
C15	O1	C12	C13	173.6(4)	C3	C4	C5	C6	1.4(7)
I1	C1	C2	I2	3.1(4)	F1	C5	C6	C1	178.3(4)
I1	C1	C2	C3	-178.1(3)	C4	C5	C6	C1	-1.1(7)
C6	C1	C2	I2	-177.1(3)	C8	C9	C10	C11	-179.2(4)
C6	C1	C2	C3	1.8(5)	C14	C9	C10	C11	0.7(6)
I1	C1	C6	C5	179.3(3)	C8	C9	C14	C13	179.2(4)
C2	C1	C6	C5	-0.5(6)	C10	C9	C14	C13	-0.7(6)
I2	C2	C3	C4	177.4(3)	C9	C10	C11	C12	0.2(6)
I2	C2	C3	C7	-1.8(5)	C10	C11	C12	O1	178.4(4)
C1	C2	C3	C4	-1.4(6)	C10	C11	C12	C13	-1.1(6)
C1	C2	C3	C7	179.3(3)	O1	C12	C13	C14	-178.4(4)
C2	C3	C4	C5	-0.1(6)	C11	C12	C13	C14	1.2(6)
C7	C3	C4	C5	179.1(4)	C12	C13	C14	C9	-0.3(6)

Table 6. Anisotropic Displacement Parameters (U_{ij}, Å^2)

Atom	U_{11}	U_{22}	U_{33}	U_{23}	U_{13}	U_{12}
I1	0.03646(11)	0.06050(15)	0.05750(12)	0.02345(11)	-0.00046(11)	0.00104(11)
I2	0.04044(11)	0.03703(11)	0.06441(13)	-0.00208(9)	-0.00114(11)	0.00220(10)
F1	0.115(3)	0.0521(14)	0.0503(12)	-0.0138(10)	-0.0008(14)	0.0115(16)
O1	0.076(2)	0.0482(15)	0.0440(13)	-0.0038(11)	-0.0077(14)	-0.0078(15)
C1	0.0335(16)	0.0436(19)	0.0433(16)	0.0112(13)	0.0023(12)	0.0001(13)
C2	0.0355(16)	0.0370(16)	0.0430(15)	0.0039(12)	0.0055(14)	-0.0001(15)
C3	0.0407(16)	0.0396(17)	0.0380(14)	0.0031(12)	0.0071(14)	0.0026(16)
C4	0.060(2)	0.0408(19)	0.0406(16)	0.0037(13)	0.0080(17)	0.0054(19)
C5	0.067(3)	0.046(2)	0.0391(16)	-0.0041(13)	0.0065(17)	0.0035(19)
C6	0.057(2)	0.057(2)	0.0352(14)	0.0030(14)	0.0010(14)	-0.0016(17)
C7	0.0434(18)	0.0377(17)	0.0432(16)	0.0035(12)	0.0064(15)	0.0029(17)
C8	0.046(2)	0.0393(18)	0.0429(17)	0.0032(13)	0.0043(13)	0.0008(14)
C9	0.0415(18)	0.0417(17)	0.0396(15)	0.0017(12)	0.0053(14)	-0.0004(14)
C10	0.049(2)	0.0380(18)	0.0438(16)	-0.0018(13)	0.0027(14)	0.0007(15)
C11	0.0457(19)	0.0400(18)	0.0431(16)	0.0039(13)	-0.0005(16)	-0.0027(17)
C12	0.046(2)	0.0424(19)	0.0414(16)	0.0003(13)	0.0034(13)	-0.0090(15)
C13	0.059(2)	0.0362(19)	0.0490(17)	-0.0027(14)	0.0026(16)	-0.0011(16)
C14	0.0477(19)	0.0400(17)	0.0473(16)	0.0032(13)	0.0031(17)	0.0029(18)
C15	0.077(3)	0.058(2)	0.050(2)	0.0043(16)	-0.014(2)	-0.012(3)

The form of the anisotropic displacement parameter is:
$\exp[-2\pi^2(h^2a^{*2}U_{11} + k^2b^{*2}U_{22} + l^2c^{*2}U_{33} + 2klb^*c^*U_{23} + 2hla^*c^*U_{13} + 2hka^*b^*U_{12})]$

Table 7. Derived Atomic Coordinates and Displacement Parameters for Hydrogen Atoms

Atom	x	y	z	U_{eq}, Å^2
H4	0.535828	0.246984	0.619904	0.056
H6	0.066699	0.380400	0.515506	0.059
H10	1.009023	0.321629	0.761338	0.052
H11	1.198483	0.370867	0.830762	0.052
H13	0.819425	0.694991	0.816703	0.058
H14	0.630933	0.644532	0.747562	0.054
H15A	1.286659	0.524056	0.928881	0.074
H15B	1.466455	0.465302	0.888212	0.074
H15C	1.114923	0.421781	0.902078	0.074

1.6.4 X-ray data of 5-chloro-2-iodo-1,3-bis{[4-trifluoromethyl)phenyl]ethynyl} benzene (35A)

STRUCTURE REPORT

XCL Code: JUS1803 **Date:** 24 September 2018

Compound: 5-chloro-2-iodo-1,3-bis{[4-trifluoromethyl)phenyl]ethynyl}benzene
Formula: $C_{24}H_{10}ClF_6I$

Supervisor: R. Al-Zoubi, Jordan University of Science and Technology

Crystallographer: M. J. Ferguson

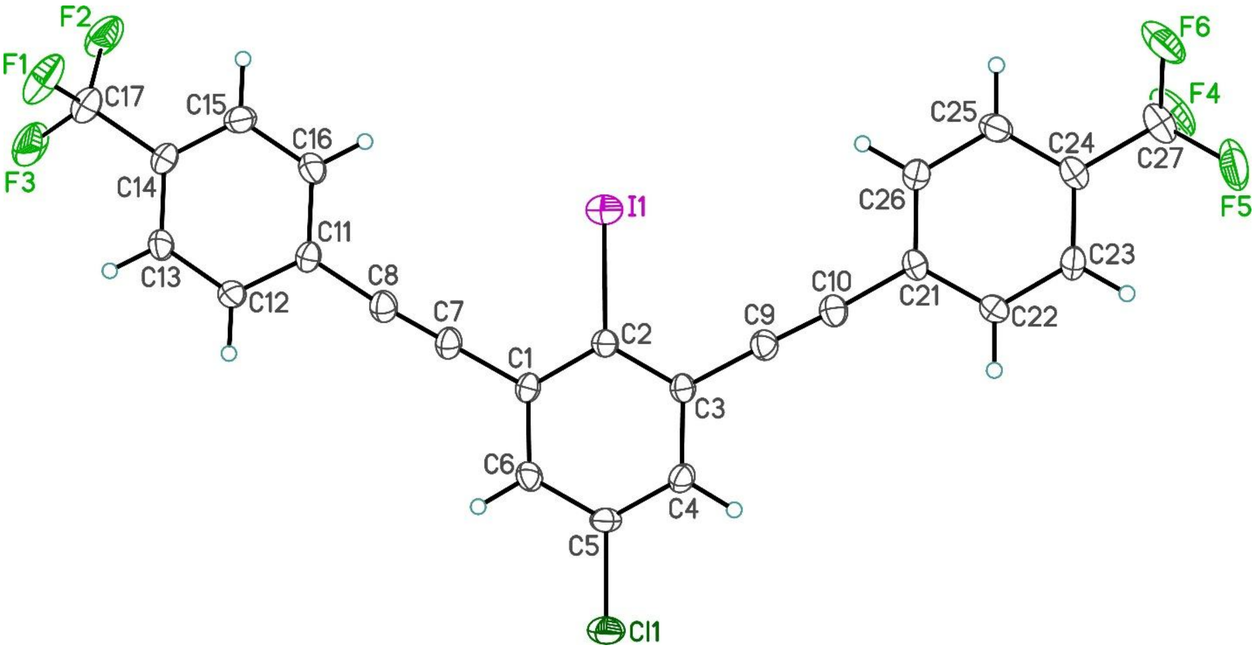

Figure Legends

Figure 1. Perspective view of the 5-chloro-2-iodo-1,3-bis{[4-trifluoromethyl)phenyl]eth–ynyl}benzene molecule showing the atom labelling scheme. Non-hydrogen atoms are represented by Gaussian ellipsoids at the 30% probability level. Hydrogen atoms are shown with arbitrarily small thermal parameters.

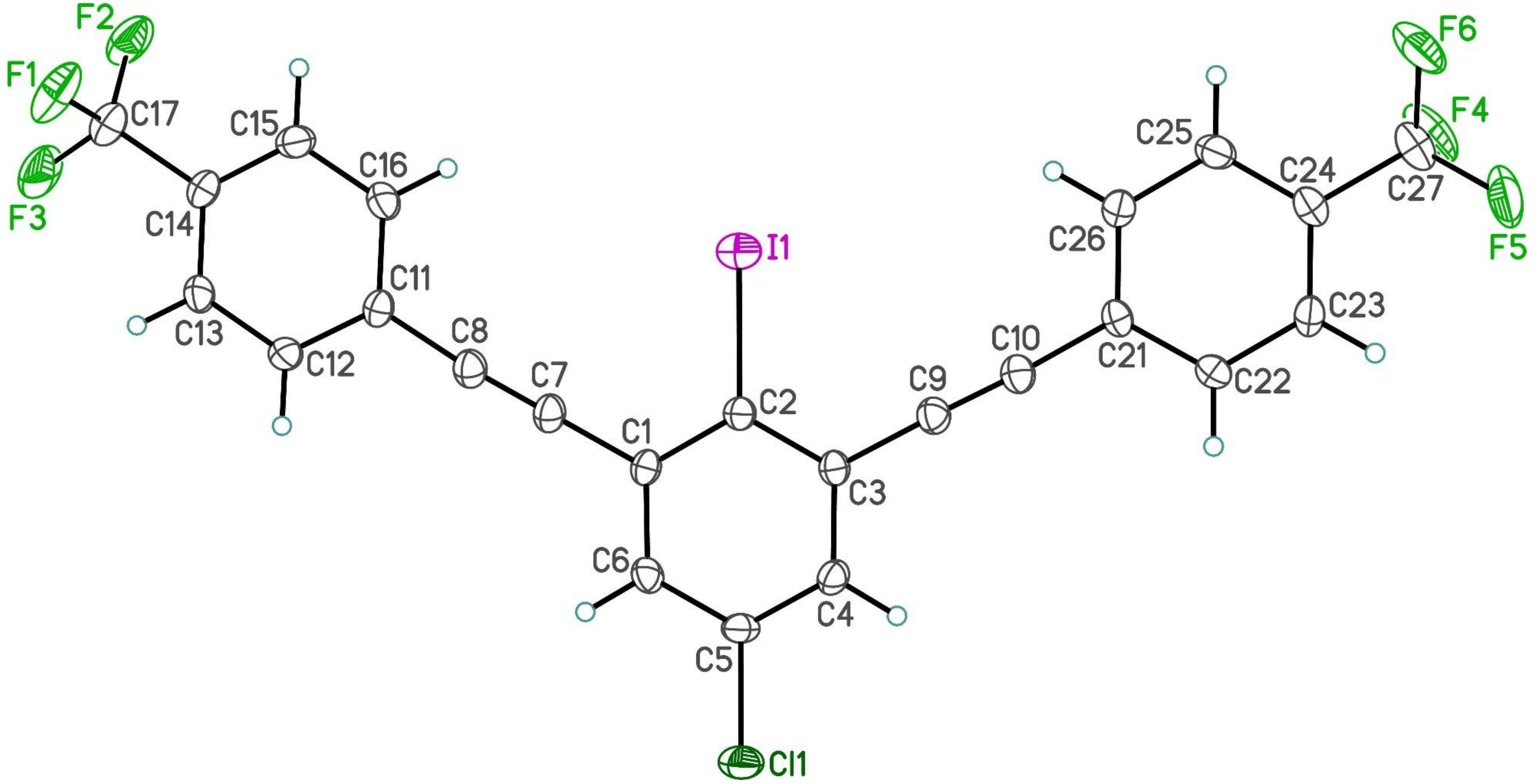
F2
F1
C17
F3
C15
C16
C14
C11
C13
C12
C8
C7
I1
C2
C1
C3
C6
C4
C5
Cl1
C9
C10
C21
C22
C23
C26
C25
C24
C27
F6
F4
F5

List of Tables

Table 1. Crystallographic Experimental Details

Table 2. Atomic Coordinates and Equivalent Isotropic Displacement Parameters

Table 3. Selected Interatomic Distances

Table 4. Selected Interatomic Angles

Table 5. Torsional Angles

Table 6. Anisotropic Displacement Parameters

Table 7. Derived Atomic Coordinates and Displacement Parameters for Hydrogen Atoms

Table 1. Crystallographic Experimental Details

A. Crystal Data	
formula	$C_{24}H_{10}ClF_6I$
formula weight	574.67
crystal dimensions (mm)	0.24 × 0.12 × 0.10
crystal system	triclinic
space group	$P\bar{1}$ (No. 2)
unit cell parameters[a]	
a (Å)	9.8013(12)
b (Å)	10.0715(12)
c (Å)	11.7314(14)
α (deg)	91.3034(15)
β (deg)	91.6389(14)
γ (deg)	111.5614(14)
V (Å^3)	1075.9(2)
Z	2
ρ_{calcd} (g cm^{-3})	1.774
μ (mm^{-1})	1.673
B. Data Collection and Refinement Conditions	
diffractometer	Bruker PLATFORM/APEX II CCD[b]
radiation (λ [Å])	graphite-monochromated Mo Kα (0.71073)
temperature (°C)	–80
scan type	ω scans (0.3°) (20 s exposures)
data collection 2θ limit (deg)	53.18
total data collected	8291 ($-12 \le h \le 12$, $-12 \le k \le 12$, $-14 \le l \le 14$)
independent reflections	4456 (R_{int} = 0.0189)
number of observed reflections (*NO*)	3710 [$F_o^2 \ge 2\sigma(F_o^2)$]
structure solution method	intrinsic phasing (*SHELXT-2014*[c])
refinement method	full-matrix least-squares on F^2 (*SHELXL–2016*[d])
absorption correction method	Gaussian integration (face-indexed)
range of transmission factors	0.9260–0.7647
data/restraints/parameters	4456 / 0 / 289
goodness-of-fit (S)[e] [all data]	1.050
final R indices[f]	
R_1 [$F_o^2 \ge 2\sigma(F_o^2)$]	0.0356
wR_2 [all data]	0.1027
largest difference peak and hole	0.504 and –0.518 e Å^{-3}

[a]Obtained from least-squares refinement of 4048 reflections with 4.48° < 2θ < 52.54°.

(continued)

Table 1. Crystallographic Experimental Details (continued)

[b]Programs for diffractometer operation, data collection, data reduction and absorption correction were those supplied by Bruker.

[c]Sheldrick, G. M. *Acta Crystallogr.* **2015**, *A71*, 3–8. (*SHELXT-2014*)

[d]Sheldrick, G. M. *Acta Crystallogr.* **2015**, *C71*, 3–8. (*SHELXL-2017*)

[e]$S = [\Sigma w(F_o^2 - F_c^2)^2/(n - p)]^{1/2}$ (n = number of data; p = number of parameters varied; $w = [\sigma^2(F_o^2) + (0.0510P)^2 + 0.8785P]^{-1}$ where $P = [\mathrm{Max}(F_o^2, 0) + 2F_c^2]/3$).

[f]$R_1 = \Sigma||F_o| - |F_c||/\Sigma|F_o|$; $wR_2 = [\Sigma w(F_o^2 - F_c^2)^2/\Sigma w(F_o^4)]^{1/2}$.

Table 2. Atomic Coordinates and Equivalent Isotropic Displacement Parameters

Atom	*x*	*y*	*z*	U_{eq}, Å^2
I1	0.33158(3)	0.65115(3)	0.25399(2)	0.05633(12)*
Cl1	-0.05318(11)	1.06507(11)	0.22057(9)	0.0539(2)*
F1	0.5486(3)	0.6717(3)	0.9926(2)	0.0763(8)*
F2	0.7593(3)	0.7694(3)	0.9250(2)	0.0725(8)*
F3	0.6677(4)	0.8953(3)	1.0176(2)	0.0873(9)*
F4	0.3254(3)	0.4116(3)	-0.4972(2)	0.0807(9)*
F5	0.0952(4)	0.2966(4)	-0.5169(2)	0.0882(10)*
F6	0.2262(4)	0.2104(3)	-0.4207(2)	0.0779(8)*
C1	0.2036(3)	0.8656(3)	0.3393(2)	0.0307(6)*
C2	0.2122(3)	0.7838(3)	0.2430(3)	0.0312(6)*
C3	0.1407(3)	0.7911(3)	0.1396(3)	0.0305(6)*
C4	0.0584(4)	0.8793(3)	0.1342(3)	0.0343(7)*
C5	0.0509(3)	0.9585(3)	0.2289(3)	0.0344(7)*
C6	0.1221(4)	0.9533(3)	0.3312(3)	0.0342(7)*
C7	0.2776(4)	0.8607(4)	0.4458(3)	0.0362(7)*
C8	0.3399(4)	0.8579(4)	0.5336(3)	0.0375(7)*
C9	0.1482(4)	0.7110(3)	0.0388(3)	0.0353(7)*
C10	0.1524(4)	0.6458(4)	-0.0463(3)	0.0362(7)*
C11	0.4184(3)	0.8468(3)	0.6363(3)	0.0326(7)*
C12	0.4207(4)	0.9265(4)	0.7354(3)	0.0374(7)*
C13	0.4924(4)	0.9085(4)	0.8334(3)	0.0396(8)*
C14	0.5628(4)	0.8131(4)	0.8339(3)	0.0355(7)*
C15	0.5640(4)	0.7352(4)	0.7357(3)	0.0423(8)*
C16	0.4911(4)	0.7508(4)	0.6370(3)	0.0400(8)*
C17	0.6337(4)	0.7873(4)	0.9416(3)	0.0482(9)*
C21	0.1617(3)	0.5636(3)	-0.1454(3)	0.0319(7)*
C22	0.1010(4)	0.5789(4)	-0.2505(3)	0.0416(8)*
C23	0.1140(4)	0.5010(4)	-0.3453(3)	0.0448(9)*
C24	0.1870(4)	0.4081(4)	-0.3368(3)	0.0368(7)*
C25	0.2470(4)	0.3908(4)	-0.2331(3)	0.0416(8)*
C26	0.2345(4)	0.4694(4)	-0.1375(3)	0.0394(8)*
C27	0.2082(5)	0.3316(4)	-0.4419(3)	0.0514(10)*

Anisotropically-refined atoms are marked with an asterisk (*). The form of the anisotropic displacement parameter is: $\exp[-2\pi^2(h^2a^{*2}U_{11} + k^2b^{*2}U_{22} + l^2c^{*2}U_{33} + 2klb^*c^*U_{23} + 2hla^*c^*U_{13} + 2hka^*b^*U_{12})]$.

Table 3. Selected Interatomic Distances (Å)

Atom1	Atom2	Distance	Atom1	Atom2	Distance
I1	C2	2.078(3)	C8	C11	1.439(4)
Cl1	C5	1.734(3)	C9	C10	1.193(4)
F1	C17	1.325(5)	C10	C21	1.436(4)
F2	C17	1.327(5)	C11	C12	1.392(4)
F3	C17	1.327(5)	C11	C16	1.396(5)
F4	C27	1.332(5)	C12	C13	1.381(5)
F5	C27	1.330(5)	C13	C14	1.373(5)
F6	C27	1.323(5)	C14	C15	1.382(5)
C1	C2	1.406(4)	C14	C17	1.499(5)
C1	C6	1.395(5)	C15	C16	1.385(5)
C1	C7	1.437(4)	C21	C22	1.390(5)
C2	C3	1.400(4)	C21	C26	1.383(5)
C3	C4	1.403(4)	C22	C23	1.382(5)
C3	C9	1.435(4)	C23	C24	1.375(5)
C4	C5	1.372(4)	C24	C25	1.379(5)
C5	C6	1.383(5)	C24	C27	1.499(5)
C7	C8	1.189(5)	C25	C26	1.392(5)

Table 4. Selected Interatomic Angles (deg)

Atom1	Atom2	Atom3	Angle	Atom1	Atom2	Atom3	Angle
C2	C1	C6	119.3(3)	C15	C14	C17	119.0(3)
C2	C1	C7	121.2(3)	C14	C15	C16	119.9(3)
C6	C1	C7	119.5(3)	C11	C16	C15	120.0(3)
I1	C2	C1	119.9(2)	F1	C17	F2	106.2(3)
I1	C2	C3	119.8(2)	F1	C17	F3	106.8(3)
C1	C2	C3	120.4(3)	F1	C17	C14	112.2(3)
C2	C3	C4	119.0(3)	F2	C17	F3	104.9(3)
C2	C3	C9	122.3(3)	F2	C17	C14	113.2(3)
C4	C3	C9	118.7(3)	F3	C17	C14	112.9(3)
C3	C4	C5	120.0(3)	C10	C21	C22	121.1(3)
Cl1	C5	C4	119.2(2)	C10	C21	C26	119.9(3)
Cl1	C5	C6	119.3(2)	C22	C21	C26	119.0(3)
C4	C5	C6	121.6(3)	C21	C22	C23	120.1(3)
C1	C6	C5	119.7(3)	C22	C23	C24	120.5(3)
C1	C7	C8	179.3(4)	C23	C24	C25	120.1(3)
C7	C8	C11	175.9(4)	C23	C24	C27	119.7(3)
C3	C9	C10	178.5(4)	C25	C24	C27	120.1(3)
C9	C10	C21	177.0(4)	C24	C25	C26	119.5(3)
C8	C11	C12	121.8(3)	C21	C26	C25	120.7(3)
C8	C11	C16	118.9(3)	F4	C27	F5	106.1(4)
C12	C11	C16	119.3(3)	F4	C27	F6	106.7(3)
C11	C12	C13	120.0(3)	F4	C27	C24	111.6(3)
C12	C13	C14	120.5(3)	F5	C27	F6	105.5(3)
C13	C14	C15	120.3(3)	F5	C27	C24	113.0(3)
C13	C14	C17	120.7(3)	F6	C27	C24	113.4(3)

Table 5. Torsional Angles (deg)

Atom1	Atom2	Atom3	Atom4	Angle	Atom1	Atom2	Atom3	Atom4	Angle
C6	C1	C2	I1	-179.5(2)	C17	C14	C15	C16	175.9(3)
C6	C1	C2	C3	0.5(5)	C13	C14	C17	F1	97.8(4)
C7	C1	C2	I1	0.7(4)	C13	C14	C17	F2	-142.0(4)
C7	C1	C2	C3	-179.3(3)	C13	C14	C17	F3	-23.0(5)
C2	C1	C6	C5	0.1(5)	C15	C14	C17	F1	-79.7(5)
C7	C1	C6	C5	179.9(3)	C15	C14	C17	F2	40.6(5)
I1	C2	C3	C4	178.9(2)	C15	C14	C17	F3	159.5(4)
I1	C2	C3	C9	-0.9(4)	C14	C15	C16	C11	1.0(5)
C1	C2	C3	C4	-1.1(5)	C10	C21	C22	C23	178.6(3)
C1	C2	C3	C9	179.1(3)	C26	C21	C22	C23	-0.1(5)
C2	C3	C4	C5	1.1(5)	C10	C21	C26	C25	-178.8(3)
C9	C3	C4	C5	-179.1(3)	C22	C21	C26	C25	0.0(5)
C3	C4	C5	Cl1	-179.2(3)	C21	C22	C23	C24	-0.1(6)
C3	C4	C5	C6	-0.5(5)	C22	C23	C24	C25	0.6(6)
Cl1	C5	C6	C1	178.7(2)	C22	C23	C24	C27	-176.1(4)
C4	C5	C6	C1	-0.1(5)	C23	C24	C25	C26	-0.8(5)
C8	C11	C12	C13	177.4(3)	C27	C24	C25	C26	175.9(3)
C16	C11	C12	C13	-1.0(5)	C23	C24	C27	F4	84.0(5)
C8	C11	C16	C15	-178.2(3)	C23	C24	C27	F5	-35.4(5)
C12	C11	C16	C15	0.2(5)	C23	C24	C27	F6	-155.4(4)
C11	C12	C13	C14	0.5(5)	C25	C24	C27	F4	-92.7(4)
C12	C13	C14	C15	0.8(5)	C25	C24	C27	F5	147.9(4)
C12	C13	C14	C17	-176.6(3)	C25	C24	C27	F6	27.9(5)
C13	C14	C15	C16	-1.5(5)	C24	C25	C26	C21	0.5(5)

Table 6. Anisotropic Displacement Parameters (U_{ij}, Å^2)

Atom	U_{11}	U_{22}	U_{33}	U_{23}	U_{13}	U_{12}
I1	0.0649(2)	0.05985(19)	0.05778(18)	-0.00224(13)	-0.00423(13)	0.03966(15)
Cl1	0.0568(6)	0.0547(6)	0.0632(6)	-0.0088(4)	-0.0079(5)	0.0370(5)
F1	0.0568(15)	0.093(2)	0.0664(16)	0.0457(15)	-0.0075(12)	0.0111(14)
F2	0.0518(15)	0.108(2)	0.0642(15)	0.0278(15)	-0.0060(12)	0.0371(15)
F3	0.119(3)	0.101(2)	0.0502(14)	-0.0153(14)	-0.0433(15)	0.0552(19)
F4	0.094(2)	0.0621(16)	0.0786(18)	-0.0101(13)	0.0501(16)	0.0178(15)
F5	0.096(2)	0.117(2)	0.0537(15)	-0.0472(16)	-0.0154(14)	0.0465(19)
F6	0.125(2)	0.0498(14)	0.0692(16)	-0.0104(12)	0.0246(16)	0.0431(16)
C1	0.0291(16)	0.0317(16)	0.0273(14)	-0.0016(12)	-0.0043(12)	0.0071(13)
C2	0.0315(16)	0.0314(16)	0.0308(15)	-0.0015(12)	-0.0015(12)	0.0120(13)
C3	0.0308(16)	0.0292(15)	0.0285(14)	-0.0035(12)	-0.0019(12)	0.0082(13)
C4	0.0355(17)	0.0354(17)	0.0315(15)	-0.0033(13)	-0.0088(13)	0.0133(14)
C5	0.0310(16)	0.0310(16)	0.0444(18)	-0.0036(13)	-0.0035(13)	0.0159(13)
C6	0.0358(17)	0.0330(16)	0.0322(16)	-0.0082(13)	-0.0025(13)	0.0115(14)
C7	0.0341(17)	0.0408(18)	0.0301(16)	-0.0026(13)	-0.0037(13)	0.0102(14)
C8	0.0341(17)	0.0430(19)	0.0329(16)	-0.0017(14)	0.0000(13)	0.0112(15)
C9	0.0375(18)	0.0338(17)	0.0330(16)	-0.0025(13)	-0.0013(13)	0.0117(14)
C10	0.0381(18)	0.0340(17)	0.0338(16)	-0.0014(13)	0.0006(13)	0.0102(14)
C11	0.0300(16)	0.0364(17)	0.0274(14)	0.0015(12)	-0.0021(12)	0.0079(13)
C12	0.0393(18)	0.0425(18)	0.0348(16)	-0.0050(14)	-0.0043(14)	0.0211(15)
C13	0.0431(19)	0.051(2)	0.0274(15)	-0.0073(14)	-0.0041(14)	0.0214(17)
C14	0.0341(17)	0.0388(18)	0.0307(15)	0.0031(13)	-0.0055(13)	0.0102(14)
C15	0.047(2)	0.0405(19)	0.0454(19)	-0.0010(15)	-0.0063(16)	0.0246(16)
C16	0.045(2)	0.0430(19)	0.0333(16)	-0.0087(14)	-0.0015(14)	0.0189(16)
C17	0.047(2)	0.055(2)	0.0401(19)	0.0076(17)	-0.0072(16)	0.0164(18)
C21	0.0334(16)	0.0299(15)	0.0292(15)	-0.0052(12)	0.0004(12)	0.0082(13)
C22	0.053(2)	0.0371(18)	0.0413(18)	-0.0089(14)	-0.0070(16)	0.0256(17)
C23	0.060(2)	0.048(2)	0.0287(16)	-0.0069(15)	-0.0109(15)	0.0248(18)
C24	0.0366(18)	0.0344(17)	0.0364(17)	-0.0075(14)	0.0047(14)	0.0097(14)
C25	0.044(2)	0.0389(18)	0.0475(19)	-0.0041(15)	0.0019(16)	0.0222(16)
C26	0.049(2)	0.0406(18)	0.0315(16)	-0.0018(14)	-0.0051(14)	0.0201(16)
C27	0.058(2)	0.046(2)	0.046(2)	-0.0108(17)	0.0130(18)	0.0147(19)

The form of the anisotropic displacement parameter is:
$\exp[-2\pi^2(h^2a^{*2}U_{11} + k^2b^{*2}U_{22} + l^2c^{*2}U_{33} + 2klb^*c^*U_{23} + 2hla^*c^*U_{13} + 2hka^*b^*U_{12})]$

Table 7. Derived Atomic Coordinates and Displacement Parameters for Hydrogen Atoms

Atom	*x*	*y*	*z*	U_{eq}, Å^2
H4	0.007930	0.884074	0.065037	0.041
H6	0.115507	1.009177	0.395615	0.041
H12	0.372872	0.993197	0.735534	0.045
H13	0.493120	0.962492	0.901033	0.048
H15	0.614870	0.670985	0.735828	0.051
H16	0.490584	0.696131	0.569793	0.048
H22	0.050579	0.642960	-0.257173	0.050
H23	0.072076	0.511694	-0.416921	0.054
H25	0.296429	0.325840	-0.226874	0.050
H26	0.276421	0.458271	-0.066027	0.047
